# Designed Synthesis of Unsymmetrical (Deuterated) 1,1-Diarylethylenes via Simple Sequential Coupling of Arenes and Aldehydes

**DOI:** 10.1021/acs.joc.6c00935

**Published:** 2026-05-29

**Authors:** Nicole Hanania, Rafael Snyder, Amit Garti, Ahmad Masarwa

**Affiliations:** Institute of Chemistry, The Center for Nanoscience and Nanotechnology, and Casali Center for Applied Chemistry, 98519 The Hebrew University of Jerusalem, Jerusalem 9190401,Israel

## Abstract

A practical and efficient method for synthesizing unsymmetrical (deuterated) 1,1-diarylethylenes is reported. The strategy relies on simple and selective sequential coupling processes in which (1) commercially available arenes react with aldehydes to form benzhydrylphosphonium salts in a regioselective manner, (2) followed by a chemoselective Wittig-type olefination with (deuterated) formaldehyde. This approach affords (deuterated) 1,1-diarylethylenes in good yields with high levels of isotopic incorporation. This protocol provides straightforward access to versatile building blocks of interest for synthetic and medicinal applications.

## Graphical abstract

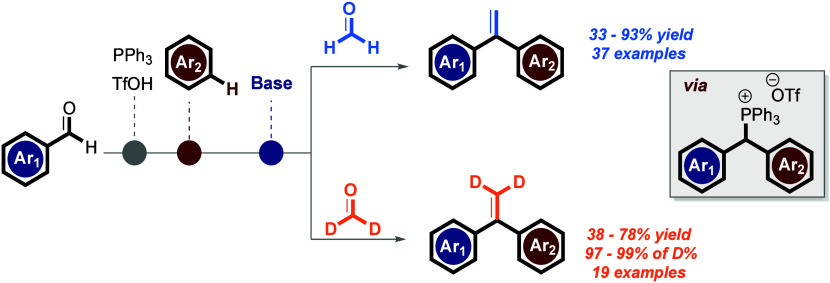

## Introduction

Diarylethylenes are an important class of molecules in modern organic synthesis^1–6^ and materials science,^7–9^ among which (unsymmetrical) 1,1-diarylethylenes have emerged as particularly significant due to their frequent presence in polymeric materials,^10–12^ as well as in pharmaceutically relevant and biologically active compounds (e.g., **I**, **II**, and **III**, Scheme 1A).^13–19^ Reflecting this importance, extensive efforts have been devoted to developing strategies for their synthesis, especially for their unsymmetrical variants.^20–24^ Representative methods include the hydroarylation (**b′**) and decarboxylative hydroarylation of alkynes with aryl boronic acids (**c**′****),^25,26^ C–H activation strategies based on cinnamic acid derivatives (**d**′****),^27^ and various transition-metal-catalyzed cross-coupling reactions.^28,29^ Among these methods are palladium-mediated, Kumada (**e**′****),^30,31^ Suzuki–Miyaura (**h**′****),^32^ and Stille couplings (**g**′****),^33,34^ all of which rely on diverse organometallic reagents as starting materials (Scheme 1B).^25–30,32,34,35^

**Scheme 1:**
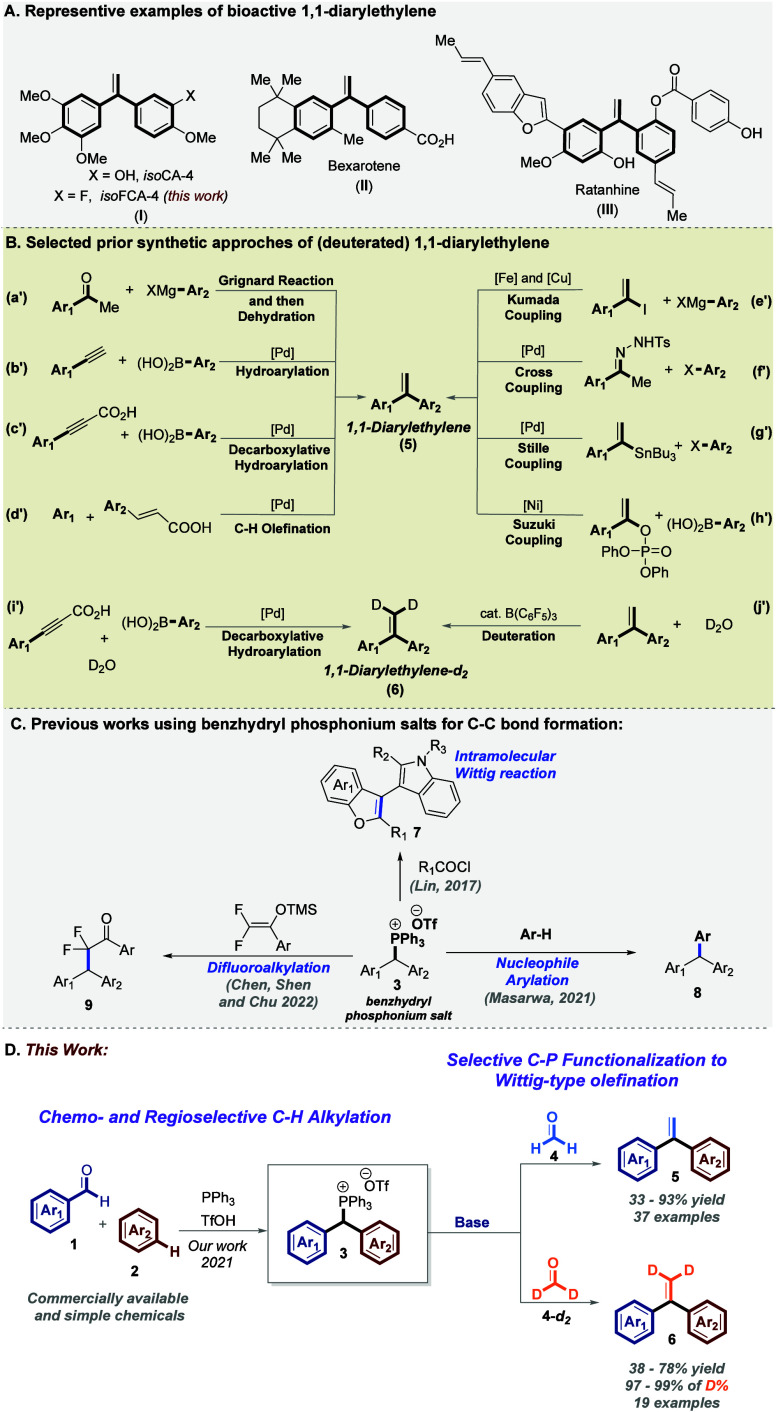
Overview of the Work

Although several methods exist for synthesizing 1,1-diarylethylenes (**5**), their deuterated analogs, 1,1-diarylethylenes-*d*_2_ (**6**), remain significantly more difficult to prepare.^25,36–38^ These isotopically labeled compounds could hold value for kinetic isotope effect studies and medicinal chemistry applications, where deuterium can enhance stability and metabolic profiles.^39–44^ Nevertheless, only a limited number of synthetic approaches to access these scaffolds have been reported (Scheme 1B).^25,37^ Thus, the development of straightforward and broadly applicable methods for synthesizing 1,1-diarylethylenes-*d*_2_ remains highly desirable.^39,41,44^

As part of our broader synthetic program aimed at accessing a wide range of diaryl-containing molecules, including our previous work on benzhydrylamines^45^ and benzhydryl thioethers,^46^ as well as our recent studies on diarylmethanes and diarylketones,^47^ we envisioned that unsymmetrical 1,1-diarylethenes (**5**) and their deuterated analogs (**6**) could be accessed from the same common precursors, namely, benzhydryl triarylphosphonium salts (**3**). This design underscores the potential of these salts as versatile surrogates of ylide intermediates in Wittig-type olefination reactions.^48^

In this context, benzhydryl triarylphosphonium salts (**3**) belong to the broader class of organophosphonium salts, characterized by the presence of a C–P moiety. These salts have long been employed in a wide range of synthetic transformations.^45–47,49–54^ More recently, strategies enabling the selective functionalization of the C­(sp^3^)–^+^PPh_3_ bond have emerged, allowing the construction of C–O, C–N, C–S, and C–C bonds.^45–47,49,50,55–59^

Notably, our group and others have reported several approaches that utilize benzhydryl phosphonium salts (**3**) as effective precursors for C–C bond formation (Scheme 1C).^45,51,56^ For example, Lin and co-workers described an acylation/intramolecular Wittig-reaction sequence between acyl chlorides and phosphonium salts to afford bis-heteroarene motifs (**7**, Scheme 1C).^51^ In addition, our group has developed a metal-free protocol for constructing triarylmethane scaffolds (**8**) via a Friedel–Crafts-like arylation of the benzylic C­(sp^3^)–^+^PPh_3_ bond, highlighting the ability of these benzhydryl phosphonium salts (**3**) to function as carbocation mimics.^45^ Later on, Chu and co-workers further demonstrated an efficient metal-free difluoroalkylation of benzhydryl triarylphosphonium salts (**3**) with difluoroenol silyl ethers.^56^ Importantly, these advances, together with the straightforward accessibility of benzhydryl phosphonium salts, have motivated the synthetic community toward developing complementary strategies that expand their derivatization scope and enable access to new molecular architectures.^45,47,52,55–57^

Inspired by these elegant studies, we investigated whether readily available benzhydryl triarylphosphonium salts (**3**) could be repurposed as Wittig-type reagents for olefination reactions, via intermolecular coupling with (deuterated) formaldehyde. In this way, this approach provides rapid access to (deuterated) 1,1-diarylethylenes (**5** and **6**) through only two programmable, sequential steps. In fact, the overall strategy can be viewed as a two-step coupling sequence of simple starting materials, i.e., aldehydes (**1**) and arenes (**2**).

Herein these steps include the following: (First) the formation of benzhydryl triarylphosphonium salts (**3**) as readily accessible synthetic platforms via a one-pot, regioselective four-component coupling from commercially available building blocks (**1**–**2**) and (second) their subsequent late-stage coupling with (deuterated) formaldehyde at the benzylic HC­(sp^3^)–^+^PPh_3_ position (Scheme 1D).

## Results and Discussion

To this end, we began by extending our earlier studies on the synthesis of benzhydryl triarylphosphonium salts (**3**).^45,47^ Accordingly, a new series of derivatives (**3a**–**3ak**) was prepared. This strategy enables the selective coupling of (hetero)­arenes (**2**) with aldehydes (**1**), affording the corresponding phosphonium salts (**3**). The aldehyde (**1**) could be first activated by PPh_3_ and TfOH, forming the (hydroxy­(phenyl)­methyl)­triphenylphosphonium trifluoromethanesulfonate intermediate (**3-Int**, see the SI, page S4), which subsequently undergoes coupling with arene derivatives (**2**) via a Friedel–Crafts-type reaction. The transformation proceeds under a simple and practical protocol using aldehyde (**1**), arene (**2**), PPh_3_, and TfOH in CH_3_CN at 45–80 °C for 24–72 h, resulting in the desired salts in up to 96% yield. In addition, the salts are purified by simple precipitation, providing a further practical advantage. Further details on their preparation are provided in the Supporting Information (pages S4–8).

Next, we explored the synthetic potential of these benzhydryl triarylphosphonium salts (**3**) as versatile building blocks through the selective vinylation of the benzylic C­(sp^3^)–P^+^ bond to access (unsymmetrical) 1,1-diarylethylenes (**5**). For that purpose, benzhydryl triarylphosphonium salt (**3p**) was used as the standard substrate for reaction optimization, subjecting it to the conditions of a Wittig-type olefination reaction with paraformaldehyde^60,61^ (**4**) (Scheme 2A,B). Screening different bases revealed that KO^*t*^Bu was the optimal base, successfully affording the desired 1,1-diarylethylene (**5j**) in 65% isolated yield after 2 h in DMSO at 60 °C (see the SI, Table S2, page S26). It is worth emphasizing that attempts to apply this transformation to other aldehydes and ketones under the same reaction conditions did not afford the corresponding olefination products. This outcome indicates that the reaction proceeds selectively with paraformaldehyde, which appears to be uniquely suited to engage this platform under the current conditions.

**Scheme 2:**
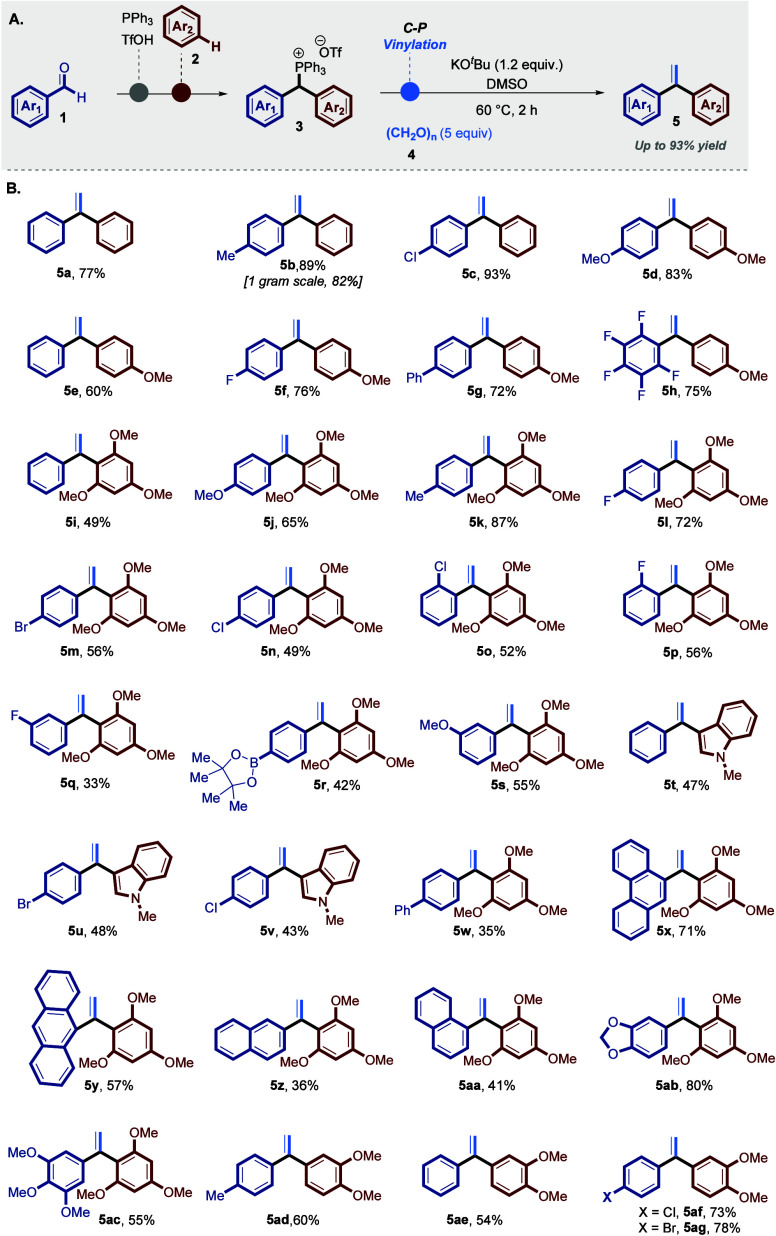
Synthesis of (Unsymmetrical) 1,1-Diarylethylenes^a^

a The reaction conditions: The reactions were carried out (prepared) in a glovebox. A mixture of **3** (0.20 mmol) and KO^*t*^Bu (0.24 mmol) in DMSO (2 mL) was stirred for 5 min, after which powdered paraformaldehyde **3** (1.0 mmol, 5 equiv) was added. The reaction mixture was then stirred at 60 °C for 2 h. The yields of isolated products are given.

After the optimal conditions were established, a series of benzhydryltriarylphosphonium salts (**3**) were reacted, efficiently affording a broad range of unsymmetrical 1,1-diarylethylenes (**5a**–**5ag**) (Scheme 2A,B). The reactions displayed wide substrate tolerance, with phosphonium salts bearing electron-donating substituents on the aryl ring (−Me, −OMe, −Ph) as well as the electron-withdrawing substituents (−F, −Cl, −Br, pentafluoro, and −Bpin) affording the corresponding products in good yields (**5b**–**5r**, Scheme 2B). Notably, heteroarene-containing indole-based phosphonium salts (**3w**–**3y**) also underwent a smooth conversion to the desired 1,1-diarylethylenes (**5t**–**5v**). Fused aromatic systems, including 1- and 2-naphthyl (**5z**–**5aa**), phenanthryl (**5x**), and anthracenyl (**5y**) derivatives, proved compatible with the protocol, highlighting its broad applicability toward extended π-systems. In addition, di- and trisubstituted aryl aldehydes afforded the expected products (**5ab**–**5ac**) in moderate yields, and a dimethoxy-substituted arene was also well tolerated, providing the desired products (**5ad**–**5ag**) in good yields.

Additionally, the vinylation of benzhydryltriphenylphosphoniumsalt **3h** was achieved, affording 1,1-diarylethylene **5b** on a gram scale (2.0 mmol, 82%) (for synthesis details, see page S33 of the Supporting Information).

Encouraged by these successful results, we anticipated that benzhydryl triarylphosphonium salts (**3**) could also serve as valuable precursors for synthesizing the elusive version of deuterium-labeled (unsymmetrical) 1,1-diarylethylenes-*d*_2_ (**6**) by incorporating two deuterium atoms into the vinylic position of a double bond (Scheme 3).

**Scheme 3:**
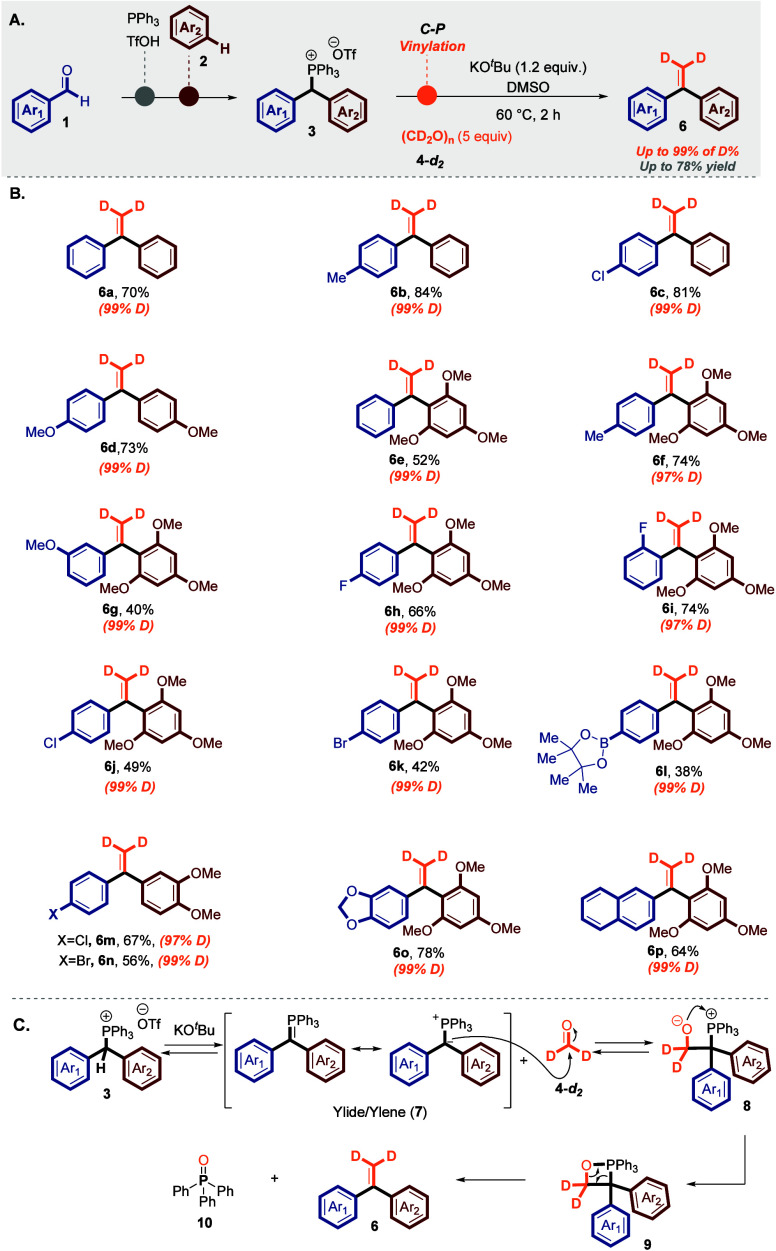
Synthesis of (Unsymmetrical) Deuterated 1,1-Diarylethylenes-*d*_2_ ^a^

a The reaction conditions: The reactions were carried out (prepared) in a glovebox. A mixture of **3** (0.20 mmol) and KO^*t*^Bu (0.24 mmol) in DMSO (2 mL) was stirred for 5 min, after which powdered deuterated paraformaldehyde-*d*_2_ (**4-** ***d***_***2***_) (1.0 mmol, 5 equiv) was added. The reaction mixture was then stirred at 60 °C for 2 h. The yields of isolated products are given. The percentage of D-incorporation for all deuterated molecules was assessed using ^1^H NMR.

As mentioned previously in the Introduction, although existing D_2_O-based methods, including decarboxylative hydroarylation^25^ and the direct deuteration of diarylethylenes,^37,38^ enable access to deuterated 1,1-diarylethylenes-*d*_*2*_ *,* we sought to develop a simpler and more direct approach to these frameworks using deuterated paraformaldehyde (**4-** ***d***_***2***_). Indeed, employing (**4-** ***d***_***2***_) as coupling partner provided fully deuterated (unsymmetrical) 1,1-diarylethylenes (**6**) with excellent isotopic incorporation (up to 99%) and good yields in a two-step coupling sequence (Scheme 3A,B).

A wide range of D-labeled (unsymmetrical) 1,1-diarylethylenes-*d*_2_ were synthesized, demonstrating a broad substrate scope and good functional group compatibility. Substituents such as −Me, −OMe, and −Cl (**6b**–**6g**) were well tolerated, as were ortho- and para-halogen substituents (−F, −Cl, and −Br, **6h**–**6k**). Furthermore, benzhydryl triphenylphosphonium salts bearing -Bpin (**6l**), −3,4-dimethoxy (**6m**–**6n**), −1,3-benzodioxole (**6o**), and naphthyl derivatives (**6p**) also underwent a smooth transformation, affording the desired products (**6l**–**6p**) in 38–78% yield.

A proposed mechanism for the Wittig-type vinylation of benzhydryltriarylphosphonium salts (**3**) is shown in Scheme 3C. The process begins with the abstraction of an acidic proton from the benzylic position of (**3**), generating the corresponding ylide species (**7**). The ylide (**7**) subsequently undergoes nucleophilic addition to (deuterated) formaldehyde (4-*d*_*2*_), possibly forming a betaine intermediate (**8**), which could rapidly cyclize to the corresponding oxaphosphetane (**9**).^62^ This intermediate then undergoes a retro-[2 + 2] cycloaddition to afford the desired (deuterated) 1,1-diarylethylene (**6**), along with triphenylphosphine oxide (**10**) as a byproduct whose formation was confirmed by ^31^P NMR (for further details, see Supporting Information, page S66).^24,48^

Notably, both aliphatic and heteroaliphatic aldehydes were compatible under the optimized conditions, affording the corresponding products (**5ah**–**5aj**) in 62–85% yield, including their deuterated analogs **6q** and **6r** (Scheme 4A). Furthermore, the methodology was successfully applied to the synthesis of **5ak** (*iso*FCA-4), a potent antimitotic agent,^63,64^ along with its corresponding deuterated analog **6s** (*iso*FCA-4)-*d*_2_ in 63% yield and 99% D-incorporation (Scheme 4B), underscoring the applicability of this strategy to biologically relevant targets.^65^

**Scheme 4:**
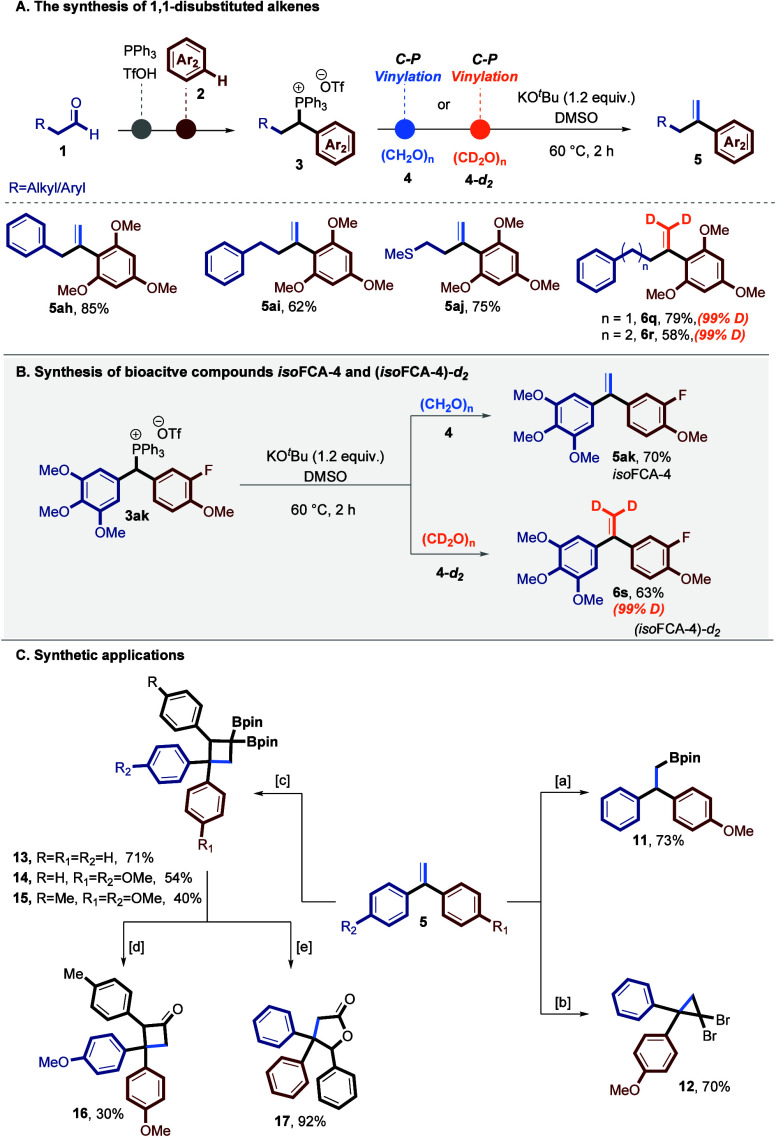
Scope with Aliphatic Substrates, Bioactive Target Synthesis, and Synthetic Applications^a^

a The reaction conditions: [a] HBpin (3.5 equiv), KO^*t*^Bu 10 mol %, toluene (2.5 M) at 130 °C, for 12 h. [b] CHBr_3_ (1.5 equiv), BnNEt_3_Cl 2 mol %, at rt for 1 h, followed by NaOH (50%) at 60 °C for 12 h. [c] *fac*-Ir­(ppy)_3_ (1 mol %)_,_ *gem*-diborylalkenes (**S-2**, 0.2 mmol), DCE (0.25 mL), 450 nm Blue LED, at rt for 48h. [d] Sodium perborate (NaBO_3_, 0.2 mmol), and H_2_O (1 mL). [e] THF (1 mL), H_2_O_2_ (0.5 mL, 30%) and NaOH (0.35 mL, 3 M).

Finally, postmodification of the 1,1-diarylethylenes showcased their synthetic versatility (Scheme 4C). For example, hydroboration of (**5e**) with HBpin and KO^*t*^Bu afforded (**11**) in 73% yield and cyclopropanation with CHBr_3_ smoothly afforded (**12**) in 70% yield. In addition, the photochemical [2 + 2] cycloaddition of the 1,1-diarylethylenes (**5**) with various *gem*-diborylalkenes (**S-2**) produced highly substituted cyclobutanes (**13**–**15**). Subsequent [2 + 2] cycloaddition, followed by two distinct oxidation protocols, afforded the corresponding cyclobutanone (**16**) in 30% yield and the γ-lactone (**17**) in 92% yield.

## Conclusions

In summary, we have developed a simple, transition-metal-free, two-step sequential protocol for synthesizing a diverse range of unsymmetrical (deuterated) 1,1-diarylethylenes, structural motifs found in many important molecules.^66^ This method features a site-selective, one-pot four-component coupling of commercially available aldehydes and arenes with PPh_3_ and TfOH, affording solid benzhydryl triarylphosphonium salts (**3**) without the need for further purification. These readily accessible intermediates are then reacted with (deuterated) paraformaldehyde under chemoselective Wittig-type conditions to construct new 1,1-diarylethylenes (**5** and **6**). The protocol tolerates a broad range of functional groups and delivers unsymmetrical 1,1-diarylethylenes in good yields. The method also enabled access to deuterated analogs (**6**) with high levels of deuterium incorporation, a particularly challenging class of compounds.

Overall, this strategy offers a streamlined and practical approach to pharmaceutically relevant 1,1-diarylethylene scaffolds and should facilitate their broader application in synthetic and medicinal chemistry.

## Supplementary Material



## Data Availability

The data underlying this study are available in the published article and its Supporting Information

## Notes

Complete experimental procedures and characterization of new products, synthetic transformations of the products, and NMR spectra (PDF)
