# Supplementary material for: Designed Synthesis of Unsymmetrical (Deuterated) 1,1-Diarylethylenes via Simple Sequential Coupling of Arenes and Aldehydes
Source: J Org Chem. 2026 May 29;91(23):8038–44. doi: 10.1021/acs.joc.6c00935 (PMC13270521; doi:10.1021/acs.joc.6c00935)
Supplement: Supplementary file 1 [file jo6c00935_si_001.pdf]

Supporting Information

The Designed Synthesis of Unsymmetrical (Deuterated) 1,1-Diarylethylenes via Simple Sequential Coupling of Arenes and Aldehydes

Nicole Hanania, Rafael Snyder,[+] Amit Garti,[+] and Ahmad Masarwa*

Institute of Chemistry, The Center for Nanoscience and Nanotechnology, and Casali Center for Applied Chemistry, The Hebrew University of Jerusalem, Jerusalem, 9190401, Israel

*Corresponding author: E-mail: Ahmad.Masarwa1@mail.huji.ac.il

Table of Contents

1. Notes……………………………………………….………………..…..……..……......S3

   1.1. Materials and General Remarks……………………...……………………..……....S3

2. Methods…………………………..……………………………………………..............S4

   2.1 General Procedure-A and Characterization for Phosphonium Salts (**3**)………………......S4

   2.2 General Procedure-B and Characterization for 1,1-Diaryletylene Products (**5**)…......….S25

   2.3 General Procedure-C and Characterization for 1,1-Diaryletylene-d_2 Products (**6**)...........S51

   2.4 Control experiment for the vinylation reaction of benzhydryl phosphonium salt (**3p**)....S66

   2.5 Procedures and Characterizations for Synthetic Application Products (**11-17**) ……......S68

3. NMR Spectra ……………..……...…………………………………………………...….S75

4. References…………………………………………………………………..…..………S162

1. Notes:

1.1. Materials and General Remarks

Unless stated otherwise, reactions were performed in oven-dried glassware/vials fitted with either rubber septa or plastic screw cap and were stirred with Teflon-coated magnetic stirring bars. All reagents were purchased from a commercial source and used as received unless otherwise noted. Trifluoromethanesulfonic acid (TfOH), PPh_3, aldehydes, arene, Paraformaldehyde (**4**) (Alfa Aesar) Paraformaldehyde-d_2 (**4-*d*₂**) (ARMAR Isotopes). Commercial grade solvents, i.e., acetonitrile, diethyl ether, *n*-pentane, ethyl acetate, dichloromethane, methanol, and ethanol were used as received unless mentioned otherwise. Thin-layer chromatography was performed using silica gel 60 F-254 precoated plates (0.25 mm) and was visualized by UV irradiation, CAM stain, $KMnO_4$ stain, and other stains. Silica gel of particle size 230 – 400 mesh was used for Column chromatography. ^{1}H and ^{13}C NMR spectra were recorded using 400 and 500 MHz spectrometers with ^{13}C operating frequencies of 100 and 125 MHz, respectively. ^{31}P and ^{19}F operation frequencies were 162 MHz, 203 MHz and 376 MHz, 471 MHz, respectively. Chemical shifts (δ) are reported in ppm relative to the residual solvent ($CDCl_3$) signal (δ = 7.26 for ^{1}H NMR and δ = 77.16 for ^{13}C NMR), (DMSO–d_6) signal (δ = 2.50 (p) for ^{1}H NMR; δ = 39.52 (septet) for ^{13}C NMR), (CD_3CN) signal (δ = 1.96 (p) for ^{1}H NMR; δ = 118.26 for ^{13}C NMR). Data for ^{1}H NMR spectra are reported as follows: chemical shift (multiplicity, coupling constants, and the number of hydrogen). Abbreviations are as follows: s (singlet), d (doublet), t (triplet), q (quartet), m (multiplet), dd (doublet of doublet), dt (doublet of triplet), ddd (doublet of doublet of doublet) brs (broad singlet). High-Resolution Mass Spectrometry (HRMS) were recorded on SCIEX X500R QTOF spectrometer using acetonitrile as solvent.

2. Methods

2.1 General Procedure-A and Characterization for Phosphonium Salts (3)

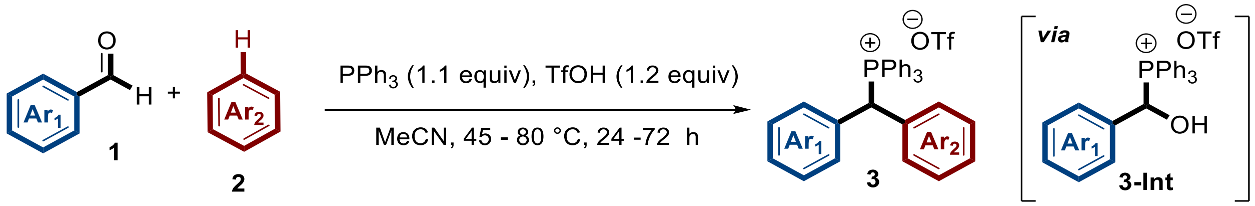

According to a literature-reported procedure,[1,2] an oven-dried, screw-capped 15 mL vial equipped with a Teflon-coated magnetic stirring bar was charged with PPh_3 (0.80 mmol, 1.1 equiv), MeCN (1 mL), and TfOH (0.87 mmol, 1.2 equiv). The reaction mixture was stirred at room temperature for 5 minutes. Subsequently, arene (**2**) (0.73 mmol, 1.0 equiv) and aldehyde (**1**) (0.73 mmol, 1.0 equiv) were added. The mixture was then stirred at either 45 °C or 80 °C in an oil bath for 24 -72 hours. Upon completion the solvent was removed under reduced pressure (by evaporator). The resulting crude product was dissolved in 1.5 mL of a 1:1:1 mixture of EtOH, EtOAc, and DCM, followed by the addition of 10 mL of a 5:1 (Et_2O:pentane) solution. The mixture was left to stand for precipitation. After standing, the excess solvent was decanted, and the precipitate was repeatedly washed with diethyl ether to yield the phosphonium salt (**3**), which was used in the subsequent step without further purification.

Notes: (1) In specific cases, the unpurified salts were used directly, without any further purification. (2) Compounds **3i**, **3k** and **3l** were stirred for 72 hours.

Table S1: Scope of the phosphonium salts (**3**):

Entry	Aldehydes (1)	Arenes (2)	Tempreature °C	Products (3)	Yield[a]
1.	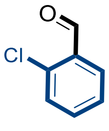 **1a**	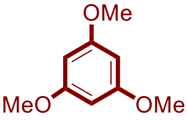 **2a**	80	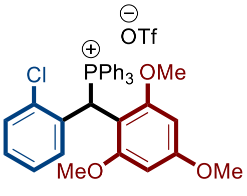 **3a**	87%
2.	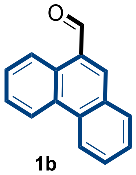 **1b**	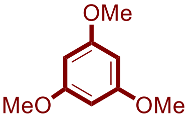 **2a**	80	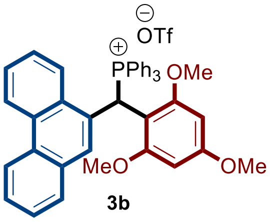 **3b**	62%
3.	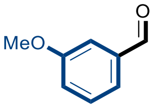 **1c**	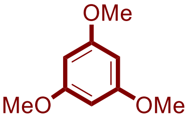 **2a**	80	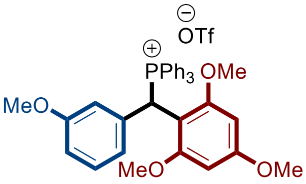 **3c**	86%
4.	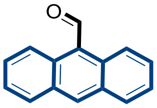 **1d**	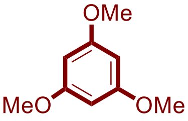 **2a**	80	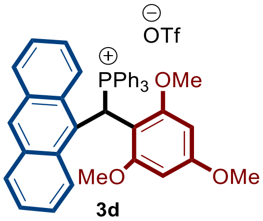 **3d**	63%
5.	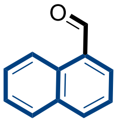 **1e**	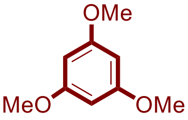 **2a**	80	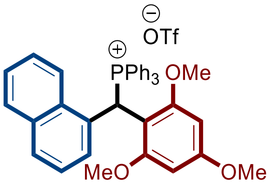 **3e**	69%
6.	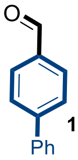 **1f**	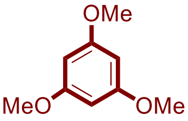 **2a**	80	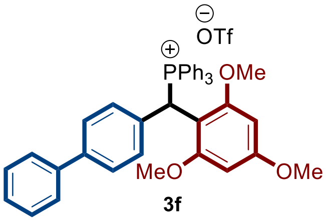 **3f**	81%

7.	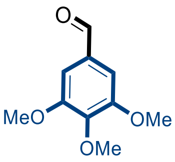 **1g**	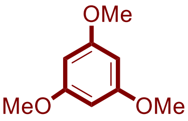 **2a**	80	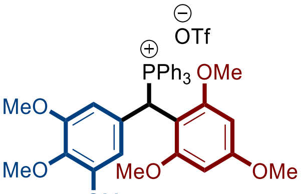 **3g**	95%
8.	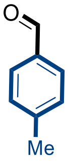 **1h**	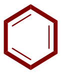 **2b**	45	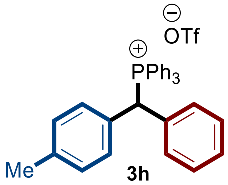 **3h**	84%
9.	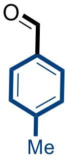 **1h**	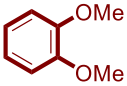 **2c**	80	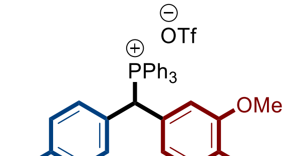 **3i**	57%
10.	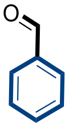 **1i**	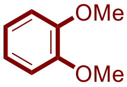 **2c**	80	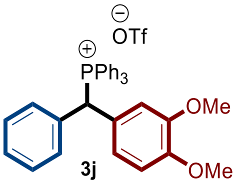 **3j**	93%
11.	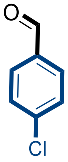 **1j**	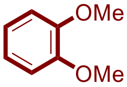 **2c**	80	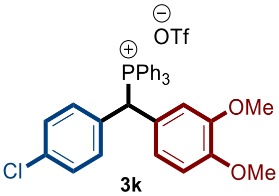 **3k**	88%
12.	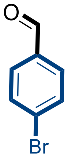 **1k**	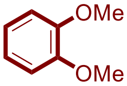 **2c**	80	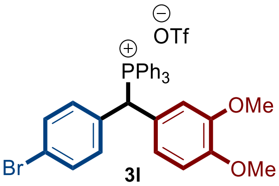 **3l**	85%
13.	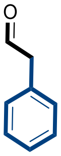 **1l**	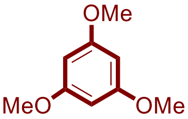 **2a**	80	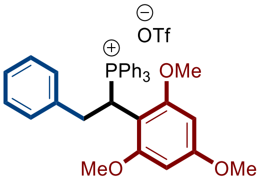 **3ah**	96%

14.	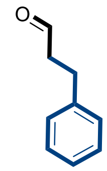 **1m**	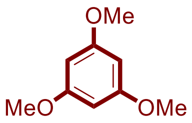 **2a**	80	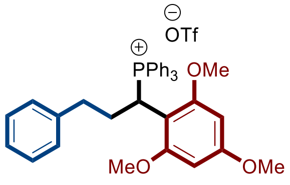 **3ai**	74%
15.	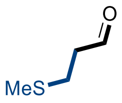 **1n**	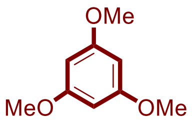 **2a**	80	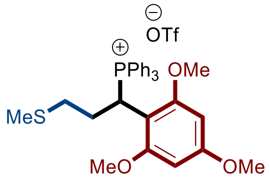 **3aj**	60%

[a] Isolated yield

Previously Reported benzhydryl phosphonium salts (3):

Note: Compounds **3m-3ag** were prepared following general Procedure-A. The spectroscopic data for the benzhydryl phosphonium salt align with those previously reported in the literature. [1-4]

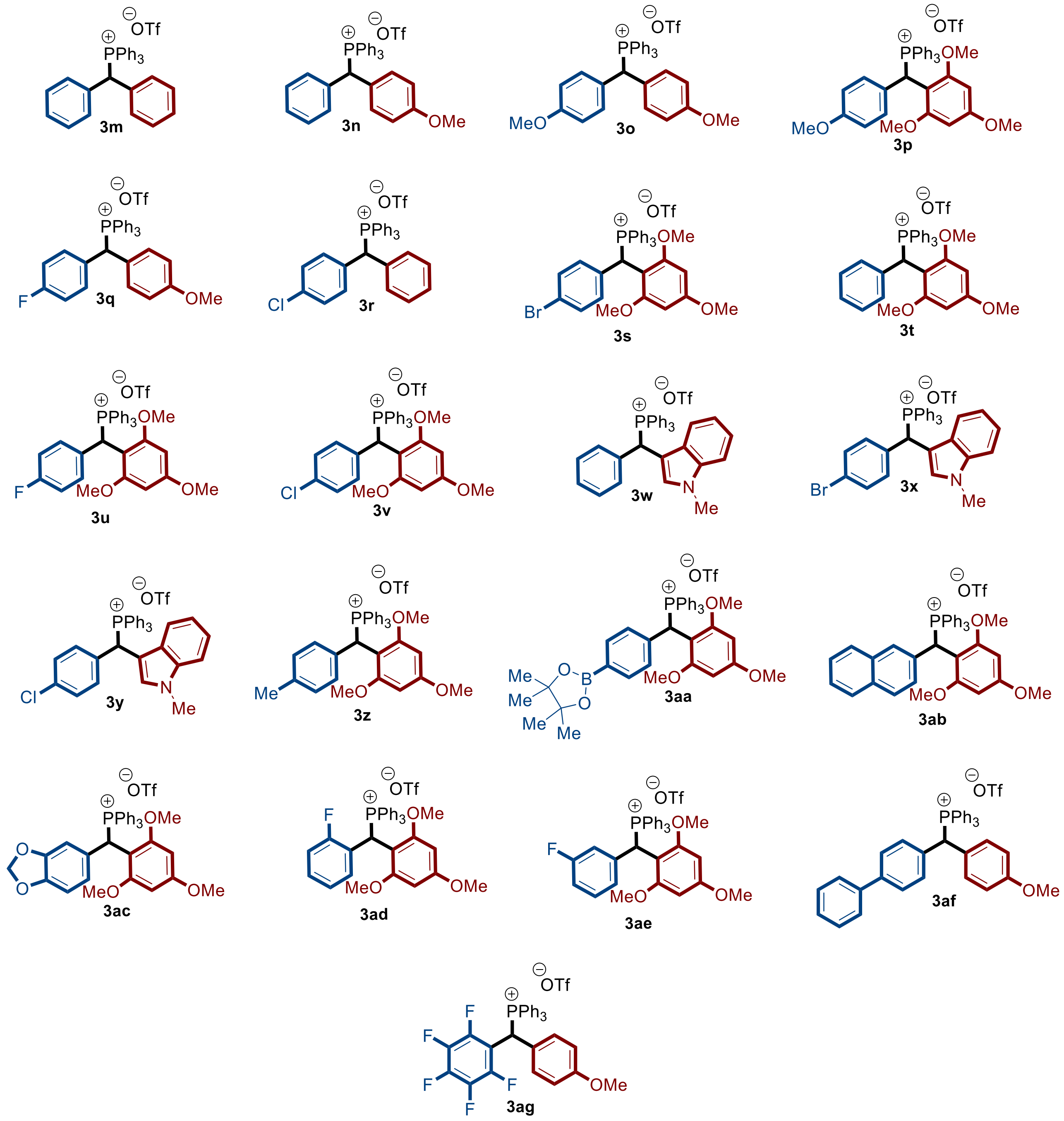

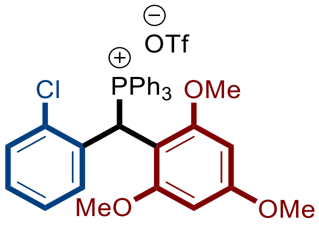

3a

((2-chlorophenyl)(2,4,6-trimethoxyphenyl)methyl)triphenylphosphonium trifluoromethanesulfonate (3a):

Prepared according to general Procedure-A using aldehyde (**1a**) (0.73 mmol, 102 mg) and arene (**2a**) (0.73 mmol, 122 mg), product (**3a**) was isolated in (445 mg, 87% yield) as a white solid.

^{1}H NMR (400 MHz, $CDCl_3$) δ: 7.75 – 7.67 (m, 3H), 7.61 – 7.51 (m, 6H), 7.51 – 7.42 (m, 7H), 7.19 – 7.08 (m, 4H), 6.09 (s, 2H), 3.82 (s, 3H), 3.51 (s, 6H).

^{13}C{^{1}H} NMR (101 MHz, $CDCl_3$) δ: δ 163.1 (d, J = 1.8 Hz), 158.5 (d, J = 5.6 Hz), 134.6 (d, J = 3.1 Hz), 134.3 (d, J = 7.5 Hz), 134.1 (d, J = 8.9 Hz), 133.0 (d, J = 4.6 Hz), 131.4, 130.2 (d, J = 2.8 Hz), 130.1 (d, J = 2.0 Hz), 129.8 (d, J = 12.2 Hz), 127.4 (d, J = 2.4 Hz), 120.6, 119.8, 100.9, 91.5, 55.8, 55.5, 36.8 (d, J = 48.7 Hz).

^{31}P NMR (162 MHz, $CDCl_3$) δ: 21.6.

^{19}F NMR (376 MHz, $CDCl_3$) δ: −78.1.

HRMS (ESI) was calculated for $[C_{34}H_{31}ClO_3P]^+$ $[M]^+$: m/z 553.1693, found = 553.1693.

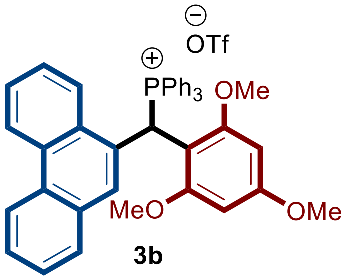

3b

phenanthren-9-yl(2,4,6-trimethoxyphenyl)methyl)triphenylphosphonium trifluoromethanesulfonate (3b):

Prepared according to general Procedure-A using aldehyde (**1b**) (0.73 mmol, 150 mg) and arene (**2a**) (0.73 mmol, 122 mg), product (**3b**) was isolated in (350 mg, 62% yield) as a white solid.

^{1}H NMR (400 MHz, $CDCl_3$) δ: 8.67 (dd, *J* = 32.5, 8.3 Hz, 2H), 8.07 (d, *J* = 8.3 Hz, 1H), 7.93 (d, *J* = 2.4 Hz, 1H), 7.73 (dq, *J* = 7.5, 3.0 Hz, 3H), 7.70 – 7.58 (m, 3H), 7.57 – 7.53 (m, 12H), 7.51 – 7.47 (m, 2H), 7.19 (d, *J* = 19.8 Hz, 1H), 6.08 (s, 2H), 3.80 (s, 3H), 3.46 (s, 6H).

^{13}C{^{1}H} NMR (101 MHz, $CDCl_3$) δ: 162.9, 158.8, 134.8 (d, *J* = 3.0 Hz), 134.5 (d, *J* = 8.9 Hz), 132.6 (d, *J* = 7.0 Hz), 130.9, 130.2, 129.9 (d, *J* = 12.1 Hz), 128.6, 128.1, 127.6, 127.4 (d, *J* = 2.0 Hz), 127.0, 123.7, 123.4, 122.6, 120.6, 119.8, 101.3, 91.6, 55.8, 55.4, 37.2 (d, *J* = 49.8 Hz).

^{31}P NMR (162 MHz, $CDCl_3$) δ: 22.7.

^{19}F NMR (376 MHz, $CDCl_3$) δ: −78.1

HRMS (ESI]) was calculated for $[C_{42}H_{36}O_3P]^+$ $[M]^+$: m/z 619.2396, found = 619.2375.

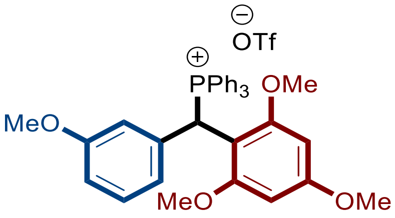

3c

3-methoxyphenyl)(2,4,6-trimethoxyphenyl)methyl)triphenylphosphonium trifluoromethanesulfonate (3c):

Prepared according to general Procedure-A using aldehyde (**1c**) (0.73 mmol, 100 mg) and arene (**2a**) (0.73 mmol, 122 mg), product (**3c**) was isolated in (435 mg, 86% yield) as a white solid.

^{1}H NMR (400 MHz, $CDCl_3$) δ: 7.82 – 7.72 (m, 3H), 7.60 (td, *J* = 8.0, 3.6 Hz, 6H), 7.39 – 7.30 (m, 6H), 7.11 (t, *J* = 8.0 Hz, 1H), 6.86 – 6.79 (m, 1H), 6.62 – 6.47 (m, 3H), 6.12 (d, *J* = 0.9 Hz, 2H), 3.84 (s, 3H), 3.55 (s, 9H).

^{13}C{^{1}H} NMR (101 MHz, $CDCl_3$): δ 162.9, 159.8 (d, *J* = 2.5 Hz), 158.6 (d, *J* = 5.8 Hz), 134.8 (d, *J* = 2.9 Hz), 134.2 (d, *J* = 8.8 Hz), 133.3 (d, *J* = 3.2 Hz), 130.0 (d, *J* = 12.1 Hz), 130.1, 122.3 (d, *J* = 4.9 Hz), 120.5, 119.7, 115.8 (d, *J* = 4.9 Hz), 114.4 (d, *J* = 3.1 Hz), 101.8 (d, *J* = 3.8 Hz), 91.3, 55.8, 55.2, 41.3 (d, *J* = 47.5 Hz).

^{31}P NMR (162 MHz, $CDCl_3$): δ 23.9.

^{19}F NMR (376 MHz, $CDCl_3$): δ −78.2.

HRMS (ESI) was calculated for $[C_{35}H_{34}O_4P+H]^+$ $[M+H]^+$: m/z 550.2267, found = 550.2247.

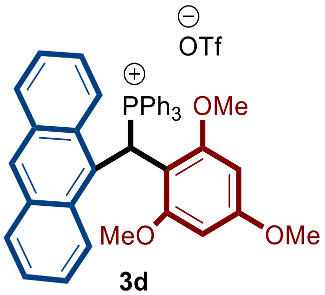

3d

anthracen-9-yl(2,4,6-trimethoxyphenyl)methyl)triphenylphosphonium trifluoromethanesulfonate (3d):

Prepared according to general Procedure-A using aldehyde (**1d**) (0.73 mmol, 150 mg) and arene (**2a**) (0.73 mmol, 122 mg), product (**3d**) was isolated in (485 mg, 63% yield) as a white solid.

^{1}H NMR (400 MHz, $CDCl_3$) δ: 7.79 – 7.67 (m, 5H), 7.58 – 7.44 (m, 9H), 7.25 – 7.16 (m, 8H), 7.12 – 7.01 (m, 3H), 6.55 (d, *J* = 2.0 Hz, 1H), 6.25 (d, *J* = 15.0 Hz, 1H), 6.13 (s, 2H), 3.87 (s, 3H), 3.65 (s, 6H).

^{13}C{^{1}H} NMR (101 MHz, $CDCl_3$) δ: 161.3, 158.4, 142.6, 137.9 (d, *J* = 5.6 Hz), 135.5 (d, *J* = 8.9 Hz), 135.3 (d, *J* = 3.0 Hz), 135.1 (d, *J* = 3.1 Hz), 133.9 (d, *J* = 9.7 Hz), 132.6 (d, *J* = 10.9 Hz), 131.2, 130.8 (d, *J* = 4.7 Hz), 130.0 (dd, *J* = 12.3, 6.2 Hz), 129.7 (d, *J* = 12.1 Hz), 128.3 (d, *J* = 4.2 Hz), 127.6 (d, *J* = 3.9 Hz), 127.3, 127.0 (d, *J* = 6.6 Hz), 126.8, 126.3 (d, *J* = 6.9 Hz), 125.9, 125.8, 123.7, 120.4 (d, *J* = 4.6 Hz), 116.7, 116.1, 90.8, 55.6, 55.5, 55.3, 48.2 (d, *J* = 38.9 Hz).

^{31}P NMR (162 MHz, $CDCl_3$) δ: 21.0.

^{19}F NMR (376 MHz, $CDCl_3$) δ: –78.1.

HRMS (ESI) was calculated for $[C_{42}H_{36}O_3P]^+$ $[M]^+$: m/z 619.2396, found = 619.2370.

Note: The NMR spectra show additional impurity signals, which we suspect correspond to (**3-Ind**). Compound **3d** was used for the next steps without further purification.

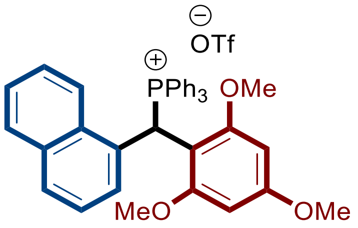

3e

naphthalen-1-yl(2,4,6-trimethoxyphenyl)methyl)triphenylphosphonium trifluoromethanesulfonate (3e):

Prepared according to general Procedure-A using aldehyde (**1e**) (0.73 mmol, 113 mg) and arene (**2a**) (0.73 mmol, 122 mg), product (**3e**) was isolated in (361 mg, 69% yield) as a white solid.

^{1}H NMR (400 MHz, $CDCl_3$) δ: 7.82 (ddd, *J* = 8.5, 7.0, 5.7 Hz, 3H), 7.77 – 7.68 (m, 3H), 7.65 (ddd, *J* = 7.4, 2.3, 1.1 Hz, 1H), 7.60 – 7.50 (m, 8H), 7.53 – 7.39 (m, 8H), 7.37 – 7.28 (m, 2H), 7.18 (d, *J* = 19.3 Hz, 1H), 6.07 (s, 2H), 3.81 (s, 3H), 3.43 (s, 6H).

^{13}C{^{1}H} NMR (101 MHz, $CDCl_3$) δ: 162.8 (d, *J* = 1.8 Hz), 158.5 (d, *J* = 5.5 Hz), 134.7 (d, *J* = 3.0 Hz), 134.4 (d, *J* = 8.9 Hz), 134.0, 132.3 (d, *J* = 10.5 Hz), 131.3 (d, *J* = 8.0 Hz), 130.7 (d, *J* = 6.4 Hz), 129.8 (d, *J* = 12.1 Hz), 129.8 (d, *J* = 2.2 Hz), 129.3, 129.2 (d, *J* = 2.0 Hz), 129.0 (d, *J* = 12.7 Hz), 127.1, 126.1, 124.7 (d, *J* = 2.2 Hz), 122.6, 120.7, 119.9, 119.3, 102.1 (d, *J* = 3.6 Hz), 91.4, 55.7, 55.3, 37.7 (d, *J* = 49.6 Hz).

^{31}P NMR (162 MHz, $CDCl_3$) δ: 22.4.

^{19}F NMR (376 MHz, $CDCl_3$) δ: −78.0.

HRMS (ESI) was calculated for $[C_{38}H_{34}O_3P+H]^+$ $[M+H]^+$: m/z 570.2318, found = 570.2295.

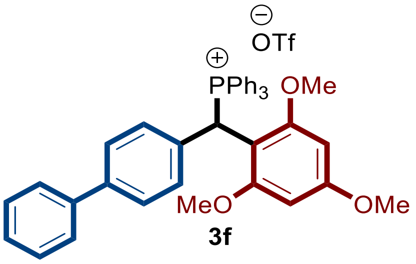

3f

[1,1'-biphenyl]-4-yl(2,4,6-trimethoxyphenyl)methyl)triphenylphosphonium trifluoromethanesulfonate (3f):

Prepared according to general Procedure-A using aldehyde (**1f**) (0.73 mmol, 132 mg) and arene (**2a**) (0.73 mmol, 122 mg), product (**3f**) was isolated in (440 mg, 81% yield) as a white solid.

^{1}H NMR (400 MHz, $CDCl_3$) δ 7.81 – 7.72 (m, 3H), 7.67 – 7.57 (m, 6H), 7.56 – 7.49 (m, 2H), 7.47 – 7.30 (m, 11H), 7.10 – 7.01 (m, 2H), 6.61 (d, J = 19.0 Hz, 1H), 6.14 (d, J = 0.8 Hz, 2H), 3.85 (s, 3H), 3.50 (brs, 6H).

^{13}C{^{1}H} NMR (101 MHz, $CDCl_3$) δ 162.9 (d, J = 1.6 Hz), 158.6 (d, J = 5.8 Hz), 141.6 (d, J = 3.3 Hz), 139.6, 134.9 (d, J = 3.0 Hz), 134.2 (d, J = 8.8 Hz), 130.9 (d, J = 3.3 Hz), 130.1 (d, J = 12.1 Hz), 129.0, 128.0, 127.5 (d, J = 2.7 Hz), 127.0, 122.7, 120.5, 119.7, 101.8 (d, J = 4.0 Hz), 91.3, 55.9, 41.2 (d, J = 47.2 Hz).

^{31}P NMR (162 MHz, $CDCl_3$) δ 23.8.

^{19}F NMR (376 MHz, $CDCl_3$) δ −78.2.

HRMS (ESI) was calculated for $[C_{40}H_{36}O_3P]^+$ $[M]^+$: m/z 595.2396, found = 595.2393.

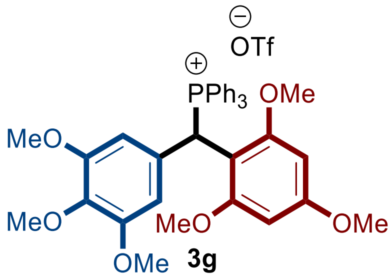

-triphenyl((2,4,6-trimethoxyphenyl)(3,4,5-trimethoxyphenyl)methyl)phosphonium trifluoromethanesulfonate (3g):

Prepared according to general Procedure-A using aldehyde (**1g**) (0.73 mmol, 143 mg) and arene (**2a**) (0.73 mmol, 122 mg), product (**3g**) was isolated in (530 mg, 95% yield) as a white solid.

^{1}H NMR (400 MHz, $CDCl_3$) δ: 7.77 (t, J = 7.6 Hz, 3H), 7.61 (tt, J = 6.9, 2.5 Hz, 6H), 7.40 – 7.28 (m, 6H), 6.51 (d, J = 18.9 Hz, 1H), 6.22 (s, 2H), 6.16 (s, 2H), 3.85 (s, 3H), 3.78 (s, 3H), 3.44 (brs, 12H).

^{13}C{^{1}H} NMR (101 MHz, $CDCl_3$) δ: 162.9, 158.7 (d, J = 5.7 Hz), 153.2 (d, J = 2.9 Hz), 138.4 (d, J = 4.0 Hz), 134.8 (d, J = 3.0 Hz), 134.2 (d, J = 8.8 Hz), 130.1 (d, J = 12.1 Hz), 126.8 (d, J = 3.5 Hz), 122.5, 120.4, 119.6, 107.5 (d, J = 4.9 Hz), 101.8 (d, J = 3.6 Hz), 91.3, 61.0 (d, J = 2.1 Hz), 55.9, 41.8 (d, J = 47.2 Hz).

^{31}P NMR (162 MHz, $CDCl_3$) δ: 23.3

^{19}F NMR (376 MHz, $CDCl_3$) δ: −78.1.

HRMS (ESI) was calculated for $[C_{37}H_{38}O_6P]^+$ $[M]^+$: m/z 609.2400, found = 609.2372.

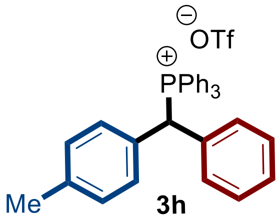

3h

triphenyl(phenyl(p-tolyl)methyl)phosphonium trifluoromethanesulfonate (3h):

Prepared according to general Procedure-A using aldehyde (**1h**) (0.73 mmol, 88 mg) and arene (**2b**) (0.73 mmol, 57 mg), product (**3h**) was isolated in (363 mg, 84% yield) as a white solid.

^{1}H NMR (400 MHz, $CDCl_3$) δ: 7.79 – 7.69 (m, 3H), 7.65 – 7.45 (m, 12H), 7.33 – 7.16 (m, 6H), 7.17 – 7.08 (m, 2H), 7.02 (dd, *J* = 8.2, 3.2 Hz, 2H), 6.70 (d, *J* = 17.4 Hz, 1H), 2.26 (s, 3H).

^{13}C{^{1}H} NMR (101 MHz, $CDCl_3$) δ: 139.1 (d, *J* = 2.9 Hz), 135.1, 134.9 (d, *J* = 9.2 Hz), 133.0 (d, *J* = 4.0 Hz), 130.7 (dd, *J* = 11.7, 7.0 Hz), 130.2 (d, *J* = 12.2 Hz), 130.0, 129.5 (d, *J* = 4.6 Hz), 129.3, 129.0 (d, *J* = 2.5 Hz), 127.5 (d, *J* = 4.6 Hz), 124.8, 122.3, 119.7, 118.6 – 118.4 (m), 117.9 (d, *J* = 5.7 Hz), 47.4 (d, *J* = 43.0 Hz), 21.

^{31}P NMR (162 MHz, $CDCl_3$) δ: 21.6.

^{19}F NMR (376 MHz, $CDCl_3$) δ: -78.1.

HRMS (ESI) was calculated for $[C_{32}H_{28}P+H]^{+}$ $[M+H]^{+}$: m/z 444.2001, found = 444.1978.

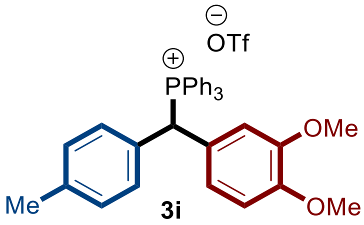

3i

((3,4-dimethoxyphenyl)(p-tolyl)methyl)triphenylphosphonium trifluoromethanesulfonate (3i):

Prepared according to general Procedure-A using aldehyde (**1h**) (0.73 mmol, 88 mg) and arene (**2c**) (0.73 mmol, 93 mg), product (**3i**) was isolated in (274 mg, 57% yield) as a beige solid.

^{1}H NMR (400 MHz, $CDCl_3$) δ: 7.83 – 7.72 (m, 5H), 7.66 – 7.45 (m, 21H), 7.13 – 6.99 (m, 7H), 6.95 – 6.88 (m, 2H), 6.71 (d, J = 8.4 Hz, 1H), 6.67 – 6.63 (m, 1H), 6.60 (d, J = 17.6 Hz, 1H), 3.81 (s, 3H), 3.59 (s, 3H), 2.27 (d, J = 1.8 Hz, 3H).

^{13}C{^{1}H} NMR (101 MHz, $CDCl_3$) δ: 171.9 (d, J = 3.6 Hz), 149.4 (d, J = 2.7 Hz), 149.3, 140.5 (d, J = 3.2 Hz), 139.1 (d, J = 2.9 Hz), 135.4 (d, J = 3.1 Hz), 135.0 (d, J = 3.1 Hz), 134.9 (d, J = 9.1 Hz), 134.8, 133.9 (d, J = 14.9 Hz), 132.1 (d, J = 10.3 Hz), 130.5 (d, J = 6.8 Hz), 130.3, 130.1 (d, J = 12.3 Hz), 130.0 (d, J = 1.8 Hz), 129.7 (d, J = 5.0 Hz), 128.9 (d, J = 12.4 Hz), 127.2, 124.8 (d, J = 4.1 Hz), 122.7 (d, J = 6.7 Hz), 122.3, 119.1, 118.8, 118.0, 116.77, 115.9, 114.3 (d, J = 7.3 Hz), 111.1 (d, J = 2.2 Hz), 56.0 (d, J = 3.3 Hz), 54.1 (d, J = 52.3 Hz), 47.1 (d, J = 42.7 Hz), 22.5, 21.3, 21.1.

^{31}P NMR (162 MHz, $CDCl_3$) δ: 21.4.

^{19}F NMR (376 MHz, $CDCl_3$) δ: -78.3.

HRMS (ESI) was calculated for $[C_{34}H_{32}O_2P]^+$ $[M]^+$: m/z 503.2134, found = 503.2113. **Note:** The NMR spectra show additional impurity signals, which we suspect correspond to (**3-Ind**). Compound **3i** was used for the next steps without further purification.

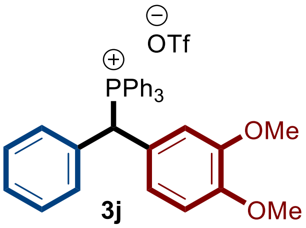

3j

((3,4-dimethoxyphenyl)(phenyl)methyl)triphenylphosphonium trifluoromethanesulfonate (3j):

Prepared according to general Procedure-A using aldehyde (**1i**) (0.73 mmol, 73 mg) and arene (**2c**) (0.73 mmol, 93 mg), product (**3j**) was isolated in (430 mg, 93% yield) as a white solid.

^{1}H NMR (400 MHz, $CDCl_3$) δ 7.78 – 7.71 (m, 3H), 7.62 – 7.47 (m, 13H), 7.25 – 7.15 (m, 4H), 6.95 (q, *J* = 2.2 Hz, 1H), 6.75 (d, *J* = 17.7 Hz, 1H), 6.72 – 6.63 (m, 2H), 3.81 (s, 3H), 3.61 (s, 3H).

^{13}C{^{1}H} NMR (101 MHz, $CDCl_3$) δ 149.4 (d, *J* = 2.7 Hz), 135.4 (d, *J* = 3.2 Hz), 135.0 (d, *J* = 3.0 Hz), 134.9, 134.8, 133.0 (d, *J* = 3.7 Hz), 130.7 (d, *J* = 6.8 Hz), 130.3, 130.1 (d, *J* = 12.1 Hz), 129.7 (d, *J* = 5.4 Hz), 129.5 (d, *J* = 2.2 Hz), 129.3 (d, *J* = 1.7 Hz), 129.0 (d, *J* = 2.5 Hz), 124.7 (d, *J* = 4.4 Hz), 122.7 (d, *J* = 6.5 Hz), 118.8, 118.0, 114.5 (d, *J* = 7.3 Hz), 111.1 (d, *J* = 2.2 Hz), 56.0, 55.9, 47.2 (d, *J* = 42.8 Hz).

^{31}P NMR (162 MHz, $CDCl_3$) δ: 23.8.

^{19}F NMR (376 MHz, $CDCl_3$) δ: -78.2.

HRMS (ESI) was calculated for $[C_{33}H_{30}O_2P]^+$ $[M]^+$: m/z 489.1977, found = 489.1960.

Note: The NMR spectra show additional impurity signals, which we suspect correspond to (**3-Ind**). Compound **3j** was used for the next steps without further purification.

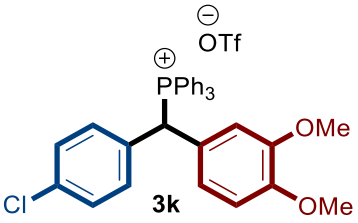

3k

(4-chloropheny)(3,4-dimethoxyphenyl)methyl)triphenylphosphonium trifluoromethanesulfonate (3k):

Prepared according to general Procedure-A using aldehyde (**1k**) (0.73 mmol, 102 mg) and arene (**2c**) (0.73 mmol, 93 mg), product (**3k**) was isolated in (435 mg, 88% yield) as a white solid.

^{1}H NMR (400 MHz, $CDCl_3$) δ 7.79 (dt, *J* = 22.8, 7.5 Hz, 5H), 7.70 – 7.47 (m, 19H), 7.24 – 7.10 (m, 6H), 6.97 (d, *J* = 8.1 Hz, 2H), 6.84 (d, *J* = 17.9 Hz, 1H), 6.70 (d, *J* = 8.4 Hz, 1H), 6.60 (d, *J* = 8.5 Hz, 1H), 3.81 (s, 3H), 3.63 (s, 3H).

^{13}C{^{1}H} NMR (101 MHz, $CDCl_3$) δ 149.5, 136.4, 135.6 (d, *J* = 3.0 Hz), 135.2 (d, *J* = 3.2 Hz), 135.1, 134.9, 134.8, 134.7, 133.9 (d, *J* = 15.7 Hz), 132.1 (d, *J* = 6.5 Hz), 131.6 (d, *J* = 3.7 Hz), 131.2 (d, *J* = 5.3 Hz), 130.4, 130.3, 130.1, 129.6 (d, *J* = 2.3 Hz), 129.3, 124.5 (d, *J* = 4.3 Hz), 122.4 (d, *J* = 6.5 Hz), 122.3, 119.1, 118.5, 117.7, 116.3, 115.5, 114.5 (d, *J* = 7.6 Hz), 111.2, 56.1, 55.9, 46.2 (d, *J* = 43.3 Hz).

^{31}P NMR (162 MHz, $CDCl_3$) δ: 21.2.

^{19}F NMR (376 MHz, $CDCl_3$) δ: -78.2.

HRMS (ESI) was calculated for $[C_{33}H_{29}ClO_2P]^+$ $[M]^+$: m/z 523.1594, found = 523.1601. **Note:** The NMR spectra show additional impurity signals, which we suspect correspond to (**3-Ind**). Compound **3k** was used for the next steps without further purification.

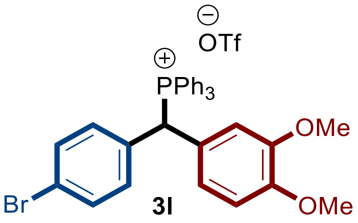

3l

(4-bromophenyl)(3,4-dimethoxyphenyl)methyl)triphenylphosphonium trifluoromethanesulfonate (3l):

Prepared according to general Procedure-A using aldehyde (**1k**) (0.73 mmol, 135 mg) and arene (**2c**) (0.73 mmol, 93 mg), product (**3l**) was isolated in (440 mg, 85% yield) as a white solid.

^{1}H NMR (400 MHz, $CDCl_3$) δ 7.86 – 7.69 (m, 5H), 7.70 – 7.47 (m, 19H), 7.42 – 7.28 (m, 3H), 7.18 – 7.11 (m, 2H), 7.10 – 7.03 (m, 1H), 6.98 – 6.91 (m, 1H), 6.81 (d, J = 17.8 Hz, 1H), 6.70 (d, J = 8.4 Hz, 1H), 6.59 (dt, J = 8.4, 2.2 Hz, 1H), 3.81 (s, 3H), 3.62 (s, 3H). **^{13}C{^{1}H} NMR** (101 MHz, $CDCl_3$) δ 149.5, 135.6 (d, J = 3.2 Hz), 135.2 (d, J = 3.0 Hz), 134.9, 134.8, 134.7, 132.6 (d, J = 2.2 Hz), 132.4, 132.3, 132.2 (d, J = 3.5 Hz), 131.4 (d, J = 5.3 Hz), 130.4, 130.3, 130.2, 124.7 (d, J = 3.8 Hz), 124.4 (d, J = 4.2 Hz), 123.4 (d, J = 3.3 Hz), 122.4 (d, J = 6.5 Hz), 122.3, 119.1, 118.5, 117.7, 116.3, 115.5, 114.5 (d, J = 7.5 Hz), 111.2, 56.0 (d, J = 11.3 Hz), 46.2 (d, J = 43.2 Hz).

^{31}P NMR (162 MHz, $CDCl_3$) δ: 21.2.

^{19}F NMR (376 MHz, $CDCl_3$) δ: -78.2.

HRMS (ESI) was calculated for $[C_{33}H_{29}BrO_2P+H]^+$ $[M+H]^+$: m/z 568.1167, found = 568.1159.

Note: The NMR spectra show additional impurity signals, which we suspect correspond to (**3-Ind**). Compound **3l** was used for the next steps without further purification.

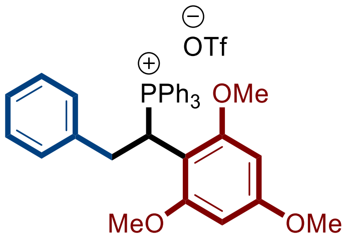

3ah

Triphenyl(2-phenyl-1-(2,4,6-trimethoxyphenyl)ethyl)phosphonium trifluoromethanesulfonate (3ah):

Prepared according to general Procedure-A using aldehyde (**1l**) (0.73 mmol, 87 mg) and arene (**2a**) (0.73 mmol, 122 mg), product (**3ah**) was isolated in (477 mg, 96% yield) as a white solid.

^{1}H NMR (400 MHz, $CDCl_3$) δ: 7.89 – 7.79 (m, 3H), 7.74 – 7.64 (m, 6H), 7.50 – 7.37 (m, 6H), 7.19 – 7.07 (m, 3H), 6.90 – 6.83 (m, 2H), 6.04 (d, J = 2.2 Hz, 1H), 5.82 (dd, J = 2.4, 1.1 Hz, 1H), 5.32 (ddd, J = 17.3, 11.7, 3.2 Hz, 1H), 3.85 (ddd, J = 13.0, 11.7, 5.3 Hz, 1H), 3.78 (s, 3H), 3.30 (ddd, J = 13.0, 7.5, 3.2 Hz, 1H), 3.24 (s, 3H), 3.18 (s, 3H).

^{13}C{^{1}H} NMR (101 MHz, $CDCl_3$) δ: 162.7, 159.8 (d, J = 3.7 Hz), 159.6 (d, J = 6.7 Hz), 137.2 (d, J = 15.1 Hz), 135.2 (d, J = 3.0 Hz), 134.1 (d, J = 9.0 Hz), 130.3 (d, J = 12.2 Hz), 128.6 (d, J = 2.5 Hz), 127.2, 119.0, 118.2, 98.9 (d, J = 4.7 Hz), 91.1 (d, J = 20.8 Hz), 56.1, 55.7, 54.8, 36.8 (d, J = 44.0 Hz), 33.9.

^{31}P NMR (162 MHz, $CDCl_3$) δ: 22.0.

^{19}F NMR (376 MHz, $CDCl_3$) δ: −78.0.

HRMS (ESI) was calculated for $[C_{35}H_{34}O_3P]^+$ $[M]^+$: m/z 533.2240, found = 533.2264.

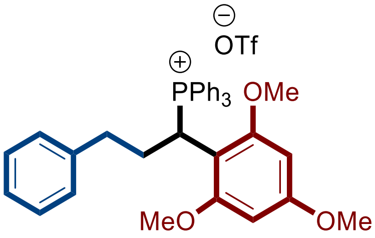

3ai

Triphenyl(3-phenyl-1-(2,4,6-trimethoxyphenyl)propyl)phosphonium trifluoromethanesulfonate (3ai):

Prepared according to general Procedure-A using aldehyde (**1m**) (0.73 mmol, 100 mg) and arene (**2a**) (0.73 mmol, 122 mg), product (**3ai**) was isolated in (520 mg, 74% yield) as a white solid.

^{1}**H NMR** (400 MHz, $CDCl_3$) δ: 7.83 – 7.72 (m, 3H), 7.63 – 7.49 (m, 6H), 7.33 – 7.25 (m, 3H), 7.24 – 7.15 (m, 6H), 7.00 (dd, *J* = 7.4, 2.0 Hz, 2H), 6.09 (d, *J* = 2.2 Hz, 1H), 6.01 (d, *J* = 1.9 Hz, 1H), 4.99 – 4.85 (m, 1H), 3.86 (s, 3H), 3.34 (s, 3H), 3.30 – 3.19 (m, 1H), 3.19 (s, 3H), 2.85 – 2.73 (m, 1H), 2.42 – 2.27 (m, 1H), 2.29 – 2.13 (m, 1H).

^{13}**C{^{1}H} NMR** (101 MHz, $CDCl_3$) δ: 163.0 (d, *J* = 2.9 Hz), 160.5 (d, *J* = 4.0 Hz), 159.8 (d, *J* = 6.6 Hz), 139.8, 135.0 (d, *J* = 3.0 Hz), 134.0 (d, *J* = 8.9 Hz), 130.1 (d, *J* = 12.1 Hz), 129.2, 128.6, 126.8, 118.4 (d, *J* = 82.5 Hz), 97.9 (d, *J* = 5.5 Hz), 91.0 (d, *J* = 17.3 Hz), 55.8, 55.7, 54.8, 33.5 (d, *J* = 6.9 Hz), 33.2 (d, *J* = 25.0 Hz), 29.6.

^{31}**P NMR** (162 MHz, $CDCl_3$) δ: 21.5

^{19}**F NMR** (376 MHz, $CDCl_3$) δ: −78.0.

HRMS (ESI) was calculated for $[C_{36}H_{36}O_3P]^+$ $[M]^+$: m/z 547.2396, found = 547.2397.

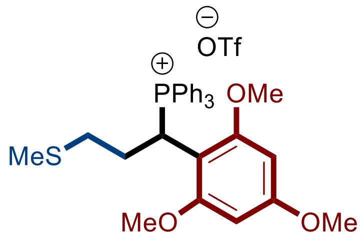

3aj

3-(methylthio)-1-(2,4,6-trimethoxyphenyl)propyl)triphenylphosphonium trifluoromethanesulfonate (3aj):

Prepared according to general Procedure-A using aldehyde (**1n**) (0.73 mmol, 69 mg) and arene (**2a**) (0.73 mmol, 122 mg), product (**3aj**) was isolated in (400 mg, 60% yield) as a white solid.

^{1}**H NMR** (400 MHz, $CDCl_3$) δ: 7.87 – 7.76 (m, 3H), 7.69 – 7.61 (m, 6H), 7.48 – 7.36 (m, 6H), 6.02 (dd, *J* = 22.1, 1.7 Hz, 2H), 5.52 (ddd, *J* = 18.0, 11.8, 2.3 Hz, 1H), 3.83 (s, 3H), 3.42 (s, 3H), 3.15 (s, 3H), 3.07 (ddq, *J* = 12.5, 8.7, 4.2 Hz, 1H), 2.62 – 2.52 (m, 1H), 2.28 – 2.09 (m, 2H), 1.95 (s, 3H).

^{13}**C{^{1}H} NMR** (101 MHz, $CDCl_3$) δ: 163.0 (d, *J* = 2.8 Hz), 160.2, 135.0 (d, *J* = 3.0 Hz), 134.2 (d, *J* = 9.0 Hz), 130.2 (d, *J* = 12.0 Hz), 119.0, 118.2, 97.9 (d, *J* = 5.2 Hz), 91.1, 56.1, 55.8, 54.8, 33.2 (d, *J* = 47.6 Hz), 32.6 (d, *J* = 15.0 Hz), 26.9, 15.7.

^{31}**P NMR** (162 MHz, $CDCl_3$) δ: 22.0.

^{19}**F NMR** (376 MHz, $CDCl_3$) δ: −78.0.

HRMS (ESI) was calculated for $[C_{31}H_{34}O_3PS]^+$ $[M]^+$: m/z 517.1960, found = 517.1985.

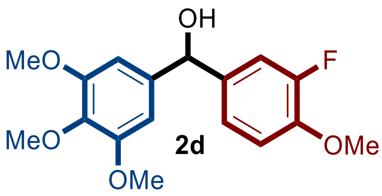

2d

(3-fluoro-4-methoxyphenyl)(3,4,5-trimethoxyphenyl)methanol (2d):

To a cold (-78 °C) solution of 4-bromo-2-fluoro-1-methoxybenzene (1 equiv, 10 mmol, 2.0 g) in THF (50 mL) inside a 250 mL Schlenk tube under N_2, *n*-BuLi (1.6M in hexane) (1.2 equiv, 12 mmol, 7.5 mL) was added in a dropwise addition. The reaction mixture was stirred for 1 hour at -78 °C. Then, a of solution of 3,4,5-trimethoxybenzaldehyde (0.8 equivalent, 8 mmol, 1.56 g) in dry THF (5 mL) was added to the reaction mixture at -78 °C. The reaction mixture was stirred for 2 h at −78 °C, then allowed to warm to room temperature and stirred for an additional 1h. The reaction mixture was concentrated under reduced pressure to remove THF. The residue was dissolved in DCM (30 mL), washed with water (20 mL) and brine (10 mL), dried over Na_2SO_4, filtered, and concentrated under reduced pressure. The crude material was purified by column chromatography on silica gel (EtOAc/hexane = 20:80) to afford the desired product as a white solid (740 mg, 29% yield).

R_f = 0.43 (50% EtOAc in Hexane).

^{1}H NMR (400 MHz, $CDCl_3$) δ: 7.17 – 7.02 (m, 2H), 6.92 (t, *J* = 8.4 Hz, 1H), 6.57 (d, *J* = 2.1 Hz, 2H), 5.69 (s, 1H), 3.87 (s, 3H), 3.83 (d, *J* = 1.2 Hz, 9H).

^{13}C{^{1}H} NMR (101 MHz, $CDCl_3$) δ: 153.47, 151.21, 147.17 (d, *J* = 10.6 Hz), 139.31, 137.51, 136.90 (d, *J* = 5.6 Hz), 122.39 (d, *J* = 3.6 Hz), 114.57 (d, *J* = 19.1 Hz), 113.34 (d, *J* = 2.1 Hz), 103.53, 75.56, 60.97, 56.44, 56.25.

^{19}F NMR (376 MHz, $CDCl_3$) δ: -134.6.

HRMS (ESI) was calculated for $[C_{17}H_{18}FO_4\text{-OH}]^+$ $[M\text{-OH}]^+$: m/z 305.1184, found = 305.1151.

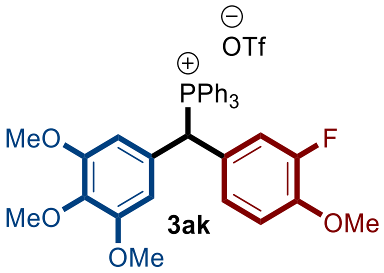

3ak

((3-fluoro-4-methoxyphenyl)(3,4,5-trimethoxyphenyl)methyl)triphenylphosphonium trifluoromethanesulfonate (3ak):

an oven-dried, screw-capped 15 mL vial equipped with a Teflon-coated magnetic stirring bar was charged with PPh_3 (0.80 mmol, 1.1 equiv), MeCN (1 mL), and TfOH (0.87 mmol, 1.2 equiv). The reaction mixture was stirred at room temperature for 5 minutes. Subsequently, (**2d**) (0.73 mmol, 235 mg) was added. The mixture was then stirred at 80 °C in an oil bath for 24 hours. Upon completion the solvent was removed under reduced pressure (by evaporator). The resulting crude product was dissolved in 1.5 mL of a 1:1:1 mixture of EtOH, EtOAc, and DCM, followed by the addition of 10 mL of a 5:1 (Et_2O:pentane) solution. The mixture was left to stand for precipitation. After standing, the excess solvent was decanted, and the precipitate was repeatedly washed with diethyl ether to yield the phosphonium salt product (**3ak**) was isolated in (468 mg, 65% yield) as a beige solid.

^{1}H NMR (400 MHz, $CDCl_3$) δ: 7.84 – 7.73 (m, 3H), 7.65 – 7.52 (m, 12H), 7.16 – 7.09 (m, 1H), 6.92 – 6.80 (m, 2H), 6.72 (d, J = 17.6 Hz, 1H), 6.47 (d, J = 1.6 Hz, 2H), 3.85 (s, 3H), 3.80 (s, 3H), 3.56 (s, 6H).

^{13}C{^{1}H} NMR (101 MHz, $CDCl_3$) δ: 153.7 (d, J = 1.8 Hz), 150.7, 148.3 (d, J = 10.5 Hz), 138.5 (d, J = 3.0 Hz), 135.3 (d, J = 3.2 Hz), 134.9 (d, J = 9.2 Hz), 134.0 (d, J = 14.3 Hz), 130.3 (d, J = 12.3 Hz), 128.7 – 126.0 (m), 124.8 (d, J = 5.6 Hz), 122.1, 118.2 (d, J = 82.1 Hz), 113.9, 108.0 (d, J = 7.3 Hz), 65.9, 61.0, 56.3, 56.2, 46.6 (d, J = 43.1 Hz).

^{31}P NMR (162 MHz, $CDCl_3$) δ: 21.4.

^{19}F NMR (376 MHz, $CDCl_3$) δ: -78.3, -132.5.

HRMS (ESI) was calculated for $[C_{35}H_{33}FO_4P]^+$ $[M]^+$: m/z 567.2095, found = 567.2082.

2.2. General Procedure-B and Characterization for 1,1-diarylethylene products (5).

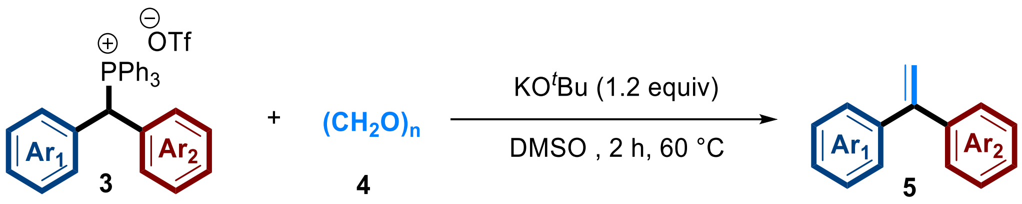

A 15 mL screw-cap vial containing a Teflon-coated magnetic stirring bar was charged with benzhydryl phosphonium salt **3** (0.2 mmol, 1.0 equiv), and taken inside the glovebox. Then, 2 mL of DMSO and KO^{t}Bu (0.24 mmol, 1.2 equiv) were added, and the mixture was stirred at room temperature for five minutes. After that, powder paraformaldehyde **4** (1.0 mmol, 5 equiv) was added, and the vial was sealed and taken out of the glove box to stir at 60 °C for 2 hours. After cooling to room temperature, the mixture was quenched with H_2O, and the aqueous phase was extracted with EtOAc (2 x 5 mL). The combined organic phases were washed with brine, dried over Na_2SO_4, filtered and concentrated under reduced pressure (by evaporator) to give the crude material, that was purified by column chromatography, to give the desired product (**5**).

Table - S2: Optimization table for 1,1-Diarylethylene (**5**) preparation.

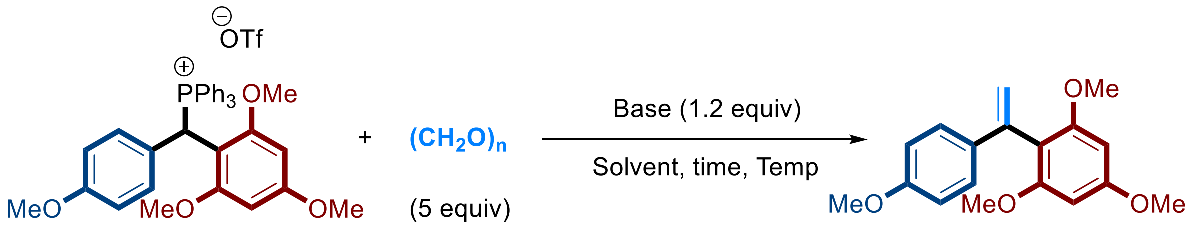

3p **4** **5j**

Entry	Solvent	Temperature (° C)	Base (Equiv.)	Time (hours)	5 (%)[b]
1.	THF	60	NaH (1.2)	4	43%
2.	THF	60	$NaNH_3$ (1.2)	4	NR
3.	THF	60	LDA (1.2)	4	24%
4.	THF	80	KO^{t}Bu (1.2)	4	50%
5.	Toluene	110	KO^{t}Bu (1.2)	4	20%[c]
6.	MeCN	60	KO^{t}Bu (1.2)	4	45%[c]
7.	THF	60	KO^{t}Bu (2.0)	4	NR
8.	DMSO	60	KO^{t}Bu (1.2)	4	55%
9.	DMSO	60	KO^{t}Bu (1.2)	2	60%
**10.**	**DMSO**	**60**	**KO^{t}Bu (1.2)**	**2**	**65%[c]**
11.	DMSO	60	NaH (1.2)	4	NR
12.	DMSO	60	NaO^{t}Bu (1.2)	4	10%[c]

Reaction conditions: [a] All reactions were carried out using 0.2 mmol of benzhydryl phosphonium salt **3p** under air. [b] All reported yields are isolated yields. [c] The reaction was carried out in the glove box, under N_2. NR = No reaction was observed at all.

Table - S3: Scope of the 1,1-Diarylethylene preparation (**5**).

Entry	Phosphonium Salts (3)	1,1-Diarylethylene (5)	Yield[a]
1.	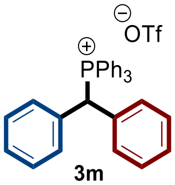 **3m**	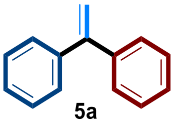 **5a**	77%
2.	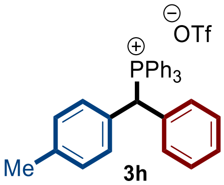 **3h**	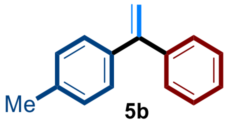 **5b**	89%
3.	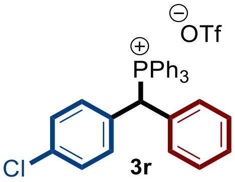 **3r**	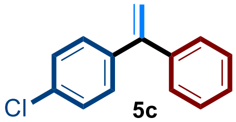 **5c**	93%
4.	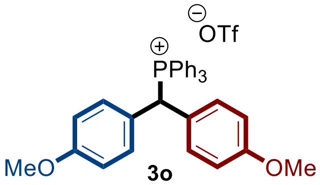 **3o**	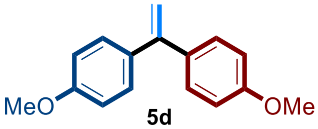 **5d**	83%
5.	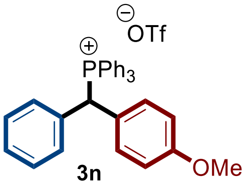 **3n**	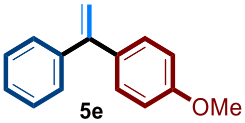 **5e**	60%
6.	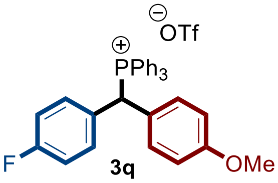 **3q**	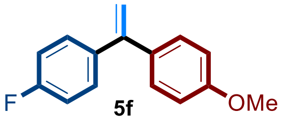 **5f**	76%

7.	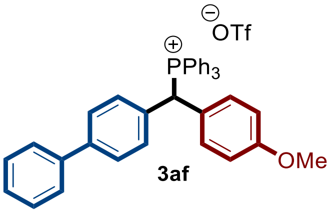 **3af**	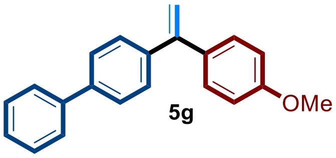 **5g**	72%
8.	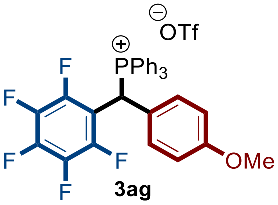 **3ag**	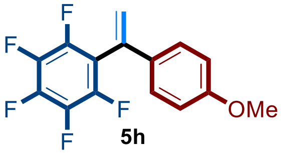 **5h**	75%
9.	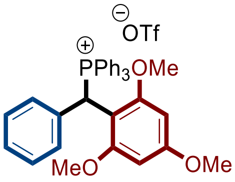 **3t**	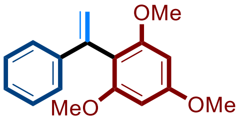 **5i**	49%
10.	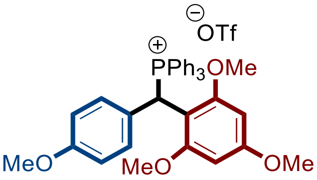 **3p**	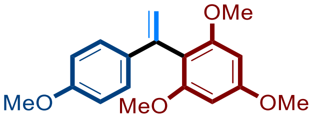 **5j**	65%
11.	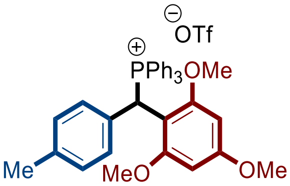 **3z**	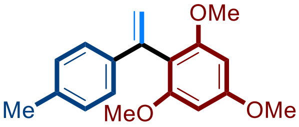 **5k**	87%
12.	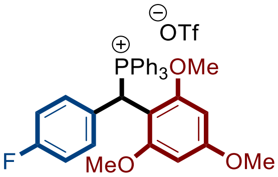 **3u**	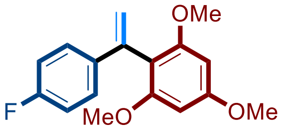 **5l**	72%

13.	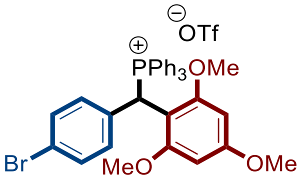 **3s**	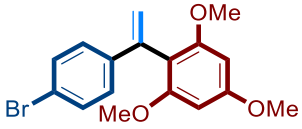 **5m**	56%
14.	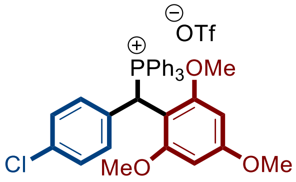 **3v**	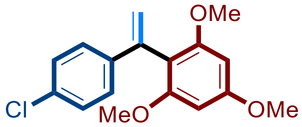 **5n**	49%
15.	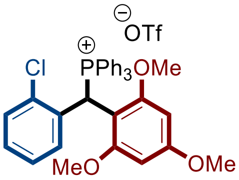 **3a**	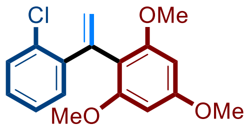 **5o**	52%
16.	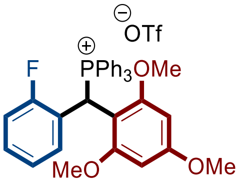 **3ad**	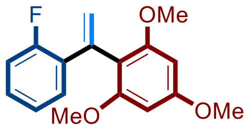 **5p**	56%
17.	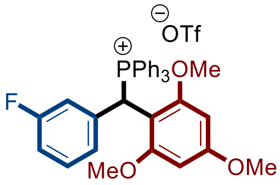 **3ae**	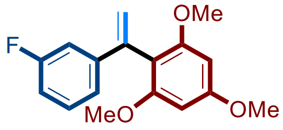 **5q**	33%
18.	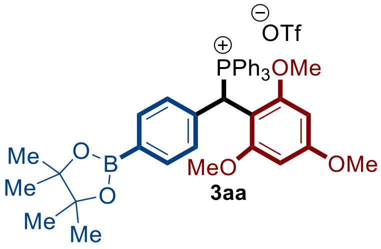 **3aa**	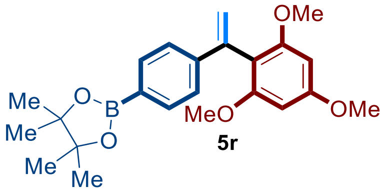 **5r**	42%

19.	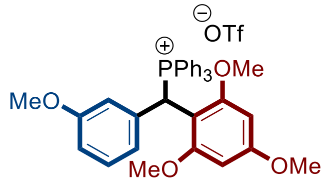<br>**3c**	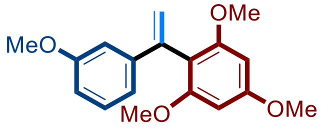<br>**5s**	55%
20.	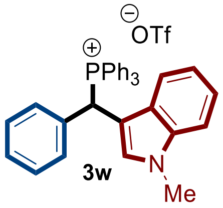<br>**3w**	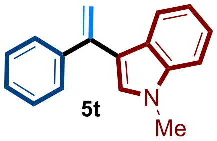<br>**5t**	47%
21.	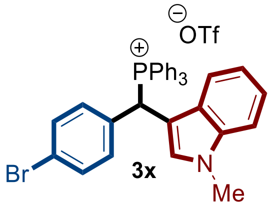<br>**3x**	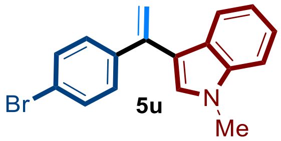<br>**5u**	48%
22.	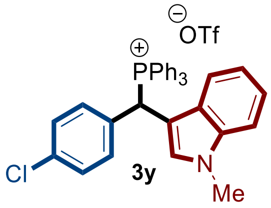<br>**3y**	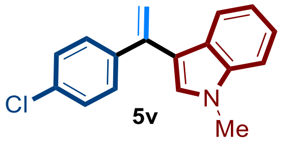<br>**5v**	43%
23.	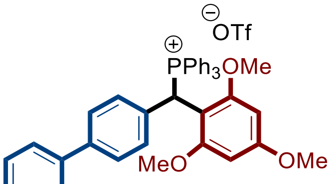<br>**3f**	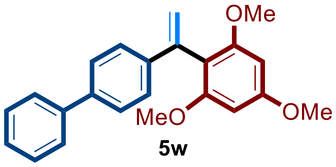<br>**5w**	35%
24.	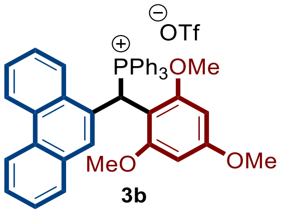<br>**3b**	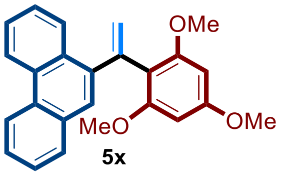<br>**5x**	71%

25.	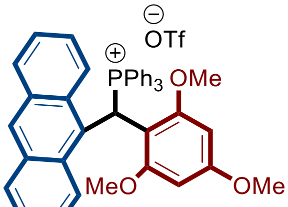 **3d**	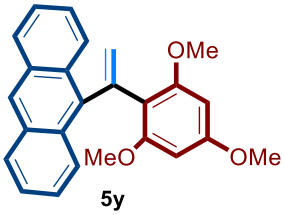 **5y**	57%
26.	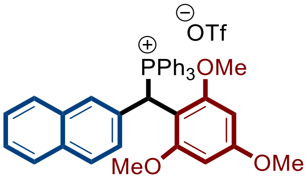 **3ab**	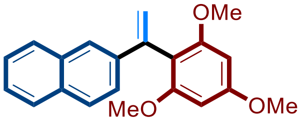 **5z**	36%
27.	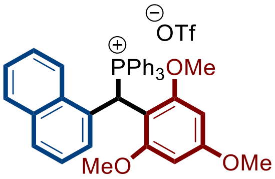 **3e**	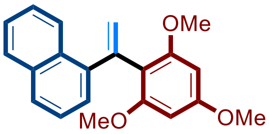 **5aa**	41%
28.	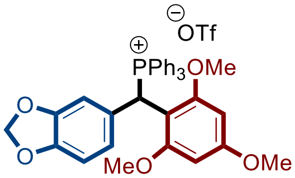 **3ac**	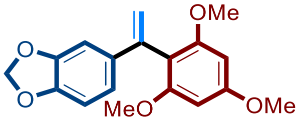 **5ab**	80%
29.	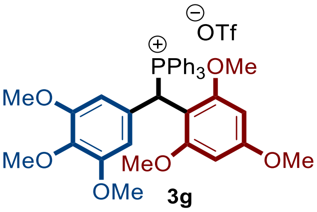 **3g**	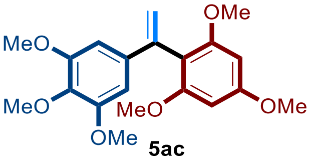 **5ac**	55%
30.	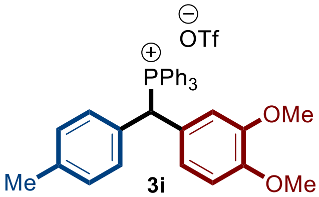 **3i**	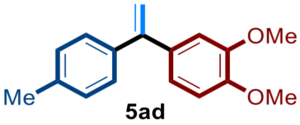 **5ad**	60%

31.	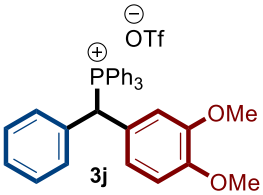 3j	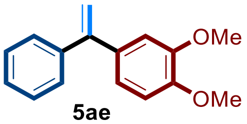 5ae	54%
32.	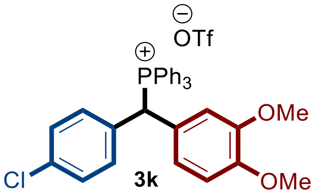 3k	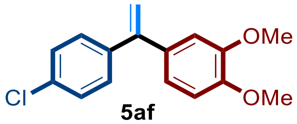 5af	73%
33.	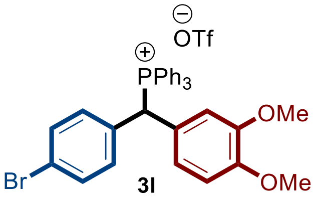 3l	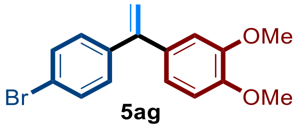 5ag	78%
34.	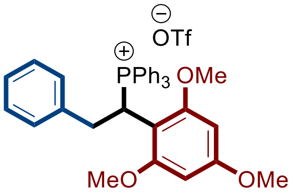 3ah	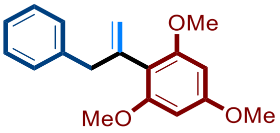 5ah	85%
35.	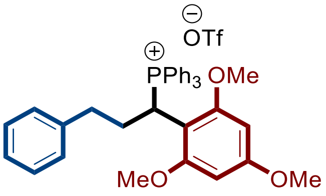 3ai	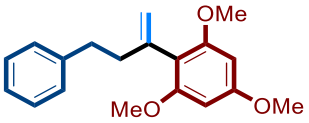 5ai	62%
36.	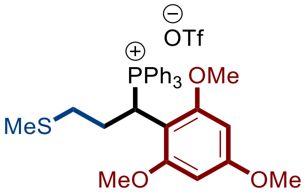 3aj	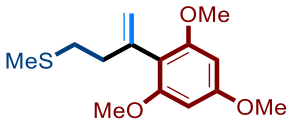 5aj	75%

37.	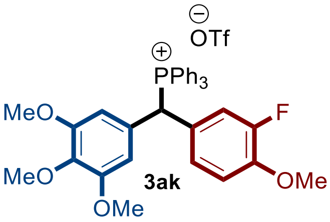 3ak	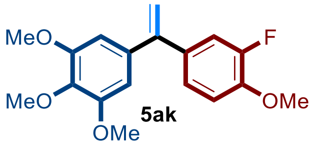 5ak	70%

[a] Isolated yield.

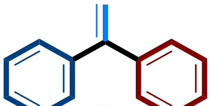

5a

Ethene-1,1-diyldibenzene (5a):

Prepared according to general Procedure-B using phosphonium salt (**3m**) (0.2 mmol, 115 mg) product (**5a**) was isolated in (28 mg, 77% yield) as a colorless oil by column chromatography on silica gel (EtOAc/Hexane = 02:98).

R_f = 0.75 (5% EtOAc in hexane).

^{1}H NMR (400 MHz, $CDCl_3$) δ 7.36 – 7.32 (m, 11H), 5.47 (s, 2H)

The spectral data are consistent with those reported in the literature.[5]

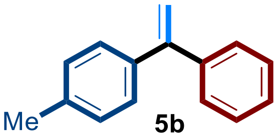

5b

1-methyl-4-(1-phenylvinyl)benzene (5b):

Prepared according to general Procedure-B using phosphonium salt (**3h**) (0.2 mmol, 120 mg) product (**5b**) was isolated in (34 mg, 89% yield) as a colorless oil by column chromatography on silica gel (EtOAc/Hexane = 02:98). A 1.0 -gram scale of **3h** reaction gave product **5b** (320 mg, 82% yield).

R_f = 0.73 (5% EtOAc in hexane).

^{1}H NMR (400 MHz, $CDCl_3$) δ: 7.39 (brs, 5H), 7.33 – 7.10 (m, 4H), 5.48 (d, J = 8.2 Hz, 2H), 2.42 (s, 3H).

The spectral data are consistent with those reported in the literature.[6]

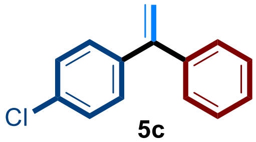

5c

1-chloro-4-(1-phenylvinyl)benzene (5c):

Prepared according to general Procedure-B using phosphonium salt (**3r**) (0.2 mmol, 122 mg) product (**5c**) was isolated in (40 mg, 93% yield) as a white solid by column chromatography on silica gel (EtOAc/Hexane = 02:98).

R_f = 0.65 (5% EtOAc in hexane).

^{1}**H NMR** (400 MHz, $CDCl_3$) δ: 7.45 – 7.21 (m, 9H), 5.49 (d, J = 7.5 Hz, 2H).

The spectral data are consistent with those reported in the literature.[7]

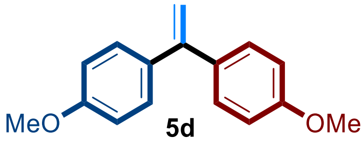

5d

4,4'-(ethene-1,1-diyl)bis(methoxybenzene) (5d):

Prepared according to general Procedure-B using phosphonium salt (**3o**) (0.2 mmol, 127 mg) product (**5d**) was isolated in (40 mg, 83% yield) as a white solid by column chromatography on silica gel (EtOAc/Hexane = 05:95).

R_f = 0.54 (10% EtOAc in hexane).

^{1}**H NMR** (400 MHz, $CDCl_3$) δ: 7.31 – 7.26 (m, 4H), 6.90 – 6.85 (m, 4H), 5.30 (s, 2H), 3.83 (s, 6H).

The spectral data are consistent with those reported in the literature.[8]

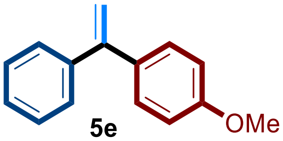

5e

1-methoxy-4-(1-phenylvinyl)benzene (5e):

Prepared according to general Procedure-B using phosphonium salt (**3n**) (0.2 mmol, 121 mg) product (**5e**) was isolated in (25 mg, 60% yield) as a white solid by column chromatography on silica gel (EtOAc/Hexane = 05:95).

R_f = 0.60 (10% EtOAc in hexane).

^{1}H NMR (400 MHz, $CDCl_3$) δ: 7.38 – 7.31 (m, 5H), 7.30 – 7.26 (m, 2H), 6.92 – 6.82 (m, 2H), 5.38 (dd, *J* = 17.2, 1.3 Hz, 2H), 3.83 (s, 3H).

The spectral data are consistent with those reported in the literature.[6]

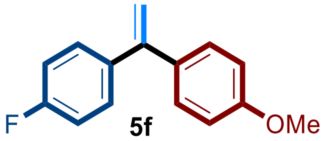

5f

1-fluoro-4-(1-(4-methoxyphenyl)vinyl)benzene (5f):

Prepared according to general Procedure-B using phosphonium salt (**3q**) (0.2 mmol, 125 mg) product (**5f**) was isolated in (35 mg, 76% yield) as a colorless solid by column chromatography on silica gel (EtOAc/Hexane = 05:95).

R_f = 0.70 (10% EtOAc in hexane).

^{1}H NMR (400 MHz, $CDCl_3$) δ: 7.33 – 7.28 (m, 2H), 7.27 – 7.24 (m, 2H), 7.05 – 6.98 (m, 2H), 6.90 – 6.83 (m, 2H), 5.37 (d, *J* = 1.2 Hz, 1H), 5.31 (d, *J* = 1.2 Hz, 1H), 3.83 (s, 3H).

The spectral data are consistent with those reported in the literature.[6]

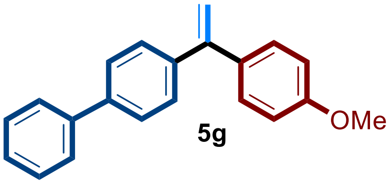

5g

4-(1-(4-methoxyphenyl)vinyl)-1,1'-biphenyl (5g):

Prepared according to general Procedure-B using phosphonium salt (**3af**) (0.2 mmol, 137 mg) product (**5g**) was isolated in (41 mg, 72% yield) as a white solid by column chromatography on silica gel (EtOAc/Hexane = 05:95).

R_f = 0.62 (10% EtOAc in hexane).

^{1}H NMR (400 MHz, $CDCl_3$) δ 7.65 – 7.60 (m, 2H), 7.60 – 7.55 (m, 2H), 7.49 – 7.40 (m, 4H), 7.39 – 7.30 (m, 3H), 6.93 – 6.85 (m, 2H), 5.48 – 5.36 (m, 2H), 3.84 (s, 3H).

The spectral data are consistent with those reported in the literature.[9]

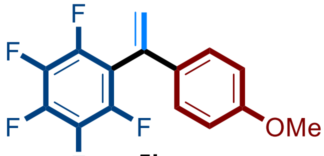

5h

1,2,3,4,5-pentafluoro-6-(1-(4-methoxyphenyl)vinyl)benzene (5h)

Prepared according to general Procedure-B using phosphonium salt (**3ag**) (0.2 mmol, 140 mg) product (**5h**) was isolated in (45 mg, 75% yield) as a white solid by column chromatography on silica gel (EtOAc/Hexane = 05:95).

R_f = 0.72 (15% EtOAc in hexane).

^{1}H NMR (400 MHz, $CDCl_3$) δ 7.18 – 7.10 (m, 2H), 6.85 – 6.68 (m, 2H), 5.88 (s, 1H), 5.25 (s, 1H), 3.73 (s, 3H).

^{13}C{^{1}H} NMR (101 MHz, $CDCl_3$) δ 160.0, 137.2 (dd, *J* = 10.4, 6.8 Hz), 133.9, 133.7, 130.8, 128.8, 128.6 (d, *J* = 7.0 Hz), 127.2, 118.8, 114.1, 55.4.

^{19}F NMR (376 MHz, $CDCl_3$) δ -140.3 – -140.4 (m), -155.3 (t, *J* = 20.8 Hz), -161.9 – -162.1 (m).

HRMS (ESI) was calculated for $[C_{15}H_9F_5O+H]^+$ $[M+H]^+$: m/z 301.0652, found = 301.0646.

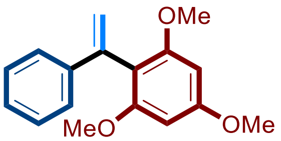

5i

1,3,5-trimethoxy-2-(1-phenylvinyl)benzene (5i):

Prepared according to general Procedure-B using phosphonium salt (**3t**) (0.2 mmol, 133 mg) product (**5i**) was isolated in (27 mg, 49% yield) as a colorless solid by column chromatography on silica gel (EtOAc/Hexane = 05:95).

R_f = 0.71 (10% EtOAc in hexane).

^{1}H NMR (400 MHz, $CDCl_3$) δ 7.35 – 7.33 (m, 2H), 7.28 – 7.21 (m, 3H), 6.22 (s, 2H), 5.96 (d, *J* = 1.5 Hz, 1H), 5.22 (d, *J* = 1.5 Hz, 1H), 3.87 (s, 3H), 3.70 (s, 6H).

The spectral data are consistent with those reported in the literature.[10]

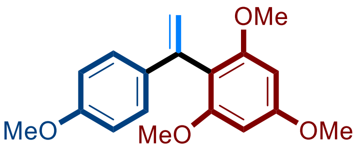

5j

1,3,5-trimethoxy-2-(1-(4-methoxyphenyl)vinyl)benzene (5j):

Prepared according to general Procedure-B using phosphonium salt (**3p**) (0.2 mmol, 139 mg) product (**5j**) was isolated in (39 mg, 65% yield) as a white solid by column chromatography on silica gel (EtOAc/Hexane = 05:95).

R_f = 0.30 (10% EtOAc in hexane).

^{1}H NMR (400 MHz, $CDCl_3$) δ: 7.27 (s, 1H), 7.25 (s, 1H), 6.78 (d, J = 8.9 Hz, 2H), 6.20 (s, 2H), 5.85 (d, J = 1.5 Hz, 1H), 5.09 (d, J = 1.5 Hz, 1H), 3.86 (s, 3H), 3.78 (s, 3H), 3.70 (s, 6H).

The spectral data are consistent with those reported in the literature. [11]

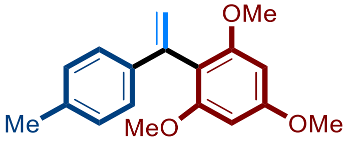

5k

1,3,5-trimethoxy-2-(1-(p-tolyl)vinyl)benzene (5k):

Prepared according to general Procedure-B using phosphonium salt (**3z**) (0.2 mmol, 136 mg) product (**5k**) was isolated in (49 mg, 87% yield) as a white solid by column chromatography on silica gel (EtOAc/Hexane = 05:95).

R_f = 0.65 (10% EtOAc in hexane).

^{1}H NMR (400 MHz, $CDCl_3$) δ 7.25 – 7.22 (m, 2H), 7.08 – 7.05 (m, 2H), 6.22 (s, 2H), 5.93 (d, J = 1.5 Hz, 1H), 5.15 (d, J = 1.5 Hz, 1H), 3.86 (s, 3H), 3.70 (s, 6H), 2.32 (s, 3H).

The spectral data are consistent with those reported in the literature.[12]

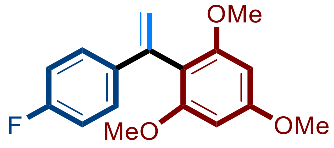

5l

2-(1-(4-fluorophenyl)vinyl)-1,3,5-trimethoxybenzene (5l):

Prepared according to general Procedure-B using phosphonium salt (**3u**) (0.2 mmol, 137 mg) product (**5l**) was isolated in (41 mg, 72% yield) as a colorless oil by column chromatography on silica gel (EtOAc/Hexane = 05:95).

R_f = 0.40 (10% EtOAc in hexane).

^{1}H NMR (500 MHz, $CDCl_3$) δ: 7.30 – 7.26 (m, 2H), 6.94 – 6.91 (m, 2H), 6.20 (s, 2H), 5.86 (d, *J* = 1.4 Hz, 1H), 5.18 (d, *J* = 1.4 Hz, 1H), 3.86 (s, 3H), 3.69 (s, 6H).

The spectral data are consistent with those reported in the literature. [11]

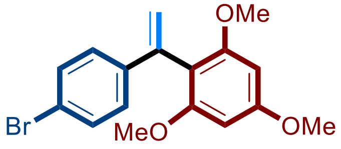

5m

2-(1-(4-bromophenyl)vinyl)-1,3,5-trimethoxybenzene (5m):

Prepared according to general Procedure-B using phosphonium salt (**3s**) (0.2 mmol, 150 mg) product (**5m**) was isolated in (39 mg, 56% yield) as a colorless oil by column chromatography on silica gel (EtOAc/Hexane = 05:95).

R_f = 0.30 (10% EtOAc in hexane).

^{1}H NMR (400 MHz, $CDCl_3$) δ: 7.36 (d, *J* = 8.6 Hz, 2H), 7.18 (d, *J* = 8.6 Hz, 2H), 6.19 (s, 2H), 5.92 (d, *J* = 1.3 Hz, 1H), 5.22 (d, *J* = 1.3 Hz, 1H), 3.85 (s, 3H), 3.69 (s, 6H).

^{13}C{^{1}H} NMR (101 MHz, $CDCl_3$) δ: 160.9, 158.8, 140.2, 140.1, 131.2, 127.6, 121.1, 117.0, 111.8, 91.0, 56.1, 55.5.

HRMS (ESI) was calculated for $[C_{17}H_{17}BrO_3+H]^+$ $[M+H]^+$: m/z 349.0433, found = 349.0432.

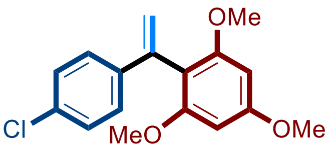

5n

2-(1-(4-chlorophenyl)vinyl)-1,3,5-trimethoxybenzene (5n):

Prepared according to general Procedure-B using phosphonium salt (**3v**) (0.2 mmol, 140 mg) product (**5n**) was isolated in (30 mg, 49% yield) as a white solid by column chromatography on silica gel (EtOAc/Hexane = 05:95).

R_f = 0.46 (10% EtOAc in hexane).

^{1}H NMR (400 MHz, $CDCl_3$) δ: 7.23 (q, J = 9.1 Hz, 4H), 6.20 (s, 2H), 5.92 (d, J = 1.3 Hz, 1H), 5.22 (d, J = 1.3 Hz, 1H), 3.86 (s, 3H), 3.69 (s, 6H).

^{13}C{^{1}H} NMR (101 MHz, $CDCl_3$) δ: 160.9, 158.8, 140.1, 139.5, 132.8, 128.2, 127.3, 116.9, 111.8, 91.0, 56.1, 55.5.

HRMS (ESI) was calculated for $[C_{17}H_{17}ClO_3+H]^+$ $[M+H]^+$: m/z 305.0939, found = 305.0933.

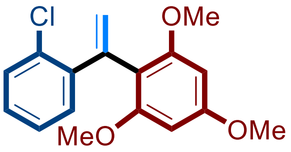

5o

2-(1-(2-chlorophenyl)vinyl)-1,3,5-trimethoxybenzene (5o):

Prepared according to general Procedure-B using phosphonium salt (**3a**) (0.2 mmol, 140 mg) product (**5o**) was isolated in (31 mg, 52% yield) as a white solid by column chromatography on silica gel (EtOAc/Hexane = 05:95).

R_f = 0.40 (10% EtOAc in hexane).

^{1}H NMR (400 MHz, $CDCl_3$) δ: 7.31 – 7.29 (m, 1H), 7.26 – 7.24 (m, 1H), 7.15 – 7.10 (m, 2H), 6.14 (s, 2H), 5.70 (d, J = 1.9 Hz, 1H), 5.54 (d, J = 1.9 Hz, 1H), 3.82 (s, 3H), 3.67 (s, 6H).

^{13}C{^{1}H} NMR (101 MHz, $CDCl_3$) δ: 160.7, 158.8, 142.1, 139.1, 132.0, 130.7, 129.7, 127.5, 126.1, 122.4, 113.1, 91.1, 56.0, 55.4.

HRMS (ESI) was calculated for $[C_{17}H_{17}ClO_3+H]^+$ $[M+H]^+$: m/z 305.0939, found = 305.0932.

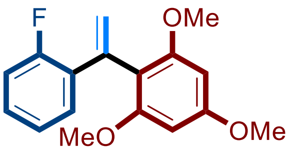

5p

2-(1-(2-fluorophenyl)vinyl)-1,3,5-trimethoxybenzene (5p)

Prepared according to general Procedure-B using phosphonium salt (**3ad**) (0.2 mmol, 137.3 mg) product (**5p**) was isolated in (32.3 mg, 56% yield) as a white solid by column chromatography on silica gel (EtOAc/Hexane = 05:95).

R_f = 0.65 (10% EtOAc in hexane).

^{1}H NMR (400 MHz, $CDCl_3$) δ 7.16 – 7.10 (m, 1H), 7.07 (dt, *J* = 8.0, 2.0 Hz, 1H), 7.03 – 6.95 (m, 2H), 6.18 (s, 2H), 5.96 (d, *J* = 1.8 Hz, 1H), 5.45 (t, *J* = 1.7 Hz, 1H), 3.84 (s, 3H), 3.69 (s, 6H).

The spectral data are consistent with those reported in the literature. [11]

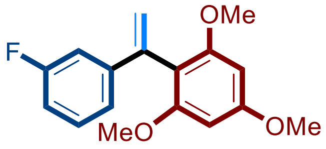

5q

2-(1-(3-fluorophenyl)vinyl)-1,3,5-trimethoxybenzene (5q)

Prepared according to general Procedure-B using phosphonium salt (**3ae**) (0.2 mmol, 123 mg) product (**5q**) was isolated in (19 mg, 33% yield) as a colorless oil by column chromatography on silica gel (EtOAc/Hexane = 05:95).

R_f = 0.32 (10% EtOAc in hexane).

^{1}H NMR (400 MHz, $CDCl_3$) δ 7.20 (q, *J* = 7.4 Hz, 1H), 7.12 (d, *J* = 7.8 Hz, 1H), 6.99 (d, *J* = 10.8 Hz, 1H), 6.90 (t, *J* = 8.7 Hz, 1H), 6.20 (s, 2H), 5.94 (s, 1H), 5.25 (s, 1H), 3.86 (s, 3H), 3.70 (s, 6H).

^{13}C{^{1}H} NMR (101 MHz, $CDCl_3$) δ 164.2, 161.8, 160.9, 158.8, 143.5 (d, *J* = 7.5 Hz), 140.2 (d, *J* = 2.4 Hz), 129.4 (d, *J* = 8.4 Hz), 121.6 (d, *J* = 2.6 Hz), 117.5, 113.9 (d, *J* = 21.4 Hz), 112.9 (d, *J* = 22.1 Hz), 111.8, 91.0, 56.1, 55.5.

^{19}F NMR (376 MHz $CDCl_3$) δ -114.5 (s).

HRMS (ESI) was calculated for $[C_{17}H_{17}FO_3+H]^+$ $[M+H]^+$: m/z 289.1234, found = 289.1221.

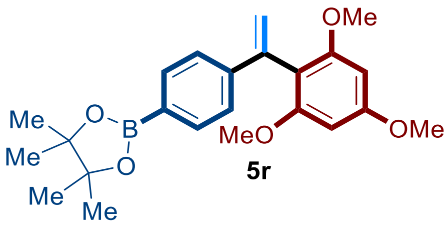

5r

4,4,5,5-tetramethyl-2-(4-(1-(2,4,6-trimethoxyphenyl)vinyl)phenyl)-1,3,2-dioxaborolane (5r):

Prepared according to general Procedure-B using phosphonium salt (**3aa**) (0.2 mmol, 158 mg) product (**5r**) was isolated in (33 mg, 42% yield) as a white solid by column chromatography on silica gel (EtOAc/Hexane = 10:90).

R_f = 0.51 (20% EtOAc in hexane).

^{1}H NMR (400 MHz, $CDCl_3$) δ: 7.70 – 7.67 (m, 2H), 7.32 – 7.30 (m, 2H), 6.20 (s, 2H), 5.99 (d, *J* = 1.5 Hz, 1H), 5.24 (d, *J* = 1.5 Hz, 1H), 3.86 (s, 3H), 3.67 (s, 6H), 1.33 (s, 12H).

^{13}C{^{1}H} NMR (101 MHz, $CDCl_3$) δ 160.8, 158.9, 141.2, 134.7, 125.2, 117.3, 112.3, 91.1, 83.7, 56.1, 55.5, 25.0.

^{11}B NMR (128 MHz, $CDCl_3$) δ: 30.9.

HRMS (ESI) was calculated for $[C_{23}H_{29}BO_5 + H]^+$ $[M + H]^+$: m/z 397.2180, found = 397.2175.

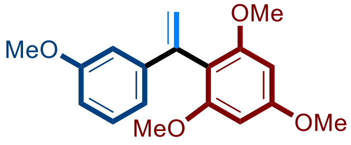

5s

1,3,5-trimethoxy-2-(1-(3-methoxyphenyl)vinyl)benzene (5s):

Prepared according to general Procedure-B using phosphonium salt (**3c**) (0.2 mmol, 140 mg) product (**5s**) was isolated in (30 mg, 55% yield) as a colorless oil by column chromatography on silica gel (EtOAc/Hexane = 05:95).

R_f = 0.29 (10% EtOAc in hexane).

^{1}H NMR (400 MHz, $CDCl_3$) δ: 7.17 (td, *J* = 7.6, 0.5 Hz, 1H), 6.94 – 6.89 (m, 2H), 6.77 (ddd, *J* = 8.2, 2.6, 1.0 Hz, 1H), 6.20 (s, 2H), 5.95 (d, *J* = 1.5 Hz, 1H), 5.21 (d, *J* = 1.5 Hz, 1H), 3.85 (s, 3H), 3.77 (s, 3H), 3.70 (s, 6H).

The spectral data are consistent with those reported in the literature. [11]

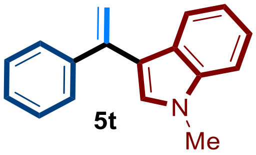

5t

1-methyl-3-(1-phenylvinyl)-1H-indole (5t):

Prepared according to general Procedure-B using phosphonium salt (**3w**) (0.2 mmol, 127 mg) product (**5t**) was isolated in (22 mg, 47% yield) as a white solid by column chromatography on silica gel (EtOAc/Hexane = 05:95).

R_f = 0.39 (10% EtOAc in hexane).

^{1}**H NMR** (400 MHz, $CDCl_3$) δ: 7.56 (dt, J = 8.1, 0.9 Hz, 1H), 7.50 – 7.46 (m, 2H), 7.36 – 7.33 (m, 4H), 7.25 (d, J = 1.2 Hz, 1H), 7.12 – 7.08 (m, 1H), 6.98 (s, 1H), 5.57 (d, J = 1.7 Hz, 1H), 5.37 (d, J = 1.6 Hz, 1H), 3.78 (s, 3H).

HRMS (ESI) was calculated for $[C_{17}H_{15}N + H]^+$ $[M + H]^+$: m/z 234.1277, found = 234.1277.

The spectral data are consistent with those reported in the literature.[13]

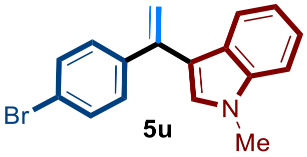

5u

3-(1-(4-bromophenyl)vinyl)-1-methyl-1H-indole (5u):

Prepared according to general Procedure-B using phosphonium salt (**3x**) (0.2 mmol, 140 mg) product (**5u**) was isolated in (30 mg, 48% yield) as a yellow solid by column chromatography on silica gel (EtOAc/Hexane = 05:95).

R_f = 0.54 (10% EtOAc in hexane).

^{1}**H NMR** (400 MHz, $CDCl_3$) δ: 7.41 (dd, J = 19.2, 8.2 Hz, 3H), 7.26 (d, J = 8.5 Hz, 3H), 7.16 (t, J = 7.9 Hz, 1H), 7.03 (t, J = 9.2 Hz, 1H), 6.89 (s, 1H), 5.48 (d, J = 1.6 Hz, 1H), 5.28 (d, J = 1.6 Hz, 1H), 3.70 (s, 3H).

The spectral data are consistent with those reported in the literature. [14]

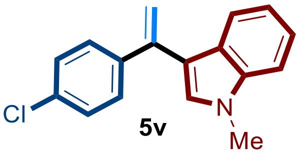

5v

3-(1-(4-chlorophenyl)vinyl)-1-methyl-1H-indole (5v):

Prepared according to general Procedure-B using phosphonium salt (**3y**) (0.2 mmol, 130 mg) product (**5v**) was isolated in (23 mg, 43% yield) as a white solid by column chromatography on silica gel (EtOAc/Hexane = 05:95).

R_f = 0.54 (10% EtOAc in hexane).

^{1}H NMR (400 MHz, $CDCl_3$) δ: 7.45 (dt, J = 8.0, 1.0 Hz, 1H), 7.36 – 7.30 (m, 2H), 7.28 (dt, J = 8.2, 1.0 Hz, 1H), 7.26 – 7.24 (m, 1H), 7.22 – 7.18 (m, 2H), 7.07 – 7.03 (m, 1H), 6.91 (s, 1H), 5.50 (d, J = 1.5 Hz, 1H), 5.30 (d, J = 1.5 Hz, 1H), 3.72 (s, 3H).

^{13}C{^{1}H} NMR (101 MHz, $CDCl_3$) δ: 142.3, 141.2, 137.5, 133.4, 129.6, 129.1, 128.3, 126.7, 122.1, 120.8, 119.9, 116.2, 112.0, 109.6, 32.9.

HRMS (ESI) was calculated for $[C_{17}H_{14}ClN+H]^+$ $[M+H]^+$: m/z 268.0887, found = 268.0888.

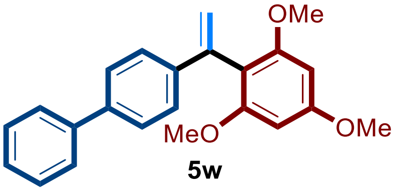

5w

4-(1-(2,4,6-trimethoxyphenyl)vinyl)-1,1'-biphenyl (5w):

Prepared according to general Procedure-B using phosphonium salt (**3f**) (0.2 mmol, 148 mg) product (**5w**) was isolated in (24 mg, 35% yield) as a white solid by column chromatography on silica gel (EtOAc/Hexane = 05:95).

R_f = 0.27 (10% EtOAc in hexane).

^{1}H NMR (400 MHz, $CDCl_3$) δ: 7.60 – 7.57 (m, 2H), 7.50 – 7.48 (m, 2H), 7.43 – 7.39 (m, 4H), 7.33 – 7.29 (m, 1H), 6.23 (s, 2H), 6.01 (d, J = 1.4 Hz, 1H), 5.22 (d, J = 1.4 Hz, 1H), 3.87 (s, 3H), 3.72 (s, 6H).

^{13}C{^{1}H} NMR (101 MHz, $CDCl_3$) δ: 160.8, 158.8, 141.1, 140.7, 139.9, 139.8, 128.8, 127.2, 127.0, 126.9, 126.3, 116.4, 112.3, 91.0, 56.2, 55.5.

HRMS (ESI) was calculated for $[C_{23}H_{22}O_3+H]^+$ $[M+H]^+$: m/z 347.1641, found = 347.1635.

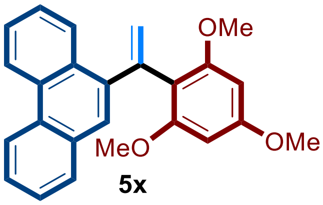

5x

9-(1-(2,4,6-trimethoxyphenyl)vinyl)phenanthrene (5x):

Prepared according to general Procedure-B using phosphonium salt (**3b**) (0.2 mmol, 153 mg) product (**5x**) was isolated in (53 mg, 71% yield) as a white solid by column chromatography on silica gel (EtOAc/Hexane = 05:95).

R_f = 0.37 (10% EtOAc in hexane).

^{1}H NMR (400 MHz, $CDCl_3$) δ: 8.71 – 8.64 (m, 2H), 8.50 (dd, J = 8.2, 1.4 Hz, 1H), 7.79 (dd, J = 7.8, 1.5 Hz, 1H), 7.63 – 7.51 (m, 5H), 6.16 (s, 2H), 5.72 (d, J = 2.2 Hz, 1H), 5.68 (d, J = 2.2 Hz, 1H), 3.82 (s, 3H), 3.65 (s, 6H).

^{13}C{^{1}H} NMR (101 MHz, $CDCl_3$) δ: 160.6, 158.8, 140.5, 140.3, 131.8, 131.2, 130.6, 129.8, 128.7, 127.4, 126.4, 126.0, 126.0, 126.0, 125.9, 122.6, 122.4, 122.2, 114.5, 91.2, 55.9, 55.4.

HRMS (ESI) was calculated for $[C_{25}H_{22}O_3 + H]^+$ $[M + H]^+$: m/z 371.1641, found = 371.1634.

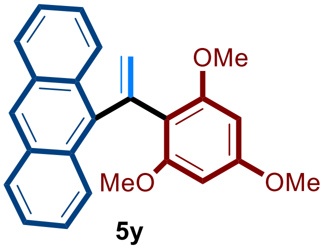

5y

9-(1-(2,4,6-trimethoxyphenyl)vinyl)anthracene (5y):

Prepared according to general Procedure-B using phosphonium salt (**3d**) (0.2 mmol, 153 mg) product (**5y**) was isolated in (42 mg, 57% yield) as a yellow oil by column chromatography on silica gel (EtOAc/Hexane = 05:95).

R_f = 0.32 (10% EtOAc in hexane).

^{1}H NMR (400 MHz, CD_3CN) δ: 7.91 – 7.89 (m, 1H), 7.71 (ddd, J = 12.3, 7.8, 1.4 Hz, 2H), 7.37 – 7.27 (m, 2H), 7.24 – 7.17 (m, 2H), 6.98 (ddd, J = 7.9, 7.3, 1.3 Hz, 1H), 6.93 (s, 1H), 6.08 (s, 2H), 5.73 (d, J = 13.9 Hz, 2H), 3.83 (s, 3H), 3.51 (s, 6H).

^{13}C{^{1}H} NMR (101 MHz, $CDCl_3$) δ: 160.9, 158.5, 142.5, 136.8, 135.4, 135.2, 134.8, 133.9, 127.8, 126.9, 126.8, 126.4, 126.0, 123.9, 123.7, 123.6, 119.4, 109.3, 108.7, 90.9, 55.4, 55.4.

HRMS (ESI) was calculated for $[C_{25}H_{22}O_3 + H]^+$ $[M + H]^+$: m/z 371.1641, found = 371.1636.

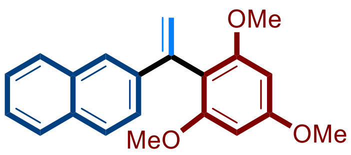

5z

2-(1-(2,4,6-trimethoxyphenyl)vinyl)naphthalene (5z)

Prepared according to general Procedure-B using phosphonium salt (**3ab**) (0.2 mmol, 144 mg) product (**5z**) was isolated in (23 mg, 36% yield) as a white solid by column chromatography on silica gel (EtOAc/Hexane = 05:95).

R_f = 0.45 (10% EtOAc in hexane).

^{1}H NMR (400 MHz, $CDCl_3$) δ 7.79 – 7.71 (m, 3H), 7.64 – 7.61 (m, 2H), 7.42 – 7.38 (m, 2H), 6.25 (s, 2H), 6.09 (d, J = 1.4 Hz, 1H), 5.31 (d, J = 1.4 Hz, 1H), 3.89 (s, 3H), 3.69 (s, 6H).

The spectral data are consistent with those reported in the literature. [11]

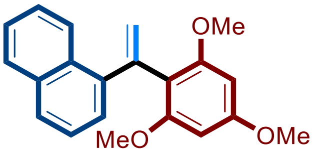

5aa

1-(1-(2,4,6-trimethoxyphenyl)vinyl)naphthalene (5aa)

Prepared according to general Procedure-B using phosphonium salt (**3e**) (0.2 mmol, 143 mg) product (**5aa**) was isolated in (26 mg, 41% yield) as a white solid by column chromatography on silica gel (EtOAc/Hexane = 05:95).

R_f = 0.50 (10% EtOAc in hexane).

^{1}H NMR (400 MHz, $CDCl_3$) δ: 8.45 – 8.42 (m, 1H), 7.82 – 7.79 (m, 1H), 7.70 – 7.68 (m, 1H), 7.44 – 7.39 (m, 2H), 7.35 (d, J = 1.6 Hz, 1H), 7.34 (s, 1H), 6.15 (s, 2H), 5.64 (dd, J = 11.0, 2.2 Hz, 2H), 3.81 (s, 3H), 3.64 (s, 6H).

^{13}C{^{1}H} NMR (101 MHz, $CDCl_3$) δ: 160.5, 158.7, 141.9, 140.2, 133.9, 131.6, 128.1, 126.8, 126.7, 125.6, 125.2, 125.1, 122.0, 114.9, 91.2, 55.9, 55.4.

HRMS (ESI) was calculated for $[C_{21}H_{20}O_3 + H]^+$ $[M + H]^+$: m/z 321.1485, found = 321.1474.

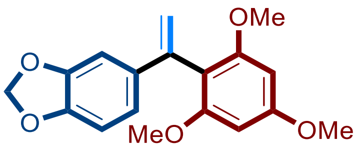

5ab

5-(1-(2,4,6-trimethoxyphenyl)vinyl)benzo[d][1,3]dioxole (5ab):

Prepared according to general Procedure-B using phosphonium salt (**3ac**) (0.2 mmol, 142 mg) product (**5ab**) was isolated in (51 mg, 80% yield) as a white solid by column chromatography on silica gel (EtOAc/Hexane = 10:90).

R_f = 0.51 (20% EtOAc in hexane).

^{1}H NMR (400 MHz, $CDCl_3$) δ: 6.88 (d, J = 2.0 Hz, 1H), 6.77 (dd, J = 8.2, 1.8 Hz, 1H), 6.68 (d, J = 8.1 Hz, 1H), 6.20 (s, 2H), 5.92 (s, 2H), 5.82 (d, J = 1.4 Hz, 1H), 5.09 (d, J = 1.4 Hz, 1H), 3.85 (s, 3H), 3.71 (s, 6H).

^{13}C{^{1}H} NMR (101 MHz, $CDCl_3$) δ: 160.7, 158.7, 147.6, 146.9, 140.7, 135.4, 119.8, 115.0, 112.6, 107.9, 106.5, 101.0, 91.0, 56.2, 55.5.

HRMS (ESI) was calculated for $[C_{18}H_{18}O_5 + H]^+$ $[M + H]^+$: m/z 315.1227, found = 315.1222.

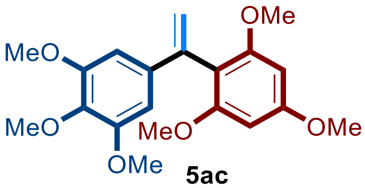

5ac

1,2,3-trimethoxy-5-(1-(2,4,6-trimethoxyphenyl)vinyl)benzene (5ac):

Prepared according to general Procedure-B using phosphonium salt (**3g**) (0.2 mmol, 151 mg) product (**5ac**) was isolated in (40 mg, 55% yield) as a white solid by column chromatography on silica gel (EtOAc/Hexane = 15:85).

R_f = 0.52 (30% EtOAc in hexane).

^{1}H NMR (400 MHz, $CDCl_3$) δ: 6.57 (d, J = 1.3 Hz, 2H), 6.21 (d, J = 1.4 Hz, 2H), 5.89 (t, J = 1.3 Hz, 1H), 5.17 (t, J = 1.1 Hz, 1H), 3.86 (d, J = 1.4 Hz, 3H), 3.82 (d, J = 1.4 Hz, 3H), 3.78 (d, J = 1.4 Hz, 6H), 3.72 (t, J = 1.0 Hz, 6H).

^{13}C{^{1}H} NMR (101 MHz, $CDCl_3$) δ: 160.7, 158.7, 152.9, 141.0, 137.7, 136.6, 115.9, 112.2, 103.4, 91.0, 60.9, 56.1, 56.1, 55.4.

HRMS (ESI) was calculated for $[C_{20}H_{24}O_6 + H]^+$ $[M + H]^+$: m/z 361.1645, found = 361.1629.

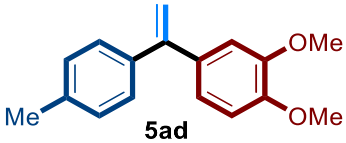

5ad

1,2-dimethoxy-4-(1-(p-tolyl)vinyl)benzene (5ad):

Prepared according to general Procedure-B using phosphonium salt (**3i**) (0.2 mmol, 130 mg) product (**5ad**) was isolated in (30 mg, 60% yield) as a white solid by column chromatography on silica gel (EtOAc/Hexane = 05:95).

R_f = 0.41 (10% EtOAc in hexane).

^{1}H NMR (400 MHz, $CDCl_3$) δ: 7.27 – 7.25 (m, 2H), 7.15 (d, J = 7.9 Hz, 2H), 6.91 – 6.82 (m, 3H), 5.36 (t, J = 0.9 Hz, 2H), 3.90 (s, 3H), 3.84 (s, 3H), 2.38 (s, 3H).

The spectral data are consistent with those reported in the literature. [15]

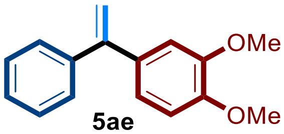

5ae

1,2-dimethoxy-4-(1-phenylvinyl)benzene (5ae):

Prepared according to general Procedure-B using phosphonium salt (**3j**) (0.2 mmol, 127 mg) product (**5ae**) was isolated in (26 mg, 54% yield) as a white solid by column chromatography on silica gel (EtOAc/Hexane = 05:95).

R_f = 0.43 (10% EtOAc in hexane).

^{1}H NMR (400 MHz, $CDCl_3$) δ: 7.37 – 7.32 (m, 5H), 6.91 – 6.82 (m, 3H), 5.40 (dd, J = 10.3, 1.3 Hz, 2H), 3.90 (s, 3H), 3.84 (s, 3H).

The spectral data are consistent with those reported in the literature. [15]

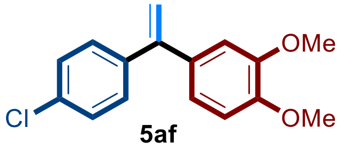

5af

4-(1-(4-chlorophenyl)vinyl)-1,2-dimethoxybenzene (5af):

Prepared according to general Procedure-B using phosphonium salt (**3k**) (0.2 mmol, 134 mg) product (**5af**) was isolated in (40 mg, 73% yield) as a colorless solid by column chromatography on silica gel (EtOAc/Hexane = 05:95).

R_f = 0.52 (10% EtOAc in hexane).

^{1}H NMR (400 MHz, $CDCl_3$) δ: 7.32 – 7.26 (m, 4H), 6.86 – 6.82 (m, 3H), 5.39 (dd, *J* = 18.6, 1.1 Hz, 2H), 3.90 (s, 3H), 3.84 (s, 3H).

^{13}C{^{1}H} NMR (101 MHz, $CDCl_3$) δ 149.1, 148.8, 148.7, 140.2, 133.9, 133.7, 129.7, 128.4, 121.0, 113.6, 111.4, 110.9, 56.0, 56.0.

HRMS (ESI) was calculated for $[C_{16}H_{15}ClO_2]^+$ $[M]^+$: m/z 274.0761, found = 274.0760.

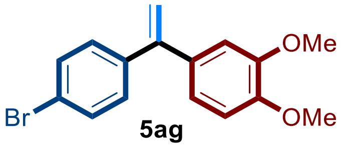

5ag

4-(1-(4-bromophenyl)vinyl)-1,2-dimethoxybenzene (5ag):

Prepared according to general Procedure-B using phosphonium salt (**3l**) (0.2 mmol, 143 mg) product (**5ag**) was isolated in (50 mg, 78% yield) as a white solid by column chromatography on silica gel (EtOAc/Hexane = 05:95).

R_f = 0.37 (10% EtOAc in hexane).

^{1}H NMR (400 MHz, $CDCl_3$) δ: 7.48 – 7.44 (m, 2H), 7.24 – 7.21 (m, 2H), 6.88 – 6.82 (m, 3H), 5.39 (dd, *J* = 18.5, 1.1 Hz, 2H), 3.90 (s, 3H), 3.84 (s, 3H).

The spectral data are consistent with those reported in the literature. [15]

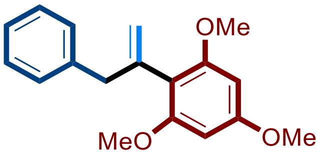

5ah

1,3,5-trimethoxy-2-(3-phenylprop-1-en-2-yl)benzene (5ah):

Prepared according to general Procedure-B using phosphonium salt (**3ah**) (0.2 mmol, 136 mg) product (**5ah**) was isolated in (49 mg, 85% yield) as a colorless oil by column chromatography on silica gel (EtOAc/Hexane = 05:95).

R_f = 0.37 (10% EtOAc in hexane).

^{1}H NMR (400 MHz, $CDCl_3$) δ: 7.24 – 7.20 (m, 2H), 7.18 – 7.12 (m, 3H), 6.10 (s, 2H), 5.18 – 5.16 (m, 1H), 4.96 – 4.94 (m, 1H), 3.80 (s, 3H), 3.68 (s, 6H), 3.59 (s, 2H).

^{13}C{^{1}H} NMR (101 MHz, $CDCl_3$) δ 160.2, 158.2, 142.9, 140.1, 129.6, 127.9, 125.8, 116.6, 113.4, 90.9, 56.0, 55.4, 43.7.

HRMS (ESI) was calculated for $[C_{18}H_{20}O_3+H]^+$ $[M+H]^+$: m/z 285.1485, found = 285.1490.

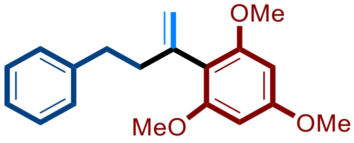

5ai

1,3,5-trimethoxy-2-(4-phenylbut-1-en-2-yl)benzene (5ai):

Prepared according to general Procedure-B using phosphonium salt (**3ai**) (0.2 mmol, 140 mg) product (**5ai**) was isolated in (37 mg, 62% yield) as a colorless oil by column chromatography on silica gel (EtOAc/Hexane = 05:95).

R_f = 0.45 (10% EtOAc in hexane).

^{1}H NMR (400 MHz, $CDCl_3$) δ: 7.25 – 7.24 (m, 2H), 7.19 (d, J = 7.5 Hz, 2H), 7.15 (t, J = 7.3 Hz, 1H), 6.17 (s, 2H), 5.35 (d, J = 1.9 Hz, 1H), 4.93 (d, J = 1.2 Hz, 1H), 3.84 (s, 3H), 3.78 (s, 6H), 2.73 – 2.71 (m, 2H), 2.65 – 2.63 (m, 2H).

^{13}C{^{1}H} NMR (101 MHz, $CDCl_3$) δ 160.2, 159.8, 158.3, 143.0, 141.5, 129.9, 128.6, 128.3, 125.7, 116.3, 115.3, 113.4, 91.1, 90.8, 56.1, 56.0, 55.4, 39.0, 34.4.

HRMS (ESI) was calculated for $[C_{19}H_{22}O_3+H]^+$ $[M+H]^+$: m/z 299.1641, found = 299.1655.

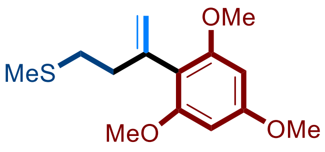

5aj

Methyl(3-(2,4,6-trimethoxyphenyl)but-3-en-1-yl)sulfane (5aj):

Prepared according to general Procedure-B using phosphonium salt (**3aj**) (0.2 mmol, 133 mg) product (**5aj**) was isolated in (40 mg, 75% yield) as a colorless oil by column chromatography on silica gel (EtOAc/Hexane = 05:95).

R_f = 0.37 (10% EtOAc in hexane).

^{1}H NMR (400 MHz, $CDCl_3$) δ: 6.14 (s, 2H), 5.35 – 5.30 (m, 1H), 4.93 (d, J = 2.0 Hz, 1H), 3.82 (s, 3H), 3.77 (s, 6H), 2.64 – 2.56 (m, 2H), 2.56 – 2.49 (m, 2H), 2.09 (s, 3H).

^{13}C{^{1}H} NMR (101 MHz, $CDCl_3$) δ 160.0, 158.1, 141.7, 131.7, 126.2, 116.2, 115.7, 90.9, 55.9, 55.4, 31.1, 17.5, 13.6.

HRMS (ESI) was calculated for $[C_{14}H_{20}O_3S+H]^+$ $[M+H]^+$: m/z 269.1205, found = 269.1219.

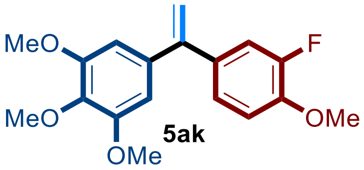

5ak

5-(1-(3-fluoro-4-methoxyphenyl)vinyl)-1,2,3-trimethoxybenzene (5ak):

Prepared according to general Procedure-B using phosphonium salt (**3ak**) (0.2 mmol, 143 mg) product (**5ak**) was isolated in (45 mg, 70% yield) as a colorless oil by column chromatography on silica gel (EtOAc/Hexane = 15:85).

R_f = 0.55 (30% EtOAc in hexane).

^{1}H NMR (400 MHz, $CDCl_3$) δ: 7.16 – 7.04 (m, 2H), 6.92 (t, J = 8.5 Hz, 1H), 6.53 (s, 2H), 5.38 (d, J = 1.1 Hz, 1H), 5.34 (d, J = 1.1 Hz, 1H), 3.91 (s, 3H), 3.88 (s, 3H), 3.82 (s, 6H).

^{13}C{^{1}H} NMR (101 MHz, $CDCl_3$) δ 153.3, 153.0, 150.9, 148.8 (d, J = 1.8 Hz), 147.5 (d, J = 10.9 Hz), 138.0, 137.0, 134.5 (d, J = 6.3 Hz), 124.1 (d, J = 3.3 Hz), 116.0 (d, J = 18.9 Hz), 113.5, 112.9 (d, J = 2.2 Hz), 105.7, 61.0, 56.4, 56.2, 56.4, 56.2.

^{19}F NMR (376 MHz $CDCl_3$) δ -135.6 (s).

The spectral data are consistent with those reported in the literature.[16]

2.3. General Procedure-C and Characterization for 1,1-Diarylethylene-*d*$_2$ Products (6)

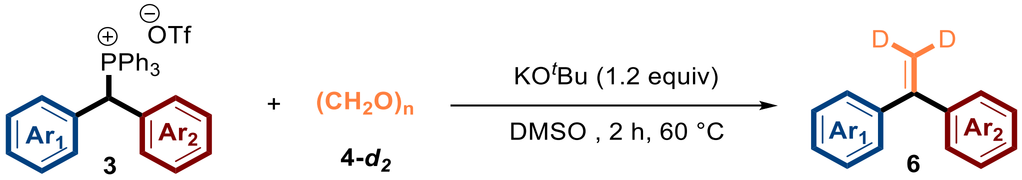

A 15 mL screw-cap vial containing a Teflon-coated magnetic stirring bar was charged with benzhydryl phosphonium salt **3** (0.2 mmol, 1.0 equiv), and taken inside the glovebox. Then, 2 mL of DMSO and KO^{t}Bu (0.24 mmol, 1.2 equiv) were added, and the mixture was stirred at room temperature for five minutes. After that, powder Paraformaldehyde-*d*$_2$ **4-*d*$_2$** (1.0 mmol, 5 equiv) was added, and the vial was sealed and taken out of the glove box to stir at 60 °C for 2 hours. After cooling to room temperature, the mixture was quenched with H_2O, and the aqueous phase was extracted with EtOAc (2 x 5 mL). The combined organic phases were washed with brine, dried over Na_2SO_4, filtered and concentrated under reduced pressure (by evaporator) to give the crude material, that was purified by column chromatography, to give the desired product (**6**).

Notes: (1) The percentage of deuterium (D%) incorporation for all the deuterated molecules were determined by ^{1}H-NMR after column chromatography purification. (2) Products **6c** and **6e** were analyzed with ^{2}H-NMR.

Table - S4: Scope of the reduction reaction of 1,1-Diarylethylene-d_2 products (**6**).

**Entry**	**Phosphonium Salts (3)**	**1,1-Diarylethylene-d_2 (6)**	**Yield[a] (D % incorporation)**
1.	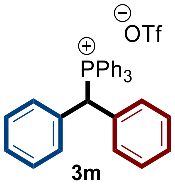 **3m**	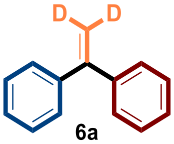 **6a**	70% (99% D)
2.	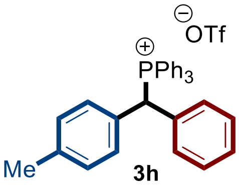 **3h**	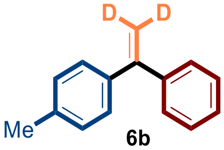 **6b**	84% (99% D)
3.	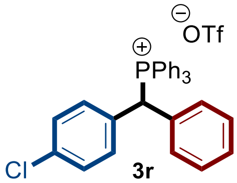 **3r**	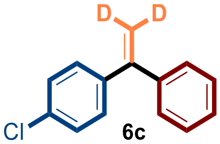 **6c**	81% (99% D)
4.	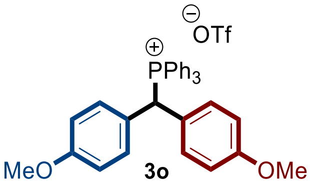 **3o**	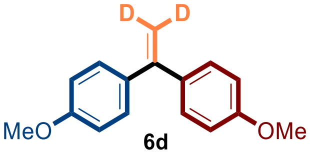 **6d**	73% (99% D)
5.	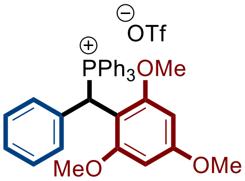 **3t**	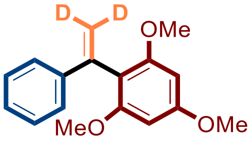 **6e**	52% (99% D)
6.	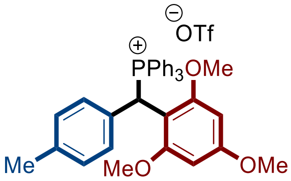 **3z**	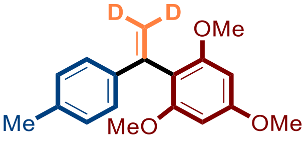 **6f**	74% (97% D)

7.	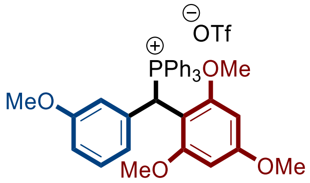 **3c**	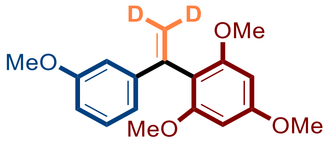 **6g**	40% (99% D)
8.	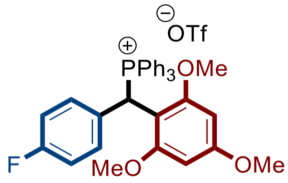 **3u**	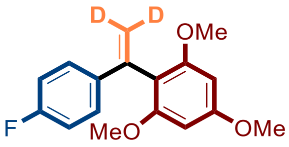 **6h**	66% (99% D)
9.	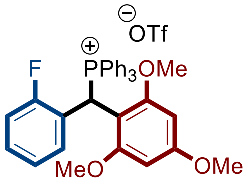 **3ad**	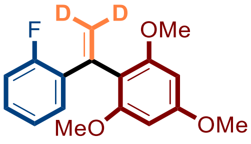 **6i**	74% (97% D)
10.	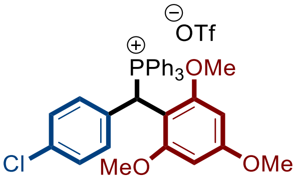 **3v**	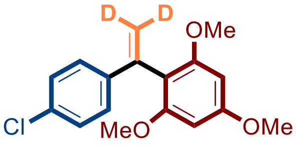 **6j**	49% (99% D)
11.	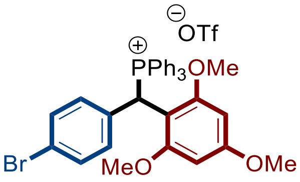 **3s**	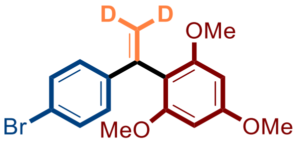 **6k**	42% (99% D)
12.	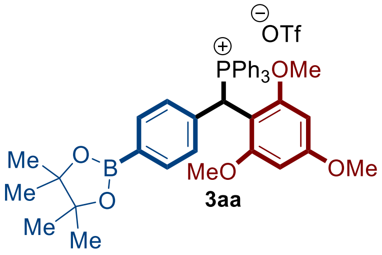 **3aa**	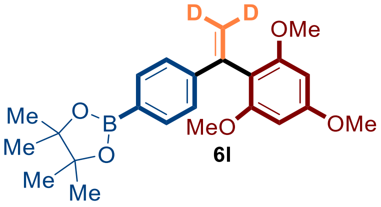 **6l**	38% (99% D)

13.	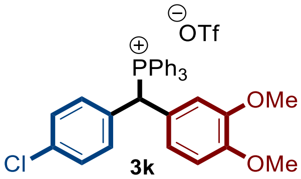 **3k**	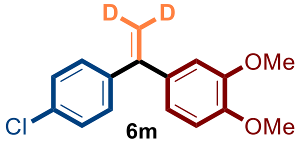 **6m**	67% (97% D)
14.	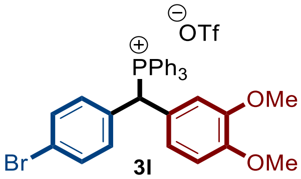 **3l**	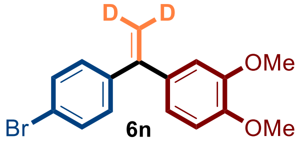 **6n**	56% (99% D)
15.	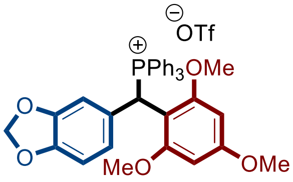 **3ac**	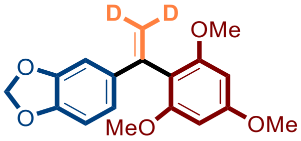 **6o**	78% (99% D)
16.	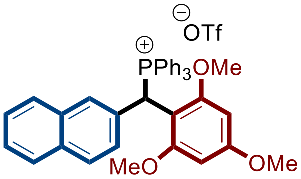 **3ab**	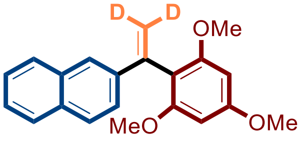 **6p**	64% (99% D)
17.	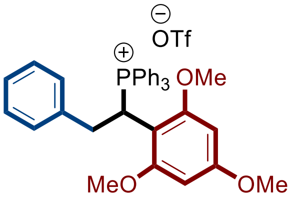 **3ah**	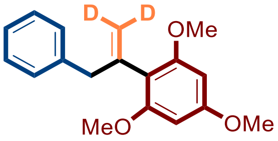 **6q**	79% (99% D)
18.	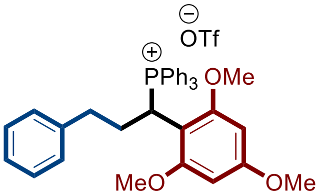 **3ai**	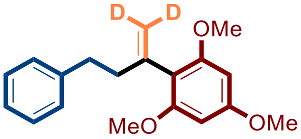 **6r**	58% (99% D)
19.	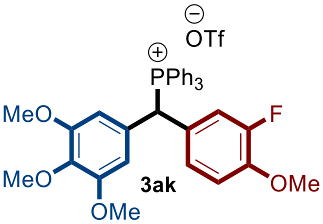 **3ak**	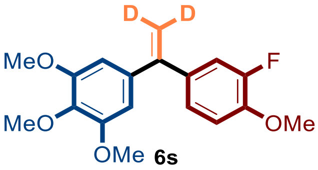 **6s**	63% (99% D)

All reactions were carried out using 0.2 mmol of benzhydryl phosphonium salt **3**. [a] Isolated yield. [b] Some examples were quenched with D_2O.

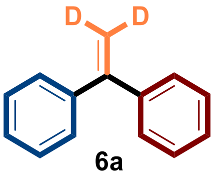

6a

(ethene-1,1-diyl-2,2-d2)dibenzene (6a):

Prepared according to general Procedure-C using phosphonium salt (**3m**) (0.2 mmol, 96 mg) product (**6a**) was isolated in (29 mg, 70% yield) as a white solid by column chromatography on silica gel (EtOAc/Hexane = 02:98). 99% Deuterium-incorporation.

R_f = 0.85 (10% EtOAc in hexane).

^{1}H NMR (400 MHz, $CDCl_3$) δ: 7.39 – 7.28 (m, 10H).

The spectral data are consistent with those reported in the literature. [17]

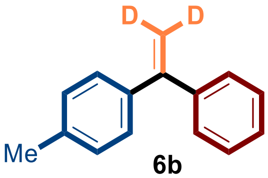

6b

1-methyl-4-(1-phenylvinyl-2,2-d2)benzene (6b):

Prepared according to general Procedure-C using phosphonium salt (**3h**) (0.2 mmol, 120 mg) product (**6b**) was isolated in (33 mg, 84% yield) as a colorless oil by column chromatography on silica gel (EtOAc/Hexane = 02:98). 99% Deuterium-incorporation.

R_f = 0.73 (5% EtOAc in hexane).

^{1}H NMR (400 MHz, $CDCl_3$) δ: 7.37 (brs, 5H), 7.25 (dd, J = 21.6, 7.7 Hz, 4H), 2.42 (s, 3H).

The spectral data are consistent with those reported in the literature. [18]

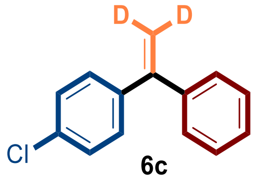

6c

1-chloro-4-(1-phenylvinyl-2,2-d2)benzene (6c):

Prepared according to general Procedure-C using phosphonium salt (**3r**) (0.2 mmol, 122 mg) product (**6c**) was isolated in (35 mg, 81% yield) as a colorless oil by column chromatography on silica gel (EtOAc/Hexane = 02:98). 99% Deuterium-incorporation.

R_f = 0.65 (5% EtOAc in hexane).

^{1}H NMR (400 MHz, $CDCl_3$) δ: 7.28 – 7.23 (m, 5H), 7.23 – 7.17 (m, 4H).

^{13}C NMR (101 MHz, $CDCl_3$) δ: 148.9, 141.1, 140.0, 133.7, 129.7, 128.5, 128.4, 128.3, 128.0, 114.2 (t, J = 3.9 Hz).

^{2}H NMR (77 MHz, $CDCl_3$) δ: 5.49 (s, 2H).

HRMS (ESI) was calculated for $[C_{14}H_9D_2Cl + H]^+$ $[M + H]^+$: m/z 217.0747, found = 217.0749.

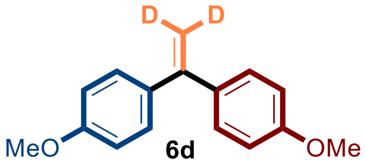

6d

4,4'-(ethene-1,1-diyl-2,2-d2)bis(methoxybenzene) (6d):

Prepared according to general Procedure-C using phosphonium salt (**3o**) (0.2 mmol, 127 mg) product (**6d**) was isolated in (35 mg, 73% yield) as a white solid by column chromatography on silica gel (EtOAc/Hexane = 05:95). 99% Deuterium-incorporation.

R_f = 0.54 (10% EtOAc in hexane).

^{1}H NMR (400 MHz, $CDCl_3$) δ: 7.30 – 7.26 (m, 4H), 6.87 (td, J = 9.2, 3.1 Hz, 4H), 3.83 (s, 6H).

The spectral data are consistent with those reported in the literature. [17]

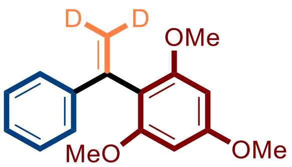

6e

1,3,5-trimethoxy-2-(1-phenylvinyl-2,2-d2)benzene (6e):

Prepared according to general Procedure-C using phosphonium salt (**3t**) (0.2 mmol, 133 mg) product (**6e**) was isolated in (28 mg, 52% yield) as a colorless solid by column chromatography on silica gel (EtOAc/Hexane = 05:95). 99% Deuterium-incorporation.

R_f = 0.58 (15% EtOAc in hexane).

^{1}H NMR (400 MHz, $CDCl_3$) δ: 7.25 – 7.19 (m, 2H), 7.17 – 7.16 (m, 1H), 7.15 – 7.08 (m, 2H), 6.11 (s, 2H), 3.76 (s, 3H), 3.59 (s, 6H).

^{13}C{^{1}H} NMR (101 MHz, $CDCl_3$) δ: 160.7, 158.8, 141.0, 140.9, 128.1, 127.2, 125.9, 112.5, 91.1, 56.1, 55.5.

^{2}H NMR (77 MHz, $CDCl_3$) δ 5.97 (s, 1H), 5.23 (s, 1H).

HRMS (ESI) was calculated for $[C_{17}H_{16}D_2O_3 + H]^+$ $[M + H]^+$: m/z 273.1454, found = 273.1445.

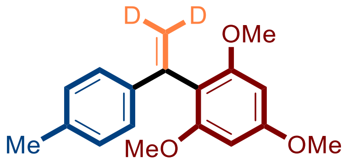

6f

1,3,5-trimethoxy-2-(1-(p-tolyl)vinyl-2,2-d2)benzene (6f):

Prepared according to general Procedure-C using phosphonium salt (**3z**) (0.2 mmol, 136 mg) product (**6f**) was isolated in (42 mg, 74% yield) as a white solid by column chromatography on silica gel (EtOAc/Hexane = 05:95). 97% Deuterium-incorporation.

R_f = 0.52 (15% EtOAc in hexane).

^{1}H NMR (400 MHz, $CDCl_3$) δ: 7.25 – 7.20 (m, 2H), 7.08 – 7.04 (m, 2H), 6.21 (s, 2H), 5.91 (s, 0.03H, 97% labeled), 5.13 (s, 0.03H, 97% labeled), 3.86 (s, 3H), 3.70 (s, 6H), 2.32 (s, 3H).

^{13}C{^{1}H} NMR (101 MHz, $CDCl_3$) δ: 160.6 (d, J = 4.2 Hz), 158.9 – 158.7 (m), 140.9, 137.9, 136.9, 128.8, 125.8, 115.3, 113.0 – 112.3 (m), 91.1, 56.1, 55.4, 21.2.

HRMS (ESI) was calculated for $[C_{18}H_{18}D_2O_3 + H]^+$ $[M + H]^+$: m/z 287.1610, found = 287.1605.

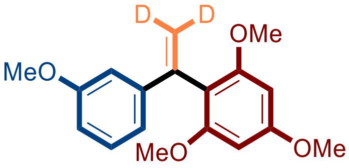

6g

1,3,5-trimethoxy-2-(1-(3-methoxyphenyl)vinyl-2,2-d2)benzene (6g):

Prepared according to general Procedure-C using phosphonium salt (**3c**) (0.2 mmol, 140 mg) product (**6g**) was isolated in (24 mg, 40% yield) as a colorless oil by column chromatography on silica gel (EtOAc/Hexane = 05:95). 99% Deuterium-incorporation.

R_f = 0.29 (10% EtOAc in hexane).

^{1}H NMR (400 MHz, $CDCl_3$) δ: 7.16 (t, J = 8.1 Hz, 1H), 6.94 – 6.87 (m, 2H), 6.77 (ddd, J = 8.2, 2.6, 1.0 Hz, 1H), 6.20 (s, 2H), 3.85 (s, 3H), 3.77 (s, 3H), 3.69 (s, 6H).

^{13}C{^{1}H} NMR (101 MHz, $CDCl_3$) δ: 160.7, 159.5, 158.8, 142.5, 141.1, 129.0, 118.7, 116.6, 112.5, 111.9, 91.1, 56.1, 55.5, 55.3.

HRMS (ESI) was calculated for $[C_{18}H_{18}D_2O_4+H]^+$ $[M+H]^+$: m/z 287.1610, found = 287.1605.

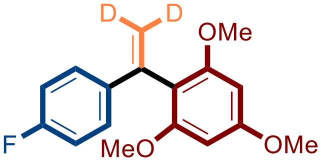

6h

2-(1-(4-fluorophenyl)vinyl-2,2-d2)-1,3,5-trimethoxybenzene (6h):

Prepared according to general Procedure-C using phosphonium salt (**3u**) (0.2 mmol, 137 mg) product (**6h**) was isolated in (38 mg, 66% yield) as a colorless oil by column chromatography on silica gel (EtOAc/Hexane = 05:95). 99% Deuterium-incorporation.

R_f = 0.40 (10% EtOAc in hexane).

^{1}H NMR (400 MHz, $CDCl_3$) δ: 7.32 – 7.26 (m, 2H), 7.01 – 6.87 (m, 2H), 6.21 (s, 2H), 3.86 (s, 3H), 3.70 (s, 6H).

^{13}C{^{1}H} NMR (101 MHz, $CDCl_3$) δ: 163.5, 161.1, 160.8, 160.8, 158.8, 158.7, 140.1 (d, J = 2.1 Hz), 140.0, 137.1 (d, J = 3.0 Hz), 127.5 (d, J = 8.0 Hz), 116.4 – 115.5 (m), 115.0, 114.8, 112.2, 91.0, 56.1, 55.4.

^{19}F NMR (376 MHz, $CDCl_3$) δ: −116.0.

HRMS (ESI) was calculated for $[C_{17}H_{15}D_2FO_3+H]^+$ $[M+H]^+$: m/z 291.1360, found = 291.1353.

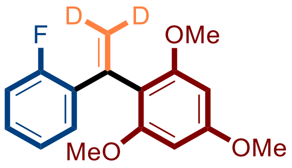

6i

2-(1-(2-fluorophenyl)vinyl-2,2-d2)-1,3,5-trimethoxybenzene (6i):

Prepared according to general Procedure-C using phosphonium salt (**3ad**) (0.2 mmol, 137 mg) product (**6i**) was isolated in (43 mg, 74% yield) as a white solid by column chromatography on silica gel (EtOAc/Hexane = 05:95). 97% Deuterium-incorporation.

R_f = 0.32 (10% EtOAc in hexane).

^{1}H NMR (400 MHz, $CDCl_3$) δ: 7.18 – 7.11 (m, 1H), 7.09 – 7.04 (m, 1H), 7.04 – 6.95 (m, 2H), 6.18 (s, 2H), 5.95 (d, J = 1.4 H, 0.04H, 97% labeled), 5.44 (d, J = 1.4 H, 0.03H, 97% labeled) 3.84 (s, 3H), 3.69 (s, 6H).

^{13}C{^{1}H} NMR (101 MHz, $CDCl_3$) δ: 161.5, 160.7, 160.7, 159.6, 158.7, 135.7, 130.0 (d, J = 3.6 Hz), 128.1 (d, J = 8.4 Hz), 123.6 (d, J = 3.7 Hz), 121.7 (m), 115.8 (d, J = 23.4 Hz), 113.0, 91.0, 56.1, 55.4.

^{19}F NMR (376 MHz, $CDCl_3$) δ: −115.7.

HRMS (ESI) was calculated for $[C_{17}H_{15}D_2FO_3+H]^+$ $[M+H]^+$: m/z 291.1360, found = 291.1353.

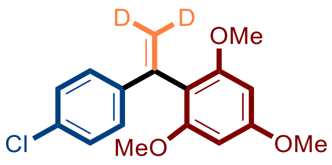

6j

2-(1-(4-chlorophenyl)vinyl-2,2-d2)-1,3,5-trimethoxybenzene (6j):

Prepared according to general Procedure-C using phosphonium salt (**3v**) (0.2 mmol, 140 mg) product (**6j**) was isolated in (30 mg, 49% yield) as a colorless oil by column chromatography on silica gel (EtOAc/Hexane = 05:95). 99% Deuterium-incorporation.

R_f = 0.46 (10% EtOAc in hexane).

^{1}H NMR (400 MHz, $CDCl_3$) δ: 7.25 – 7.16 (m, 4H), 6.20 (s, 2H), 3.85 (s, 3H), 3.69 (s, 6H).

^{13}C{^{1}H} NMR (101 MHz, $CDCl_3$) δ: 160.9, 158.8, 140.0, 139.5, 132.8, 128.2, 127.3, 111.8, 91.0, 56.1, 55.5.

HRMS (ESI) was calculated for $[C_{17}H_{15}D_2ClO_3+H]^+$ $[M+H]^+$: m/z 307.1070, found = 307.1064.

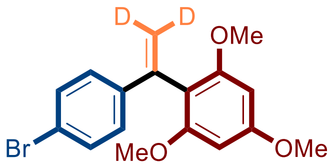

6k

2-(1-(4-bromophenyl)vinyl-2,2-d2)-1,3,5-trimethoxybenzene (6k):

Prepared according to general Procedure-C using phosphonium salt (**3s**) (0.2 mmol, 150 mg) product (**6k**) was isolated in (29 mg, 42% yield) as a colorless oil by column chromatography on silica gel (EtOAc/Hexane = 05:95). 99% Deuterium-incorporation.

R_f = 0.30 (10% EtOAc in hexane).

^{1}H NMR (400 MHz, $CDCl_3$) δ: 7.35 (td, J = 9.1, 2.6 Hz, 2H), 7.18 (td, J = 8.5, 2.7 Hz, 2H), 6.19 (s, 2H), 3.85 (s, 3H), 3.69 (s, 6H).

^{13}C{^{1}H} NMR (101 MHz, $CDCl_3$) δ: 160.9, 158.8, 140.0, 131.2, 127.6, 121.1, 111.7, 91.0, 56.1, 55.5.

HRMS (ESI) was calculated for $[C_{17}H_{15}D_2BrO_3+H]^+$ $[M+H]^+$: m/z 351.0559, found = 351.0551.

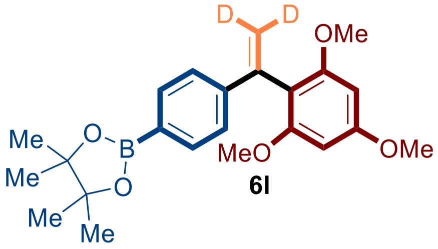

6l

4,4,5,5-tetramethyl-2-(4-(1-(2,4,6-trimethoxyphenyl)vinyl-2,2-d2)phenyl)-1,3,2-dioxaborolane (6l):

Prepared according to general Procedure-C using phosphonium salt (**3aa**) (0.2 mmol, 158 mg) product (**6l**) was isolated in (30 mg, 38% yield) as a colorless oil by column chromatography on silica gel (EtOAc/Hexane = 10:90). 99% Deuterium-incorporation.

R_f = 0.51 (20% EtOAc in hexane).

^{1}H NMR (400 MHz, $CDCl_3$) δ: 7.69 (d, J = 8.4 Hz, 2H), 7.31 (d, J = 8.4 Hz, 2H), 6.20 (s, 2H), 3.86 (s, 3H), 3.67 (s, 6H), 1.33 (s, 12H).

^{13}C{^{1}H} NMR (101 MHz, $CDCl_3$) δ: 160.8, 158.9, 143.8, 134.7, 132.4, 128.7, 125.2, 91.1, 83.7, 56.1, 55.5, 25.0.

^{11}B NMR (128 MHz, $CDCl_3$) δ: 30.9.

HRMS (ESI) was calculated for $[C_{23}H_{27}D_2BO_5 + H]^+$ $[M + H]^+$: m/z 399.2306, found = 399.2299.

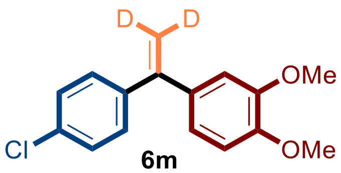

6m

4-(1-(4-chlorophenyl)vinyl-2,2-d2)-1,2-dimethoxybenzene (6m):

Prepared according to general Procedure-C using phosphonium salt (**3k**) (0.2 mmol, 134 mg) product (**6m**) was isolated in (37 mg, 67% yield) as a white solid by column chromatography on silica gel (EtOAc/Hexane = 05:95). 97% Deuterium-incorporation.

R_f = 0.52 (10% EtOAc in hexane).

^{1}H NMR (400 MHz, $CDCl_3$) δ: 7.33 – 7.26 (m, 4H), 6.89 – 6.81 (m, 3H), 5.40 (s, 0.03H, 97% labeled), 5.35 (s, 0.03H, 97% labeled), 3.90 (s, 3H), 3.84 (s, 3H).

^{13}C{^{1}H} NMR (100 MHz, $CDCl_3$) δ: 149.1, 148.7, 148.7, 148.6, 140.2, 133.9, 133.7, 129.7, 128.4, 121.0, 113.6 (m), 111.4, 110.9, 56.1, 56.03.

HRMS (ESI) was calculated for $[C_{16}H_{13}D_2ClO_2 + H]^+$ $[M + H]^+$: m/z 277.0964, found = 277.0960.

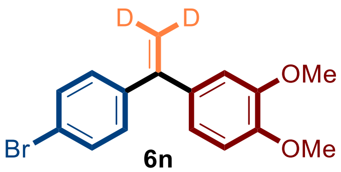

6n

4-(1-(4-bromophenyl)vinyl-2,2-d2)-1,2-dimethoxybenzene (6n):

Prepared according to general Procedure-C using phosphonium salt (**3l**) (0.2 mmol, 143 mg) product (**6n**) was isolated in (36 mg, 56% yield) as a white solid by column chromatography on silica gel (EtOAc/Hexane = 05:95). 99% Deuterium-incorporation.

R_f = 0.37 (10% EtOAc in hexane).

^{1}H NMR (400 MHz, $CDCl_3$) δ: 7.48 – 7.42 (m, 2H), 7.25 – 7.20 (m, 2H), 6.88 – 6.79 (m, 3H), 5.40 (s, 0.01H, 99% labeled), 5.35 (s, 0.01H, 99% labeled), 3.90 (s, 3H), 3.84 (s, 3H).

^{13}C{^{1}H} NMR (100 MHz, $CDCl_3$) δ: 149.1, 148.8, 148.7, 140.6, 133.8, 131.4, 130.1, 121.9, 121.0, 111.4, 110.9, 56.1, 56.0.

HRMS (ESI) was calculated for $[C_{16}H_{13}D_2BrO_2]^+$ $[M]^+$: m/z 320.0381, found = 320.0392.

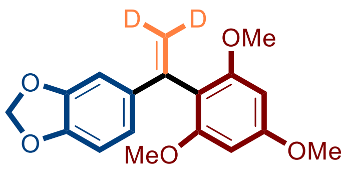

6o

5-(1-(2,4,6-trimethoxyphenyl)vinyl-2,2-d2)benzo[d][1,3]dioxole (6o):

Prepared according to general Procedure-C using phosphonium salt (**3ac**) (0.2 mmol, 142 mg) product (**6o**) was isolated in (50 mg, 78% yield) as a white solid by column chromatography on silica gel (EtOAc/Hexane = 10:90). 99% Deuterium-incorporation.

R_f = 0.51 (20% EtOAc in hexane).

^{1}H NMR (400 MHz, $CDCl_3$) δ: 6.88 (dd, J = 1.8, 0.4 Hz, 1H), 6.77 (dd, J = 6.3, 1.8 Hz, 1H), 6.68 (dd, J = 8.1, 0.4 Hz, 1H), 6.20 (s, 2H), 5.92 (s, 2H), 3.85 (s, 3H), 3.71 (s, 6H).

^{13}C{^{1}H} NMR (100 MHz, $CDCl_3$) δ: 160.7, 158.7, 147.6, 146.9, 140.5, 135.3, 119.7, 115.1 – 114.4 (m), 112.5, 107.9, 106.4, 101.0, 91.0, 56.1, 55.4.

HRMS (ESI) was calculated for $[C_{18}H_{16}D_2O_5 + H]^+$ $[M + H]^+$: m/z 317.1352, found = 317.1344.

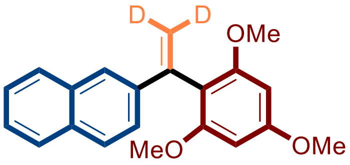

6p

2-(1-(2,4,6-trimethoxyphenyl)vinyl-2,2-d2)naphthalene (6p):

Prepared according to general Procedure-C using phosphonium salt (**3ab**) (0.2 mmol, 140 mg) product (**6p**) was isolated in (41 mg, 64% yield) as a white solid by column chromatography on silica gel (EtOAc/Hexane = 05:95). 99% Deuterium-incorporation.

R_f = 0.42 (10% EtOAc in hexane).

^{1}H NMR (400 MHz, $CDCl_3$) δ: 7.81 – 7.70 (m, 3H), 7.65 – 7.60 (m, 2H), 7.43 – 7.37 (m, 2H), 6.25 (s, 2H), 3.89 (s, 3H), 3.69 (s, 6H).

^{13}C{^{1}H} NMR (100 MHz, $CDCl_3$) δ: 160.8, 158.9, 141.0, 133.6, 132.9, 128.4, 127.6, 127.6, 125.8, 125.5, 124.8, 124.4, 91.12, 56.1, 55.0.

HRMS (ESI) was calculated for $[C_{21}H_{18}D_2O_5 + H]^+$ $[M + H]^+$: m/z 323.1616, found = 323.1594.

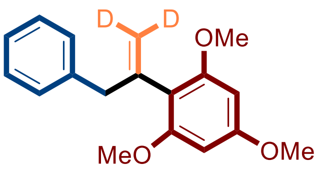

6q

1,3,5-trimethoxy-2-(3-phenylprop-1-en-2-yl-1,1-d2)benzene (6q):

Prepared according to general Procedure-C using phosphonium salt (**3ah**) (0.2 mmol, 136 mg) product (**6q**) was isolated in (45 mg, 79% yield) as a colorless oil by column chromatography on silica gel (EtOAc/Hexane = 05:95). 99% Deuterium-incorporation.

R_f = 0.37(10% EtOAc in hexane).

^{1}H NMR (400 MHz, $CDCl_3$) δ: 7.25 – 7.19 (m, 2H), 7.19 – 7.11 (m, 3H), 6.11 (s, 2H), 3.80 (s, 3H), 3.69 (s, 6H), 3.59 (s, 2H).

^{13}C{^{1}H} NMR (100 MHz, $CDCl_3$) δ: 160.2, 158.2, 142.7, 140.1, 129.6, 127.9, 125.8, 113.4, 90.9, 56.0, 55.4, 43.6.

HRMS (ESI) was calculated for $[C_{18}H_{18}D_2O_3 + H]^+$ $[M + H]^+$: m/z 287.1610, found = 287.1617.

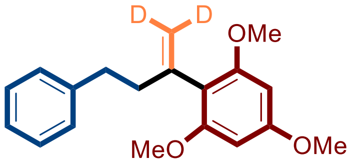

6r

1,3,5-trimethoxy-2-(4-phenylbut-1-en-2-yl-1,1-d2)benzene (6r):

Prepared according to general Procedure-C using phosphonium salt (**3ai**) (0.2 mmol, 140 mg) product (**6r**) was isolated in (35 mg, 58% yield) as a colorless oil by column chromatography on silica gel (EtOAc/Hexane = 05:95). 99% Deuterium-incorporation.

R_f = 0.48(10% EtOAc in hexane).

^{1}H NMR (400 MHz, $CDCl_3$) δ: 7.27 – 7.23 (m, 2H), 7.20 – 7.13 (m, 3H), 6.17 (s, 2H), 3.84 (s, 3H), 3.78 (s, 6H), 2.74 – 2.69 (m, 2H), 2.66 – 2.61 (m, 2H).

^{13}C{^{1}H} NMR (100 MHz, $CDCl_3$) δ: 160.2, 159.8, 158.3, 142.8, 141.5, 129.9, 128.6, 128.3, 125.7, 116.3, 91.1, 90.8, 56.1, 56.0, 55.4, 38.9, 34.4.

HRMS (ESI) was calculated for $[C_{19}H_{20}D_2O_3 + H]^+$ $[M + H]^+$: m/z 301.1767, found = 301.1776.

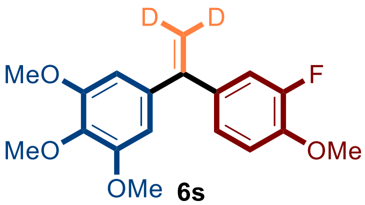

6s

5-(1-(3-fluoro-4-methoxyphenyl)vinyl-2,2-d2)-1,2,3-trimethoxybenzene (6s):

Prepared according to general Procedure-C using phosphonium salt (**3ak**) (0.2 mmol, 143 mg) product (**6s**) was isolated in (40 mg, 63% yield) as a white solid by column chromatography on silica gel (EtOAc/Hexane = 15:85). 99% Deuterium-incorporation.

R_f = 0.55 (30% EtOAc in hexane).

^{1}H NMR (400 MHz, $CDCl_3$) δ: 7.16 – 7.03 (m, 2H), 6.92 (t, *J* = 8.5 Hz, 1H), 6.53 (s, 2H), 3.91 (s, 3H), 3.88 (s, 3H), 3.82 (s, 6H).

^{13}C{^{1}H} NMR (101 MHz, $CDCl_3$) δ: 153.3, 153.0, 150.9, 148.7, 147.5 (d, *J* = 10.8 Hz), 138.0, 137.0 (d, *J* = 2.2 Hz), 134.5, 124.1 (d, *J* = 3.5 Hz), 116.0 (d, *J* = 18.8 Hz), 113.5, 113.0 (d, *J* = 2.2 Hz), 105.8, 61.0, 56.4, 56.2.

^{19}F NMR (376 MHz, $CDCl_3$) δ: –135.6.

HRMS (ESI) was calculated for $[C_{18}H_{17}D_2FO_4]^+$ $[M]^+$: m/z 320.1387, found = 320.1377.

2.4. Progress for the vinylation reaction of benzhydryl phosphonium salt 3p to produce 5j:

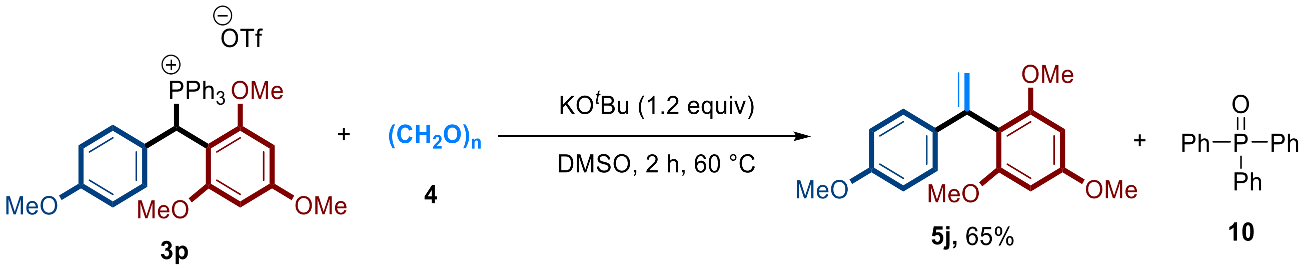

Inside a 15 mL screw-cap vial, benzhydryl phosphonium salt **3p** (0.2 mmol, 1.0 equiv), was added and taken inside the glovebox. Then, 2 mL of DMSO and KO^{t}Bu (0.24 mmol, 1.2 equiv) were added, and the mixture was stirred at room temperature for five minutes. Then powder Paraformaldehyde (1.0 mmol, 5 equivalents) was added, the vial was sealed and taken out of the glove box and stirred for 2 h at 60 °C. After cooling to room temperature, the mixture was quenched with H_2O, and the aqueous phase was extracted with EtOAc (2 x 5 mL). The combined organic phases were washed with brine, dried over $NaSO_4$, filtered and concentrated under reduced pressure (by evaporator). and the crude mixture **5j** was analyzed by NMR.

Observation: Crude P-NMR analysis showed the disappearance of 23.8 ppm peak which is associated with starting phosphonium salt **3p**. And the appearance of 29.2 ppm peak which is associated with triphenyl phosphonium oxide **10** (Ph_3P=O).

^{31}P NMR spectrum (162 MHz, $CDCl_3$) of the crude reaction mixture of **5j**:

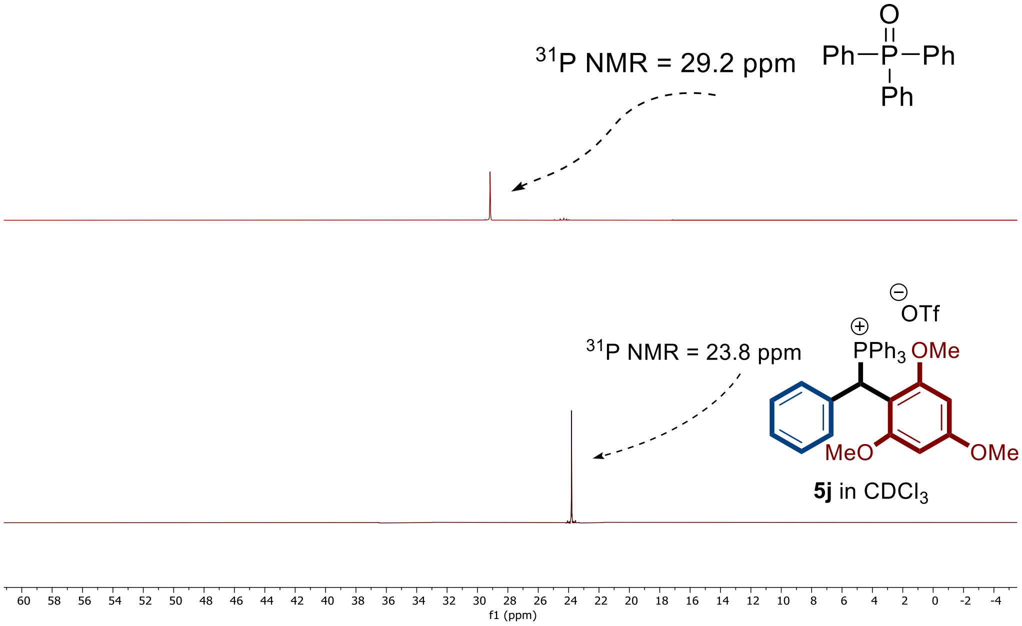

2.5. Procedures and Characterizations for Synthetic Application Products (11-17)

General Procedure-D for the preparation of product (11)

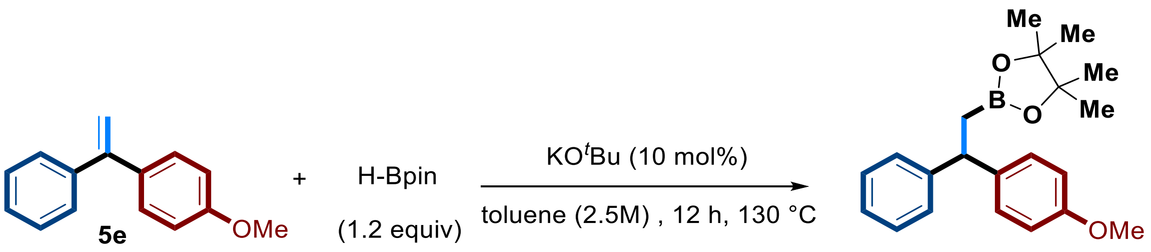

11, 73%

2-(4-methoxyphenyl)-2-phenylethyl)-4,4,5,5-tetramethyl-1,3,2-dioxaborolane (11):

A 15 mL screw-cap vial containing a Teflon-coated magnetic stirring bar was charged with KO^{t}Bu (3.2 mg, 0.025 mmol, 10 mol%), toluene (0.1 mL), H-Bpin (12 µL, 0.3 mmol, 1.2 equiv), and the mixture was stirred at room temperature for five minutes. Then 1,1-diarylethylenes **5e** (52 mg, 0.25 mmol, 1 equiv) was added, the vial was sealed and taken out of the glove box and stirred at 130 °C for 12 hours. After that, the mixture was allowed to cool to room temperature, and quenched with HCl (1.0 M in EtOAc), filtered through short pad of silica gel with EtOAc. The filtrate was concentrated under reduced pressure (by evaporator) to give the crude material, that was further purified by column chromatography, to give **11** (40 mg, 73% yield) as a colorless solid by column chromatography on silica gel (EtOAc/Hexane = 05:95).

R_f = 0.39 (10% EtOAc in hexane).

^{1}H NMR (400 MHz, $CDCl_3$) δ: 7.26 – 7.20 (m, 4H), 7.20 – 7.16 (m, 2H), 7.15 – 7.09 (m, 1H), 6.84 – 6.73 (m, 2H), 4.23 (t, J = 8.5 Hz, 1H), 3.75 (s, 3H), 1.55 (d, J = 4.5 Hz, 2H), 1.06 (s, 12H). The spectral data are consistent with those reported in the literature. [19]

General Procedure-E for the preparation of product (12)

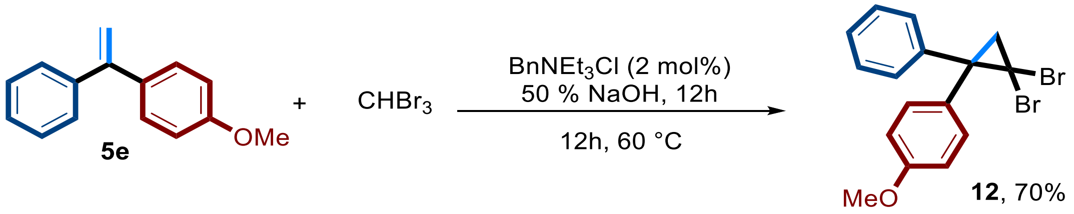

1-(2,2-dibromo-1-phenylcyclopropyl)-4-methoxybenzene (12):

A 15 mL screw-cap vial containing a Teflon-coated magnetic stirring bar was charged with 1,1-diarylethylenes **5e** (42 mg, 0.2 mmol, 1 equiv), $CHBr_3$ (27 µL, 0.3 mmol, 1.5 equiv), $BnNEt_3Cl$ (1 mg, 2 mol%), and the mixture was stirred at room temperature for 1 hour. Then a solution of NaOH 50% in water was added, and the vial was stirred at 60 °C for 12 hours. After that, the mixture was allowed to cool to room temperature, extracted with DCM (3 × 5 mL). The combined organic phases were dried over Na_2SO_4, filtered, and concentrated under reduced pressure (by evaporator) to give the crude material, that was further purified by column chromatography, to give **12** (54 mg, 70% yield) as a white solid by column chromatography on silica gel (EtOAc/Hexane = 05:95).

R_f = 0.50 (10% EtOAc in hexane).

^{1}H NMR (400 MHz, $CDCl_3$) δ: 7.53 – 7.48 (m, 2H), 7.47 – 7.41 (m, 2H), 7.36 – 7.30 (m, 2H), 7.27 – 7.21 (m, 1H), 6.88 – 6.83 (m, 2H), 3.77 (s, 3H), 2.46 (d, J = 0.9 Hz, 2H).

The spectral data are consistent with those reported in the literature. [20]

General Procedure-F for the preparation of product (13)

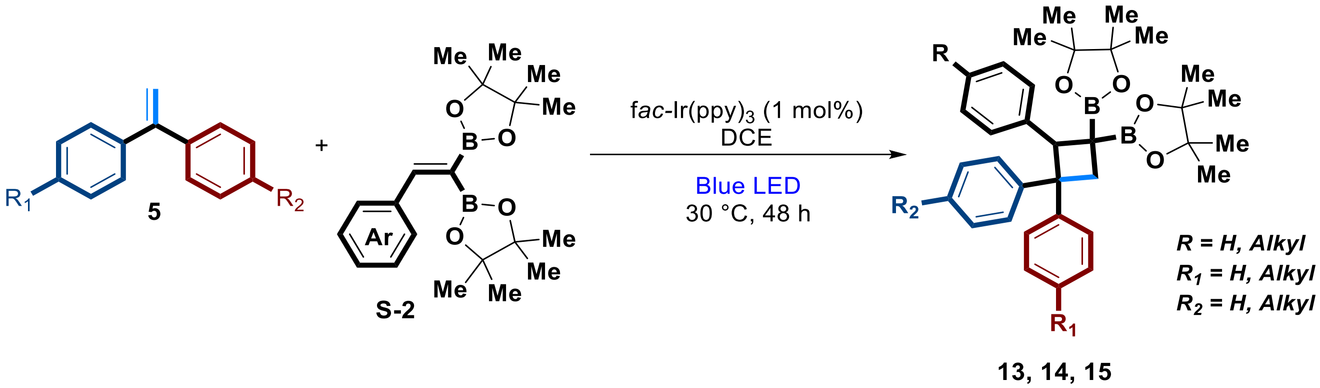

Products (**13-15**) were prepared according to a literature reported procedure.[21] Inside the glove box, into a 7 mL screw-cap vial equipped with a stir bar, *gem*-diborylalkene (**S-2**) (0.2 mmol, 1 equiv) and *fac*-Ir(ppy)$_3$ (1.0 mg, 0.01 mmol, 1 mol%) were dissolved in DCE. Then the appropriate 1,1-diarylethylenes (**5**) (1 mmol, 5 equiv) was added, and the vial was sealed and taken out of the glove box to stir under irradiation of 450 nm Blue LED for 48 hours. A cooling fan was necessary to maintain a temperature of 30 °C. After completion, the mixture was concentrated under reduced pressure (by evaporator) and the crude material was purified by flash column chromatography to give products **13, 14, 15**.

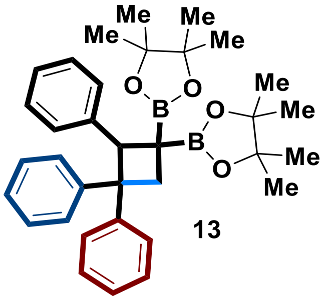

13

***2,2'-(2,3,3-triphenylcyclobutane-1,1-diyl)bis(4,4,5,5-tetramethyl-1,3,2-dioxaborolane) (13)*:** Prepared according to general Procedure-F, using substrates **S-2** and **5a**, product **13** was isolated in (76 mg, 71% yield), as a white solid by flash chromatography on silica gel (EtOAc/Hexane = 05:95).

R_f = 0.44 (10% EtOAc in hexane).

^{1}H NMR (400 MHZ, $CDCl_3$) δ: 7.63 - 7.55 (m, 2H), 7.23 - 7.10 (m, 4H), 7.04 - 6.94 (m, 3H), 6.93 - 6.81 (m, 3H), 6.81 - 6.67 (m, 3H), 4.65 (d, J = 2.6 Hz, 1H), 3.64 (d, J = 11.0 Hz, 1H), 3.19 (dd, J = 11.0, 2.6 Hz, 1H), 0.96 (s, 6H), 0.85 (s, 6H), 0.80 (s, 6H), 0.77 (s, 6H).

The spectral data are consistent with those reported in the literature.[21]

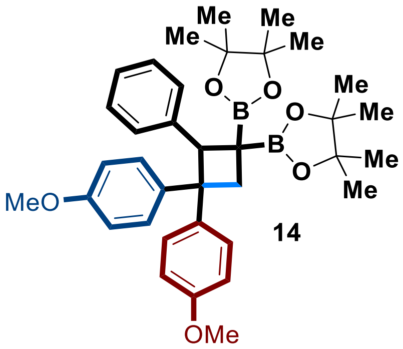

14

2,2'-(3,3-bis(4-methoxyphenyl)-2-phenylcyclobutane-1,1-diyl)bis(4,4,5,5-tetramethyl-1,3,2-dioxaborolane) (14)**:**

Prepared according to general Procedure-F, using substrates **S-2** and **5d**, product **14** was isolated in (64 mg, 54% yield), as a yellow solid by flash chromatography on silica gel (EtOAc/Hexane = 05:95).

R_f = 0.22 (15% EtOAc in hexane).

^{1}H NMR (400 MHZ, $CDCl_3$) δ: 7.58 - 7.50 (m, 2H), 7.23 - 7.17 (m, 2H), 7.10 - 7.01 (m, 2H), 7.01 - 6.92 (m, 1H), 6.84 - 6.76 (m, 2H), 6.76 - 6.69 (m, 2H), 6.52 - 6.44 (m, 2H), 4.63 (d, J = 2.5 Hz, 1H), 3.73 (s, 3H), 3.63 (d, J = 10.9 Hz, 1H), 3.60 (s, 3H), 3.17 (dd, J = 10.9, 2.6 Hz, 1H), 1.03 (s, 6H), 0.95 (s, 6H), 0.90 (s, 6H), 0.84 (s, 6H).

The spectral data are consistent with those reported in the literature.[21]

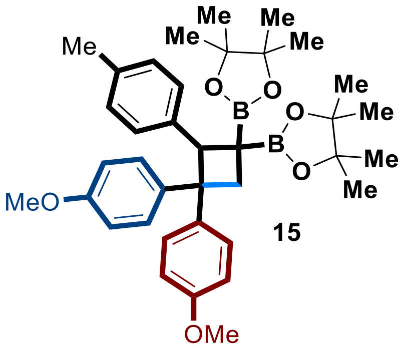

15

***2,2'-(3,3-bis(4-methoxyphenyl)-2-(p-tolyl)cyclobutane-1,1-diyl)bis(4,4,5,5-tetramethyl-1,3,2-dioxaborolane) (3ae)*:**

Prepared according to general Procedure-F, using substrates **S-2’** and **5d**, product **15** was isolated in (49 mg, 40% yield), as a yellow solid by flash chromatography on silica gel (EtOAc/Hexane = 05:95).

R_f = 0.25 (15% EtOAc in hexane).

^{1}H NMR (400 MHZ, $CDCl_3$) δ: 7.54 (d, *J* = 2.2 Hz, 2H), 7.11 - 7.04 (m, 2H), 6.86 (d, *J* = 7.8 Hz, 2H), 6.82 - 6.72 (m, 4H), 6.53 - 6.46 (m, 2H), 4.60 (d, *J* = 2.5 Hz, 1H), 3.72 (s, 3H), 3.61 (d, *J* = 1.4 Hz, 4H), 3.15 (dd, *J* = 10.9, 2.6 Hz, 1H), 2.19 (s, 3H), 1.03 (s, 6H), 0.95 (s, 6H), 0.90 (s, 6H), 0.85 (s, 6H).

The spectral data are consistent with those reported in the literature.[21]

General Procedure-G for the preparation of product (16)

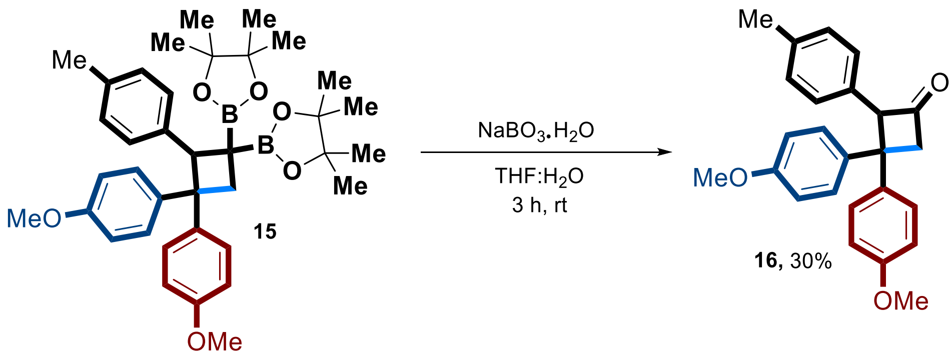

***3,3-bis(4-methoxyphenyl)-2-(p-tolyl)cyclobutan-1-one (16)*:**

Products (**16**) were prepared according to a literature reported procedure. [21] In an open flask, *gem*-diborylcyclobutane **15** (0.1 mmol, 1.0 equiv) was dissolved in THF (1 mL). Then, sodium perborate (0.2 mg, 0.2 mmol, 2 equiv) was added, followed by the addition of H_2O (1 mL), and the reaction was stirred for 3 hours at room temperature. Then, the reaction was quenched with H_2O (2 mL), and the mixture was extracted with EtOAc (3 × 5 mL). The organic layers were dried over $MgSO_4$ and concentrated under reduced pressure using an evaporator to obtain the crude material, that was further purified by column chromatography, to give **16** (12 mg, 30% yield) as a white solid by column chromatography on silica gel (EtOAc/Hexane = 10:90).

R_f = 0.34 (15% EtOAc in hexane).

^{1}H NMR (400 MHZ, $CDCl_3$) δ: 7.40 - 7.32 (m, 2H), 7.04 - 6.87 (m, 4H), 6.86 - 6.76 (m, 4H), 6.68 - 6.60 (m, 2H), 5.21 (d, J = 2.7 Hz, 1H), 3.85 - 3.81 (m, 4H), 3.71 (s, 3H), 3.67 (dd, J = 16.9, 2.7 Hz, 1H), 2.24 (s, 3H).

The spectral data are consistent with those reported in the literature. [21]

General Procedure-H for the preparation of Product (17)

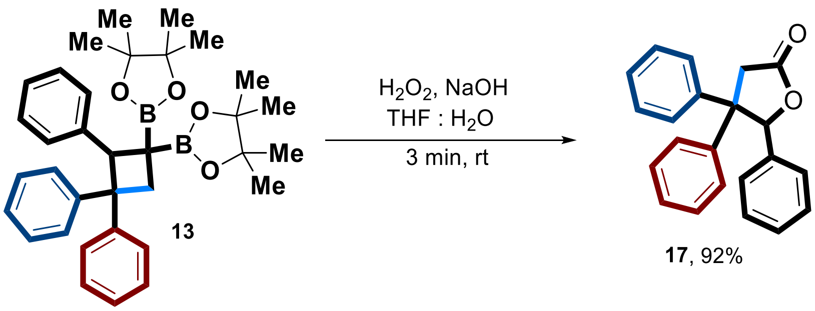

***4,4,5-triphenyldihydrofuran-2(3H)-one (17)*:**

Products (**17**) were prepared according to a literature reported procedure. [21] In an open flask, *gem*-diborylcyclobutane **13** (0.1 mmol, 1.0 equiv) was dissolved in THF (1 mL). Then, H_2O_2 (0.5 mL, 30%) and NaOH (0.35 mL, 3 M) were added, and the reaction was stirred for 3 minutes at room temperature. After the completion of the reaction, the mixture was extracted with ethyl acetate (3 × 5 mL). The organic layers were dried over $MgSO_4$ and concentrated under reduced pressure using an evaporator to obtain the crude material, that was further purified by column chromatography, to give **17** (28 mg, 92% yield) as a white solid by column chromatography on silica gel (EtOAc/Hexane = 10:90).

R_f = 0.43 (15% EtOAc in hexane).

^{1}H NMR (400 MHZ, $CDCl_3$) δ: 7.36 (d, *J* = 4.2 Hz, 4H), 7.32 - 7.26 (m, 1H), 7.15 - 7.08 (m, 6H), 6.92 - 6.86 (m, 2H), 6.85 - 6.79 (m, 2H), 6.34 (s, 1H), 3.68 (d, *J* = 16.9 Hz, 1H), 2.99 (d, *J* = 16.9 Hz, 1H).

The spectral data are consistent with those reported in the literature. [21]

2. NMR Spectra

^{1}H NMR (400 MHz, $CDCl_3$) of compound (**3a**)

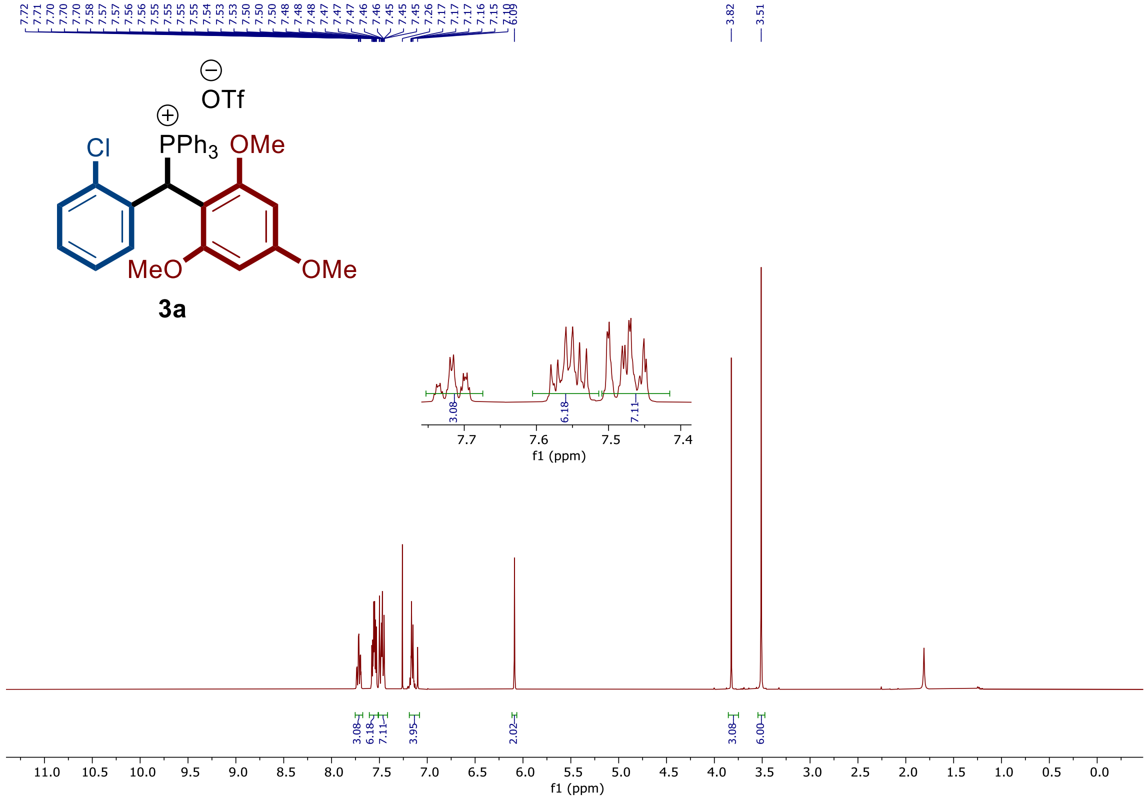

^{13}C{^{1}H} NMR (101 MHz, $CDCl_3$) of compound (**3a**)

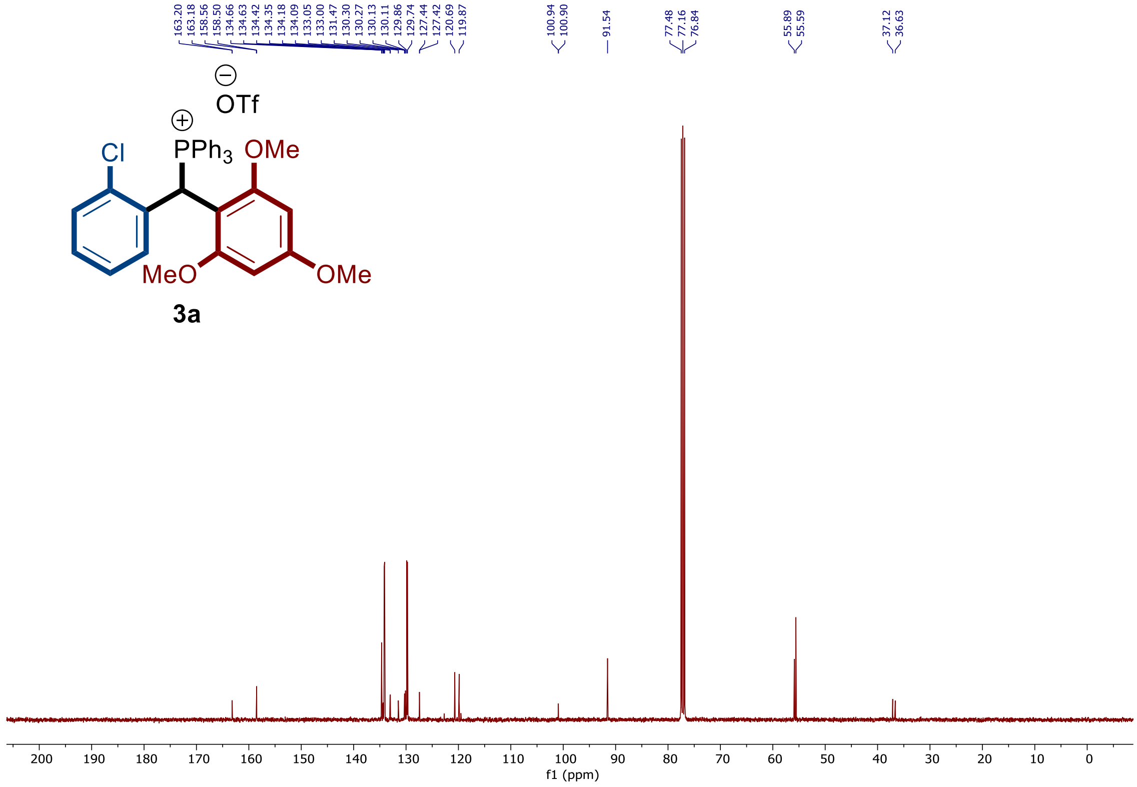

^{31}P NMR (162 MHz, $CDCl_3$) of compound (**3a**)

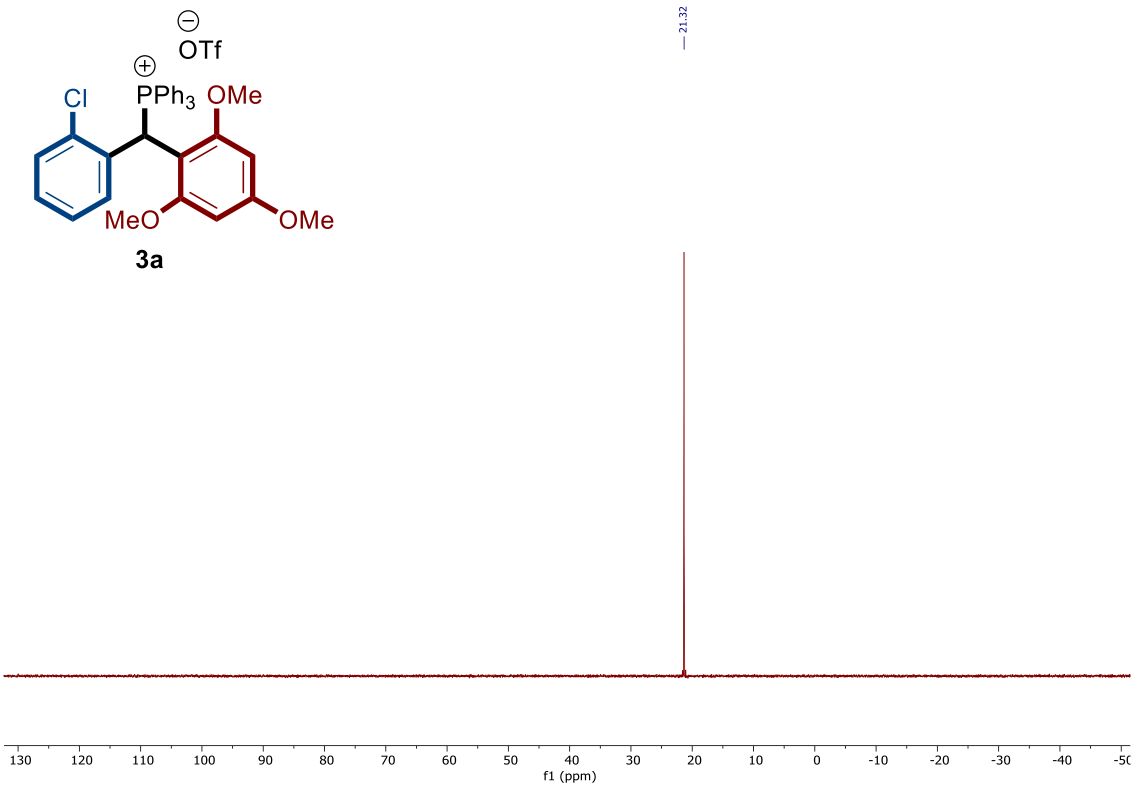

^{19}F NMR (376 MHz, $CDCl_3$) of compound (**3a**)

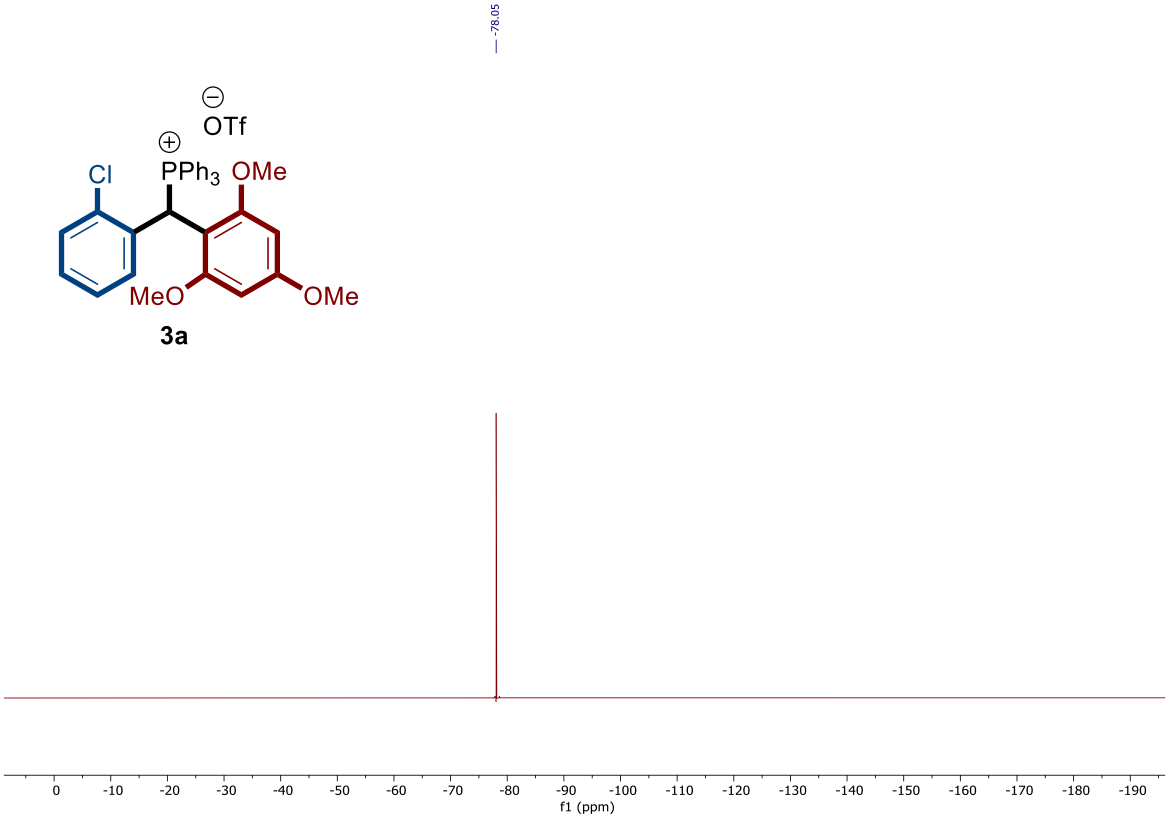

^{1}H NMR (400 MHz, CDCl$_3$) of compound (**3b**)

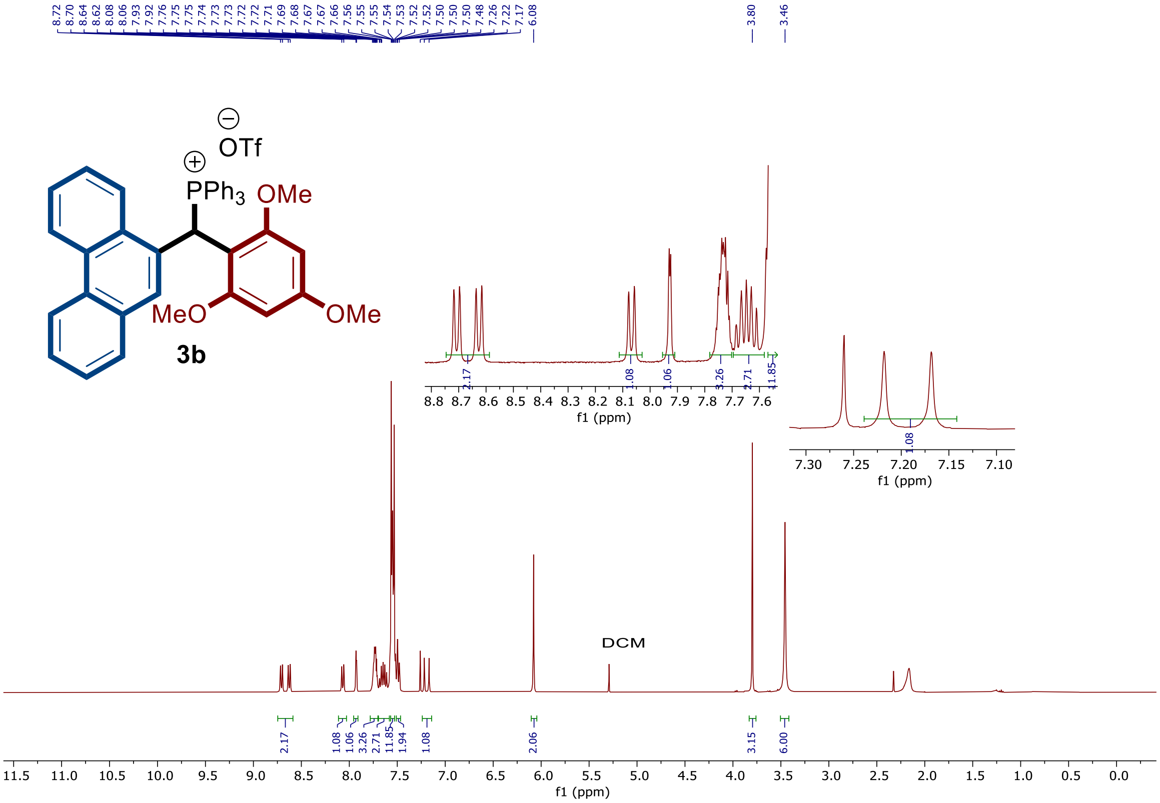

^{13}C{^{1}H} NMR (101 MHz, CDCl$_3$) of compound (**3b**)

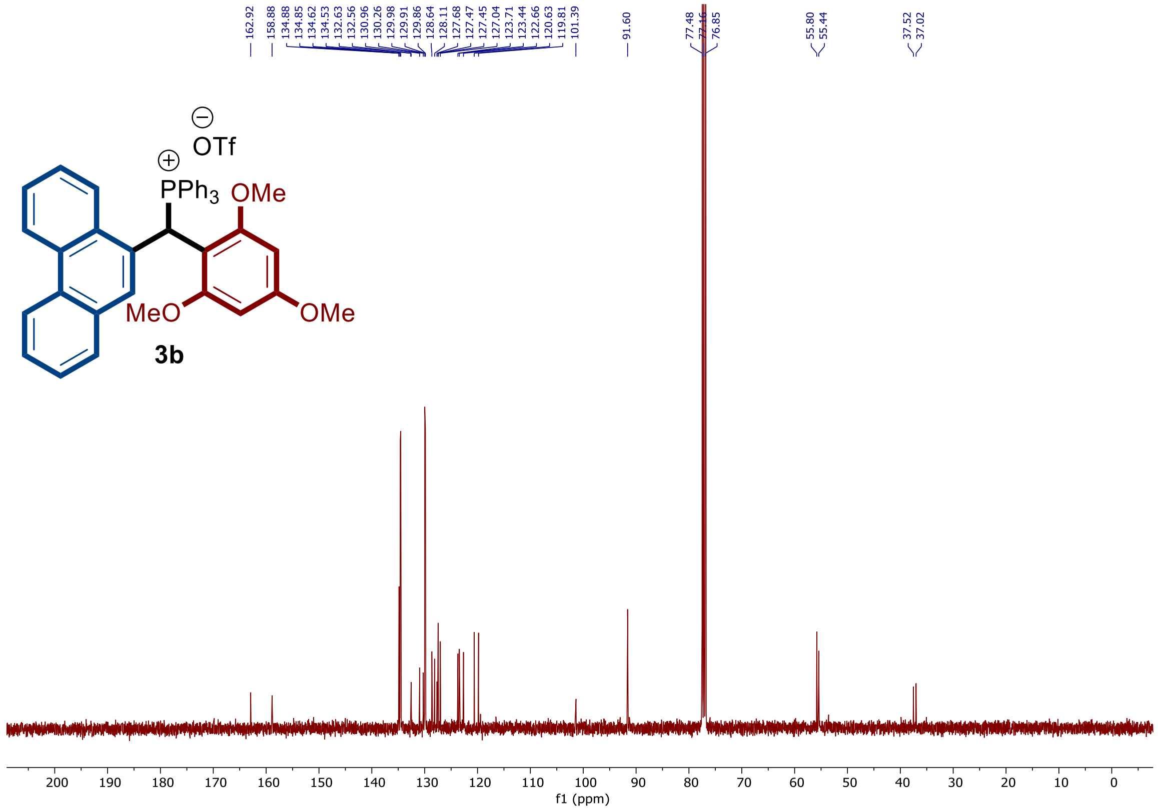

^{31}P NMR (162 MHz, $CDCl_3$) of compound (**3b**)

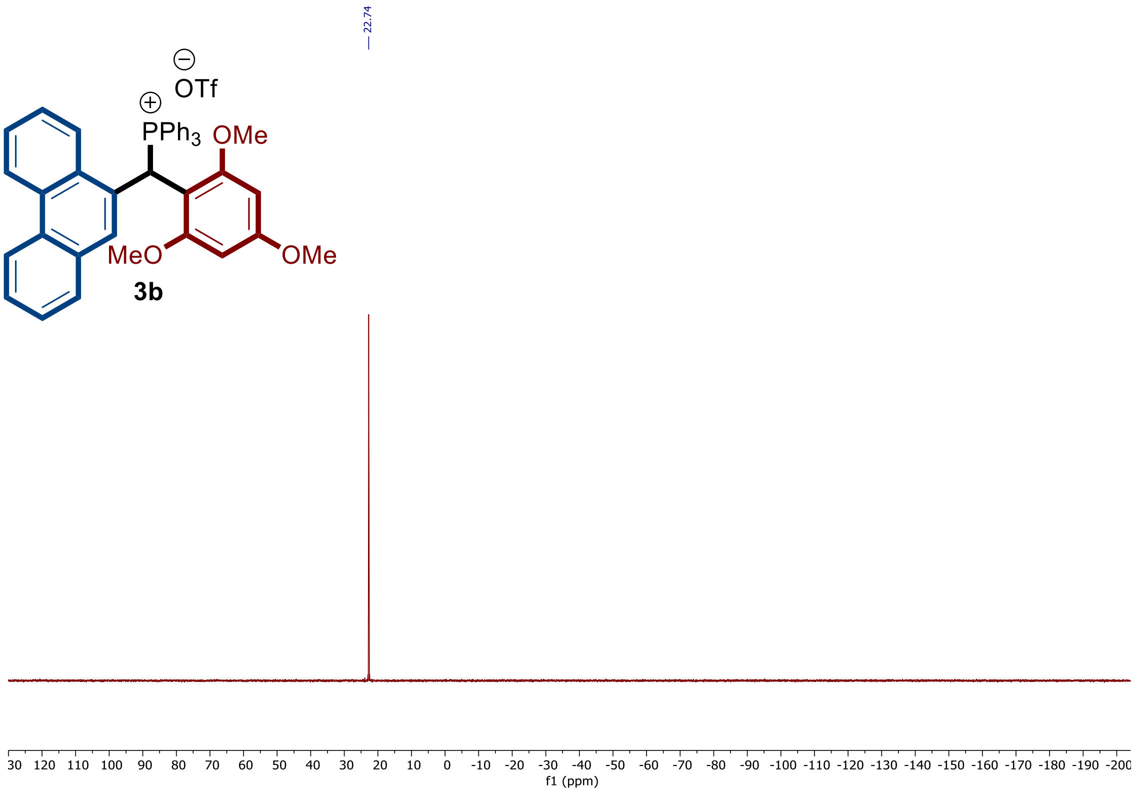

^{19}F NMR (376 MHz, $CDCl_3$) of compound (**3b**)

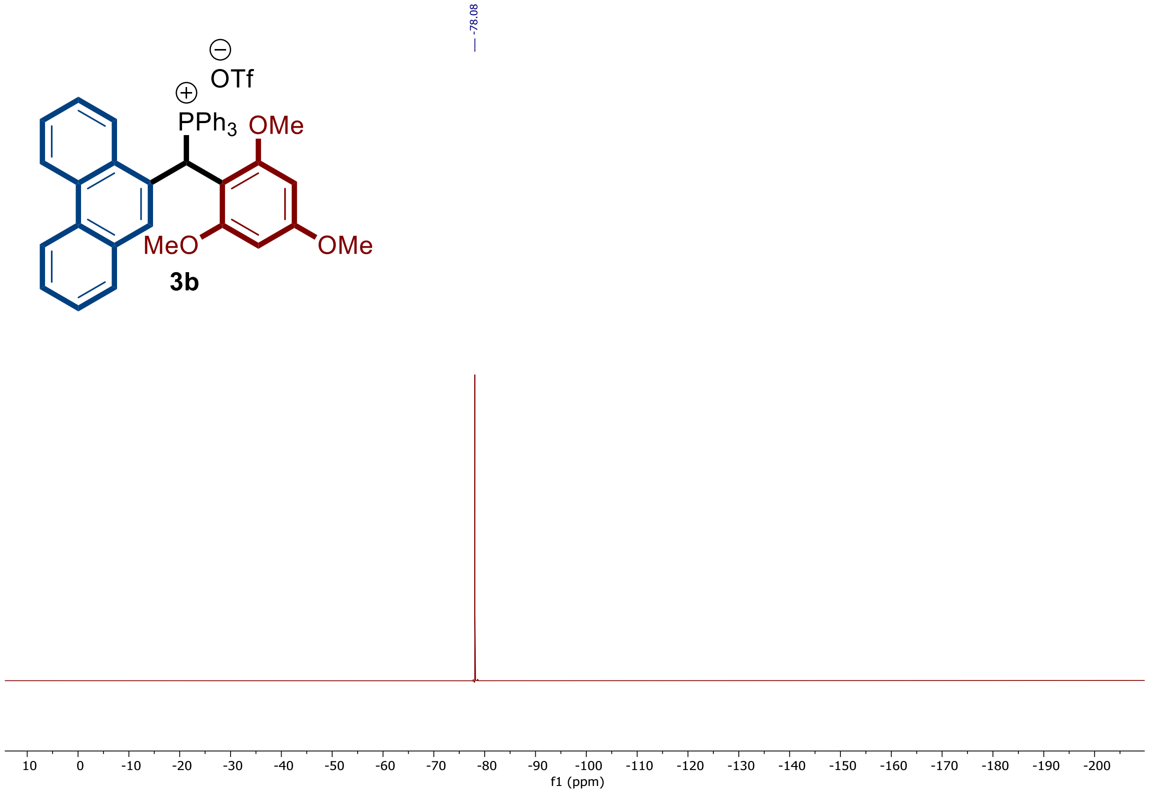

^{1}H NMR (400 MHz, $CDCl_3$) of compound (**3c**)

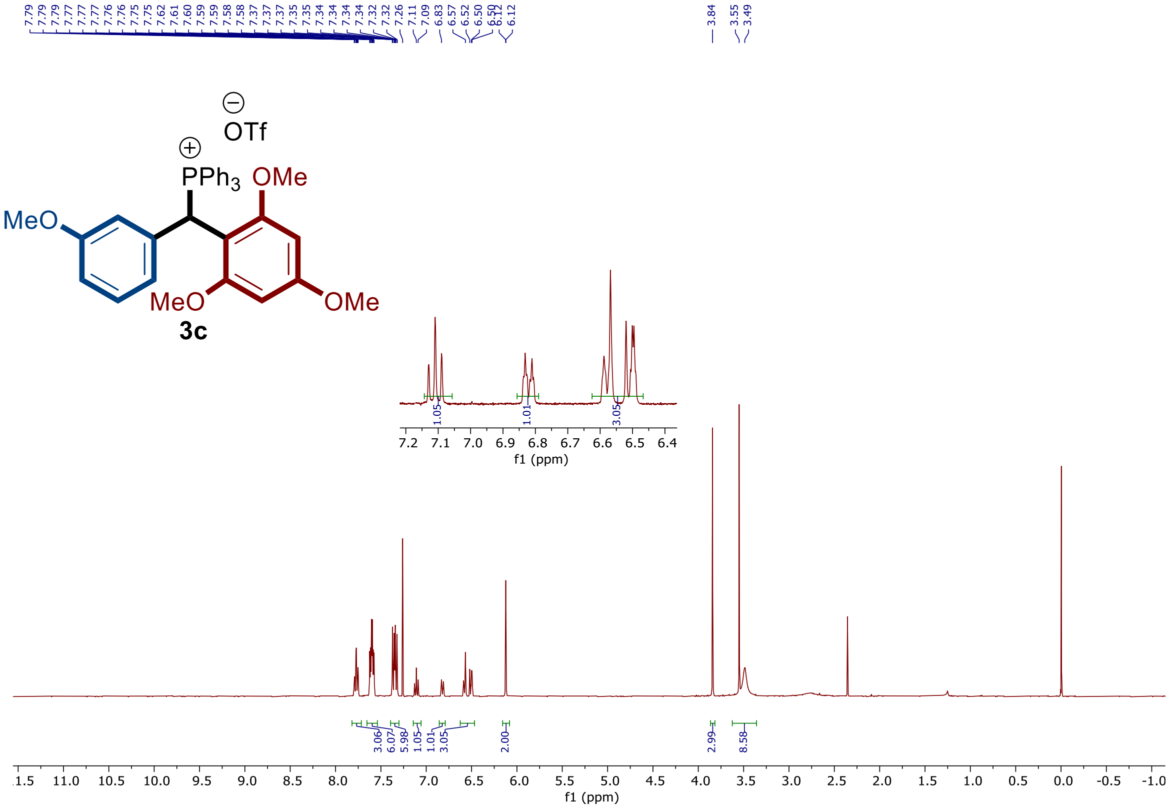

^{13}C{^{1}H} NMR (101 MHz, $CDCl_3$) of compound (**3c**)

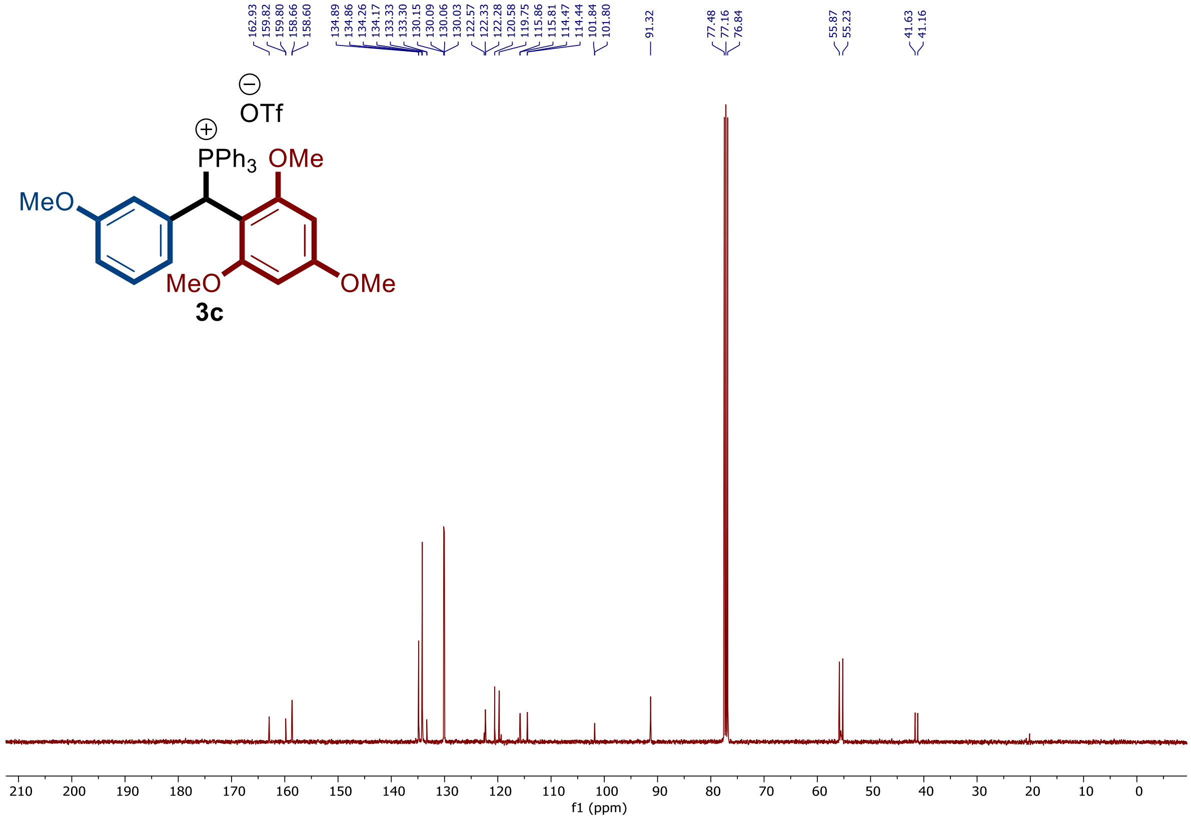

^{31}P NMR (162 MHz, $CDCl_3$) of compound (**3c**)

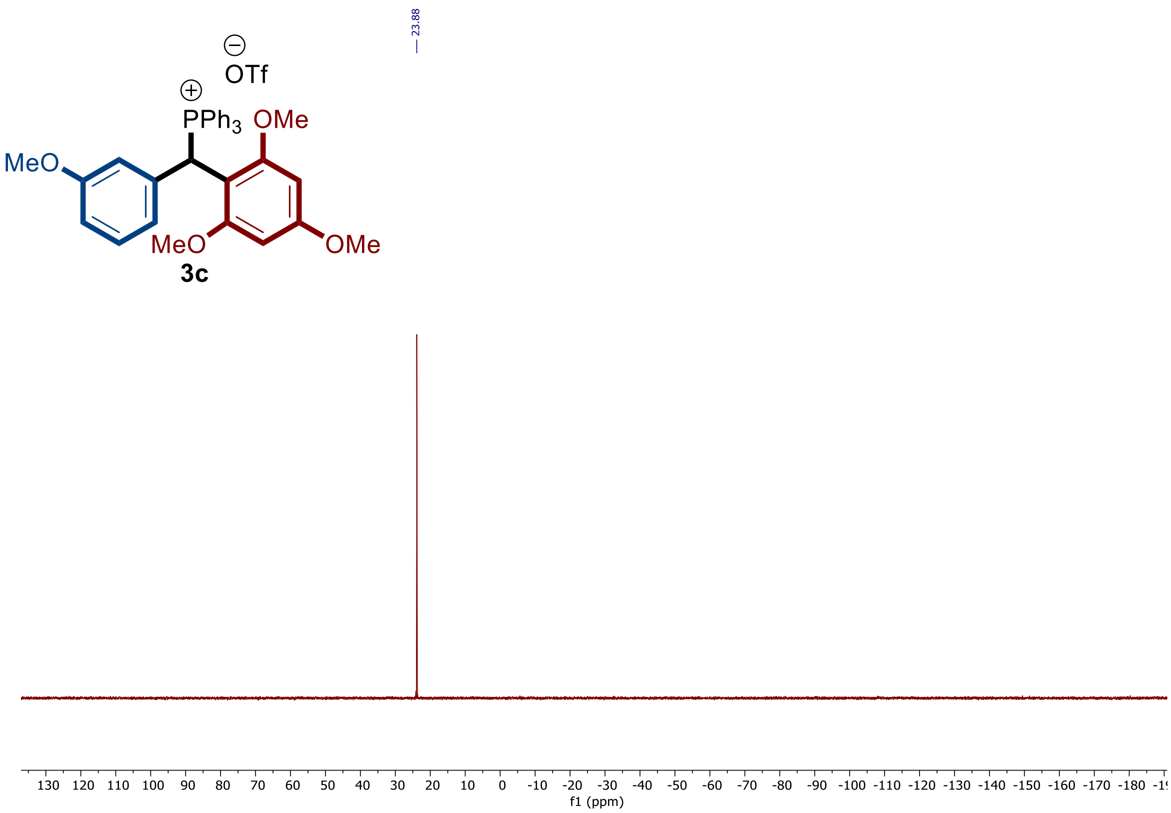

^{19}F NMR (376 MHz, $CDCl_3$) of compound (**3c**)

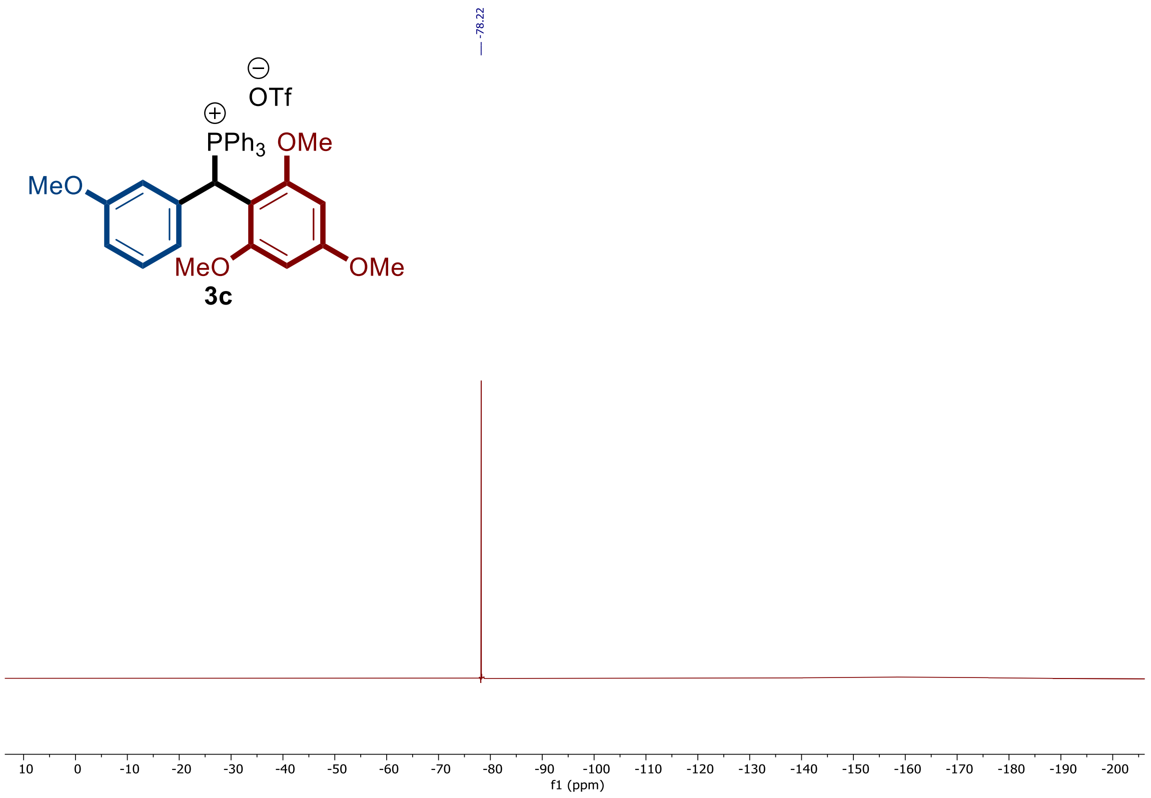

^{1}H NMR (400 MHz, $CDCl_3$) of compound (**3d**)

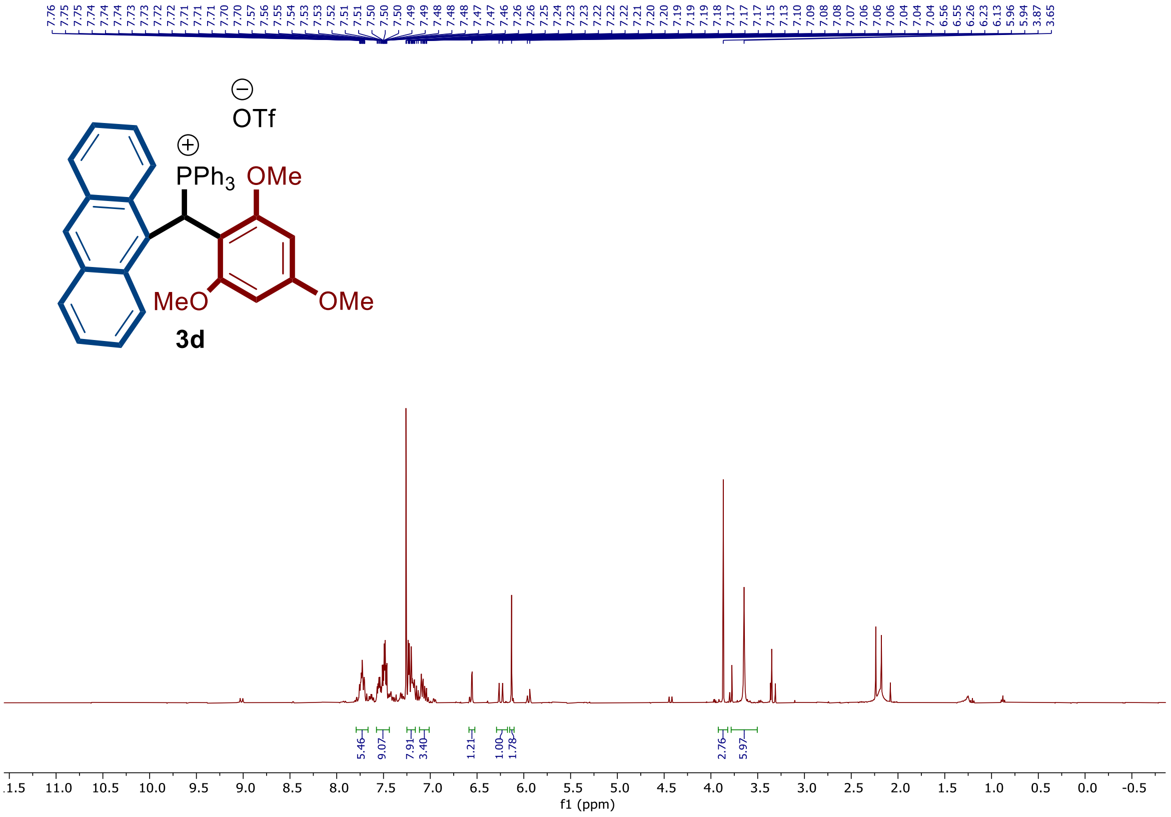

$^{13}C\{^{1}H\}$ NMR (101 MHz, $CDCl_3$) of compound (**3d**)

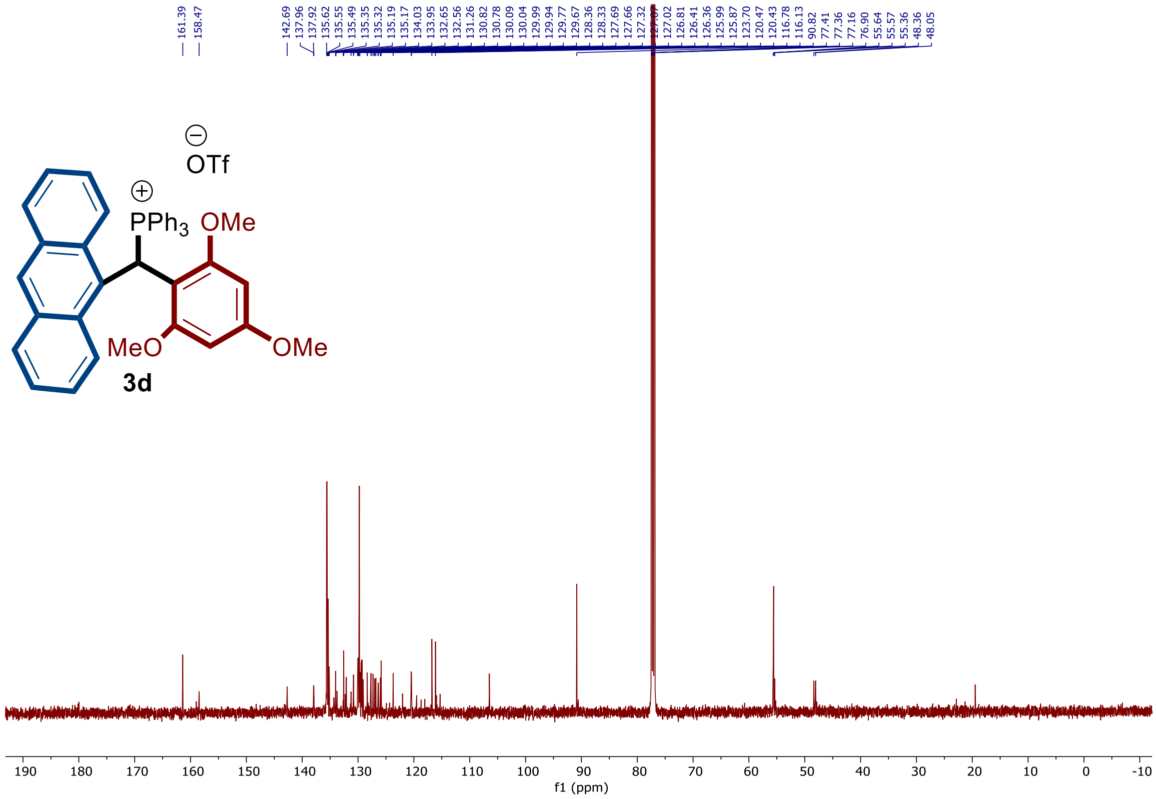

^{31}P NMR (162 MHz, $CDCl_3$) of compound (**3d**)

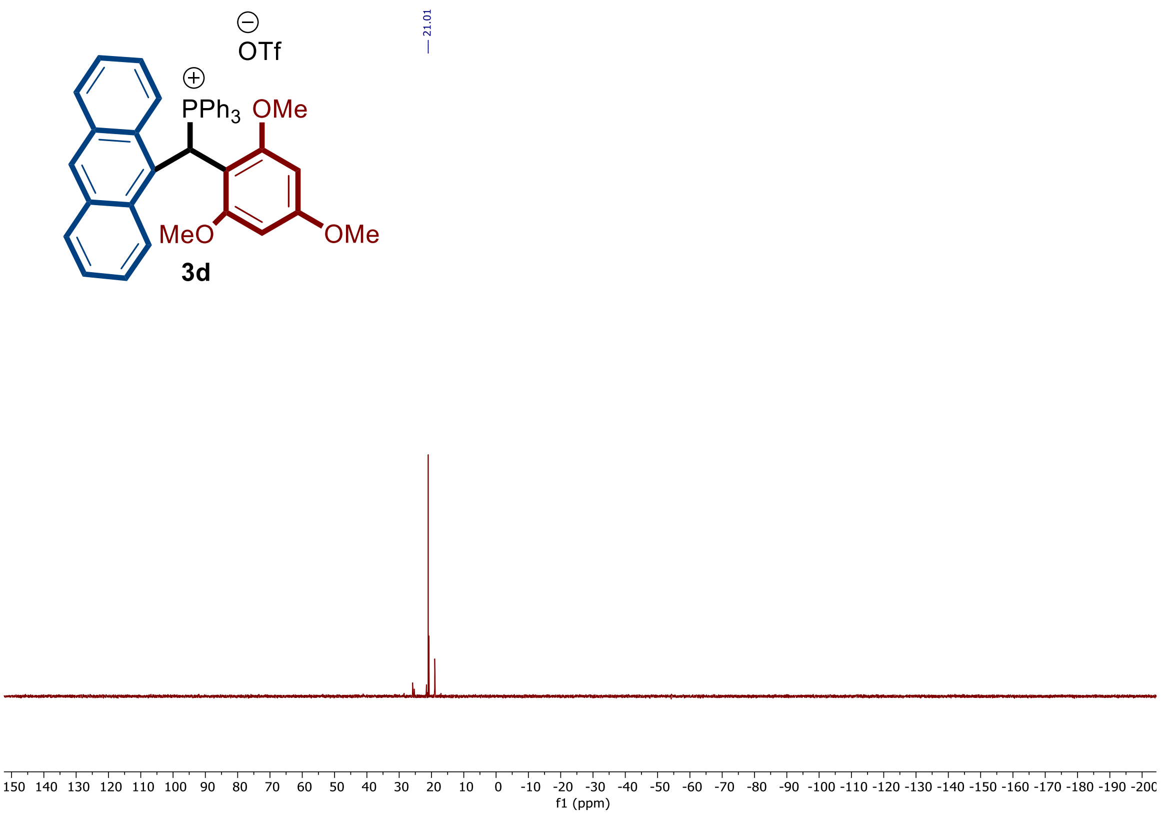

^{19}F NMR (376 MHz, $CDCl_3$) of compound (**3d**)

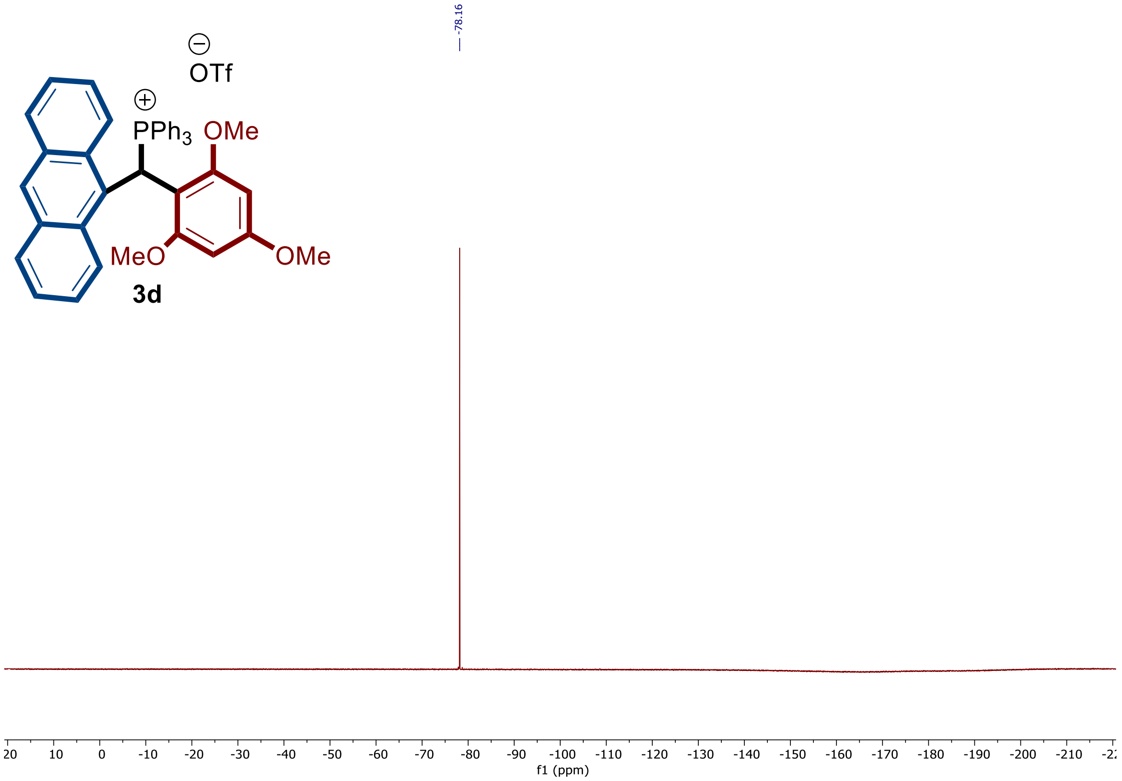

^{1}H NMR (400 MHz, CDCl$_3$) of compound (**3e**)

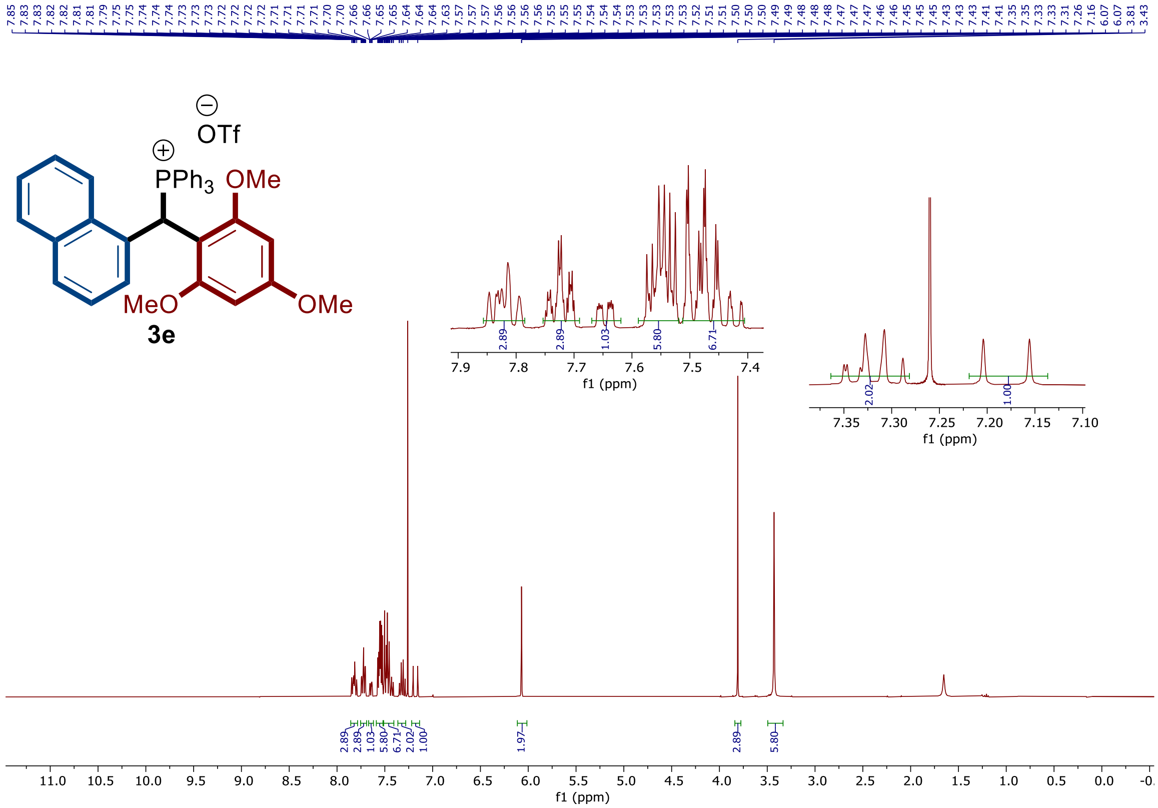

^{13}C{^{1}H} NMR (101 MHz, CDCl$_3$) of compound (**3e**)

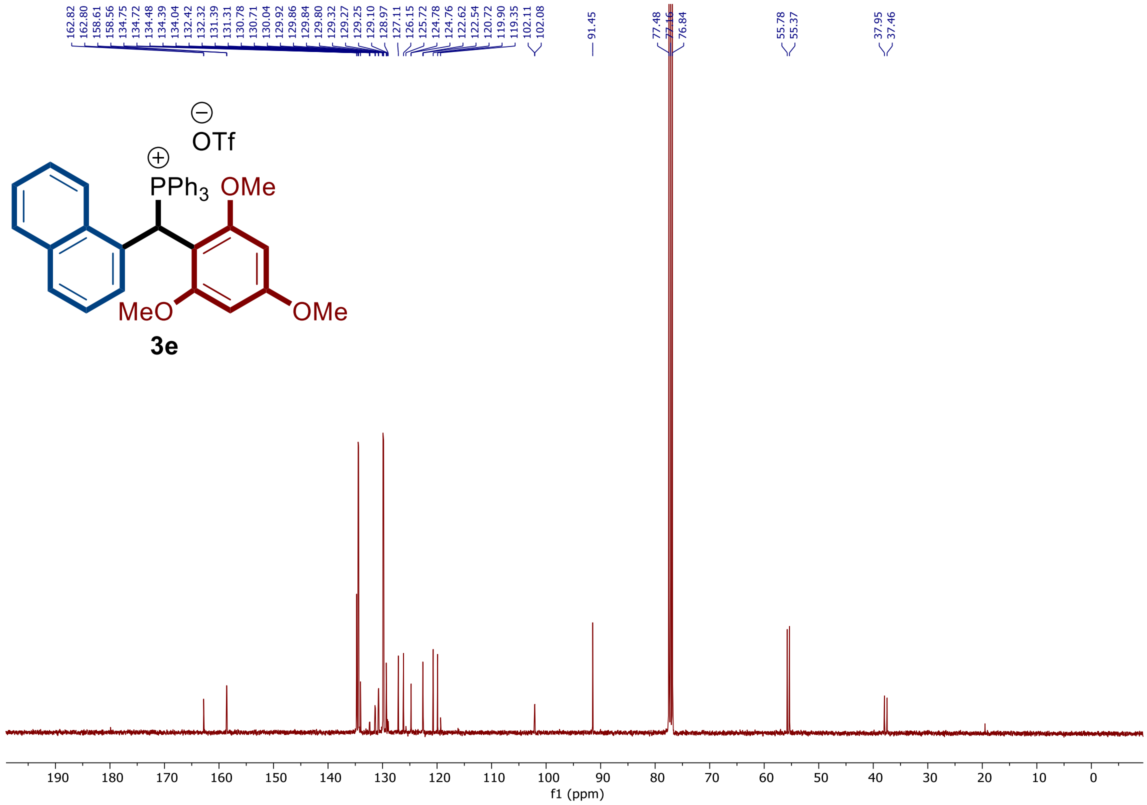

^{31}P NMR (162 MHz, $CDCl_3$) of compound (**3e**)

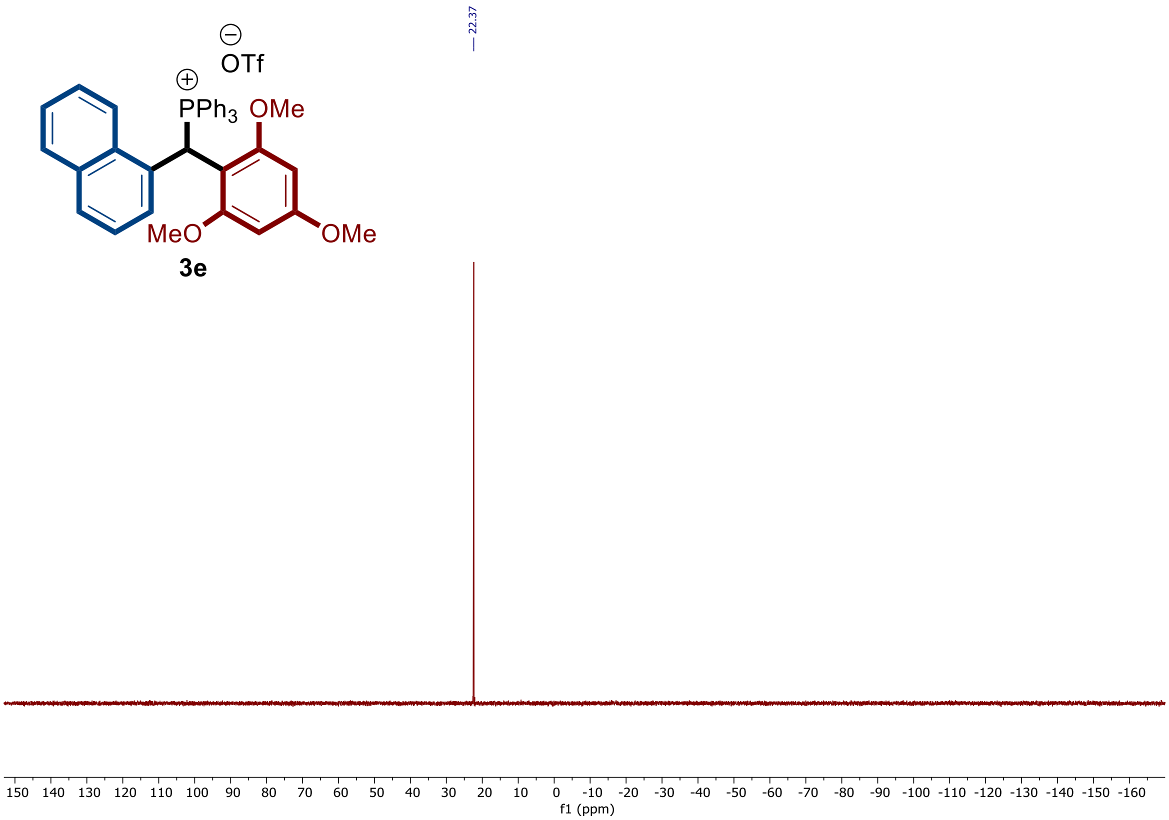

^{19}F NMR (376 MHz, $CDCl_3$) of compound (**3e**)

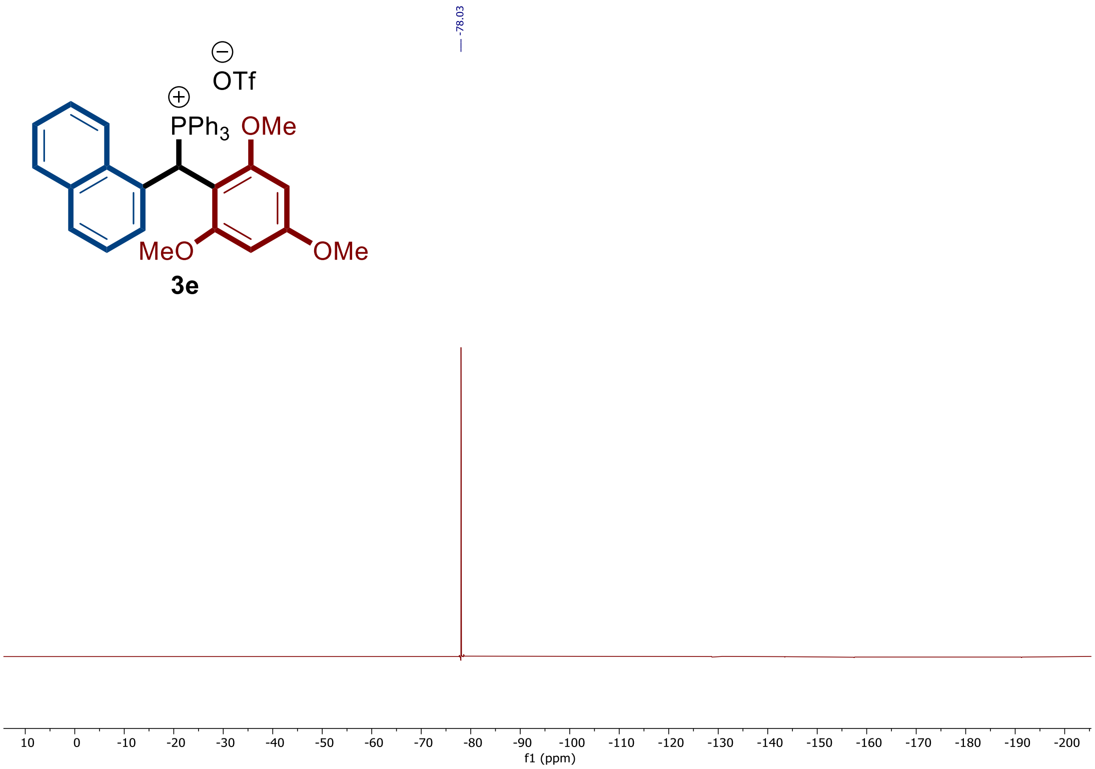

^{1}H NMR (400 MHz, $CDCl_3$) of compound (**3f**)

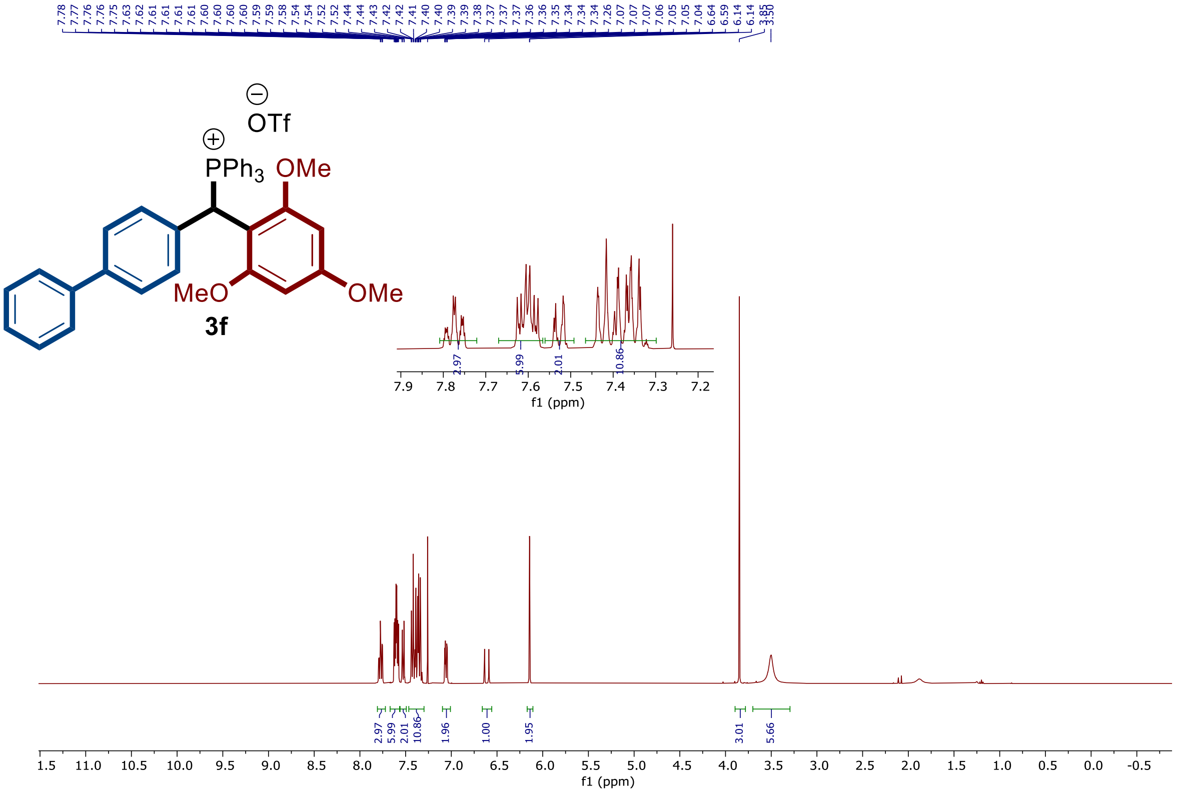

^{13}C{^{1}H} NMR (101 MHz, $CDCl_3$) of compound (**3f**)

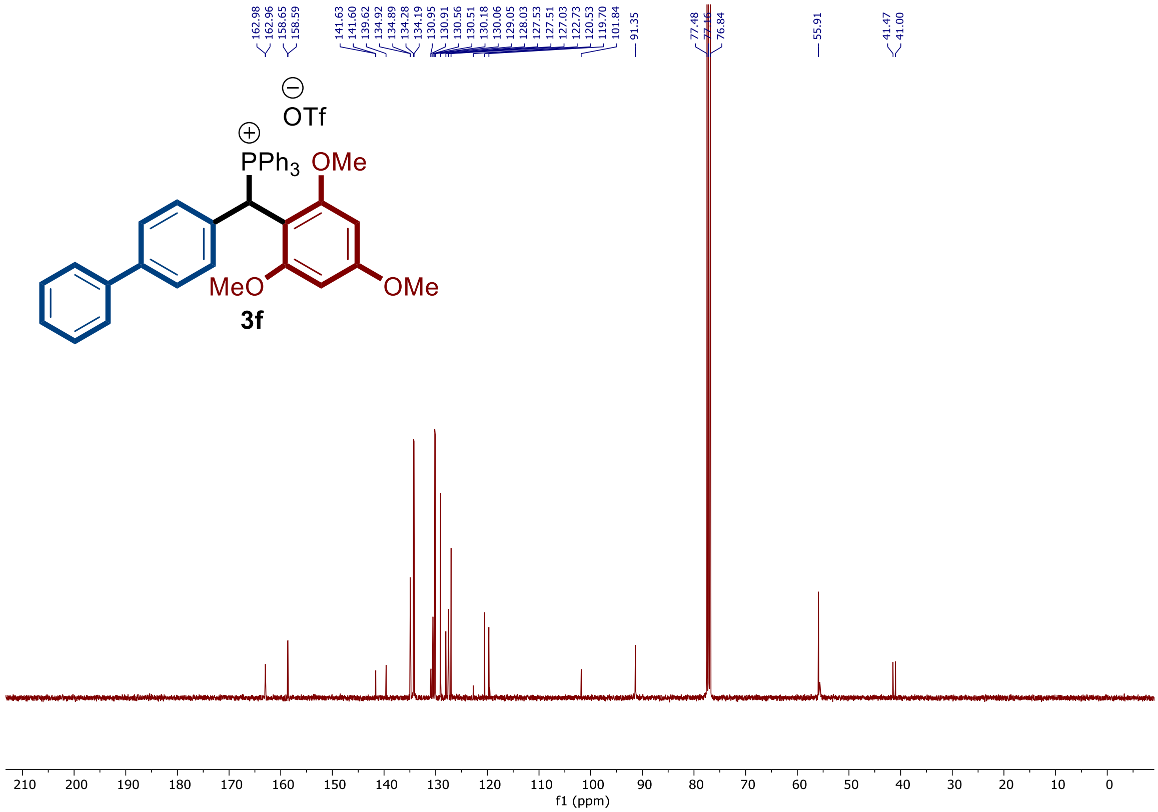

^{31}P NMR (162 MHz, $CDCl_3$) of compound (**3f**)

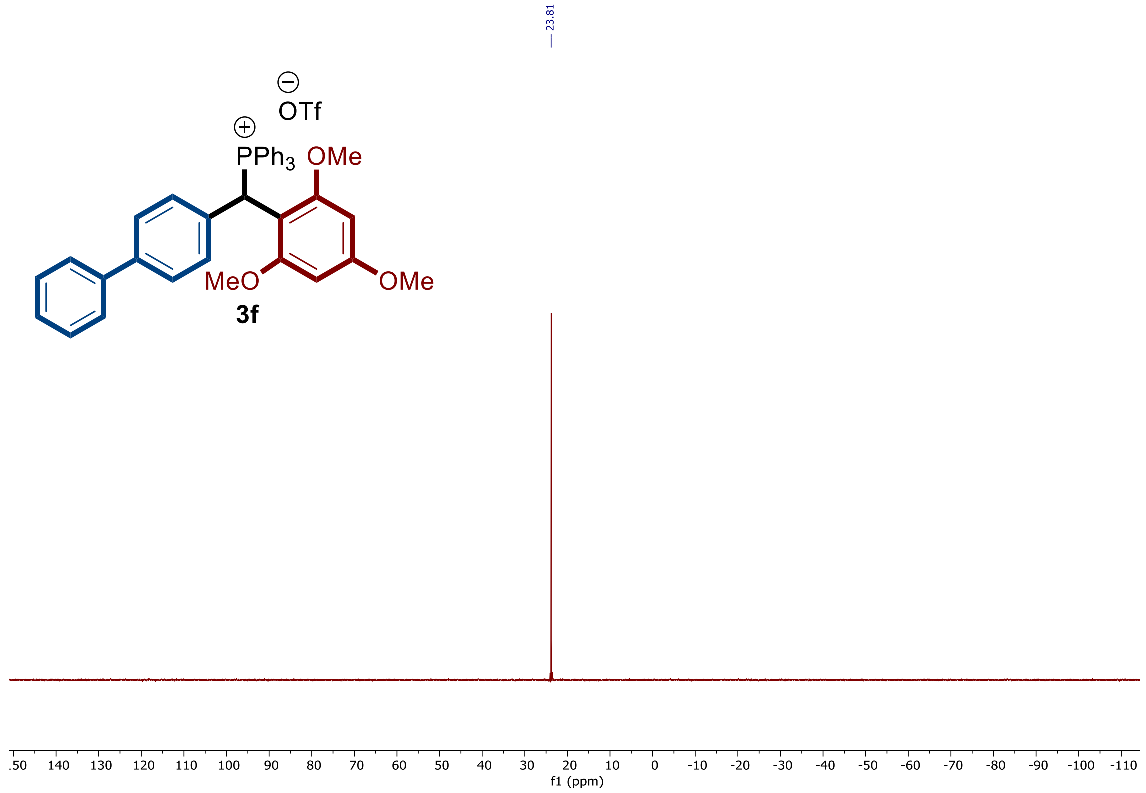

^{19}F NMR (376 MHz, $CDCl_3$) of compound (**3f**)

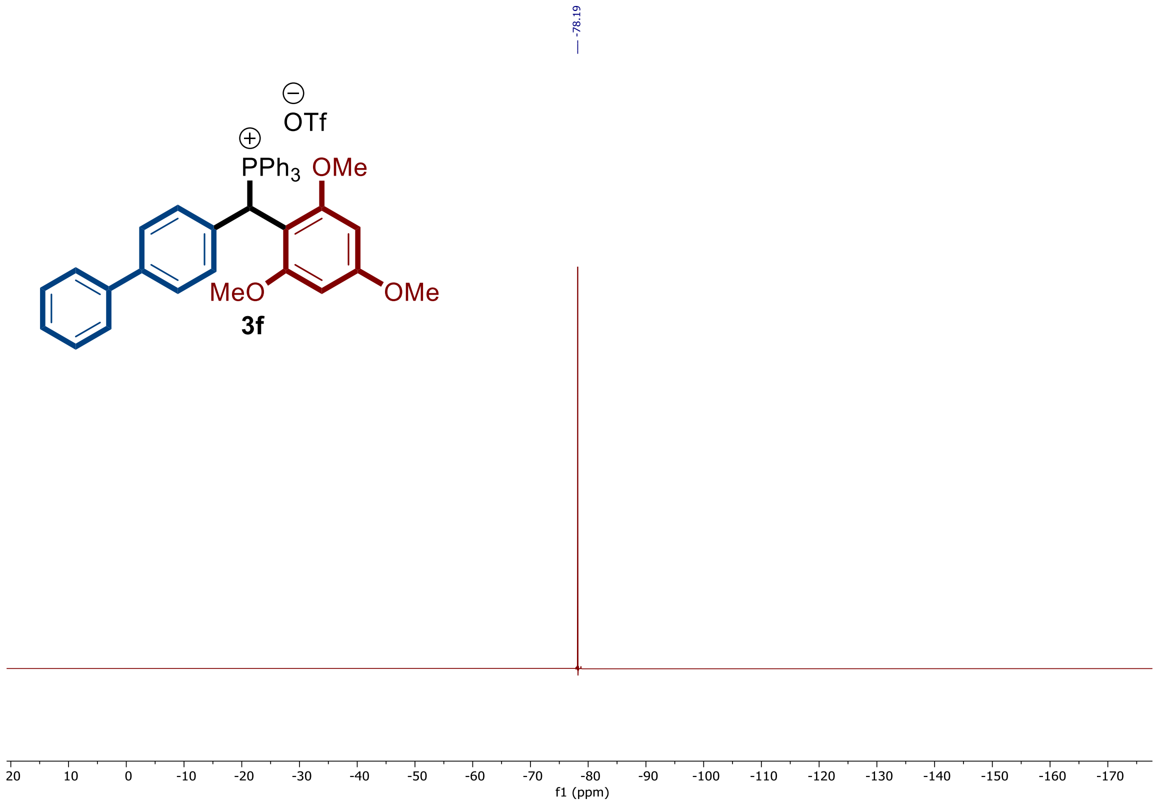

^{1}H NMR (400 MHz, $CDCl_3$) of compound (**3g**)

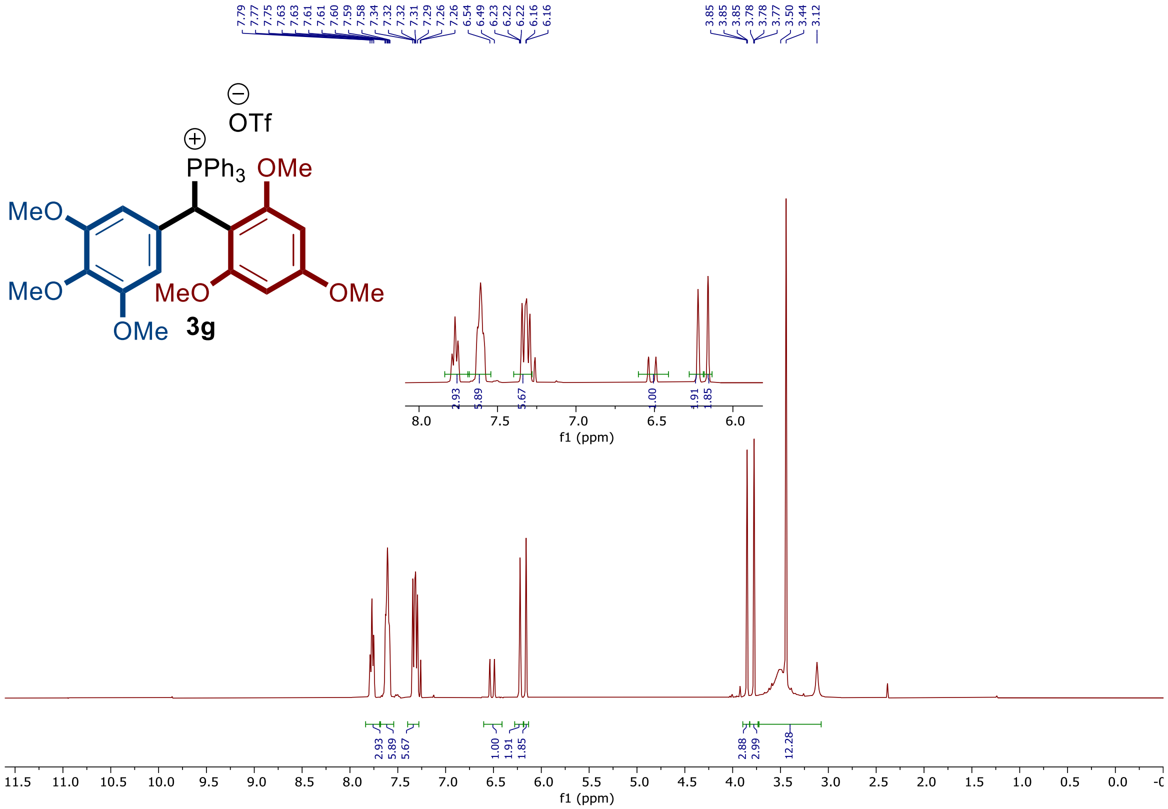

$^{13}C\{^{1}H\}$ NMR (101 MHz, $CDCl_3$) of compound (**3g**)

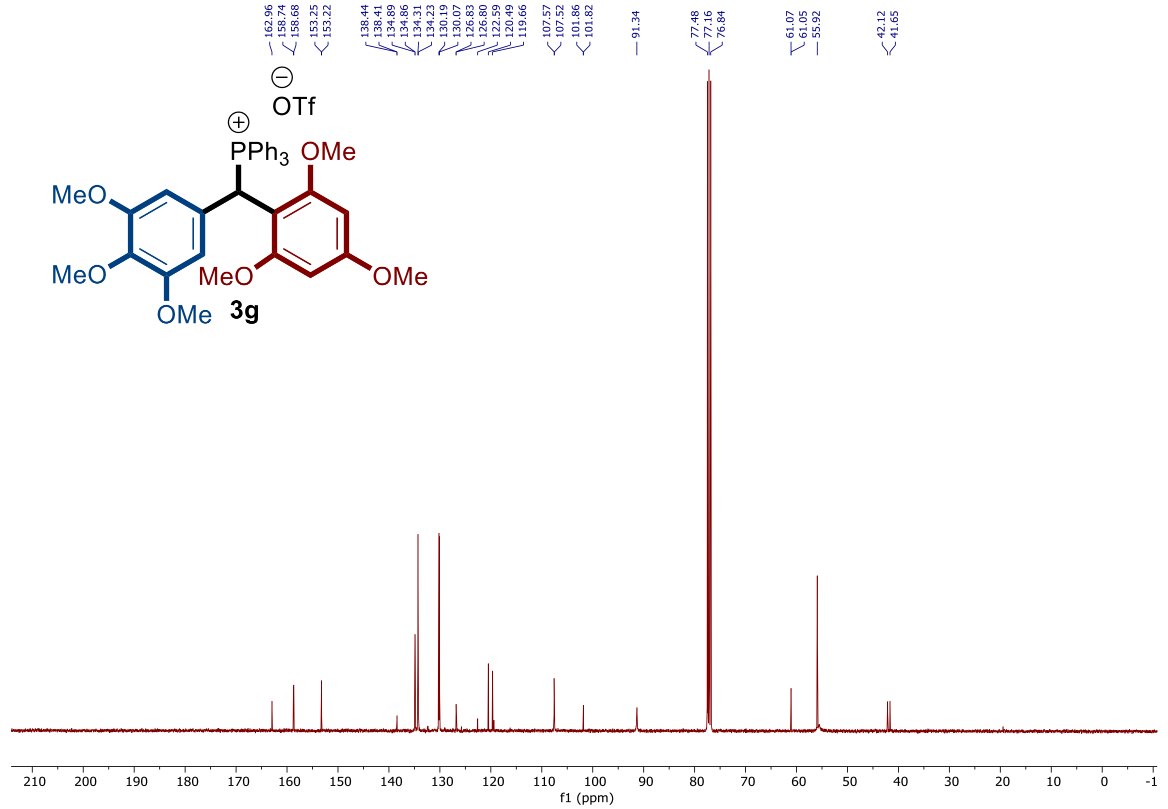

^{31}P NMR (162 MHz, $CDCl_3$) of compound (**3g**)

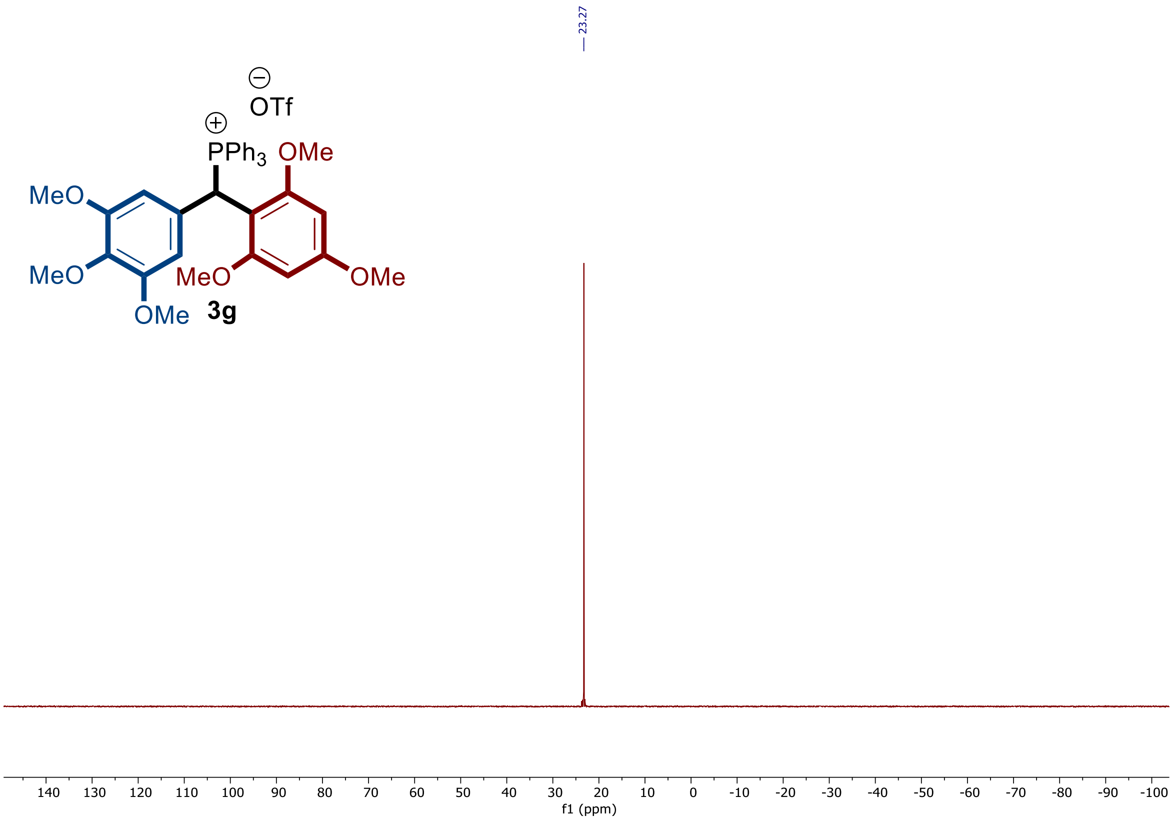

^{19}F NMR (376 MHz, $CDCl_3$) of compound (**3g**)

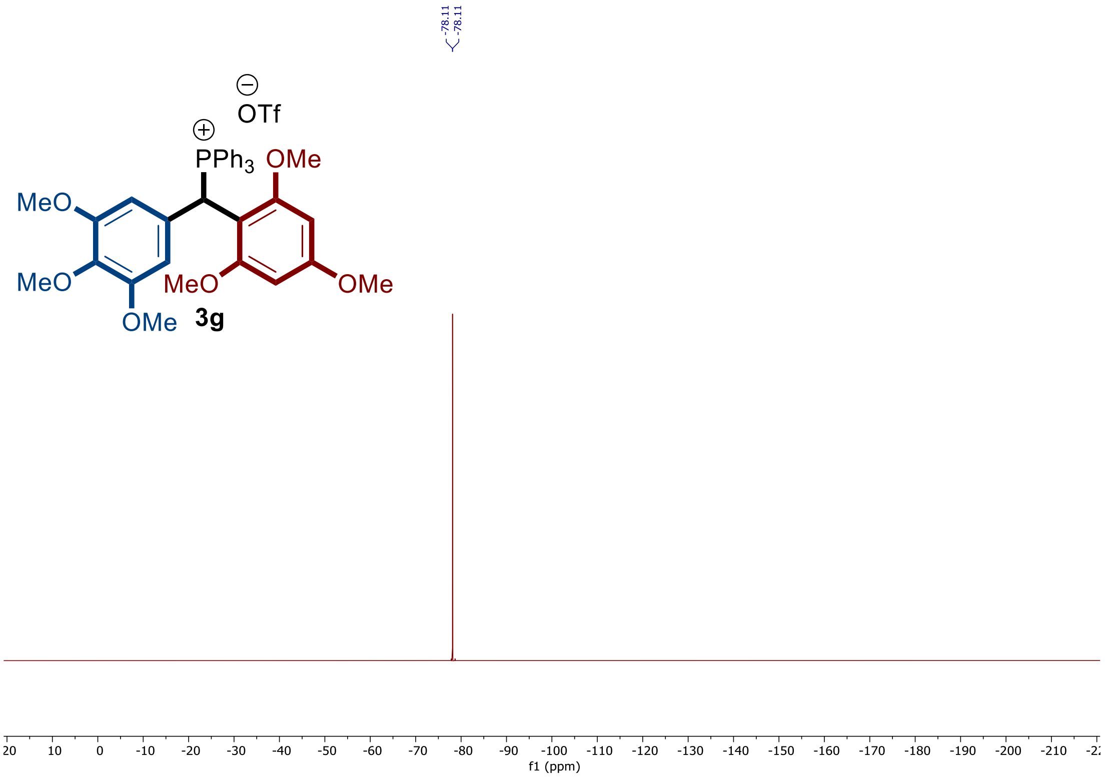

^{1}H NMR (400 MHz, $CDCl_3$) of compound (**3h**)

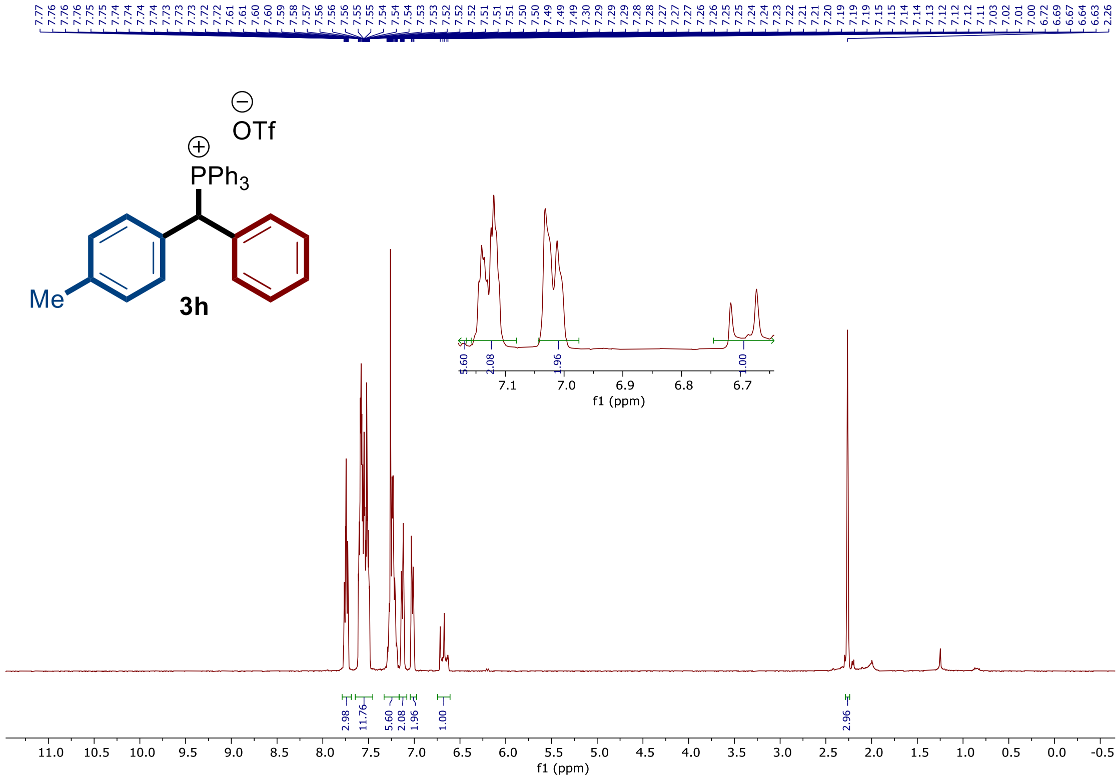

^{13}C{^{1}H} NMR (101 MHz, $CDCl_3$) of compound (**3h**)

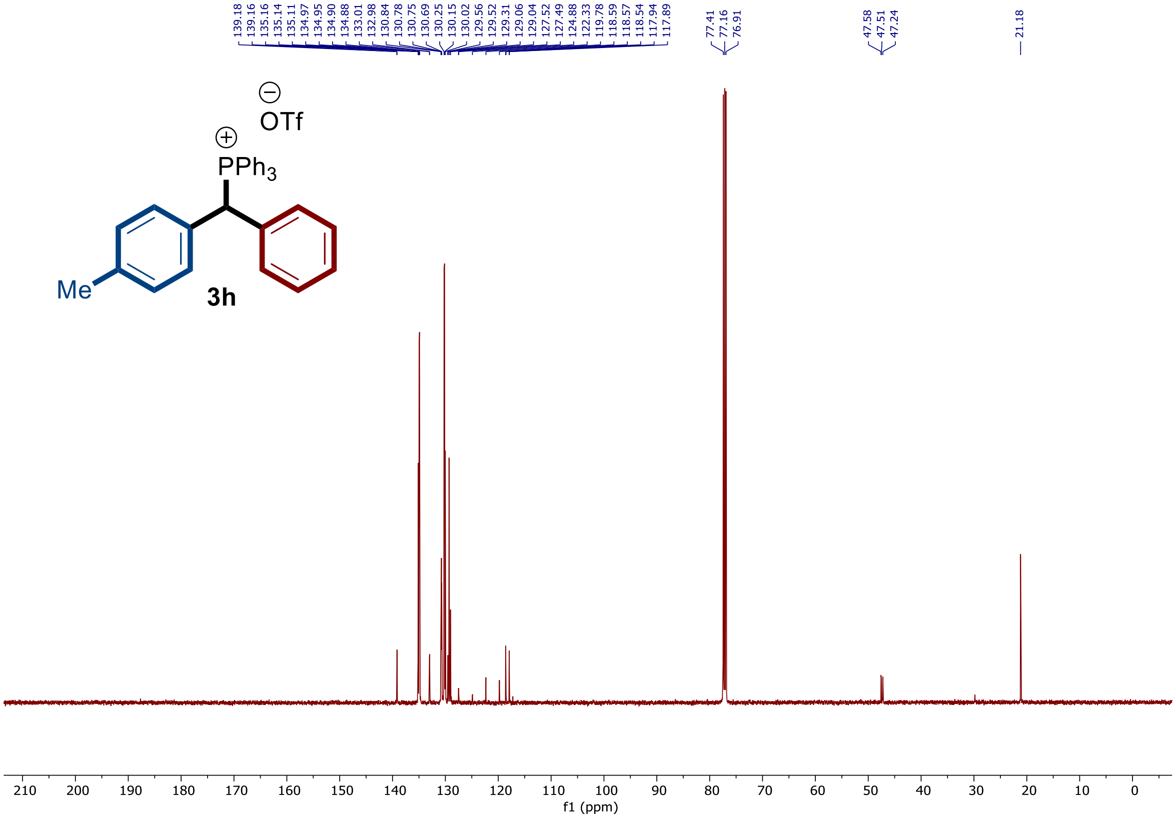

^{31}P NMR (162 MHz, $CDCl_3$) of compound (**3h**)

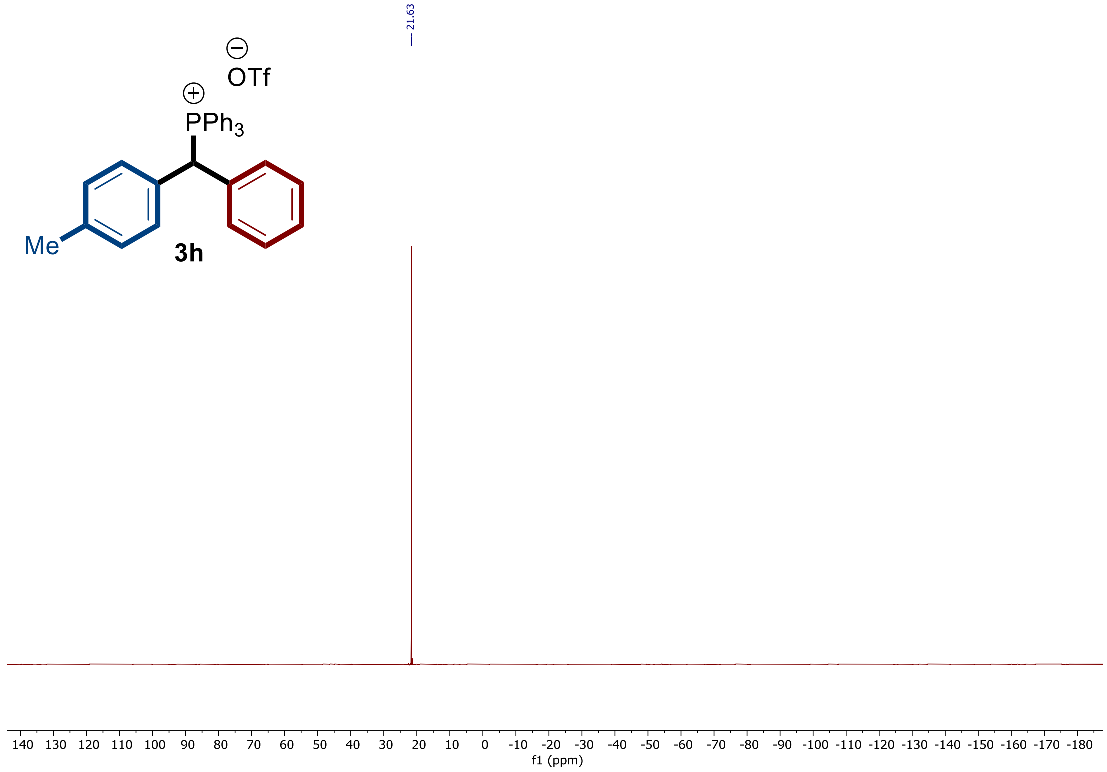

^{19}F NMR (376 MHz, $CDCl_3$) of compound (**3h**)

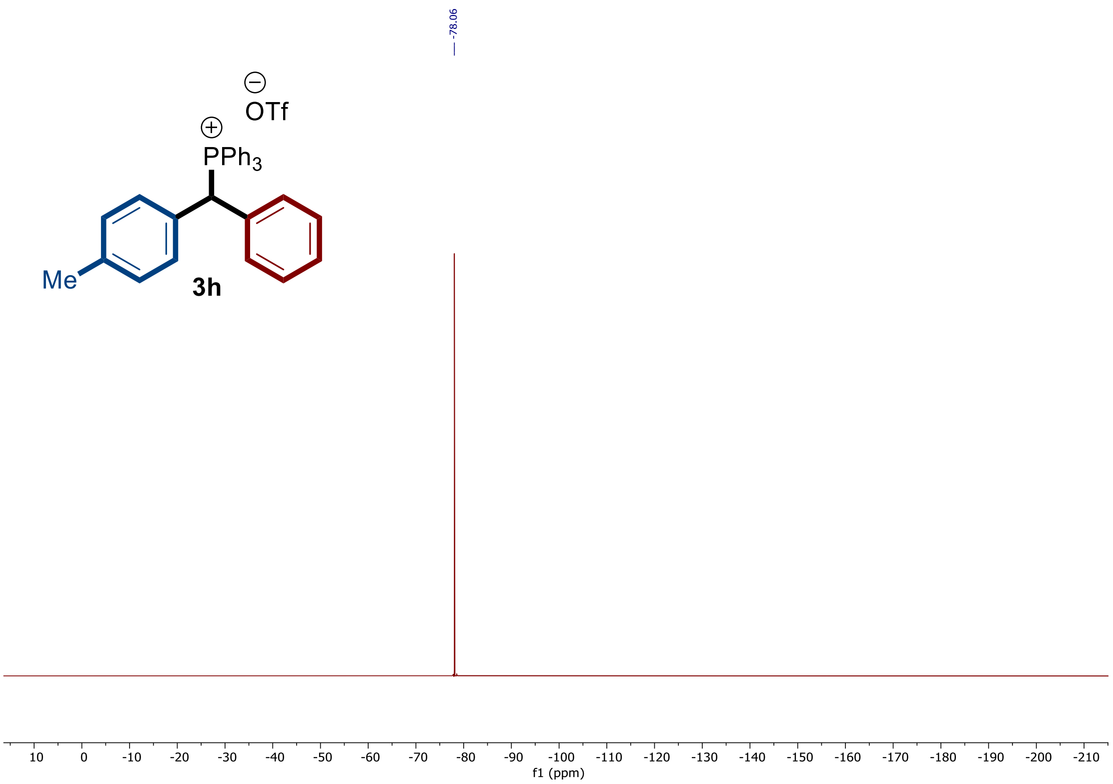

^{1}H NMR (400 MHz, $CDCl_3$) of compound (**3i**)

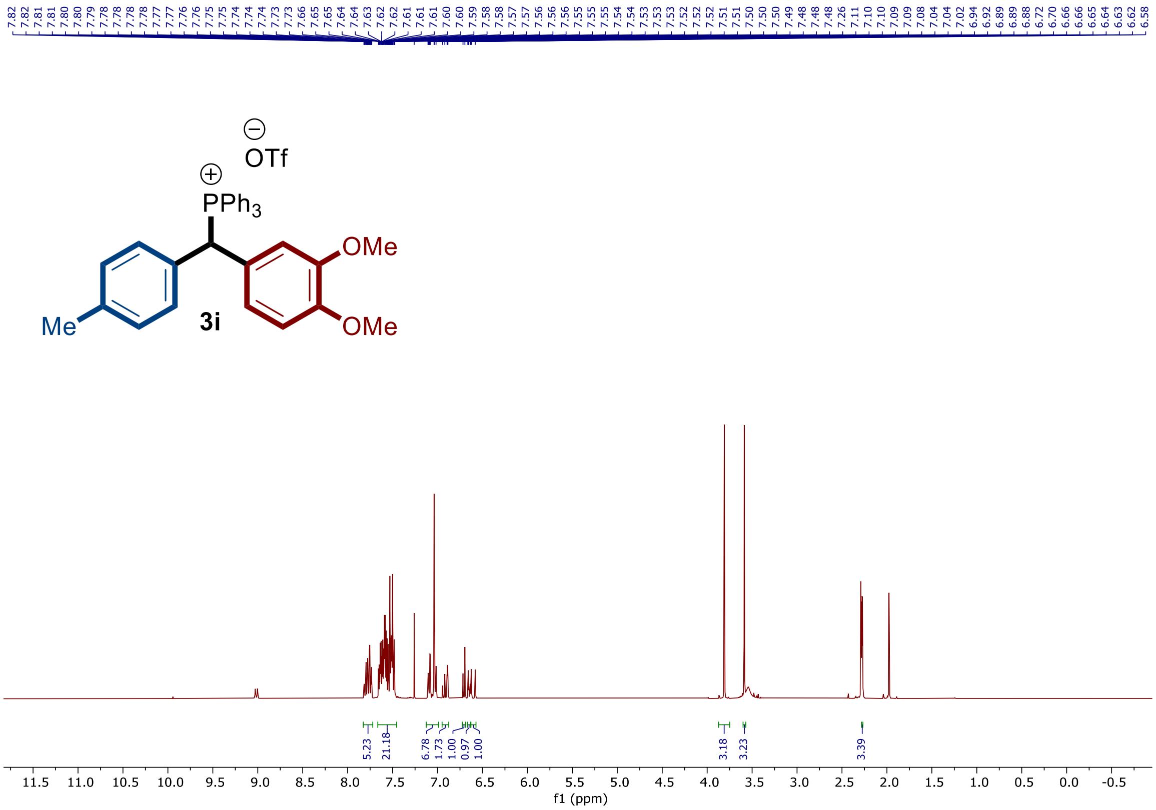

$^{13}C\{^{1}H\}$ NMR (101 MHz, $CDCl_3$) of compound (**3i**)

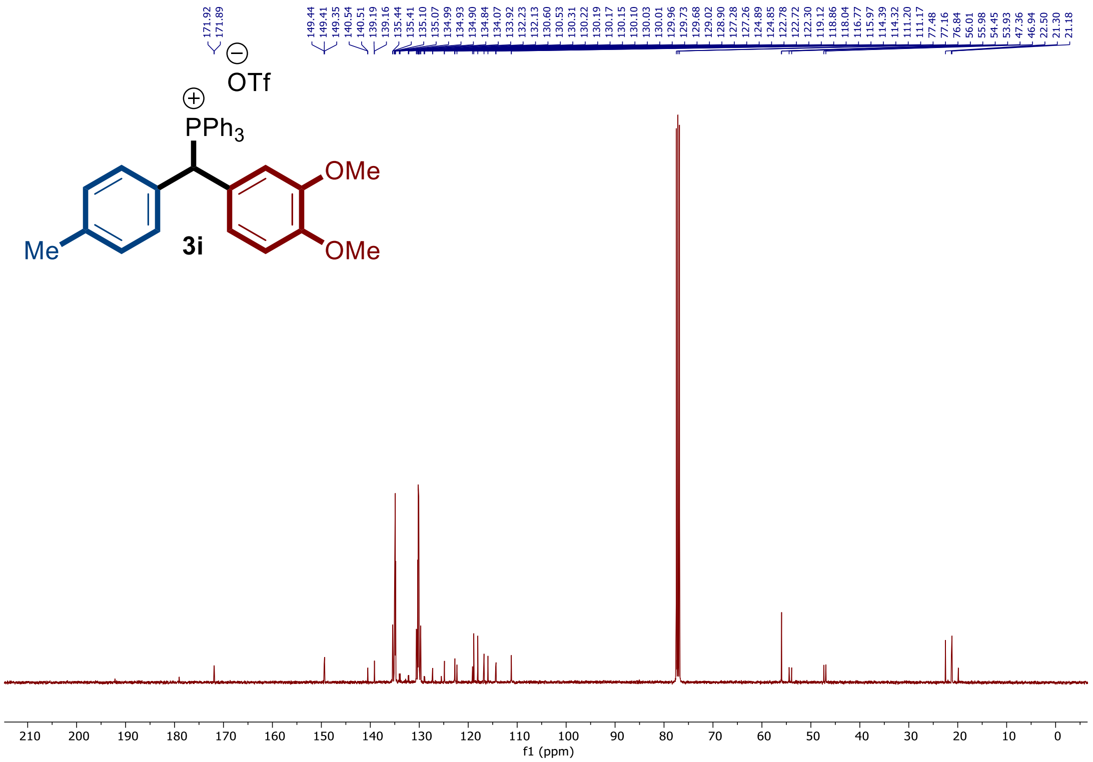

^{31}P NMR (162 MHz, $CDCl_3$) of compound (**3i**)

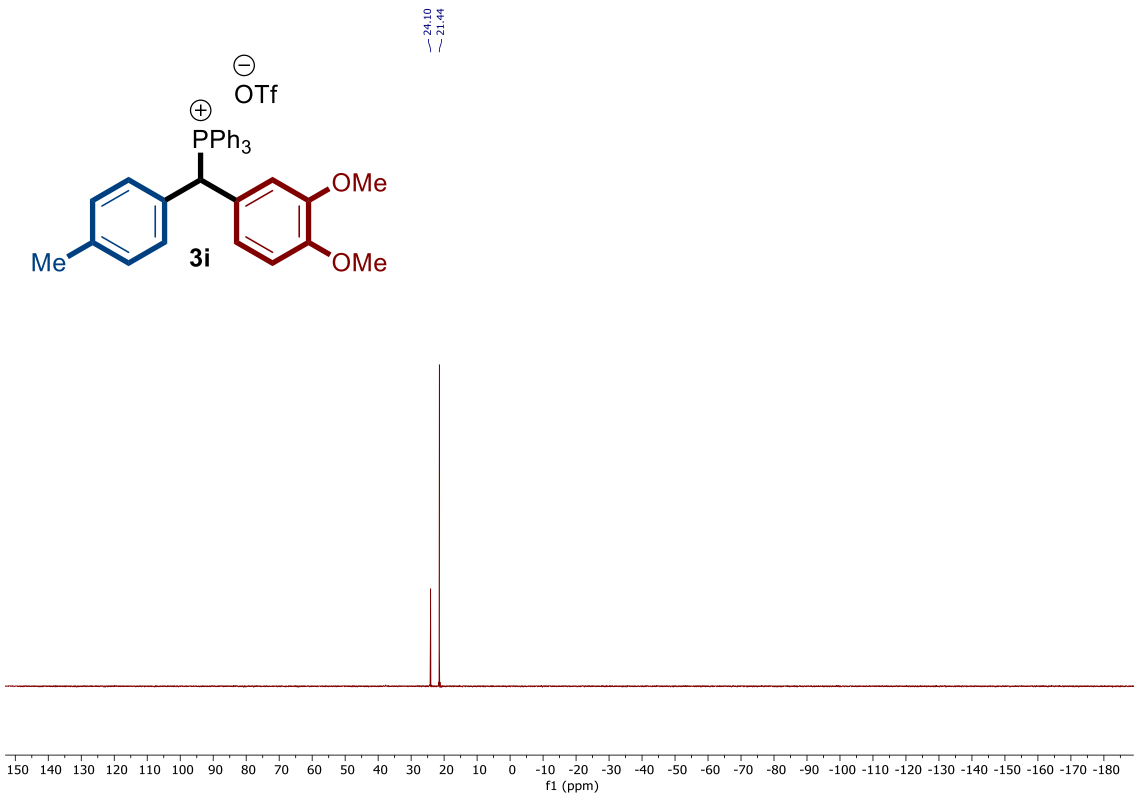

^{19}F NMR (376 MHz, $CDCl_3$) of compound (**3i**)

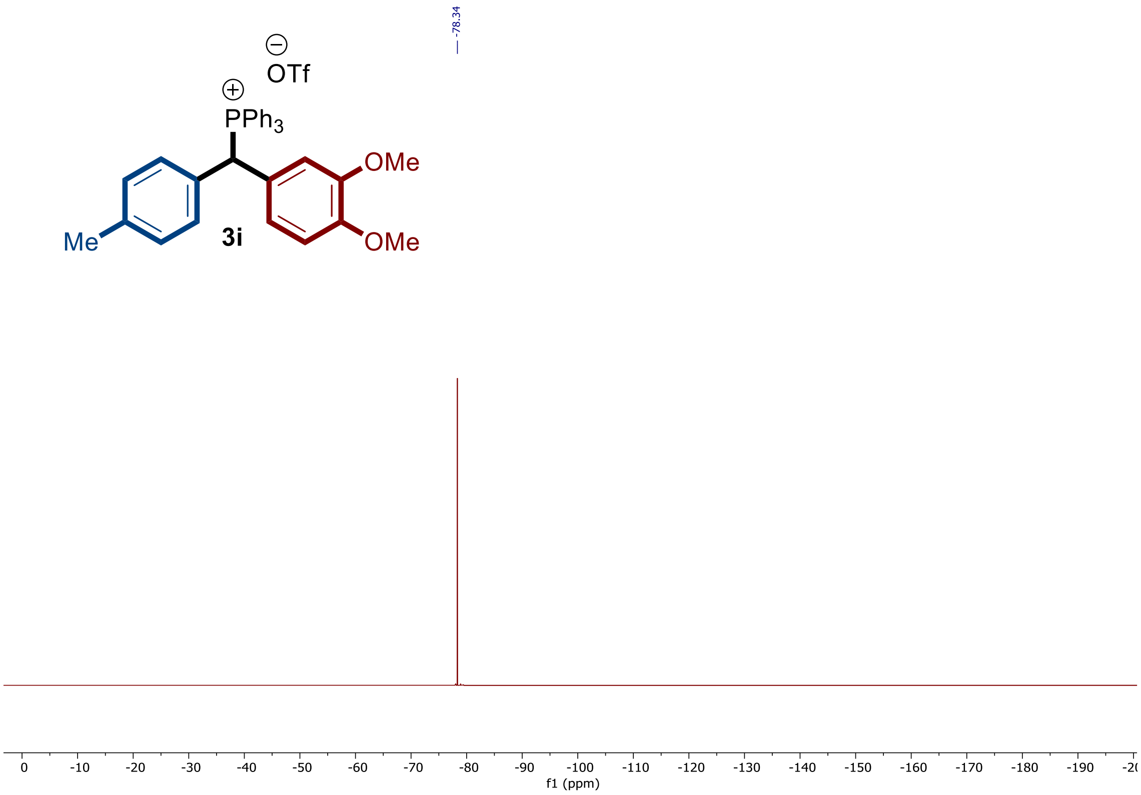

^{1}H NMR (400 MHz, $CDCl_3$) of compound (**3j**)

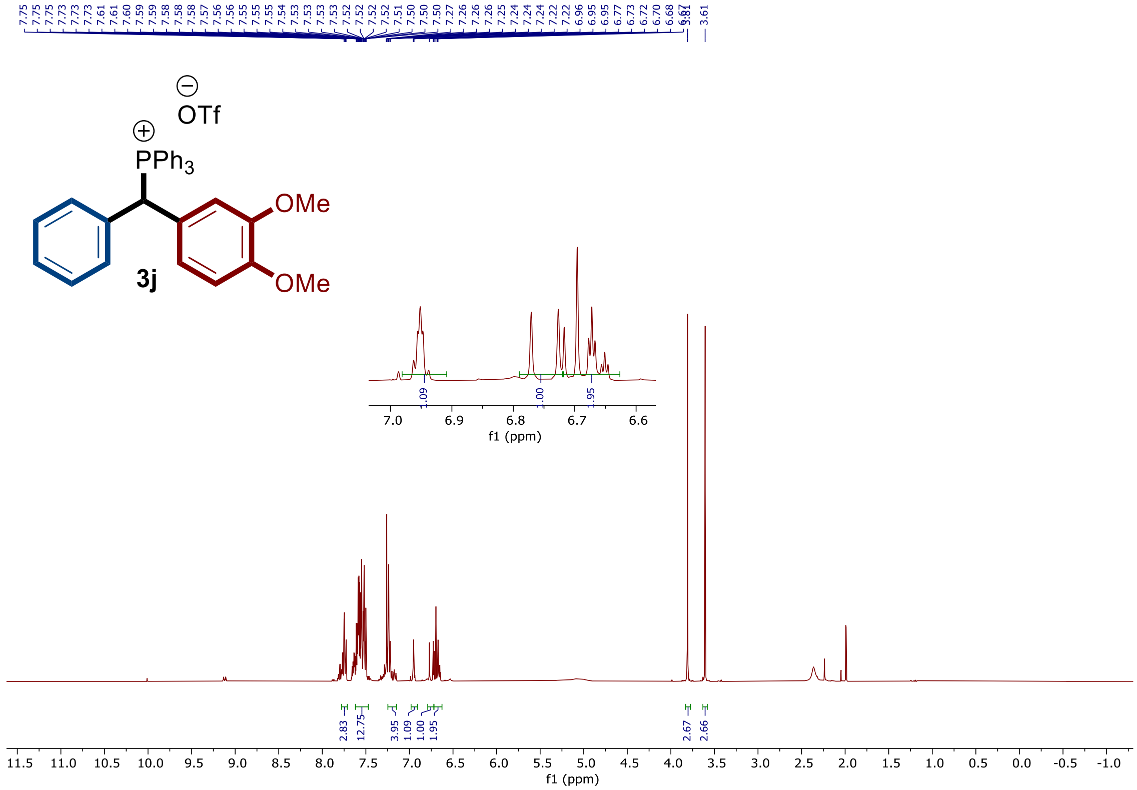

^{13}C{^{1}H} NMR (101 MHz, $CDCl_3$) of compound (**3j**)

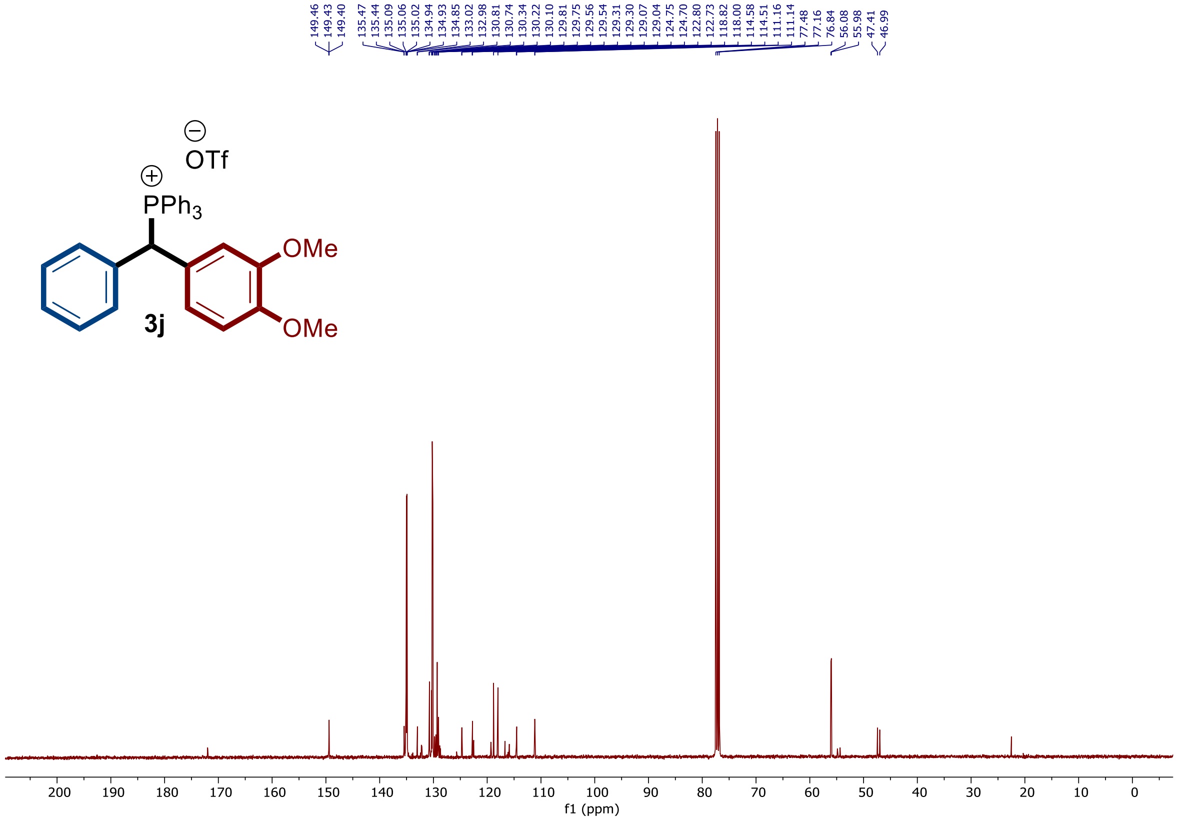

^{31}P NMR (162 MHz, CDCl$_3$) of compound (**3j**)

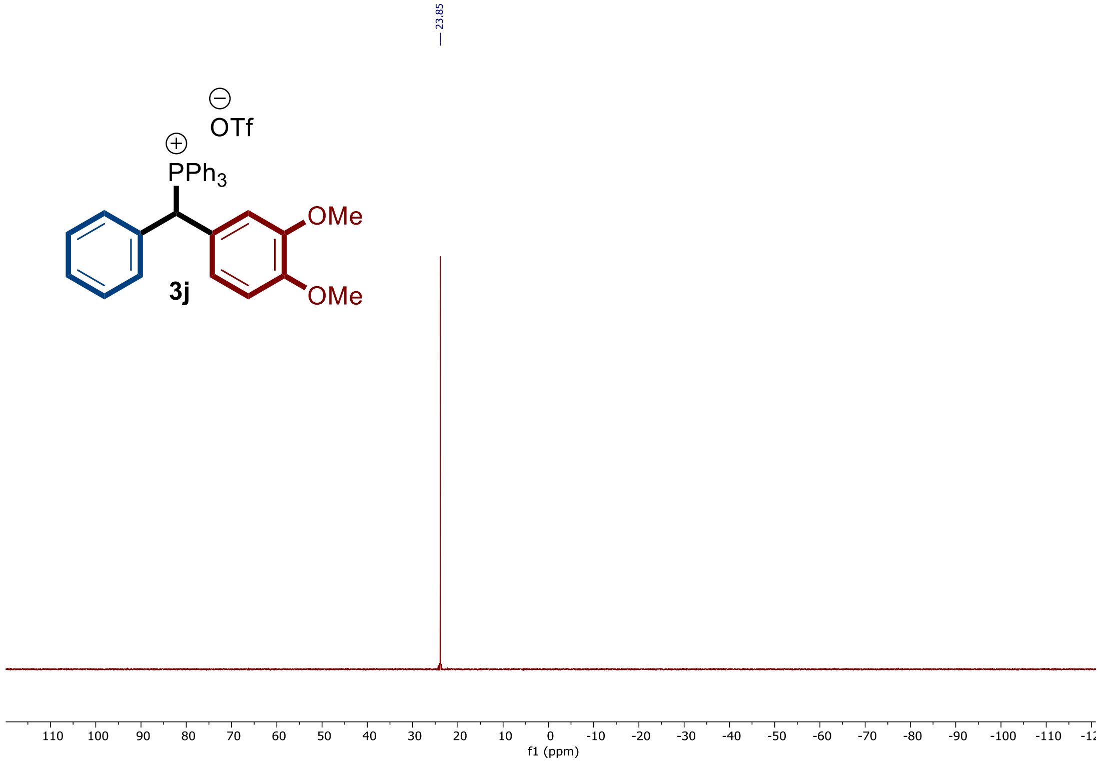

^{19}F NMR (376 MHz, CDCl$_3$) of compound (**3j**)

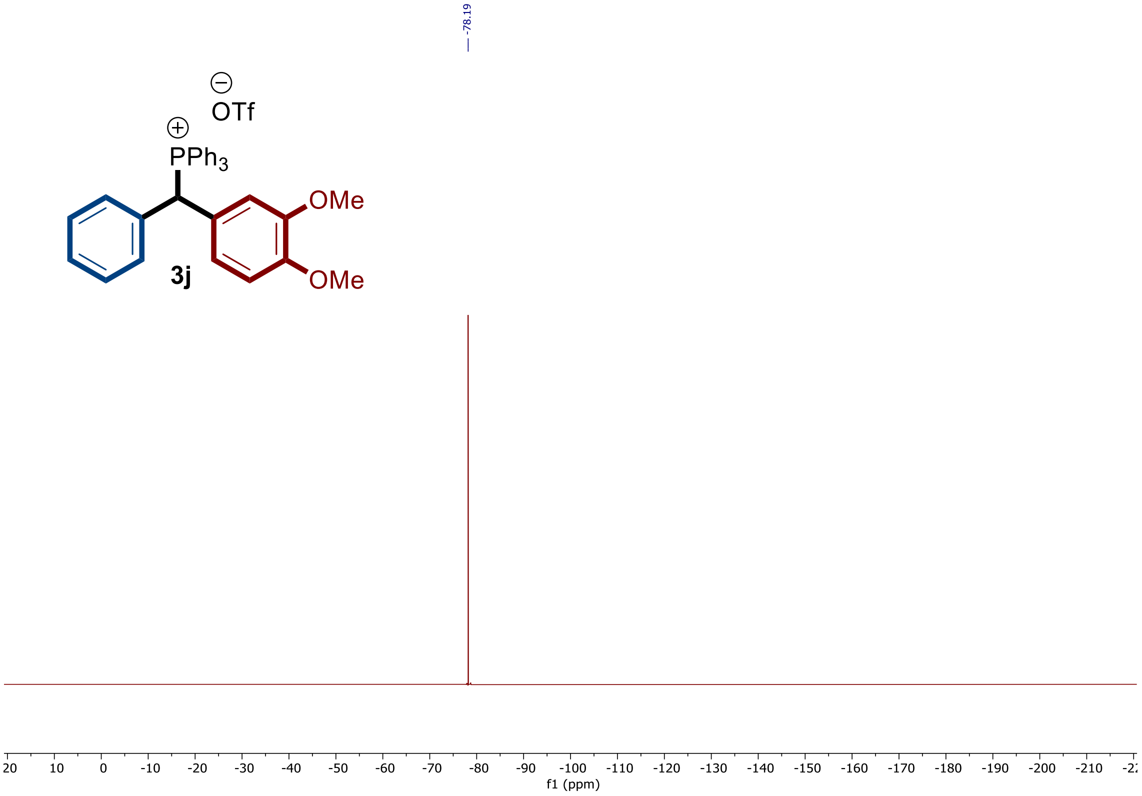

^{1}H NMR (400 MHz, $CDCl_3$) of compound (**3k**)

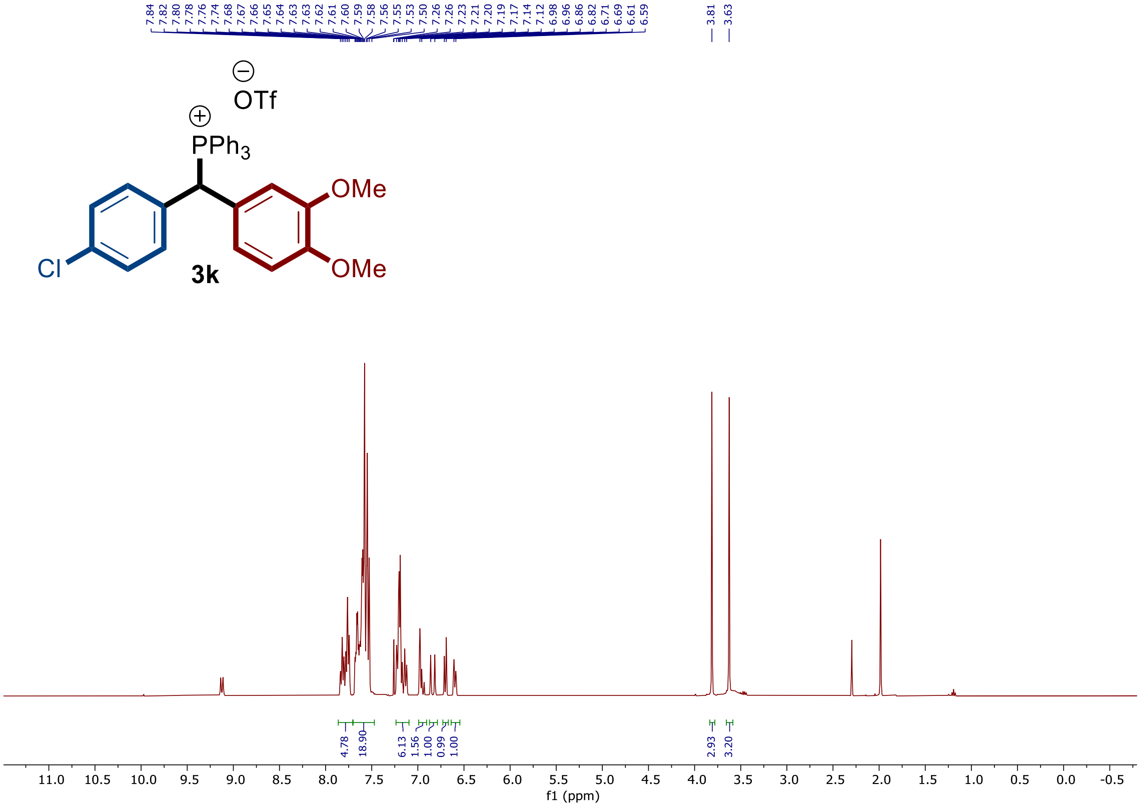

^{13}C{^{1}H} NMR (101 MHz, $CDCl_3$) of compound (**3k**)

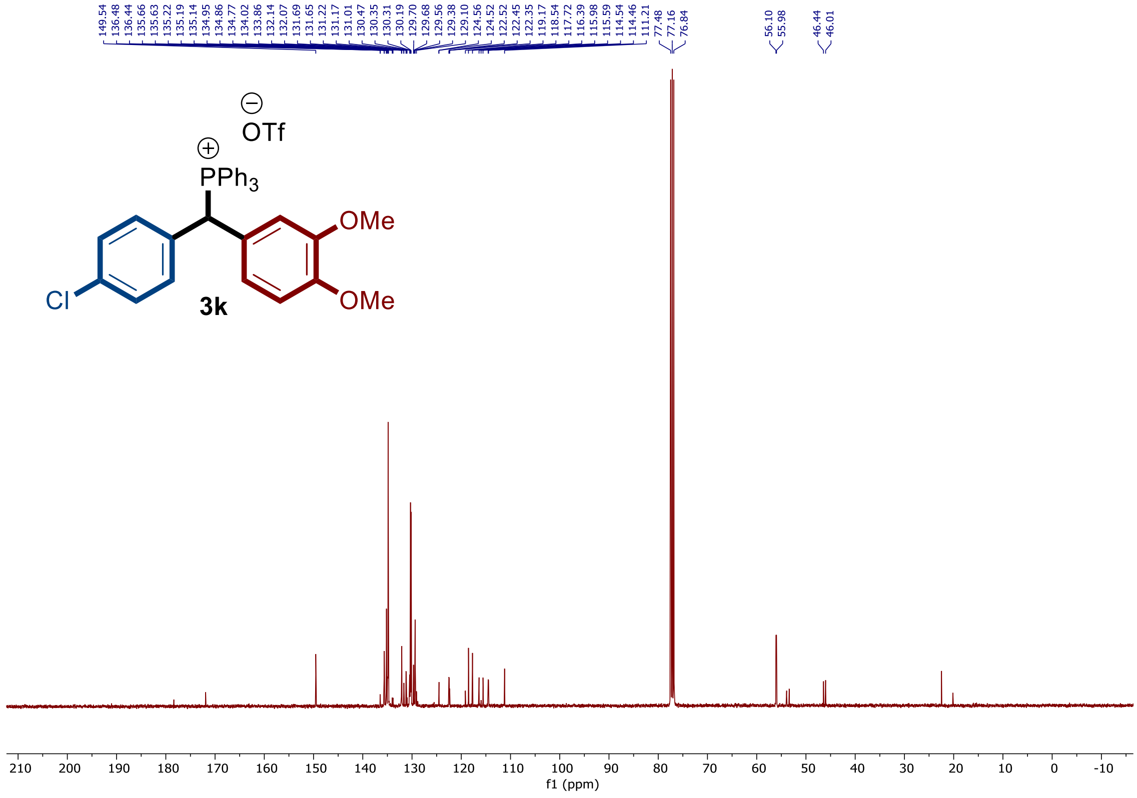

^{31}P NMR (162 MHz, $CDCl_3$) of compound (**3k**)

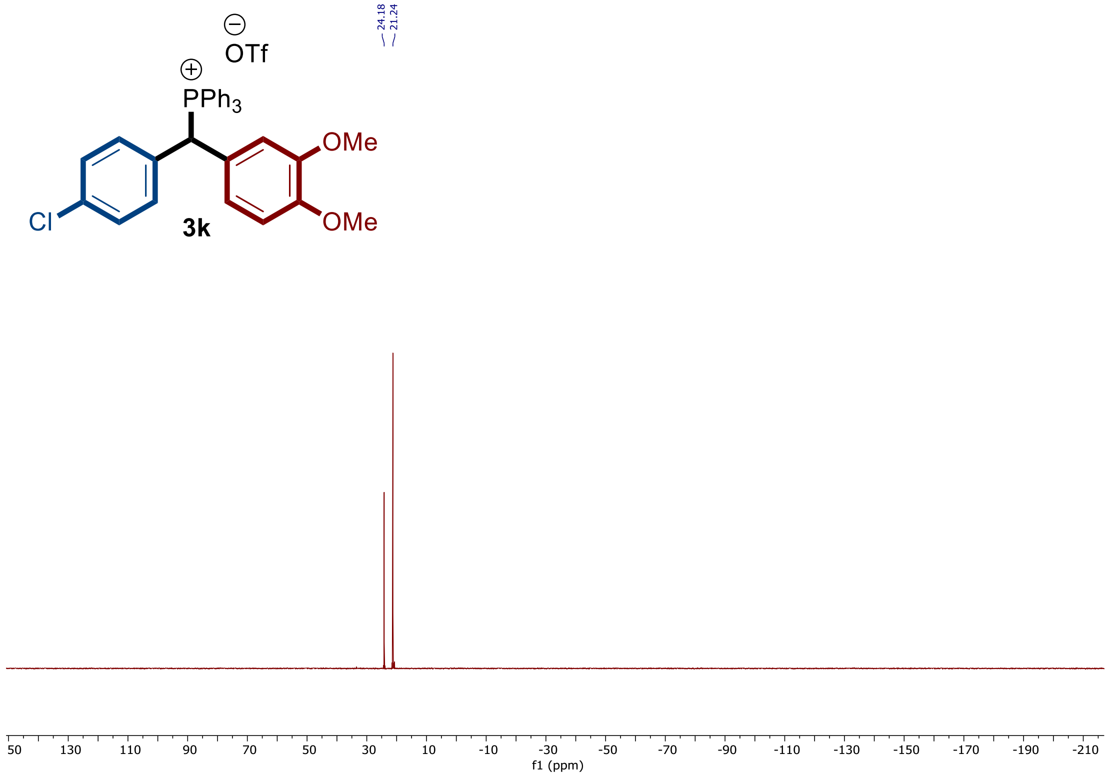

^{19}F NMR (376 MHz, $CDCl_3$) of compound (**3k**)

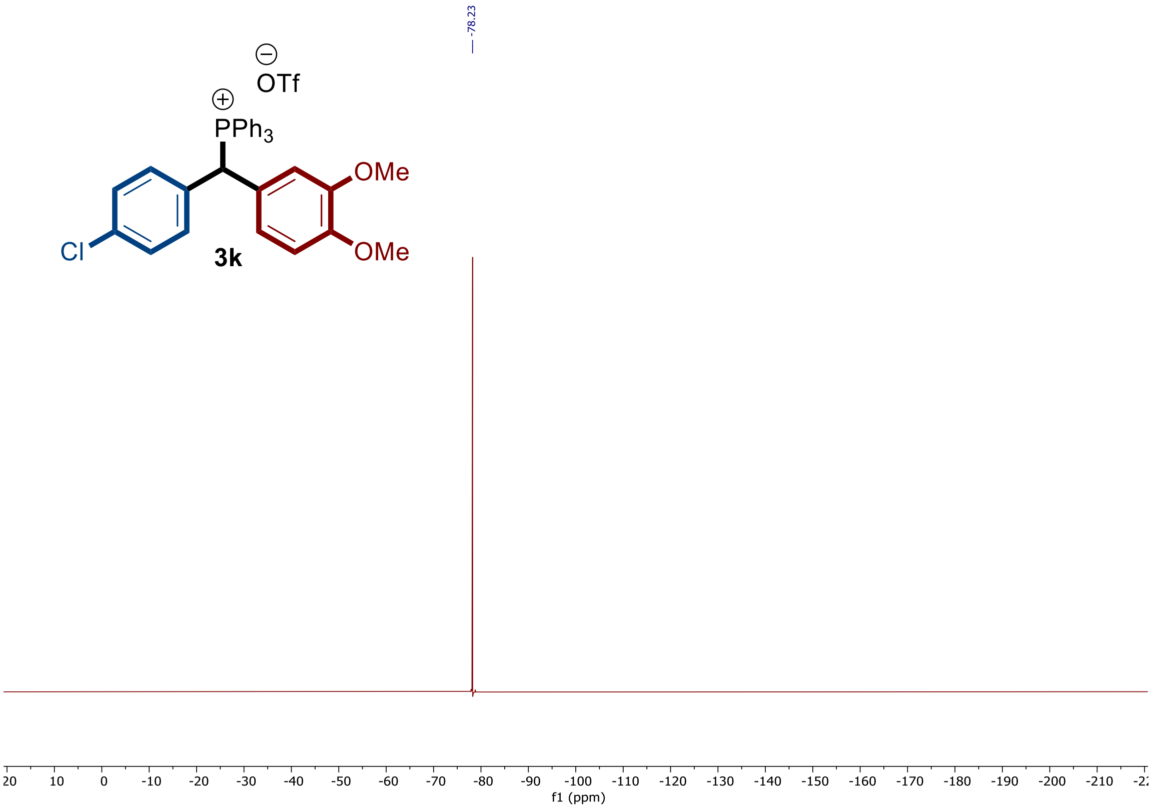

^{1}H NMR (400 MHz, $CDCl_3$) of compound (**3l**)

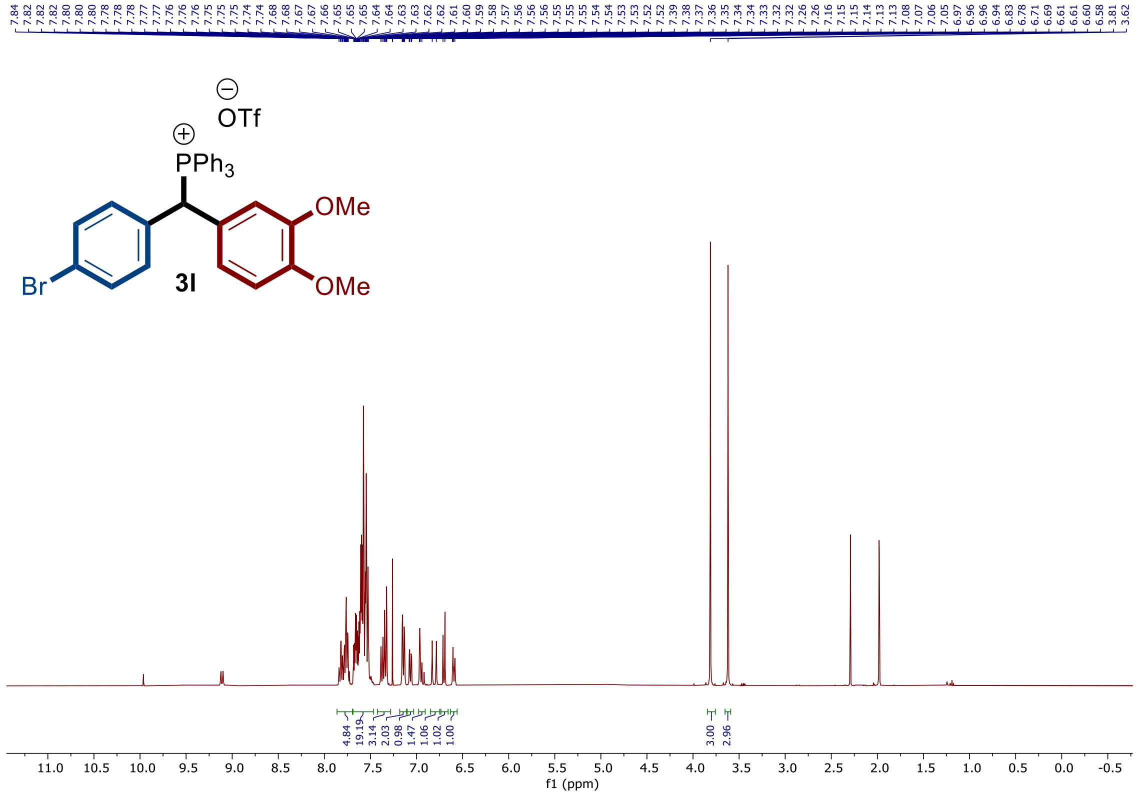

$^{13}C\{^{1}H\}$ NMR (101 MHz, $CDCl_3$) of compound (**3l**)

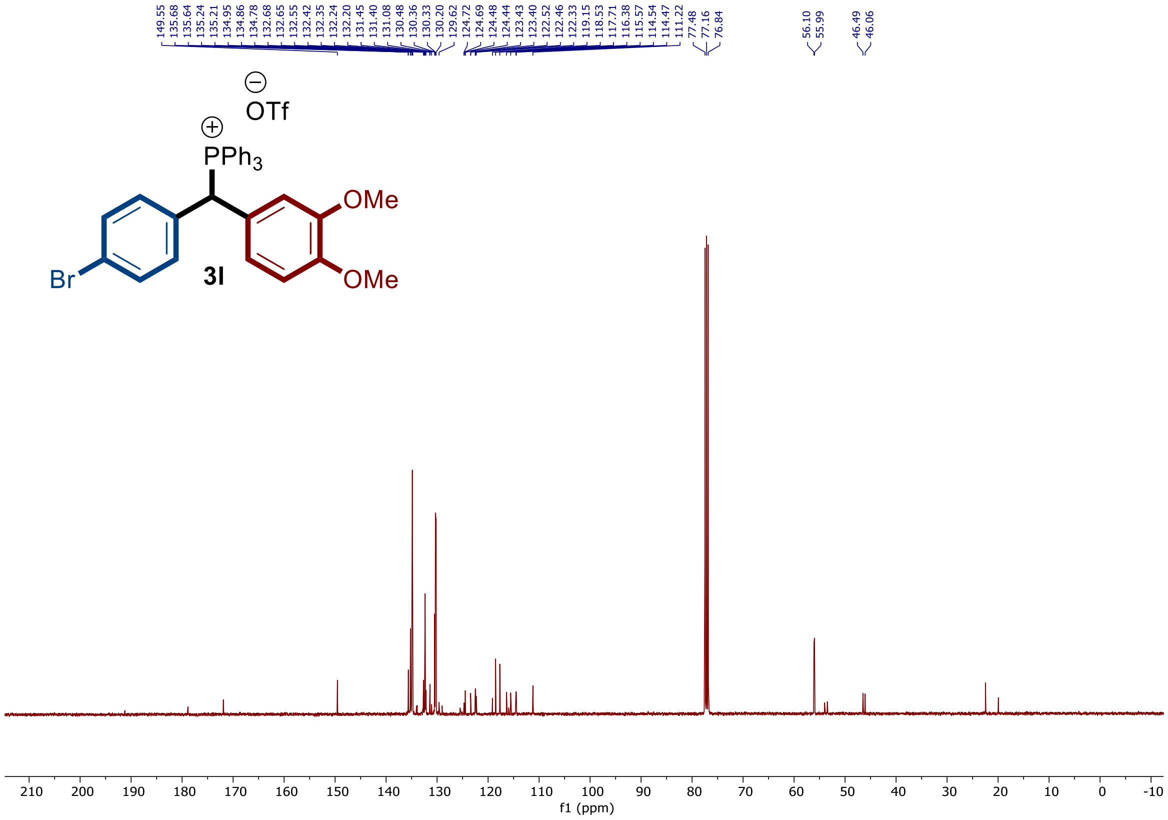

^{31}P NMR (162 MHz, $CDCl_3$) of compound (**3l**)

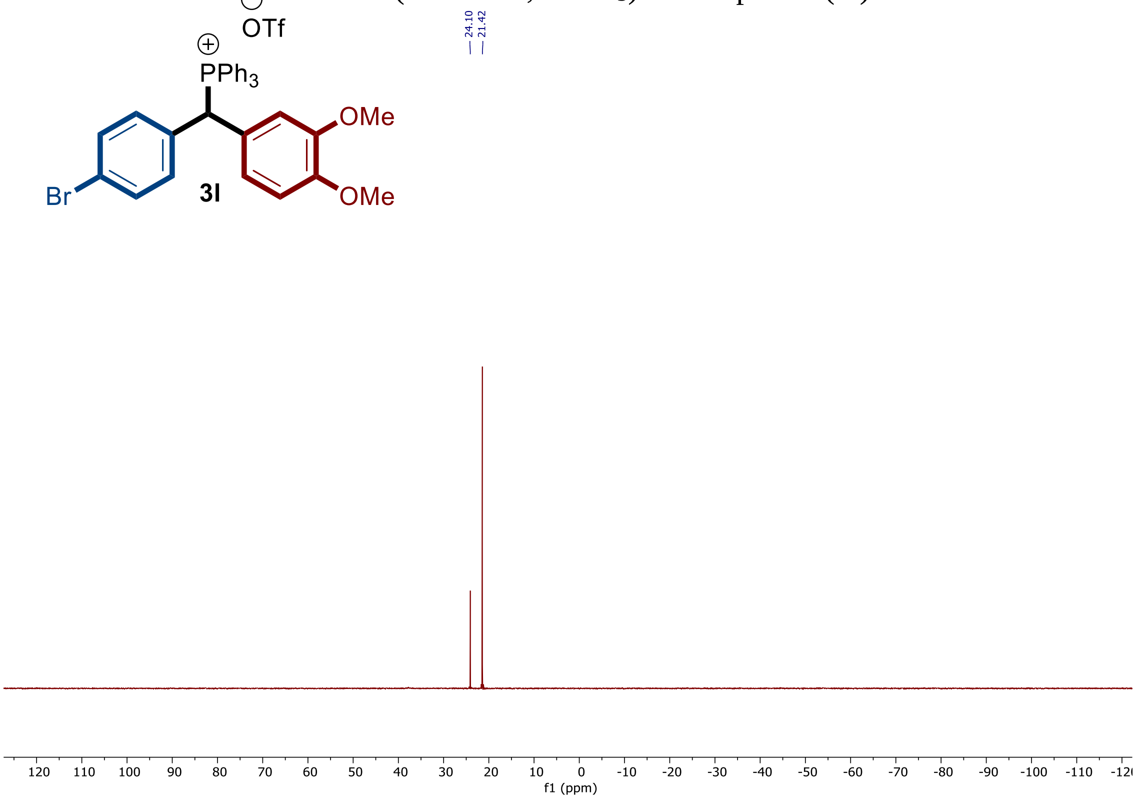

^{19}F NMR (376 MHz, $CDCl_3$) of compound (**3l**)

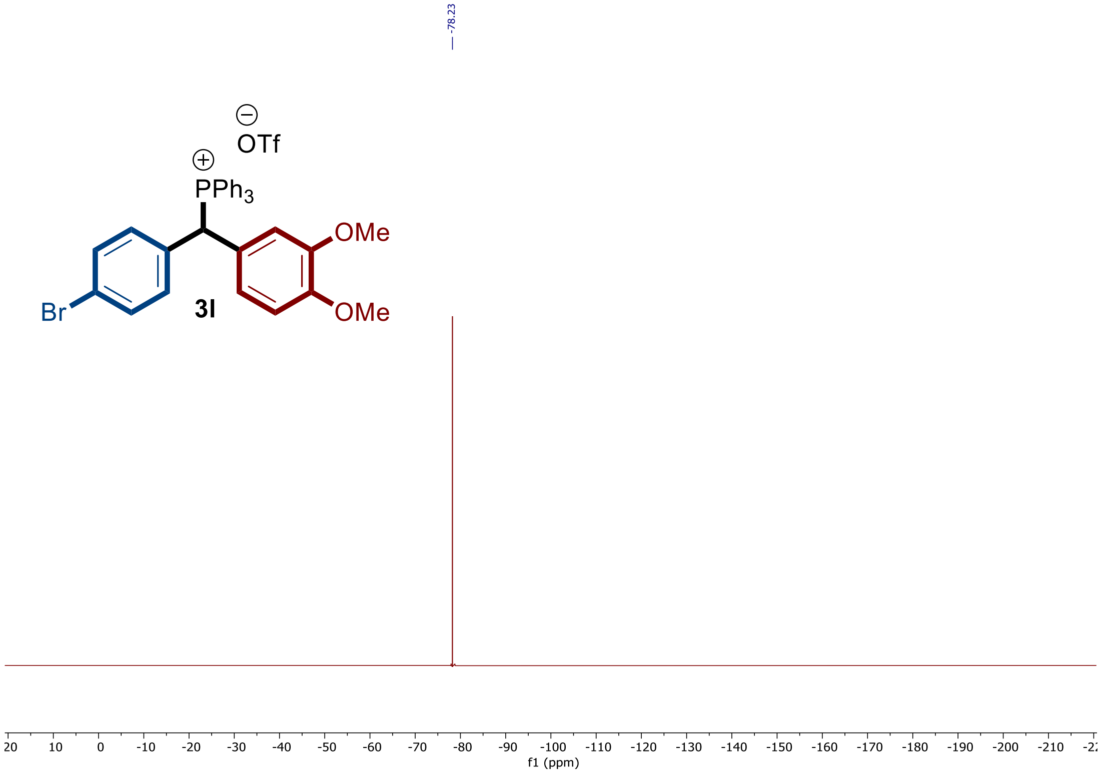

^{1}H NMR (400 MHz, $CDCl_3$) of compound (**3ah**)

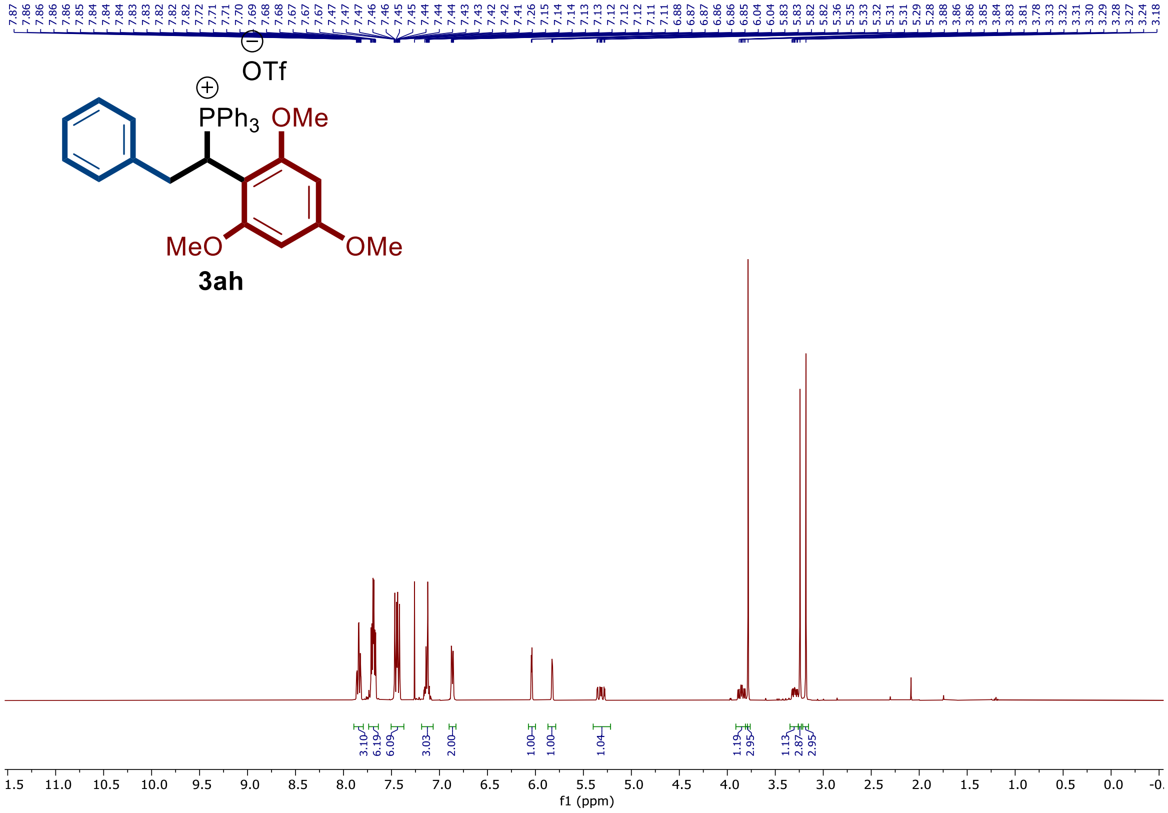

$^{13}C\{^{1}H\}$ NMR (101 MHz, $CDCl_3$) of compound (**3ah**)

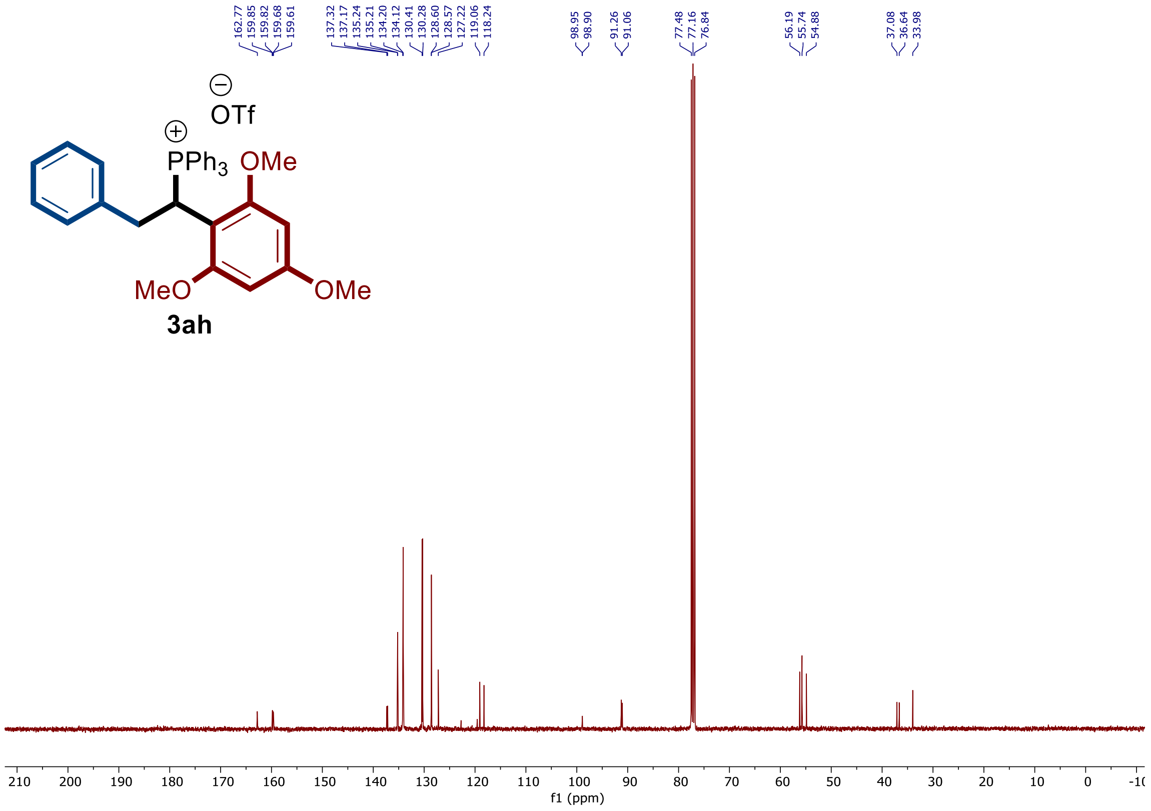

^{31}P NMR (162 MHz, $CDCl_3$) of compound (**3ah**)

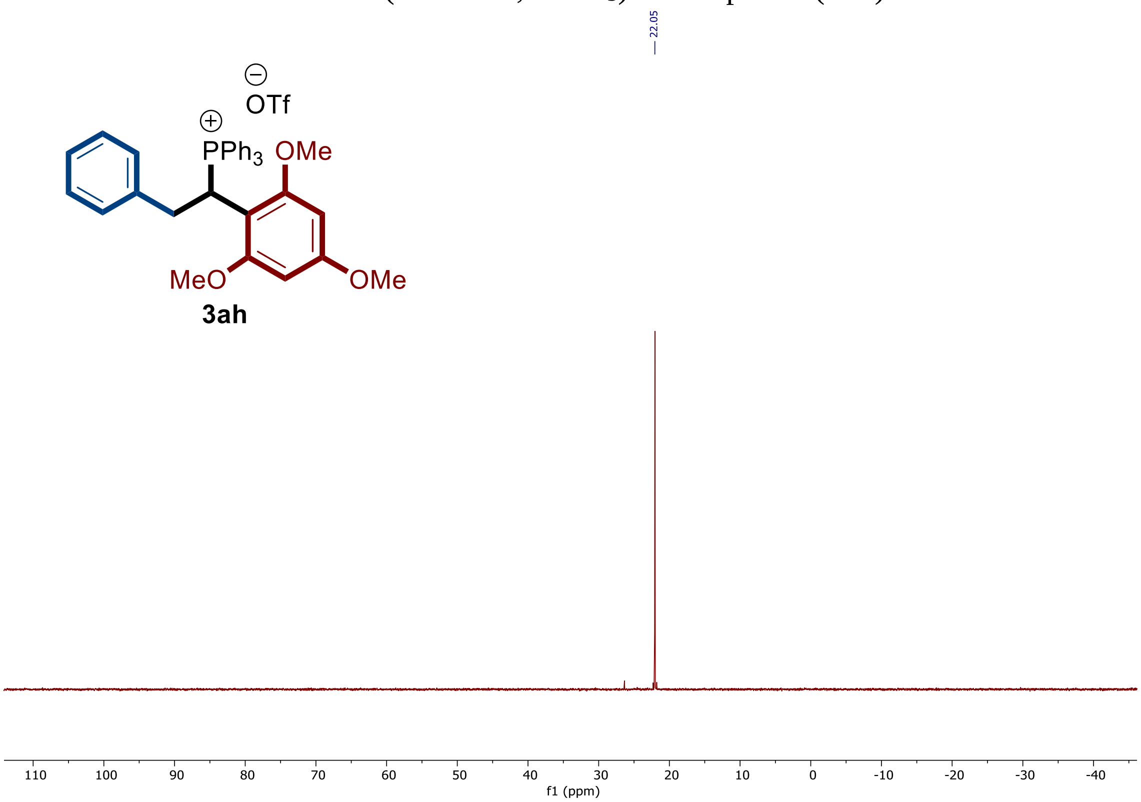

^{19}F NMR (376 MHz, $CDCl_3$) of compound (**3ah**)

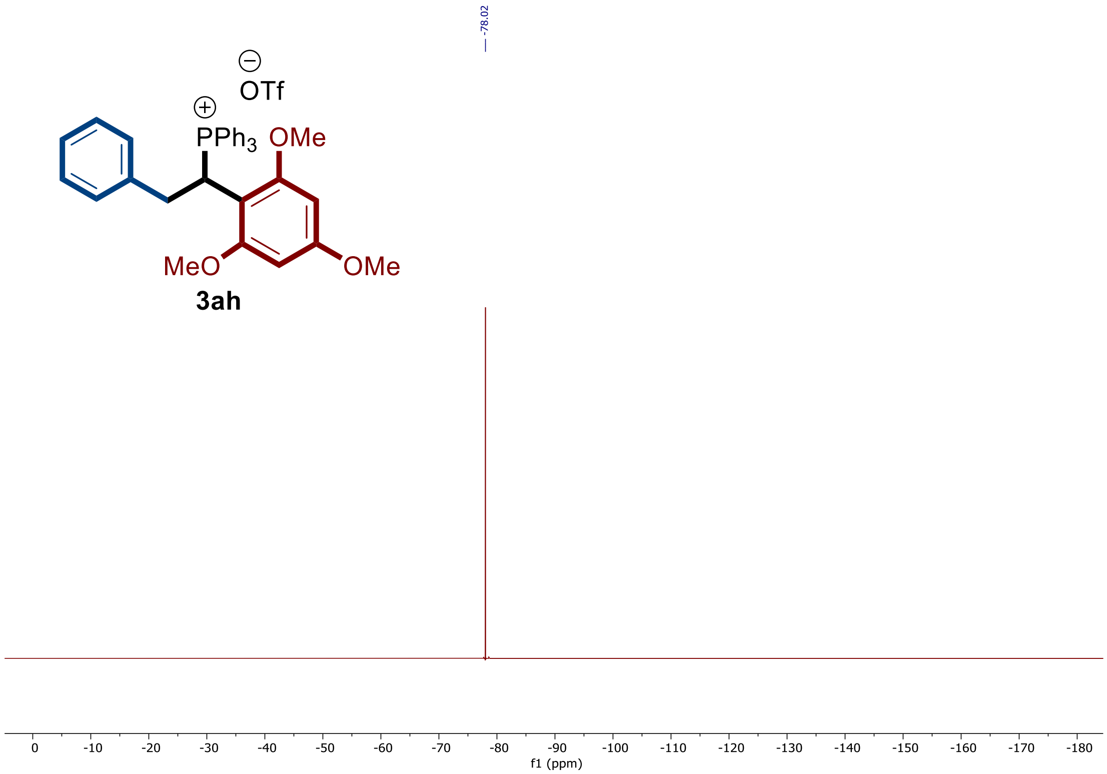

^{1}H NMR (400 MHz, CDCl3) of compound (**3ai**)

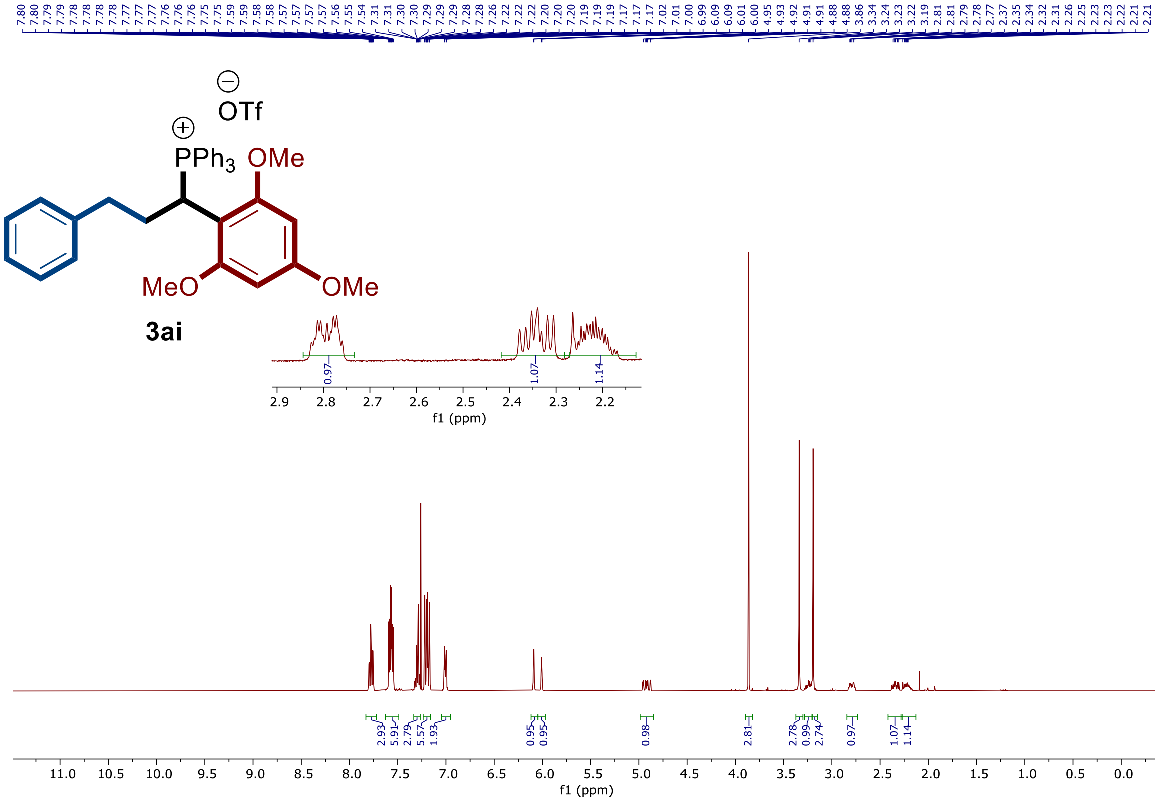

^{13}C{^{1}H} NMR (101 MHz, CDCl3) of compound (**3ai**)

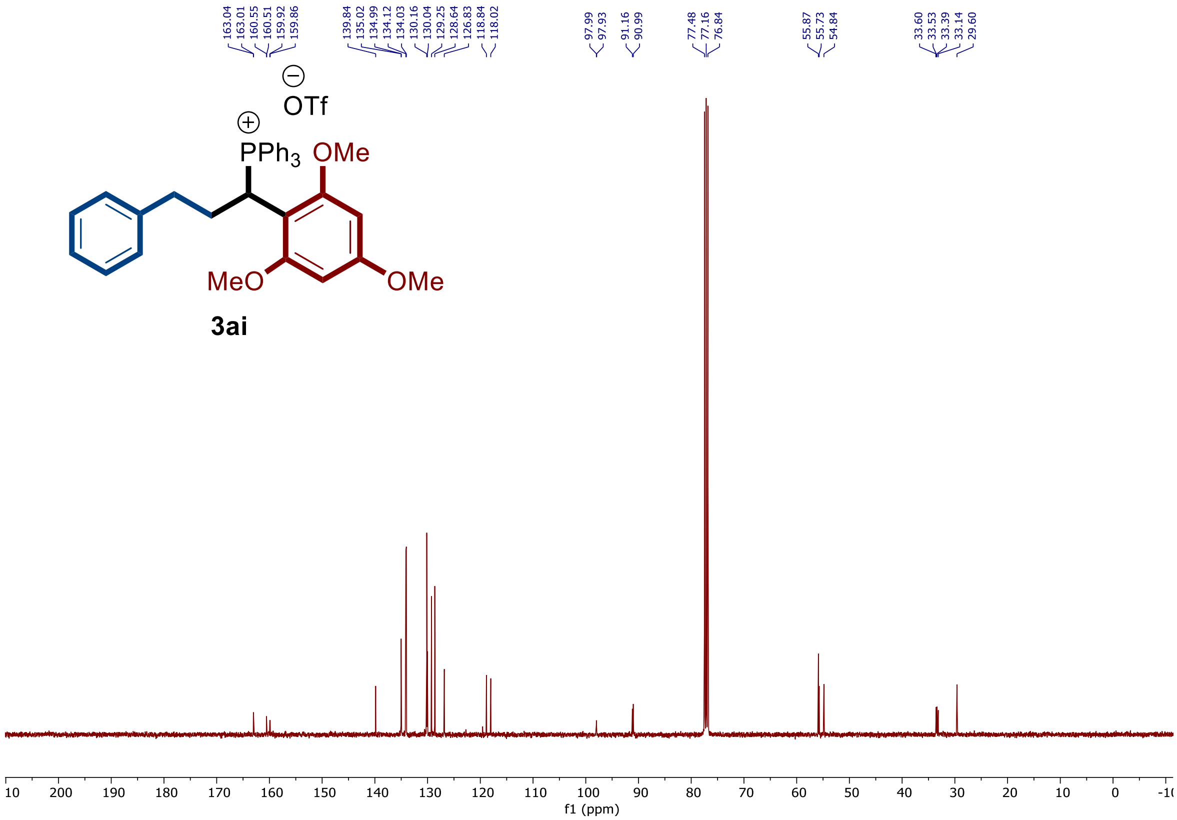

^{31}P NMR (162 MHz, $CDCl_3$) of compound (**3ai**)

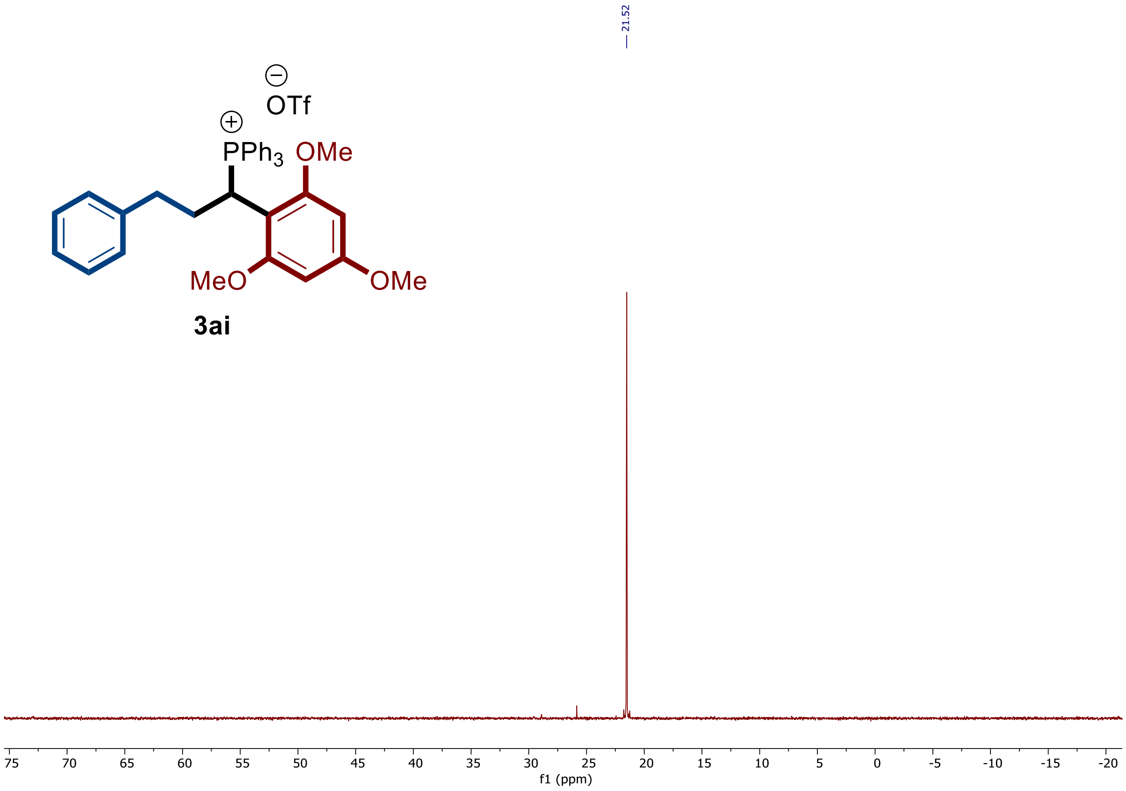

^{19}F NMR (376 MHz, $CDCl_3$) of compound (**3ai**)

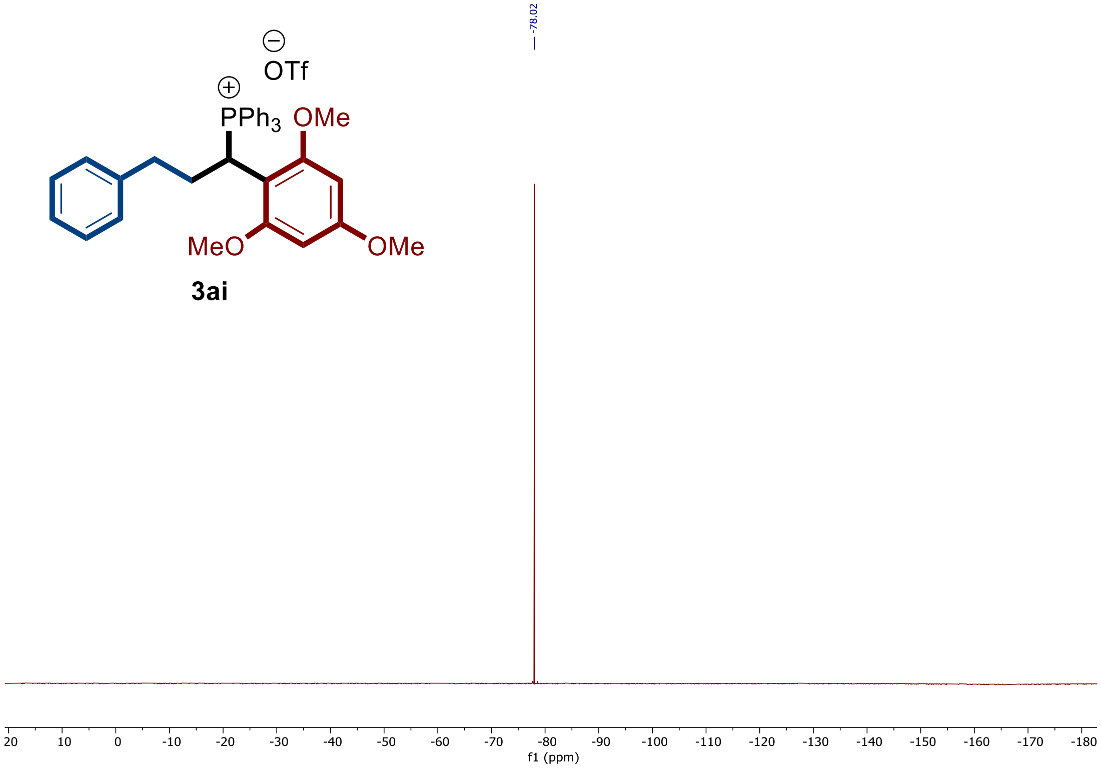

^{1}H NMR (400 MHz, $CDCl_3$) of compound (**3aj**)

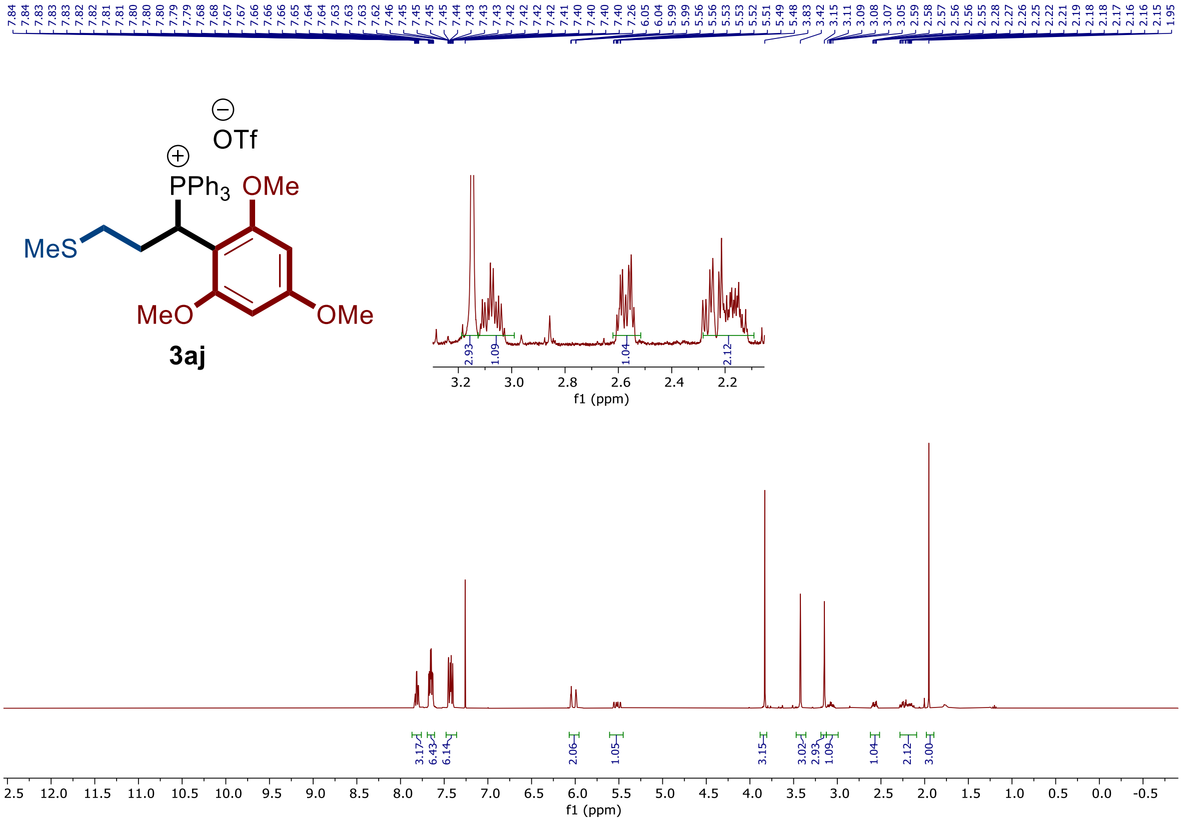

^{13}C{^{1}H} NMR (101 MHz, $CDCl_3$) of compound (**3aj**)

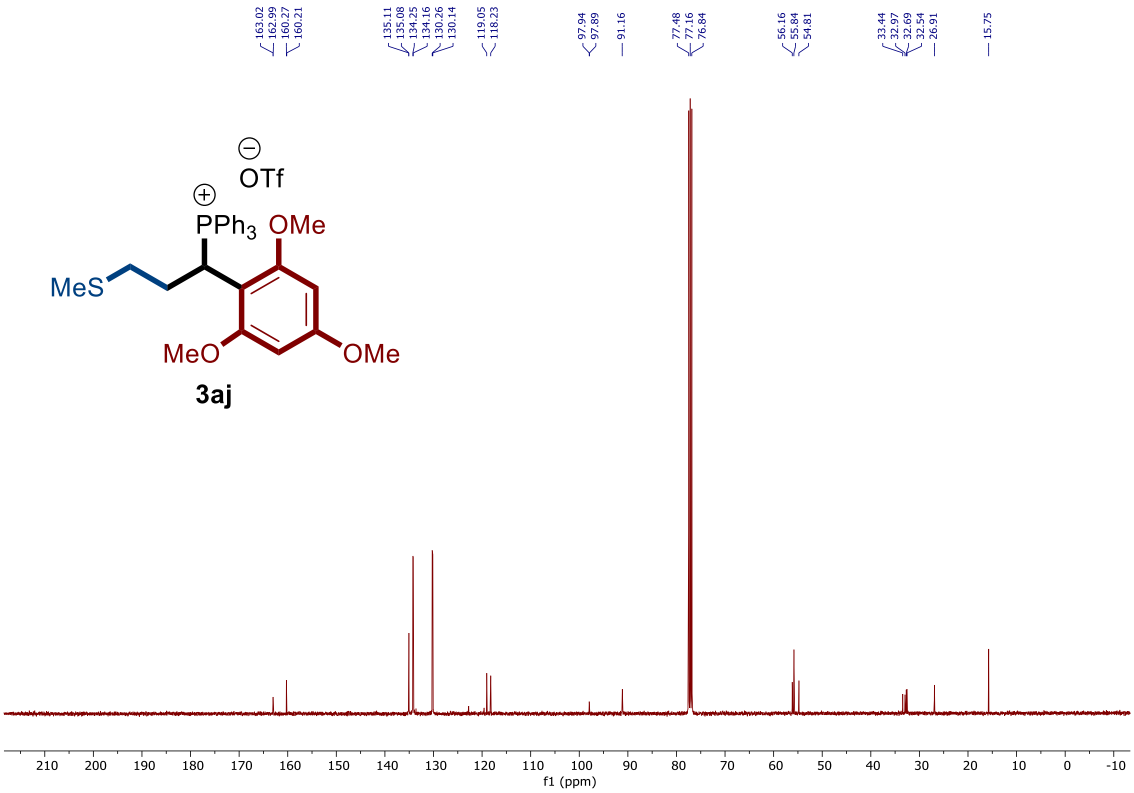

^{31}P NMR (162 MHz, $CDCl_3$) of compound (**3aj**)

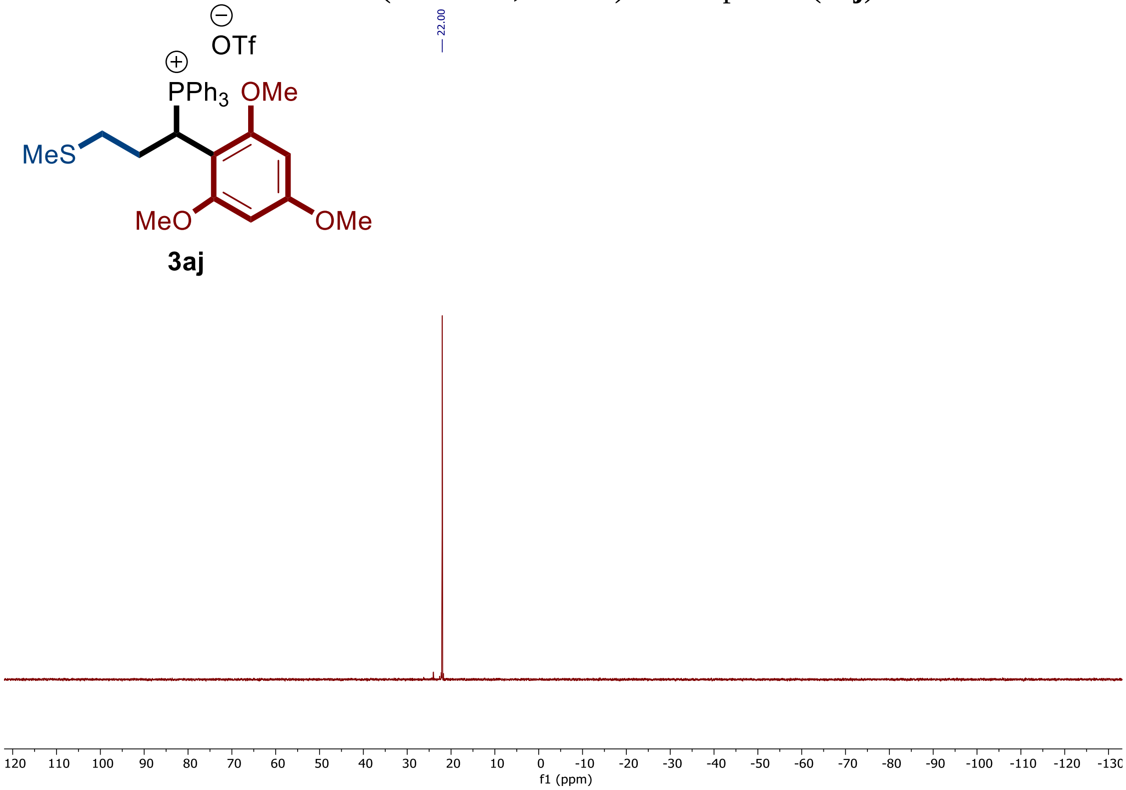

^{19}F NMR (376 MHz, $CDCl_3$) of compound (**3aj**)

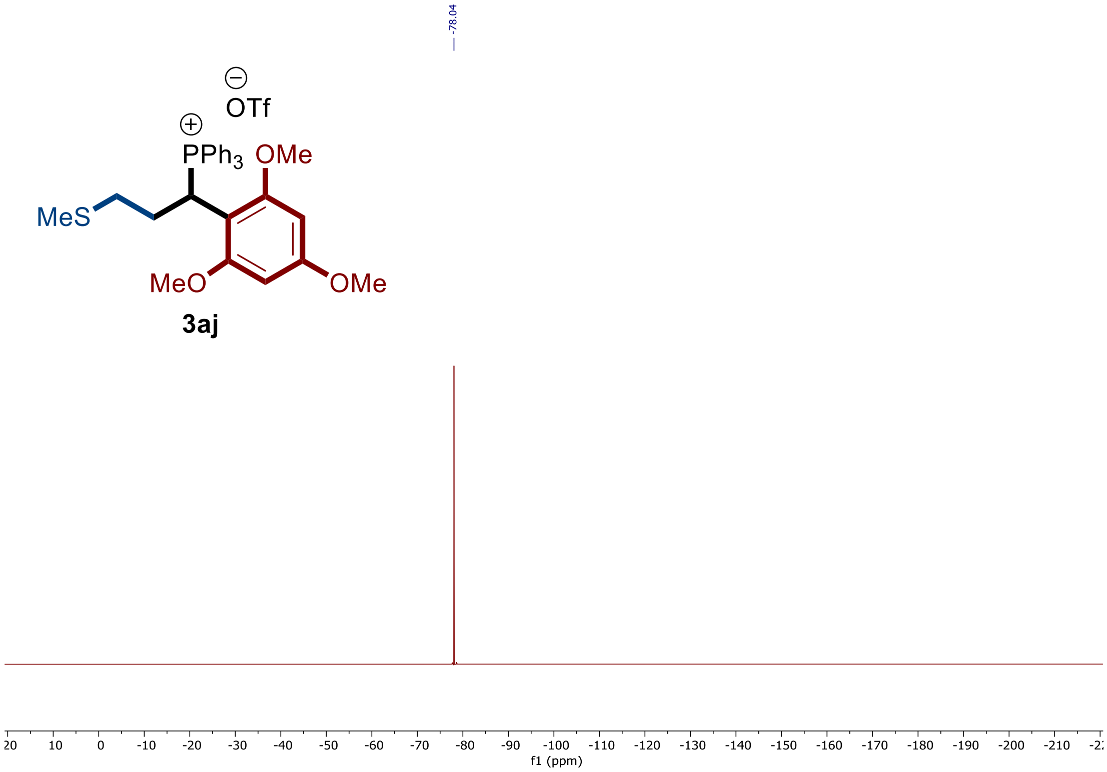

^{1}H NMR (400 MHz, CDCl3) of compound (**3ak**)

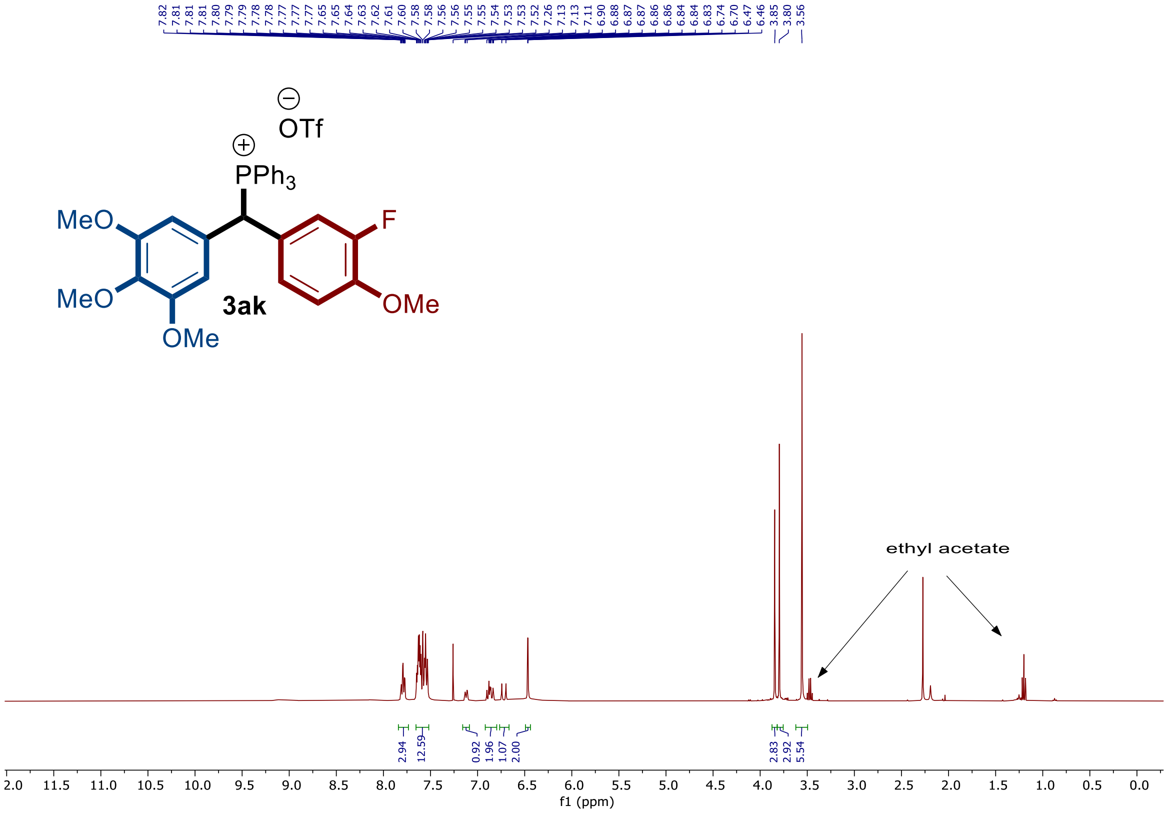

^{13}C{^{1}H} NMR (101 MHz, CDCl3) of compound (**3ak**)

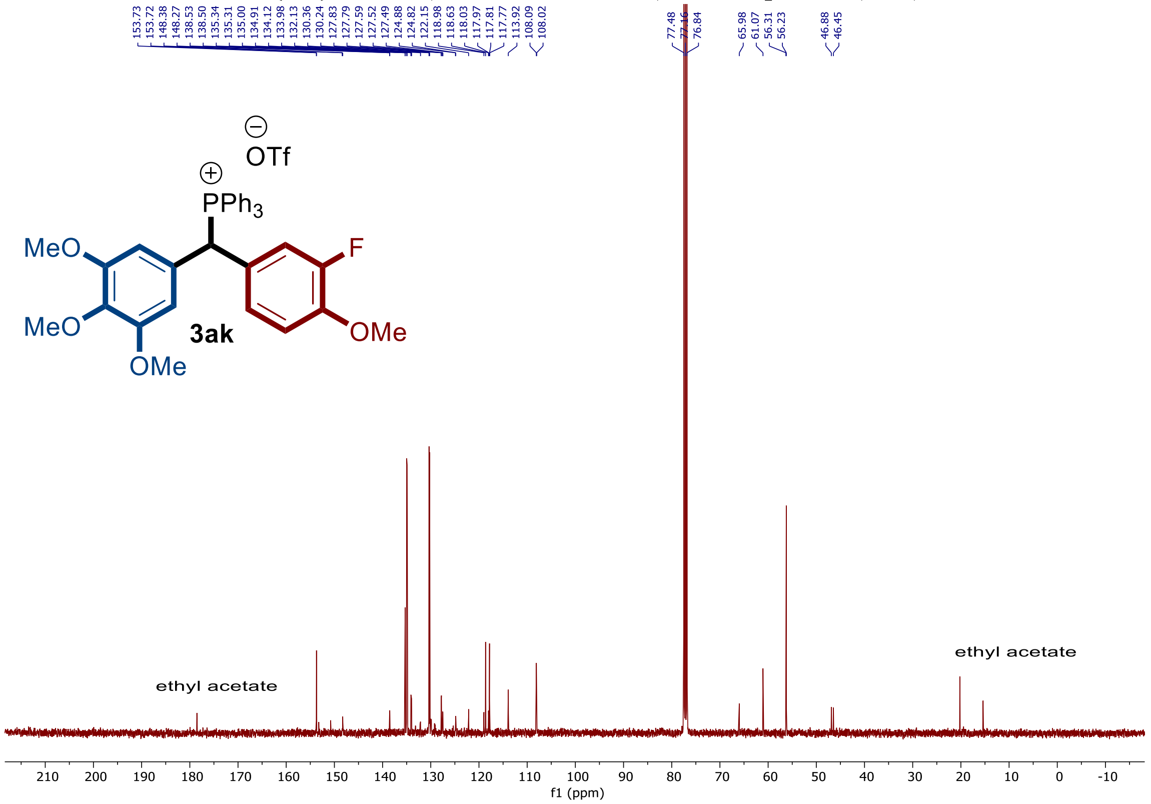

^{31}P NMR (162 MHz, $CDCl_3$) of compound (**3ak**)

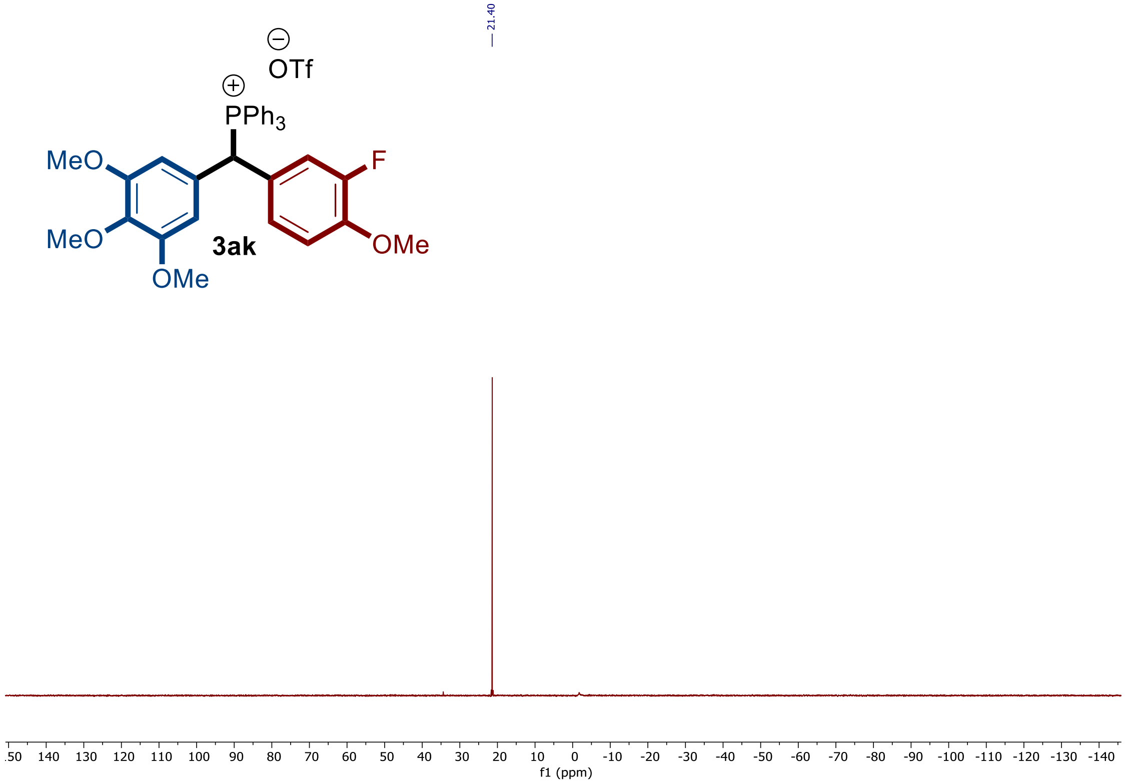

^{19}F NMR (376 MHz, $CDCl_3$) of compound (**3ak**)

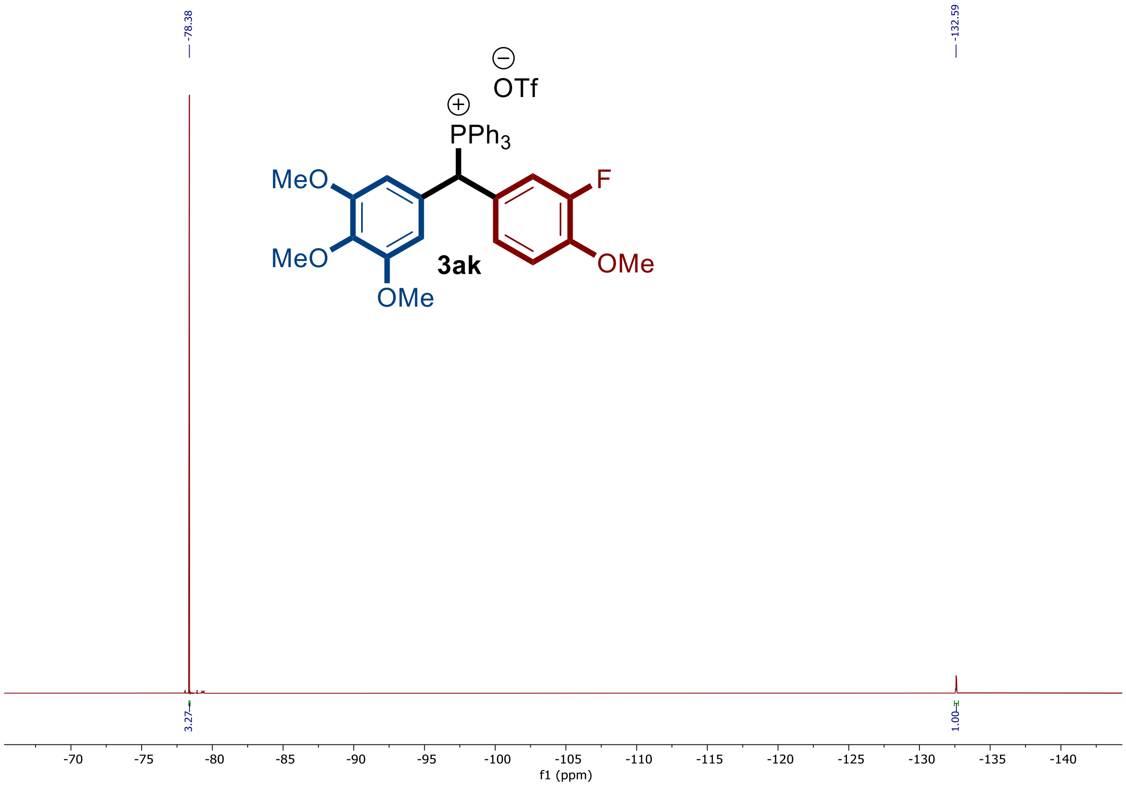

^{1}H NMR (400 MHz, $CDCl_3$) of compound (**5a**)

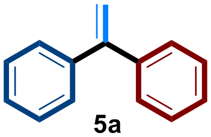

5a

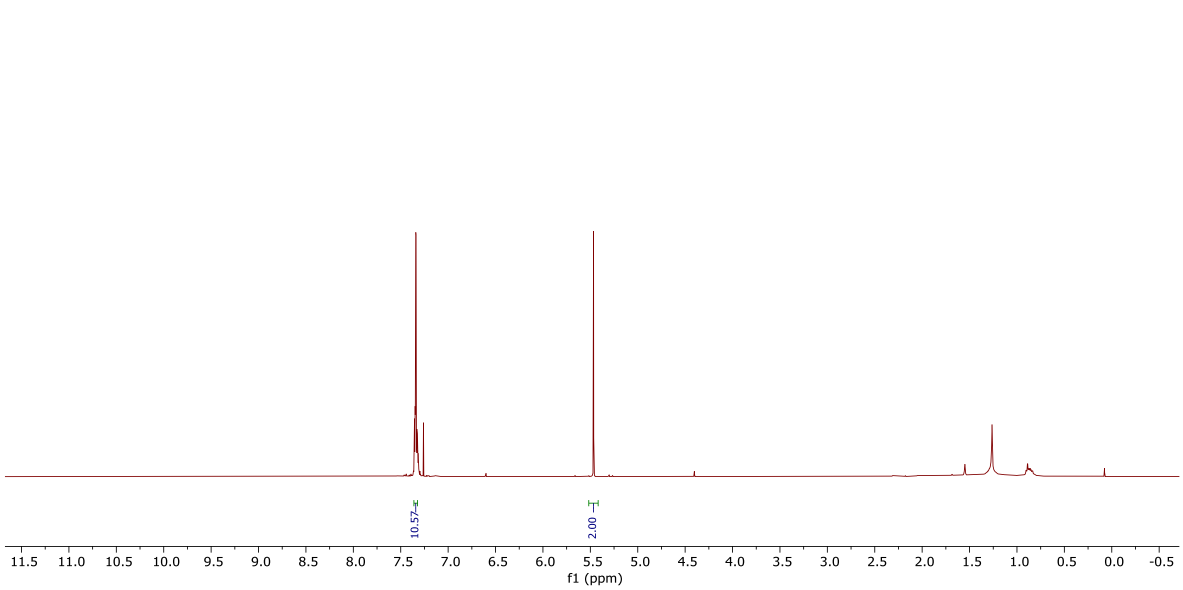

^{1}H NMR (400 MHz, $CDCl_3$) of compound (**5b**)

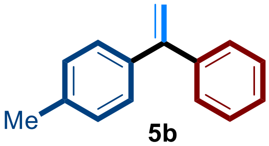

5b

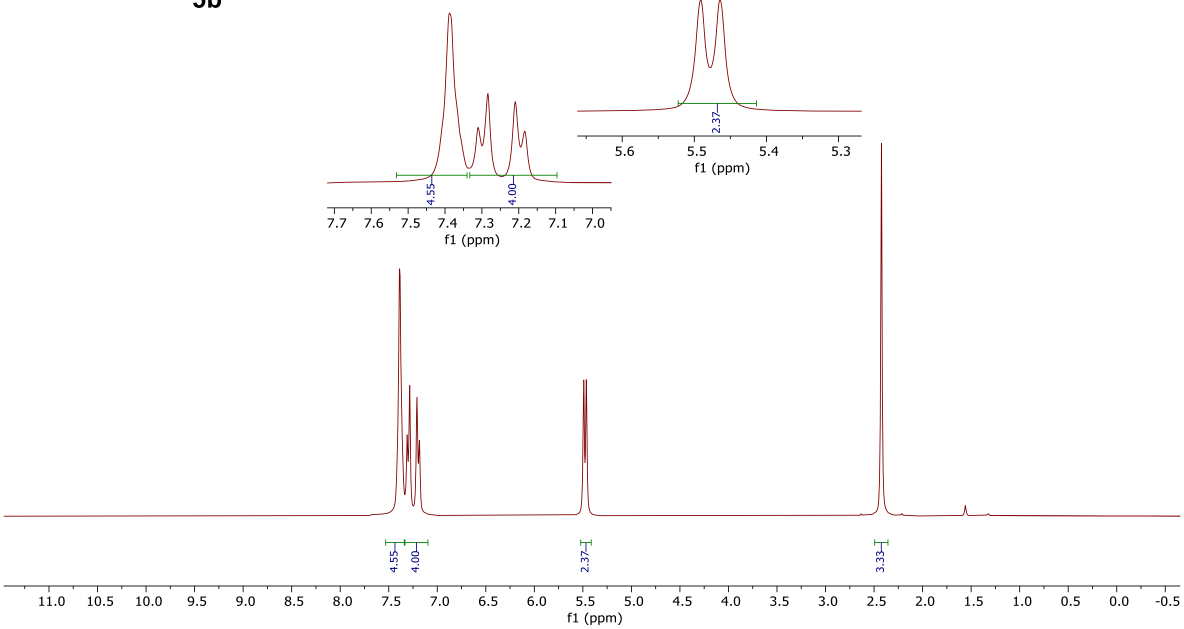

^{1}H NMR (400 MHz, $CDCl_3$) of compound (**5c**)

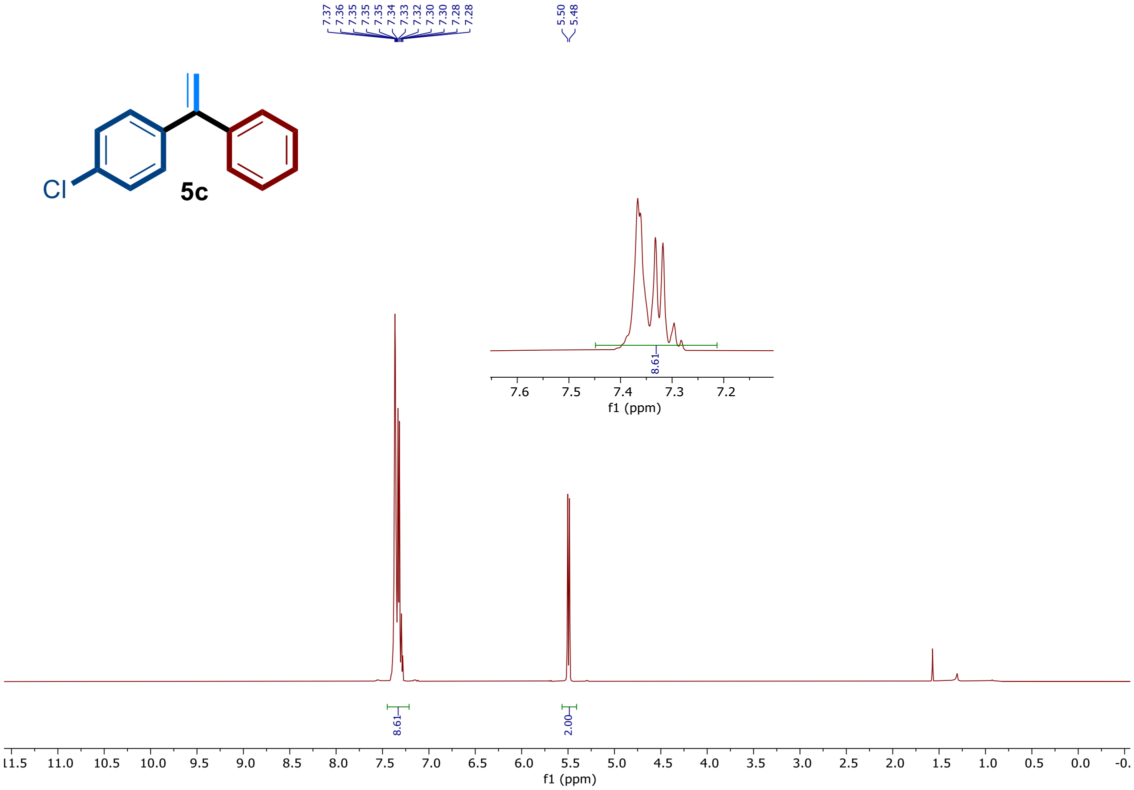

^{1}H NMR (400 MHz, $CDCl_3$) of compound (**5d**)

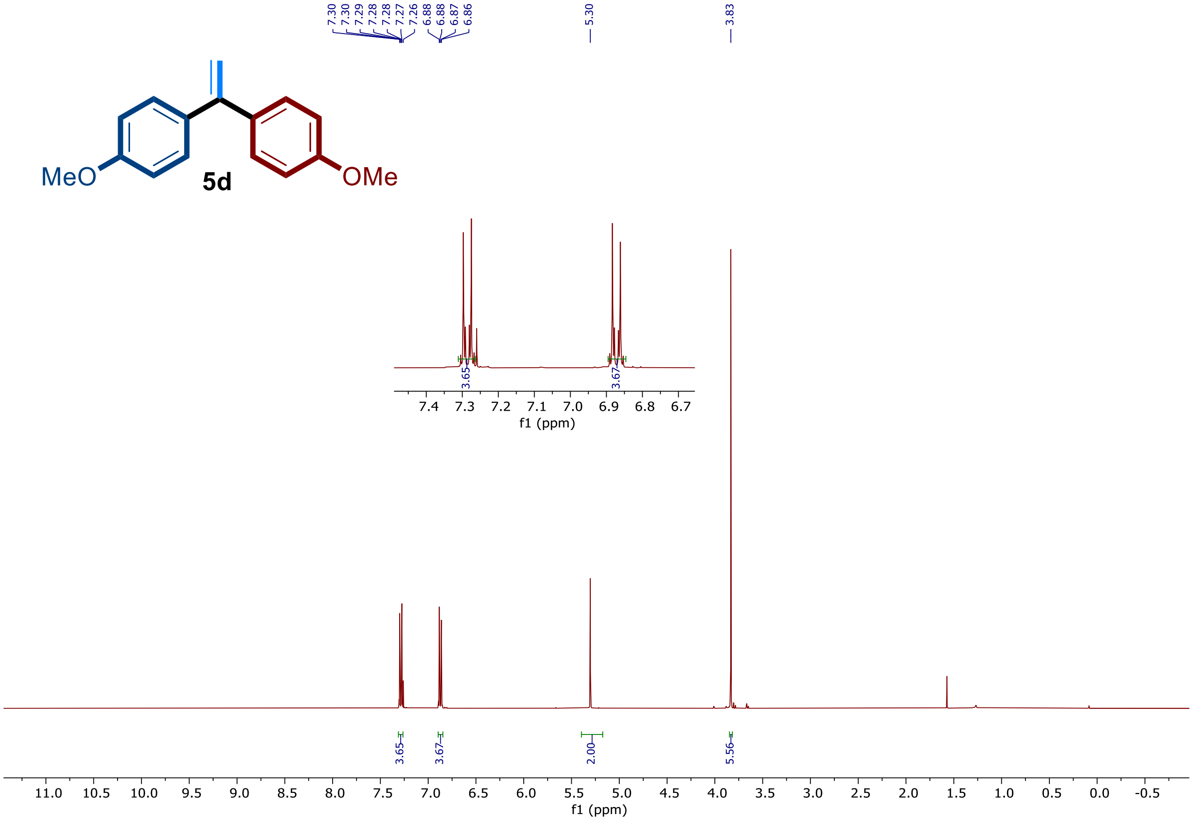

^{1}H NMR (400 MHz, $CDCl_3$) of compound (**5e**)

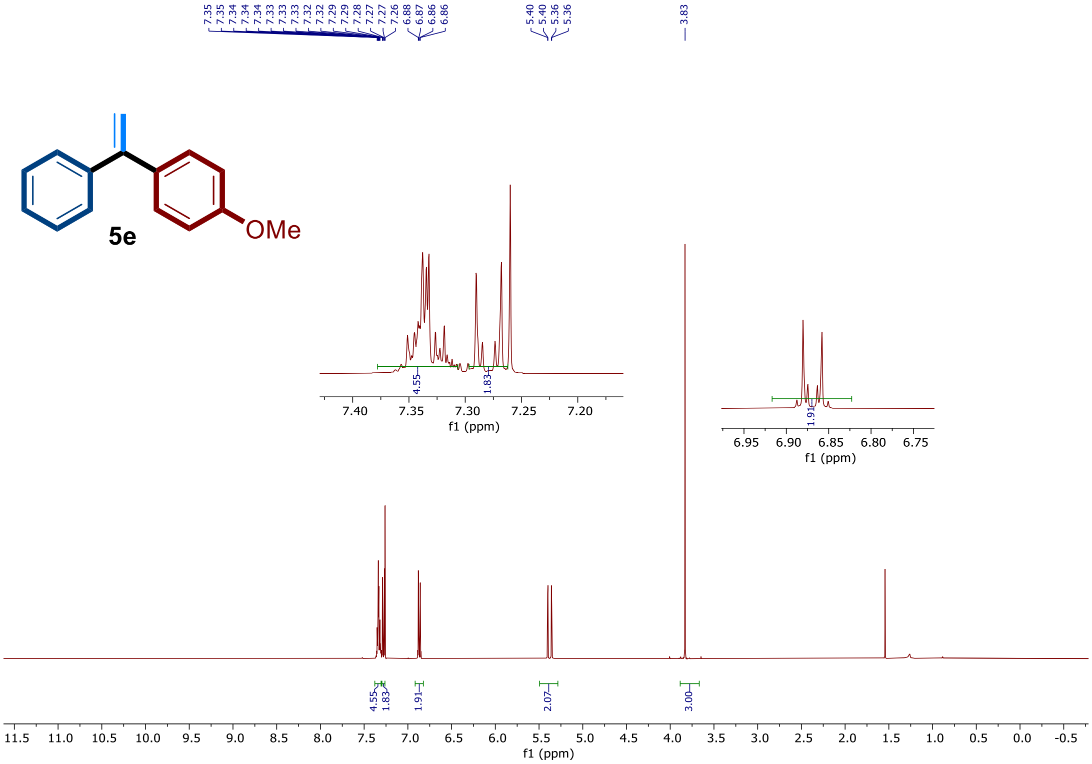

^{1}H NMR (400 MHz, $CDCl_3$) of compound (**5f**)

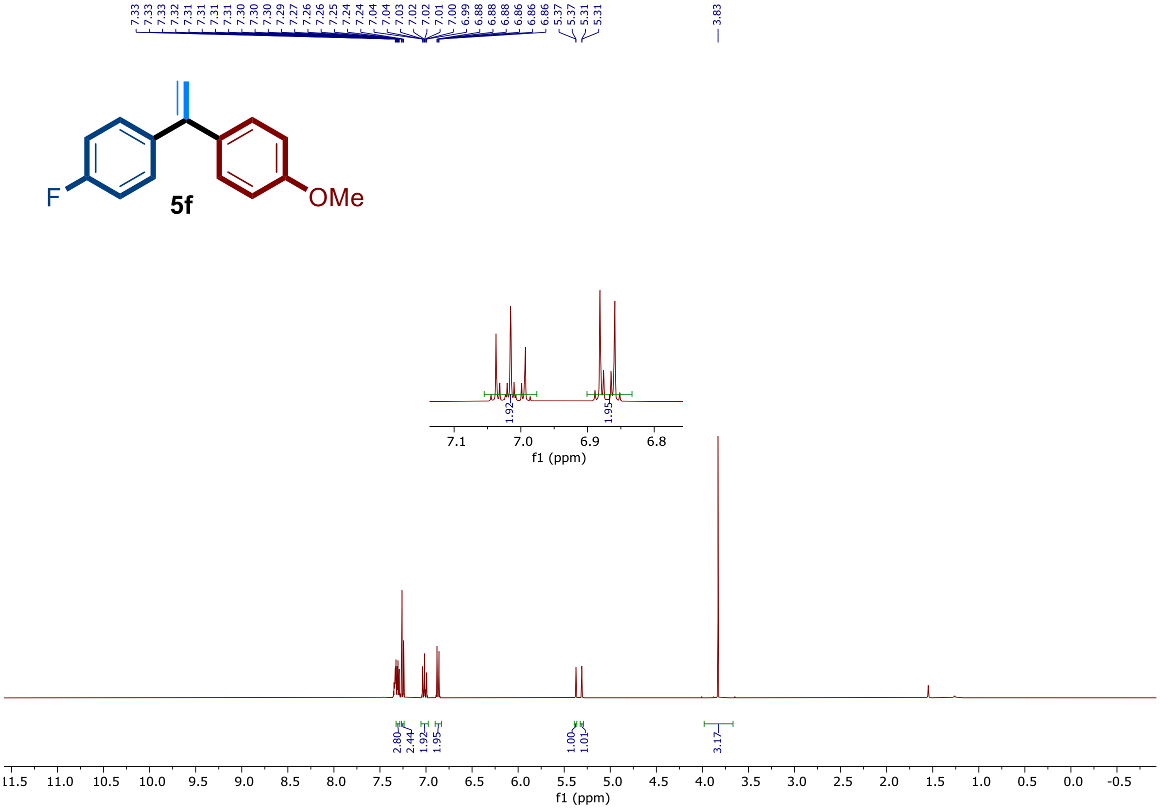

^{1}H NMR (400 MHz, $CDCl_3$) of compound (**5g**)

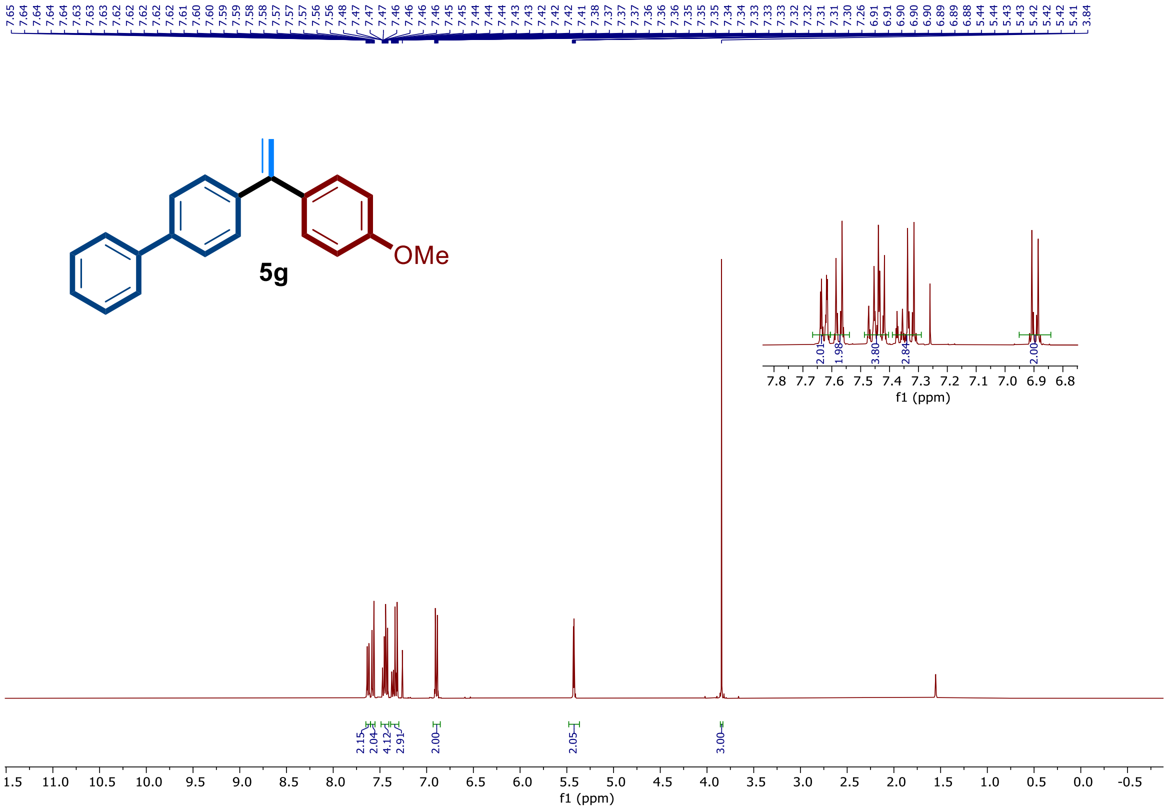

^{1}H NMR (400 MHz, $CDCl_3$) of compound (**5h**)

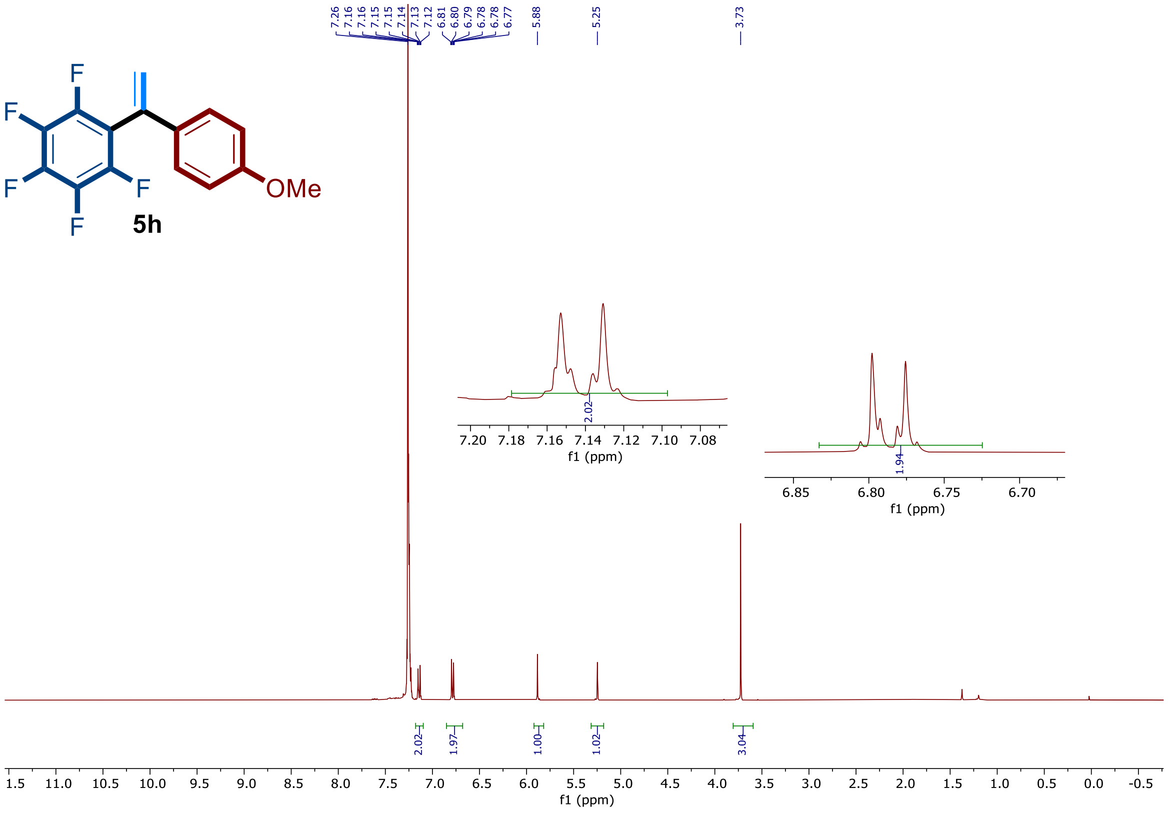

$^{13}C\{^{1}H\}$ NMR (101 MHz, $CDCl_3$) of compound (**5h**)

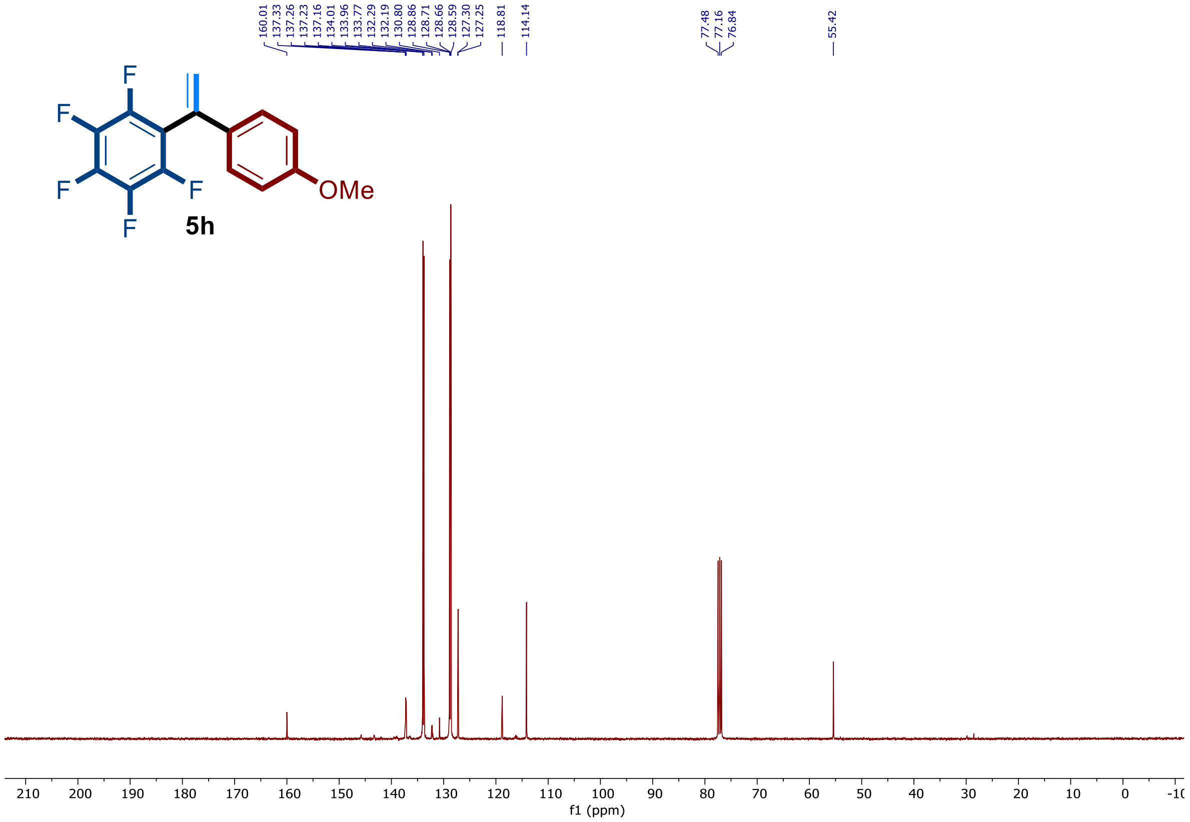

^{19}F NMR (376 MHz, $CDCl_3$) of compound (**5h**)

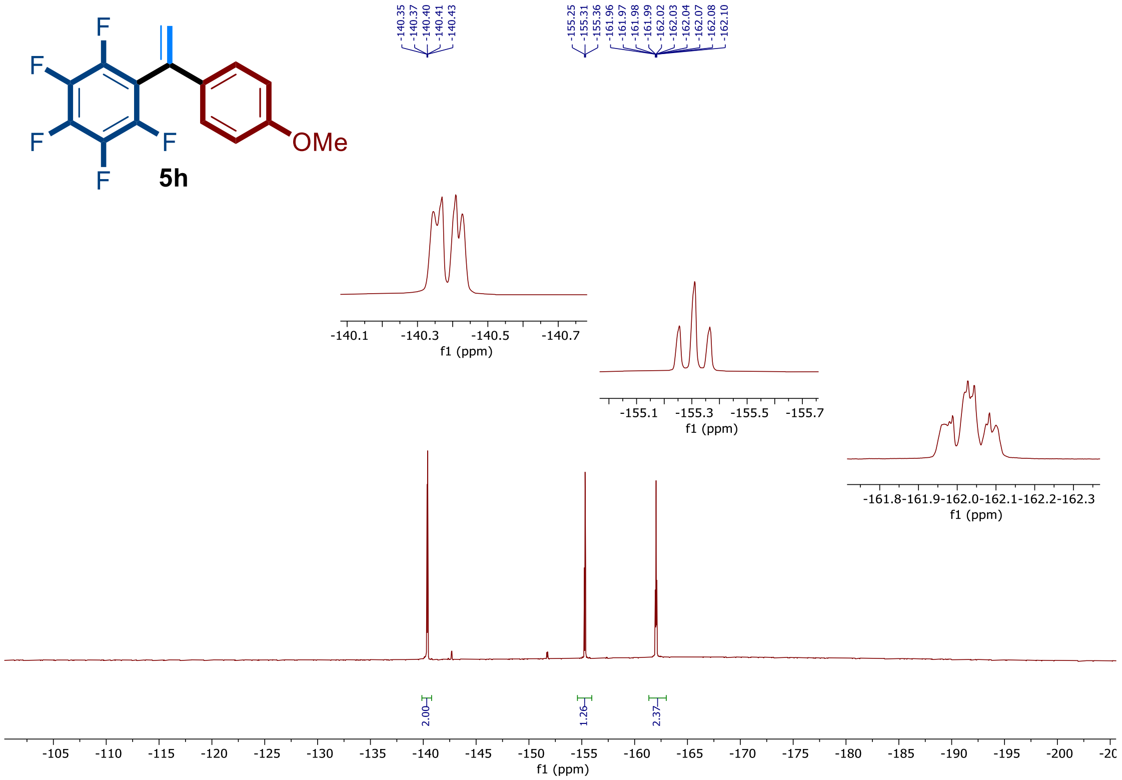

^{1}H NMR (400 MHz, $CDCl_3$) of compound (**5i**)

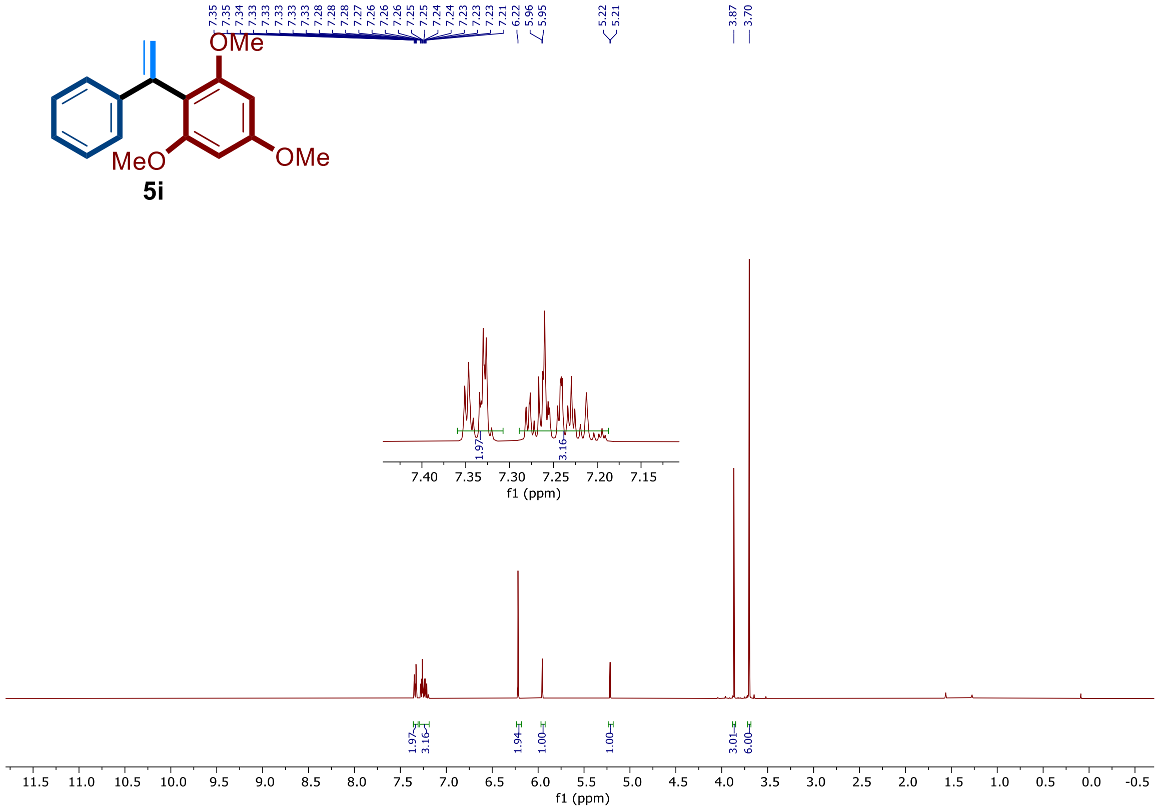

^{1}H NMR (400 MHz, $CDCl_3$) of compound (**5j**)

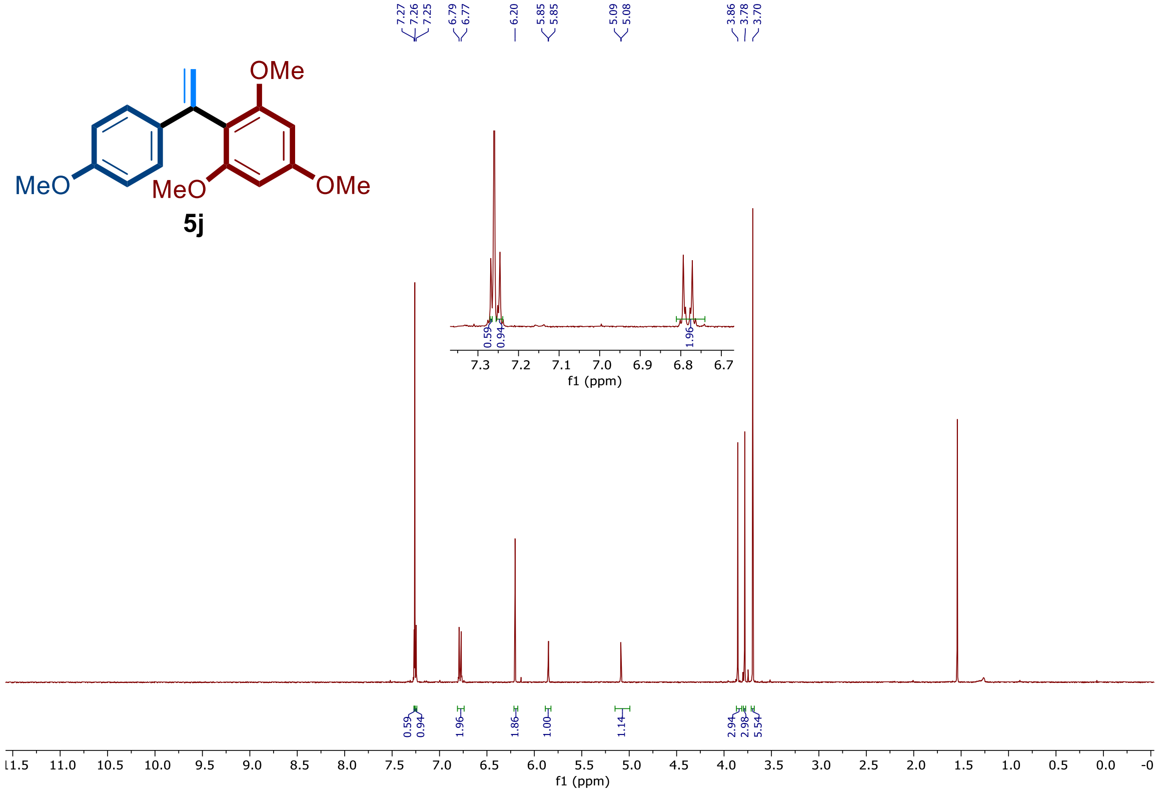

^{1}H NMR (400 MHz, $CDCl_3$) of compound (**5k**)

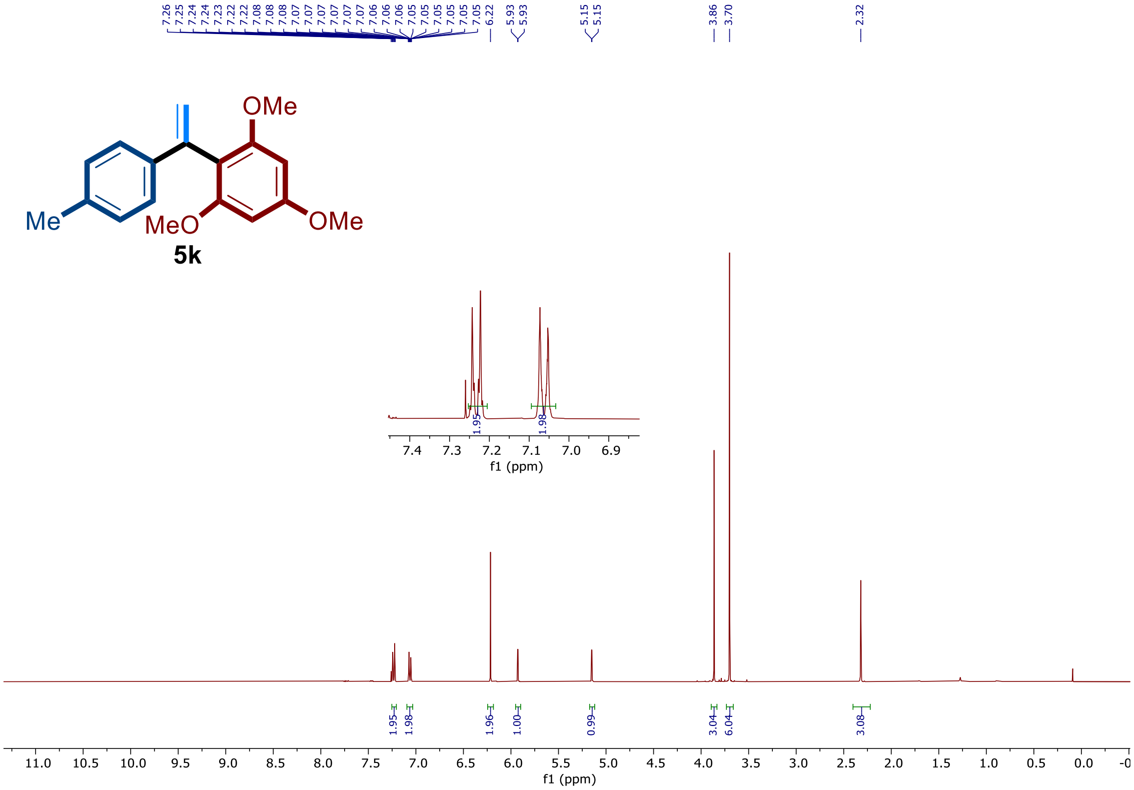

^{1}H NMR (400 MHz, $CDCl_3$) of compound (**5l**)

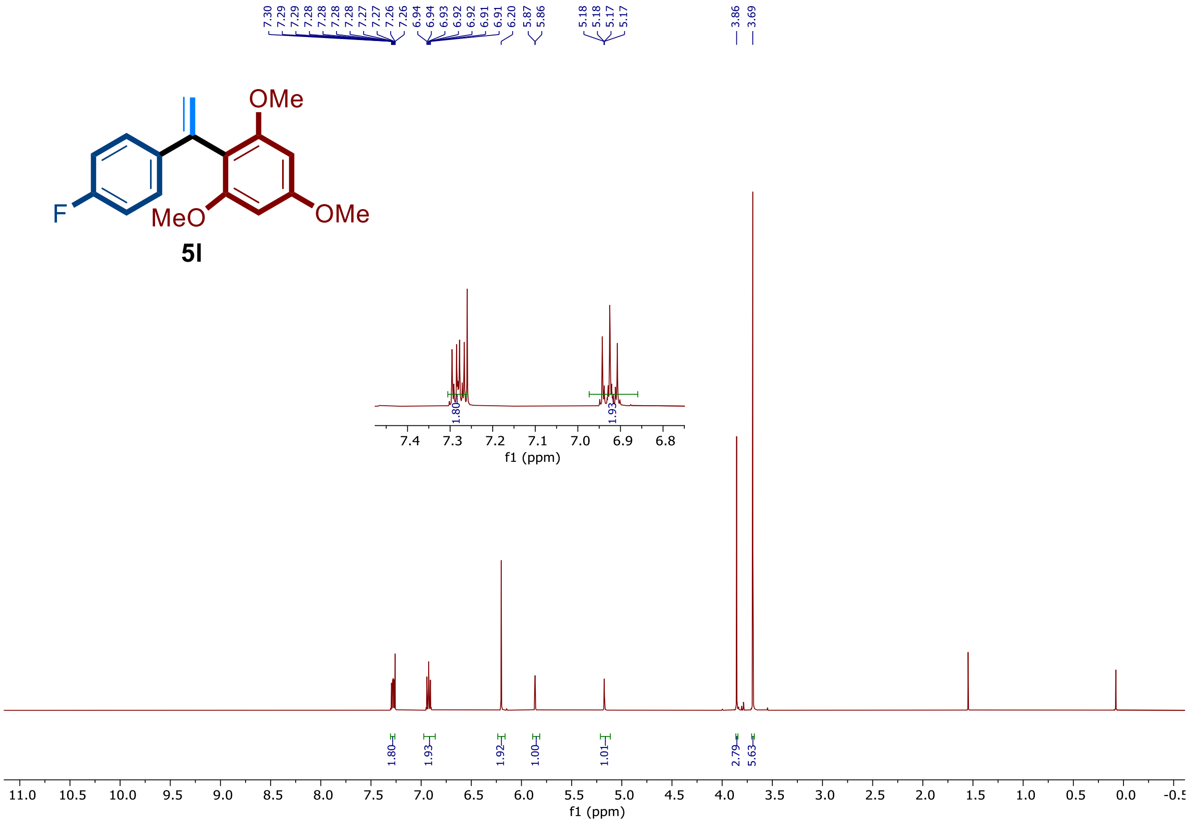

^{1}H NMR (400 MHz, $CDCl_3$) of compound (**5m**)

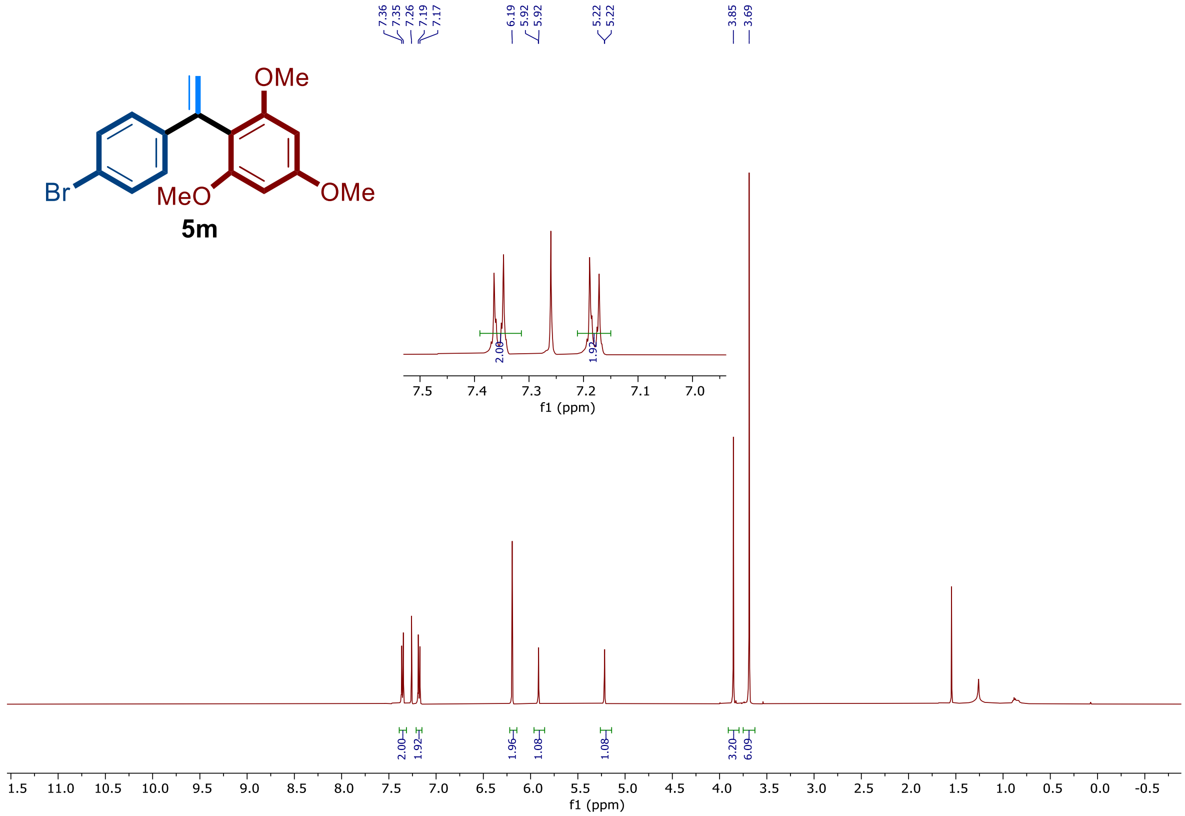

$^{13}C\{^{1}H\}$ NMR (101 MHz, $CDCl_3$) of compound (**5m**)

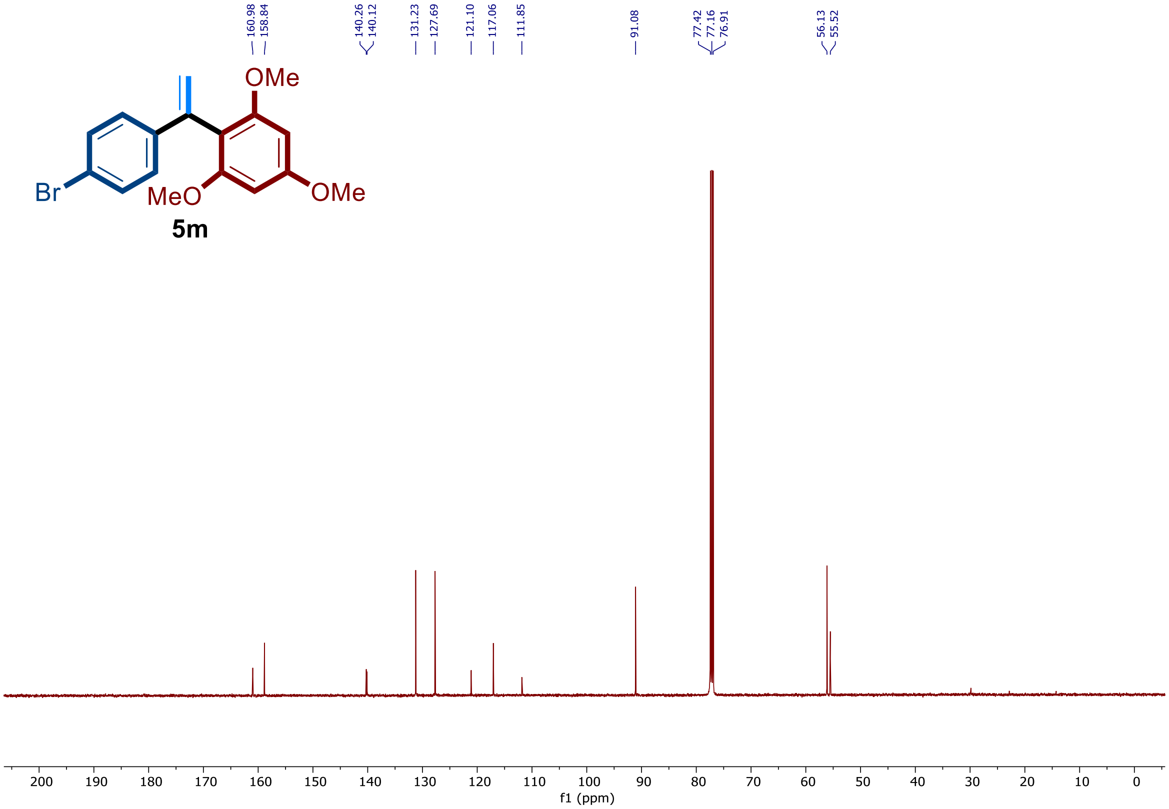

^{1}H NMR (400 MHz, $CDCl_3$) of compound (**5n**)

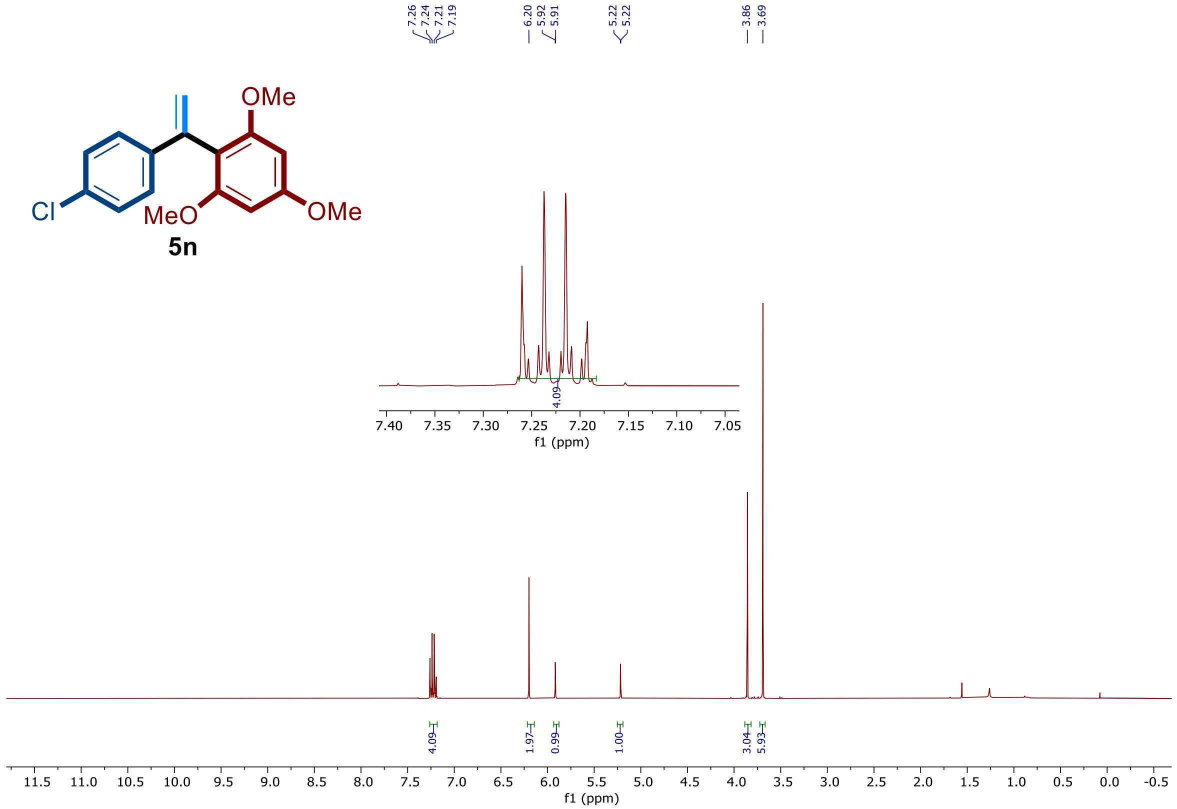

$^{13}C\{^{1}H\}$ NMR (101 MHz, $CDCl_3$) of compound (**5n**)

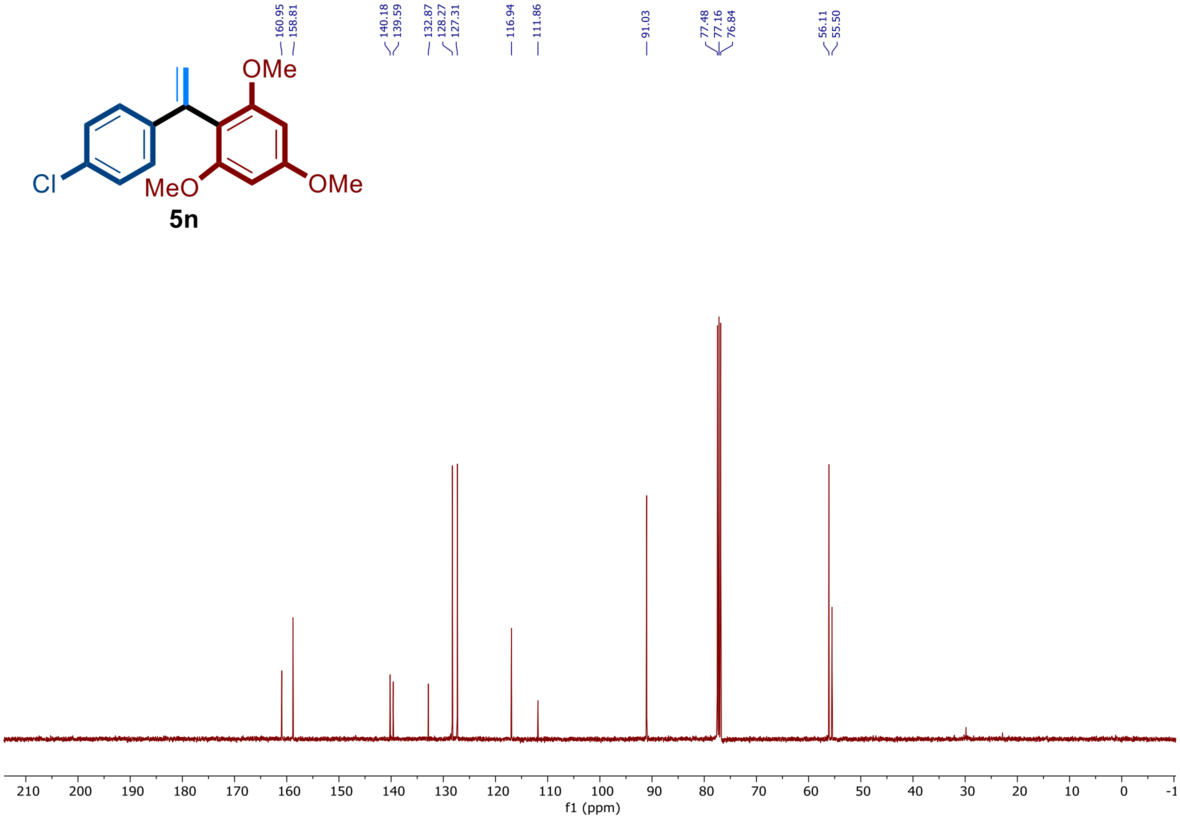

^{1}H NMR (400 MHz, $CDCl_3$) of compound (**5o**)

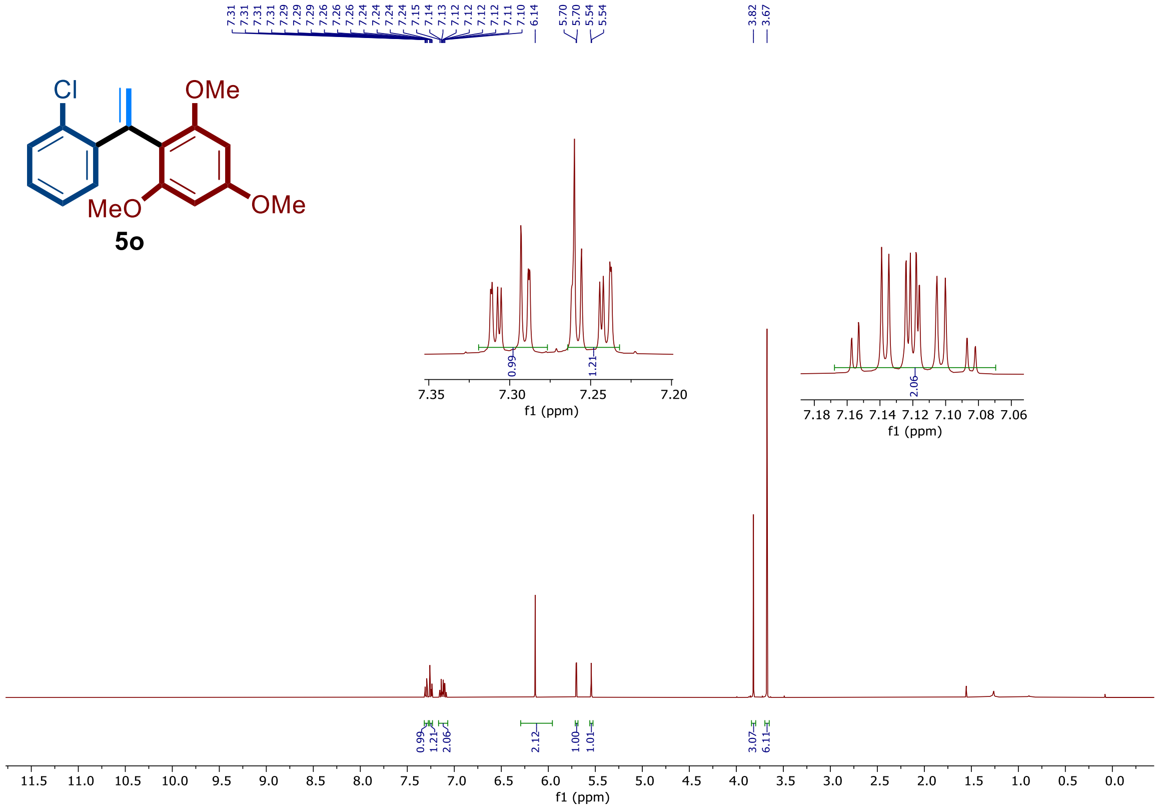

^{13}C{^{1}H} NMR (101 MHz, $CDCl_3$) of compound (**5o**)

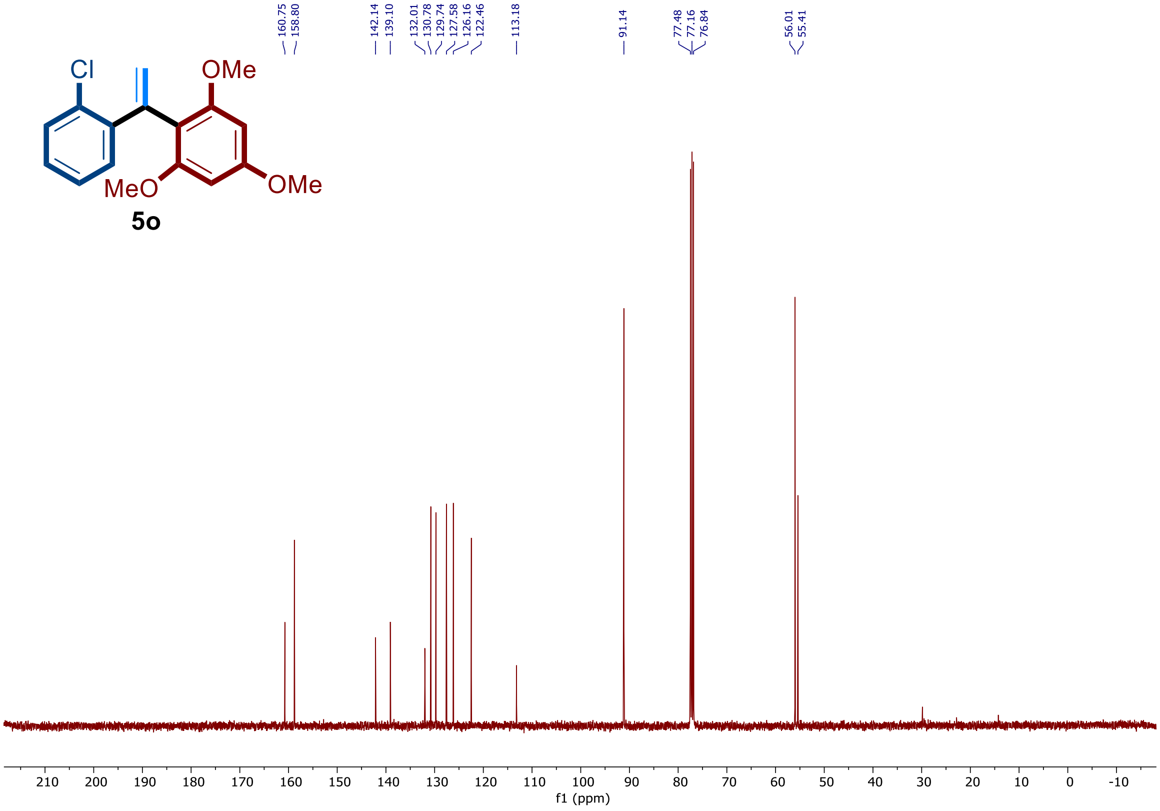

^{1}H NMR (400 MHz, $CDCl_3$) of compound (**5p**)

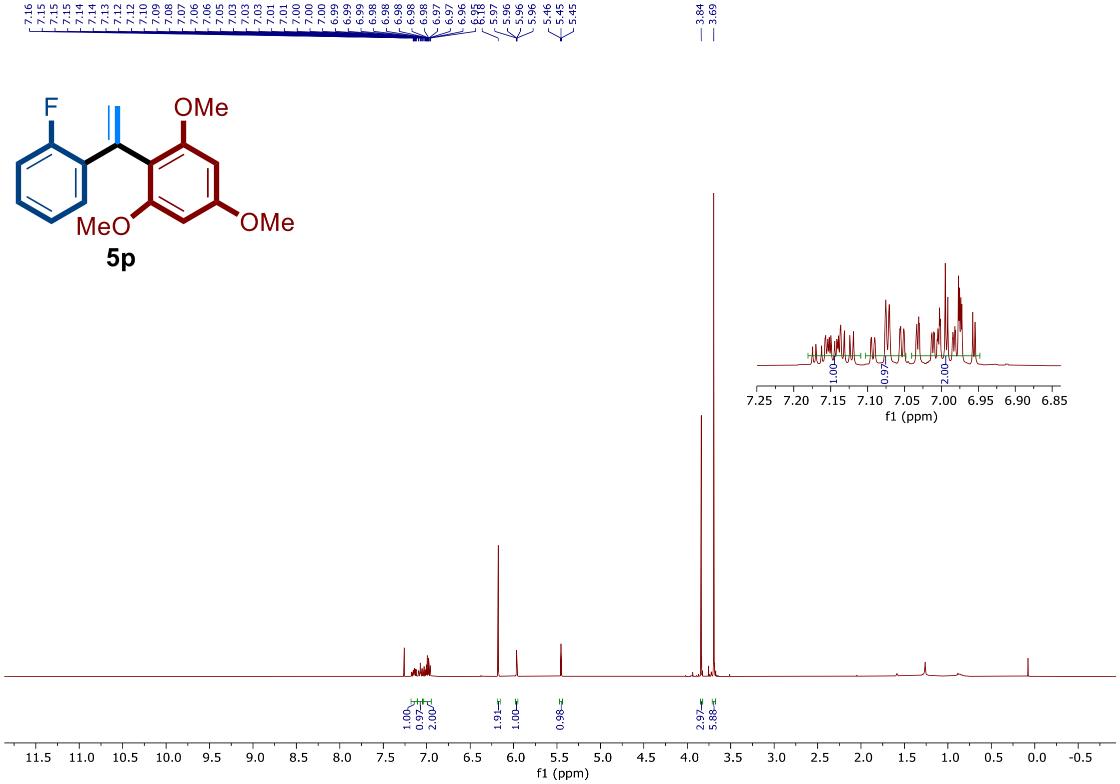

^{1}H NMR (400 MHz, $CDCl_3$) of compound (**5q**)

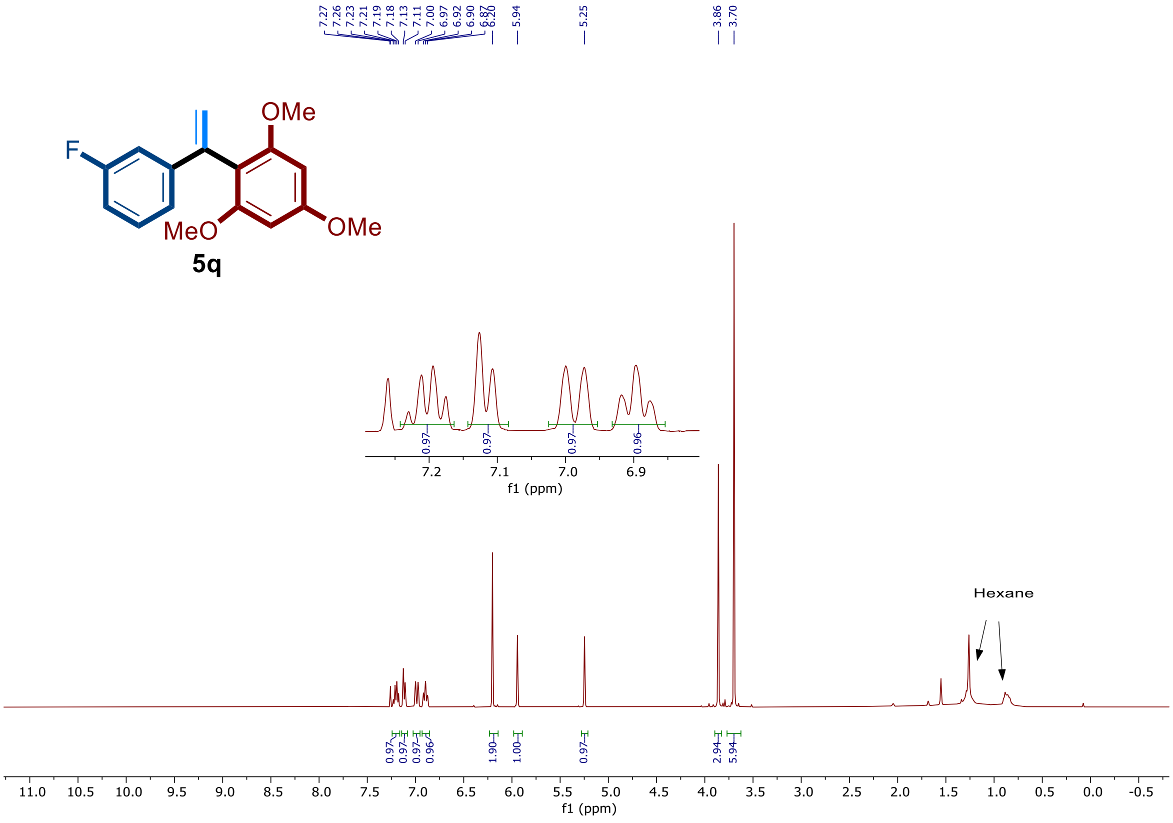

$^{13}C\{^{1}H\}$ NMR (101 MHz, $CDCl_3$) of compound (**5q**)

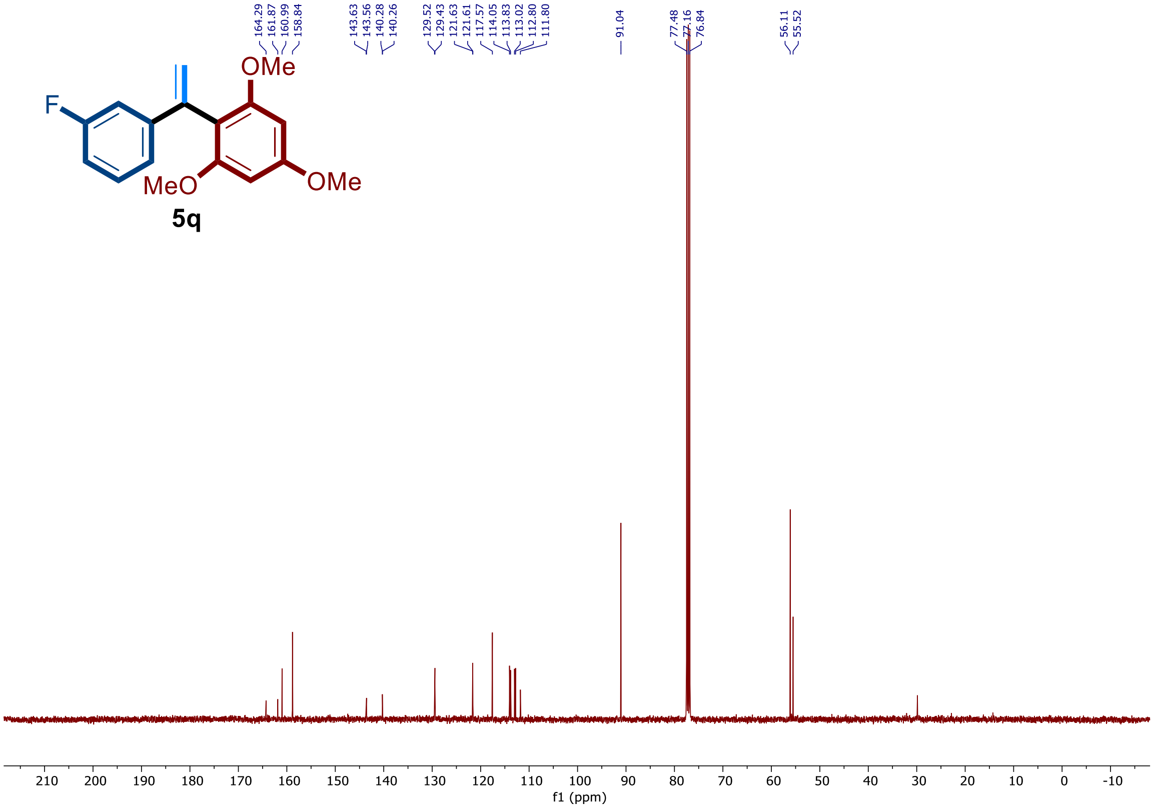

^{19}F NMR (376 MHz, $CDCl_3$) of compound (**5q**)

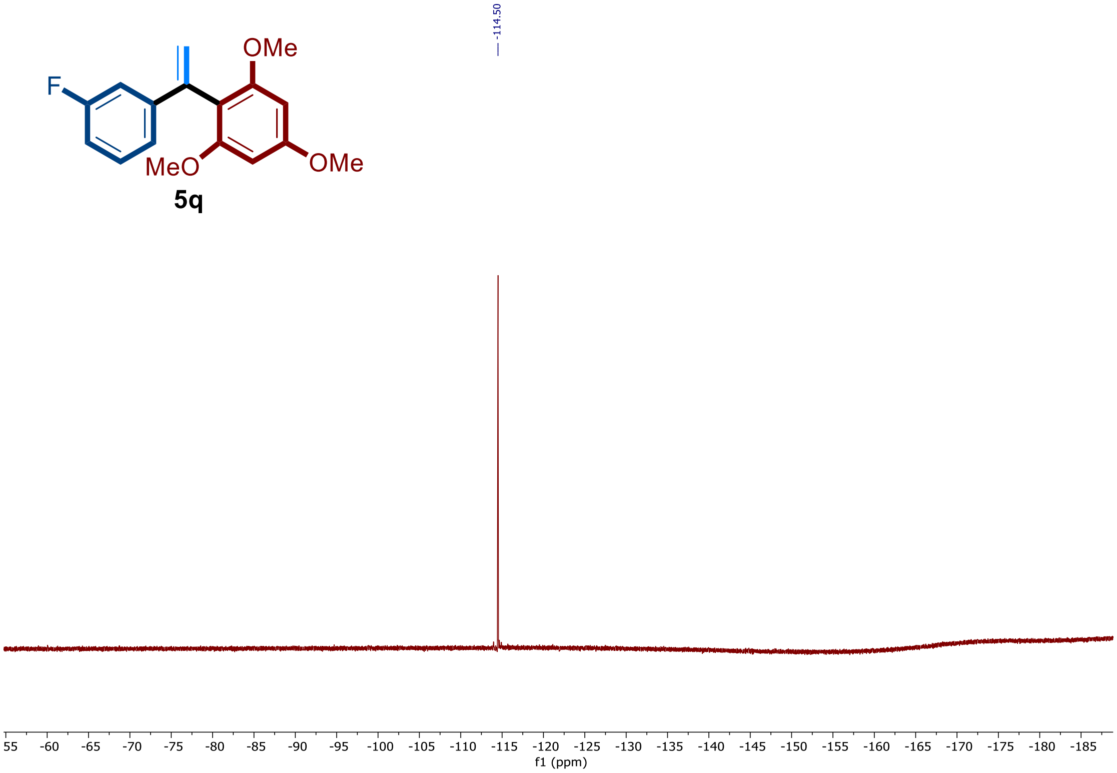

^{1}H NMR (400 MHz, $CDCl_3$) of compound (**5r**)

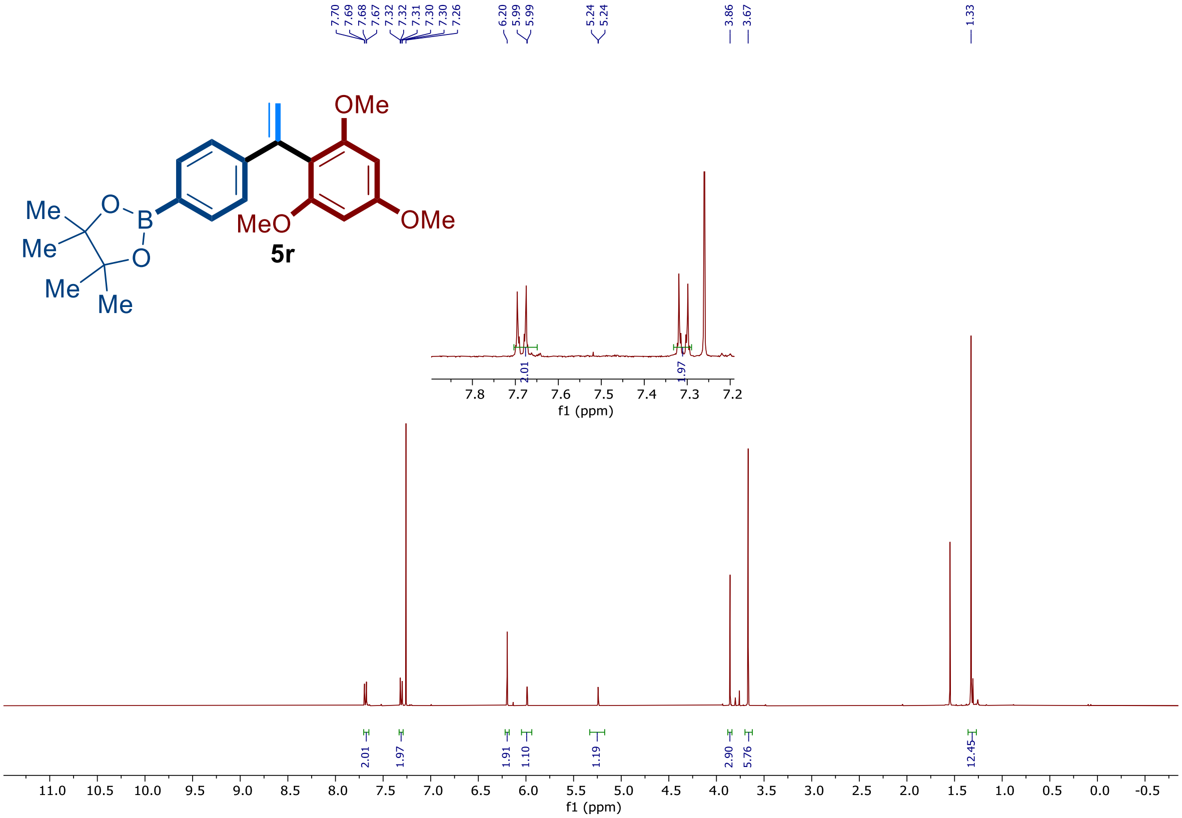

$^{13}C\{^{1}H\}$ NMR (101 MHz, $CDCl_3$) of compound (**5r**)

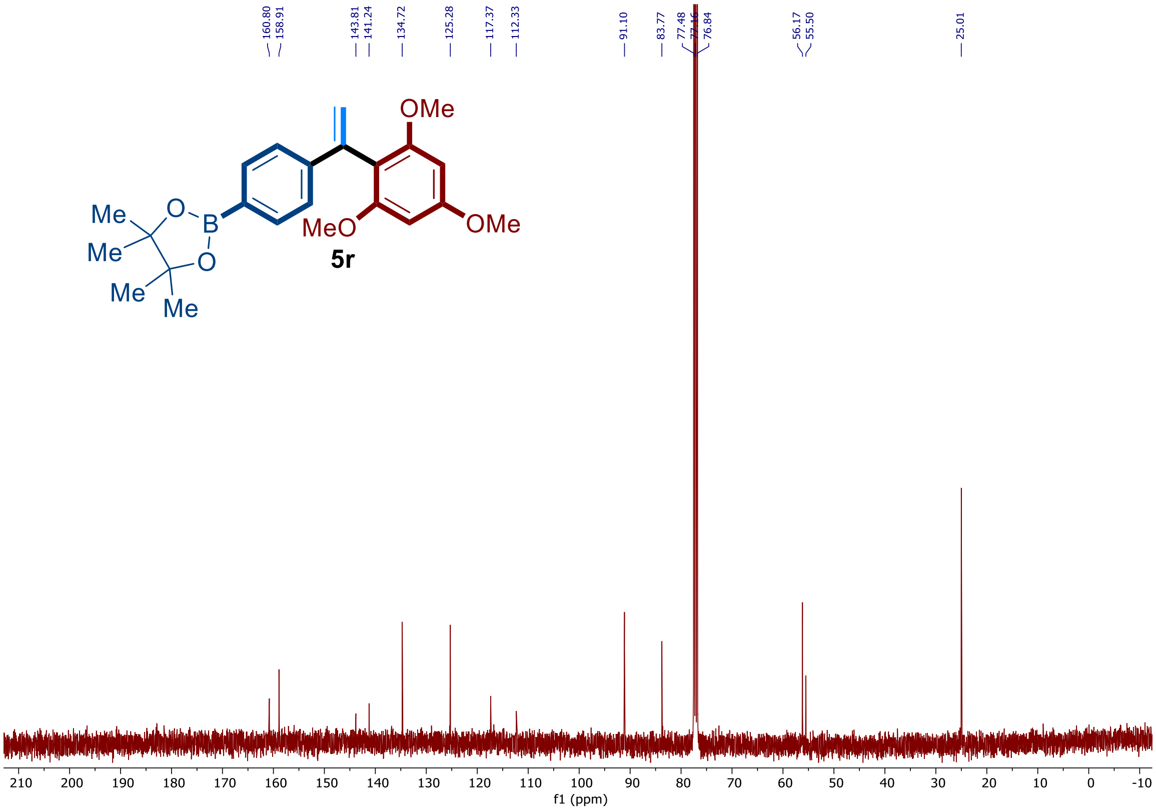

^{11}B NMR (128 MHz, $CDCl_3$) of compound (**5r**)

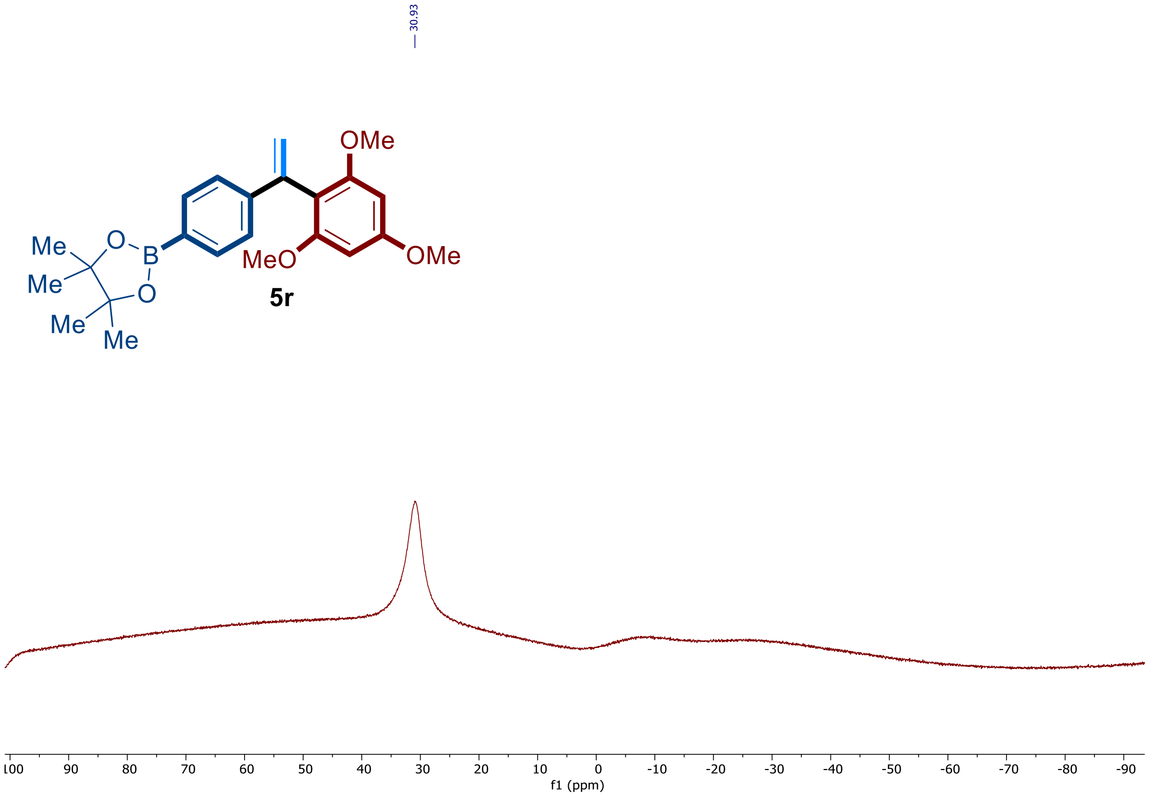

^{1}H NMR (400 MHz, $CDCl_3$) of compound (**5s**)

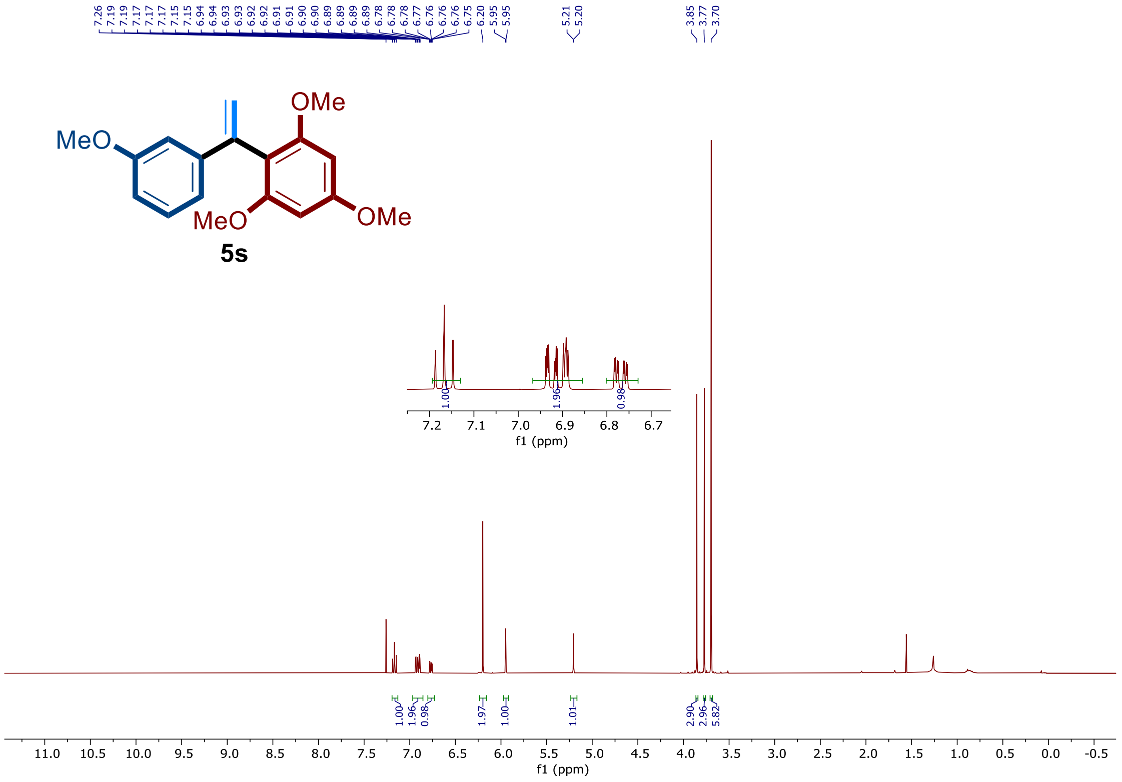

^{1}H NMR (400 MHz, $CDCl_3$) of compound (**5t**)

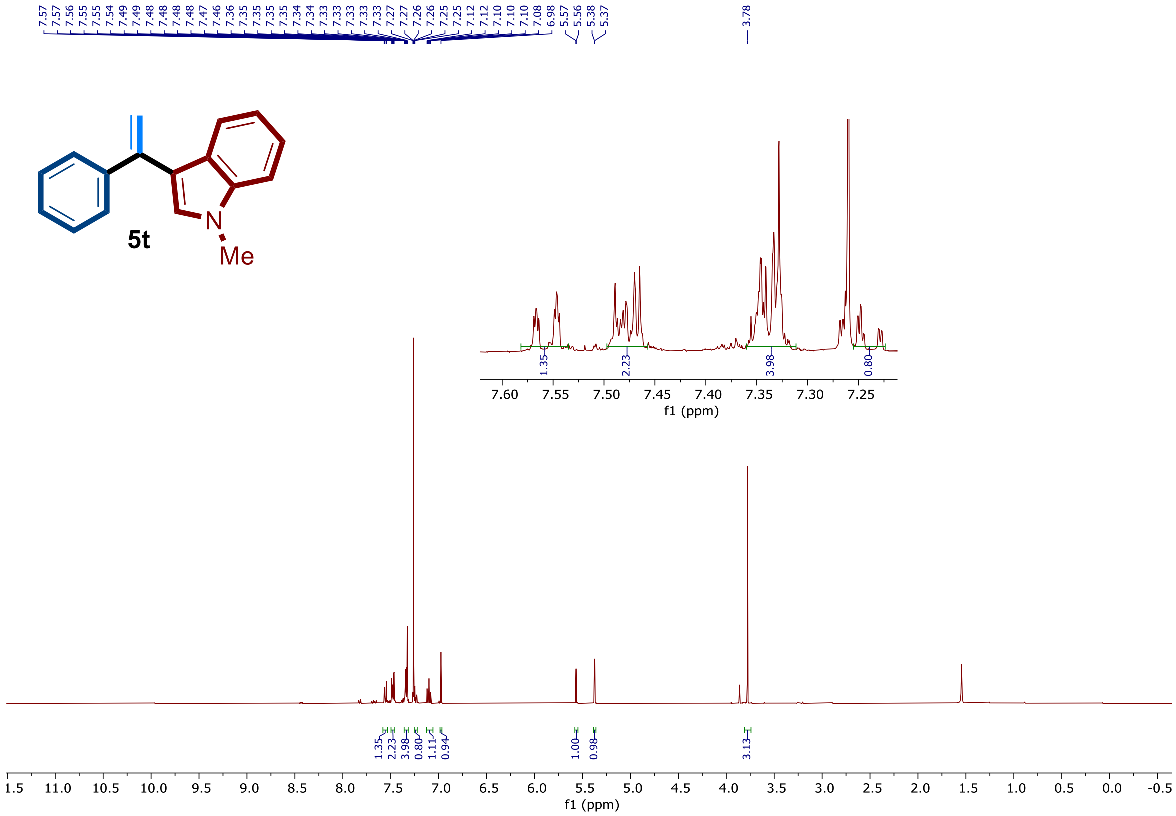

^{1}H NMR (400 MHz, $CDCl_3$) of compound (**5u**)

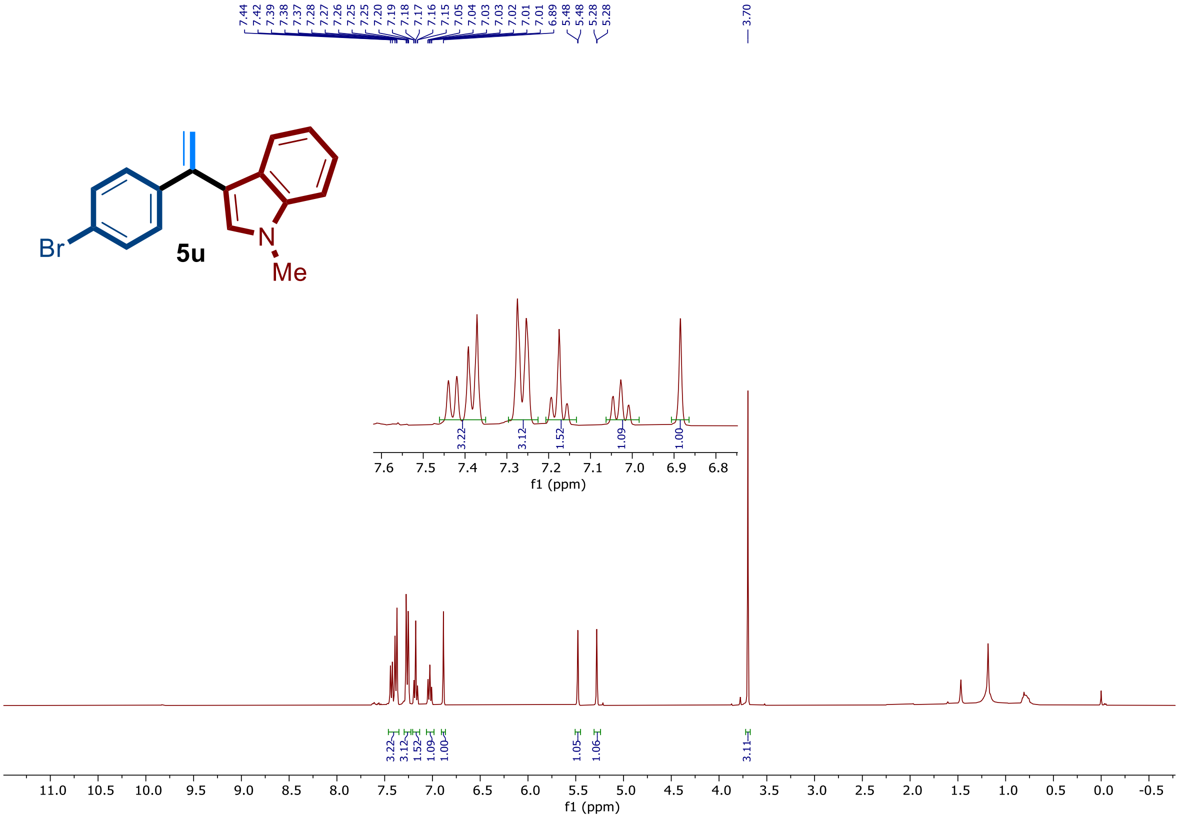

^{1}H NMR (400 MHz, $CDCl_3$) of compound (**5v**)

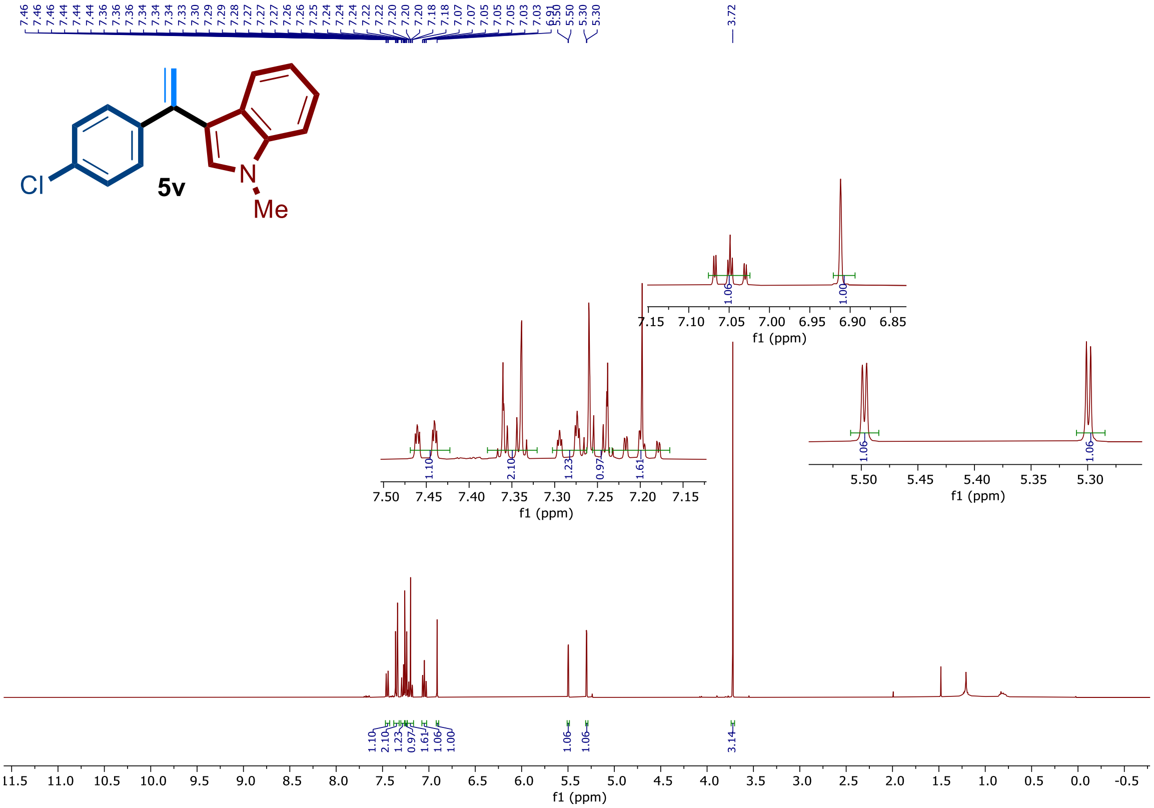

^{13}C{^{1}H} NMR (101 MHz, $CDCl_3$) of compound (**5v**)

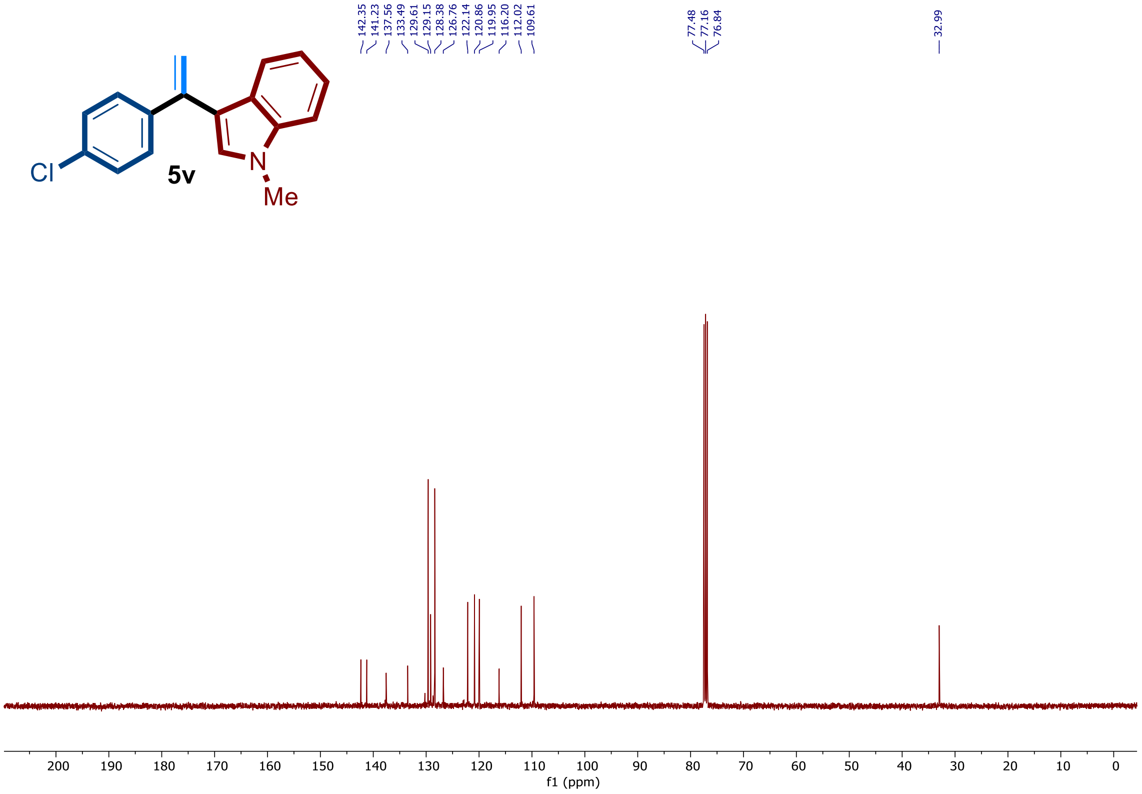

^{1}H NMR (400 MHz, $CDCl_3$) of compound (**5w**)

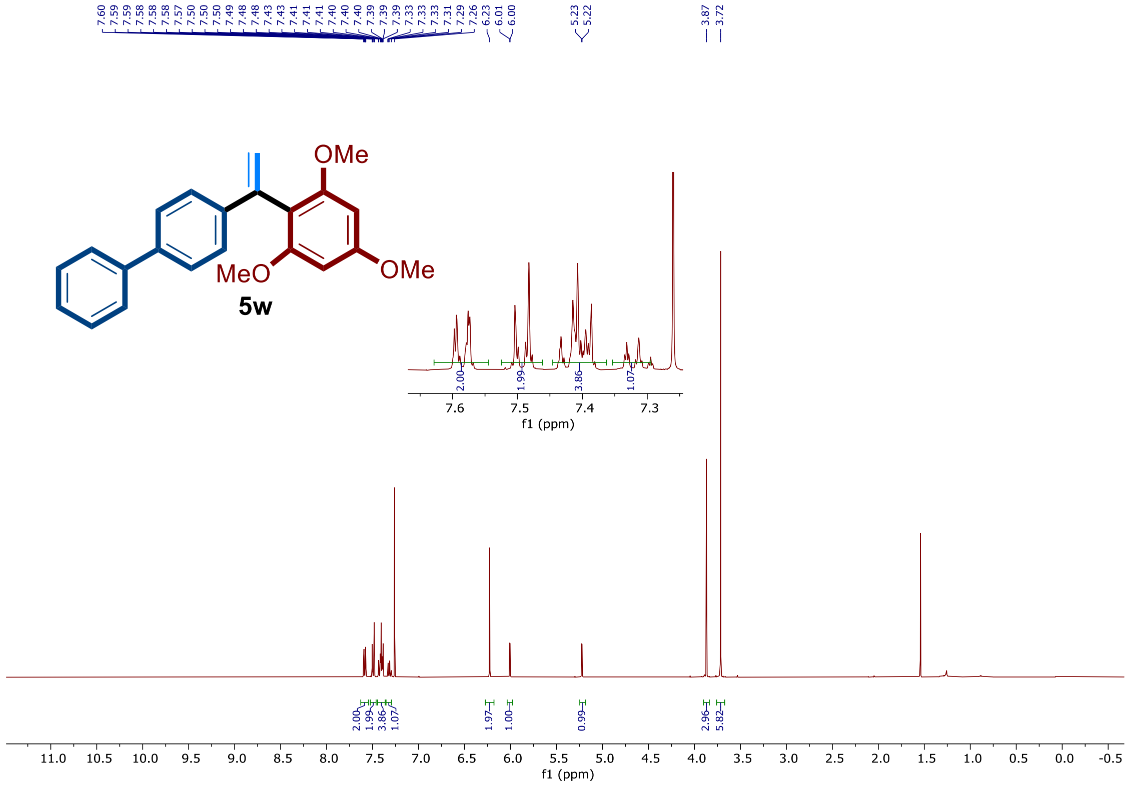

^{13}C{^{1}H} NMR (101 MHz, $CDCl_3$) of compound (**5w**)

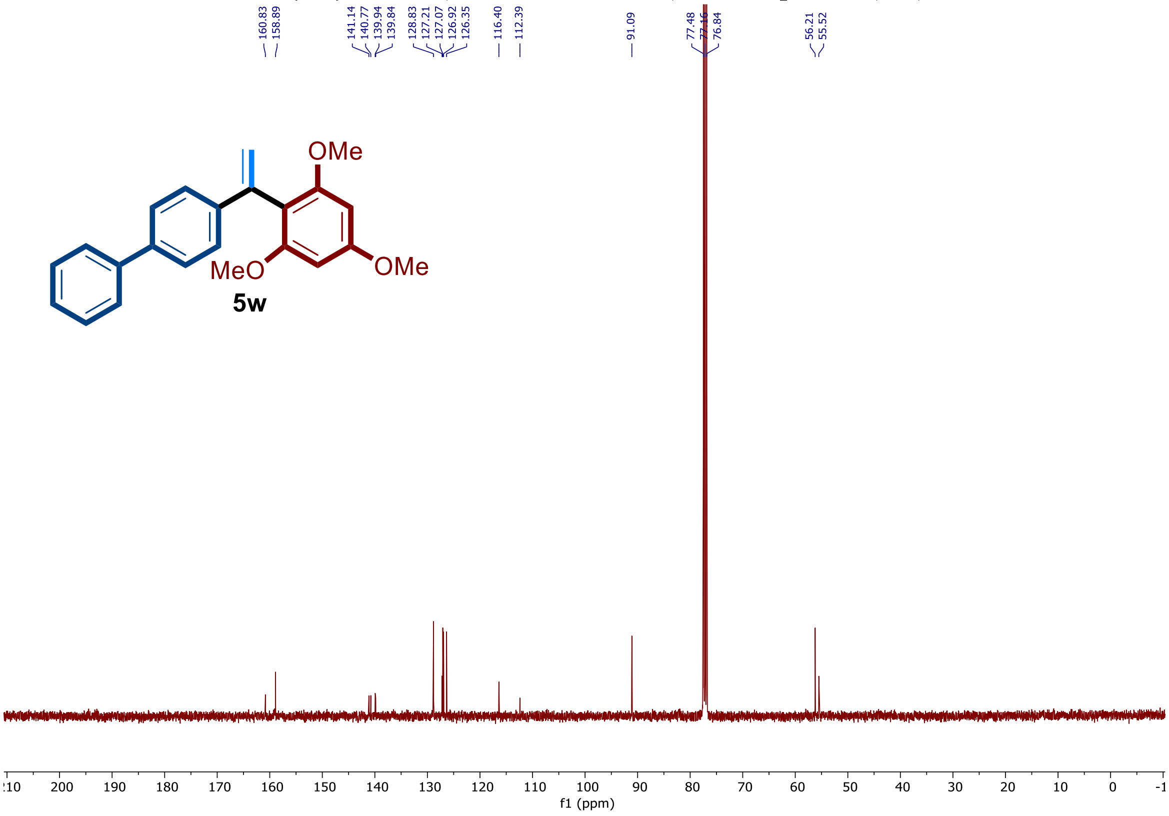

^{1}H NMR (400 MHz, $CDCl_3$) of compound (**5x**)

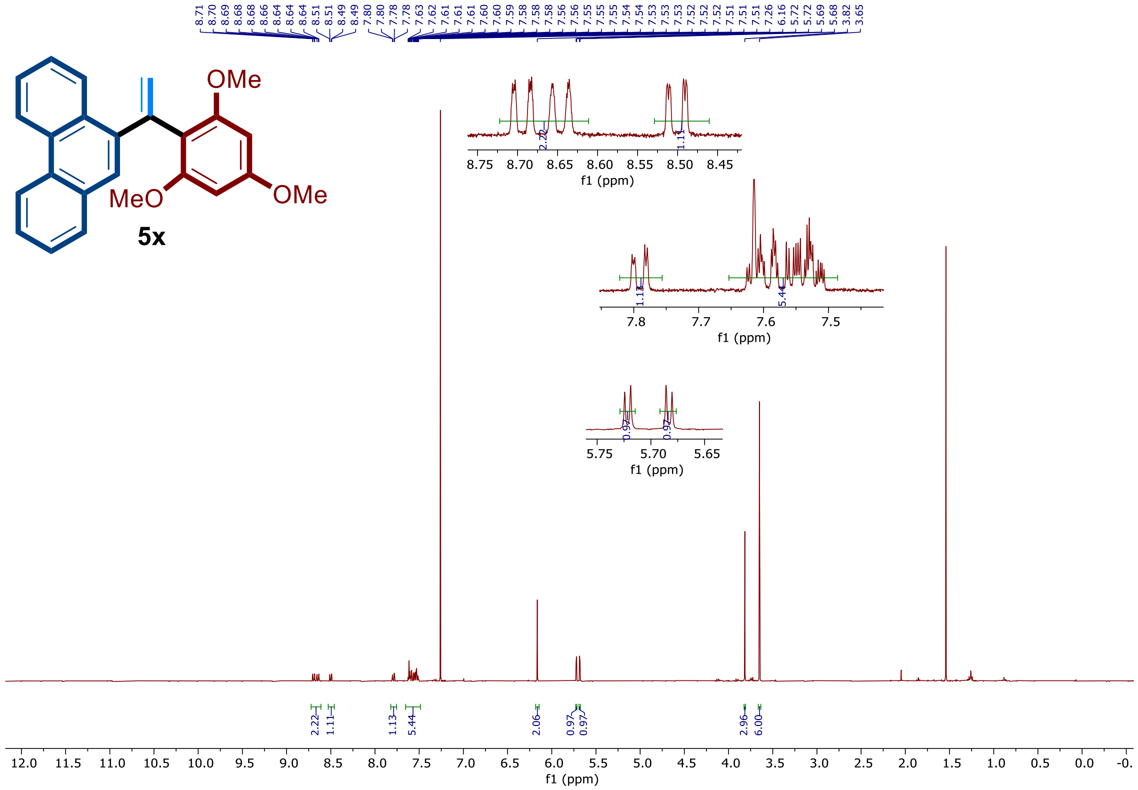

$^{13}C\{^{1}H\}$ NMR (101 MHz, $CDCl_3$) of compound (**5x**)

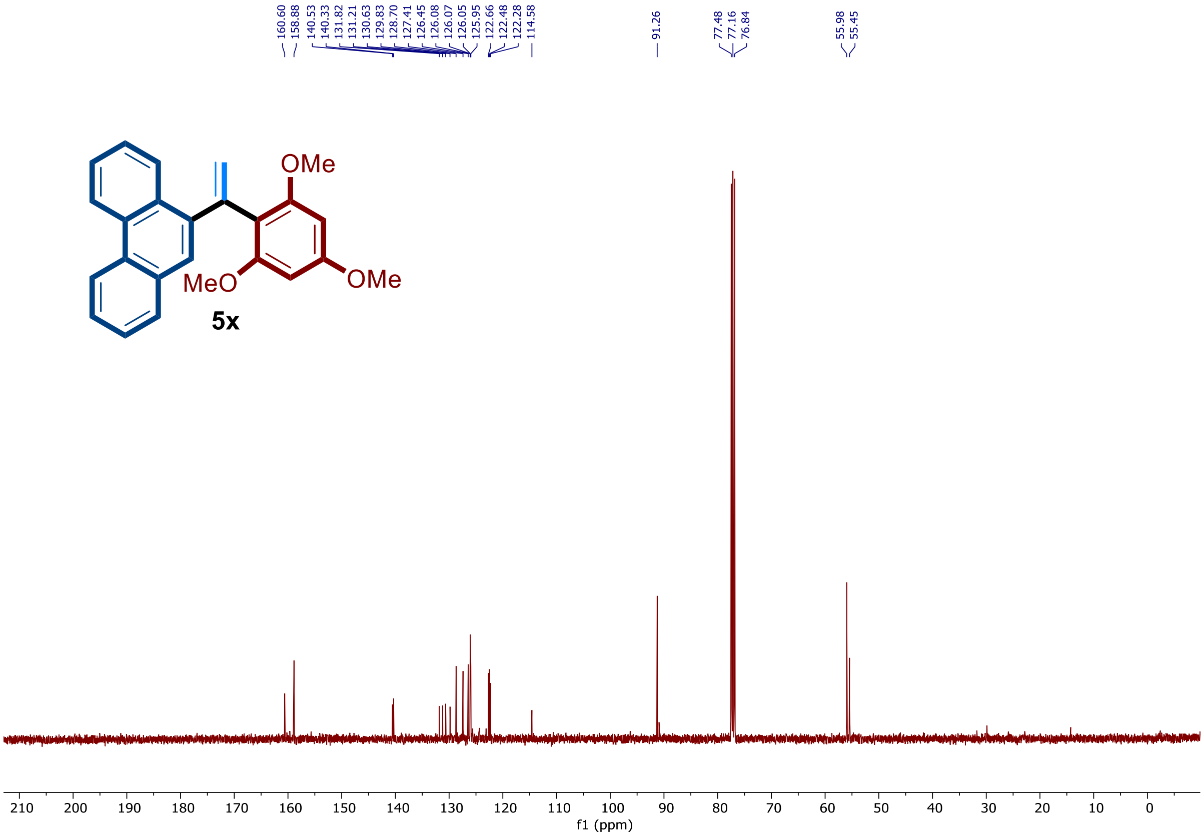

^{1}H NMR (400 MHz, $CDCl_3$) of compound (**5y**)

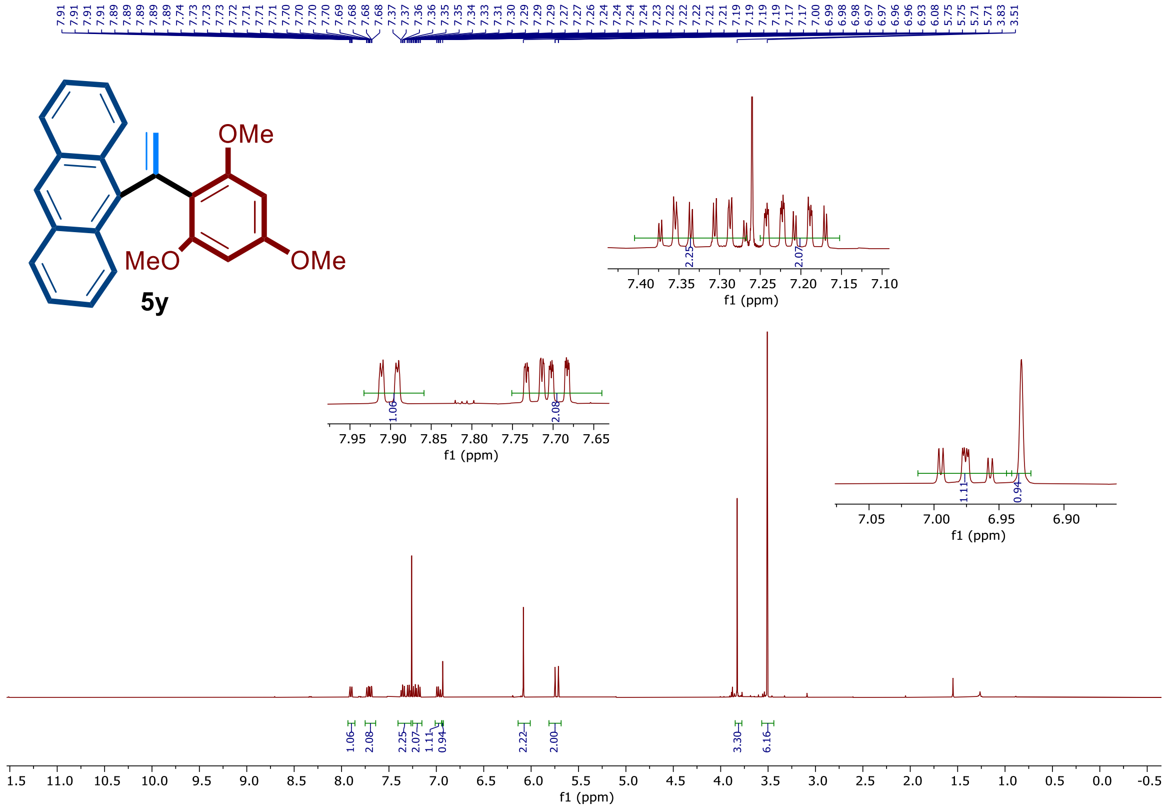

$^{13}C\{^{1}H\}$ NMR (101 MHz, $CDCl_3$) of compound (**5y**)

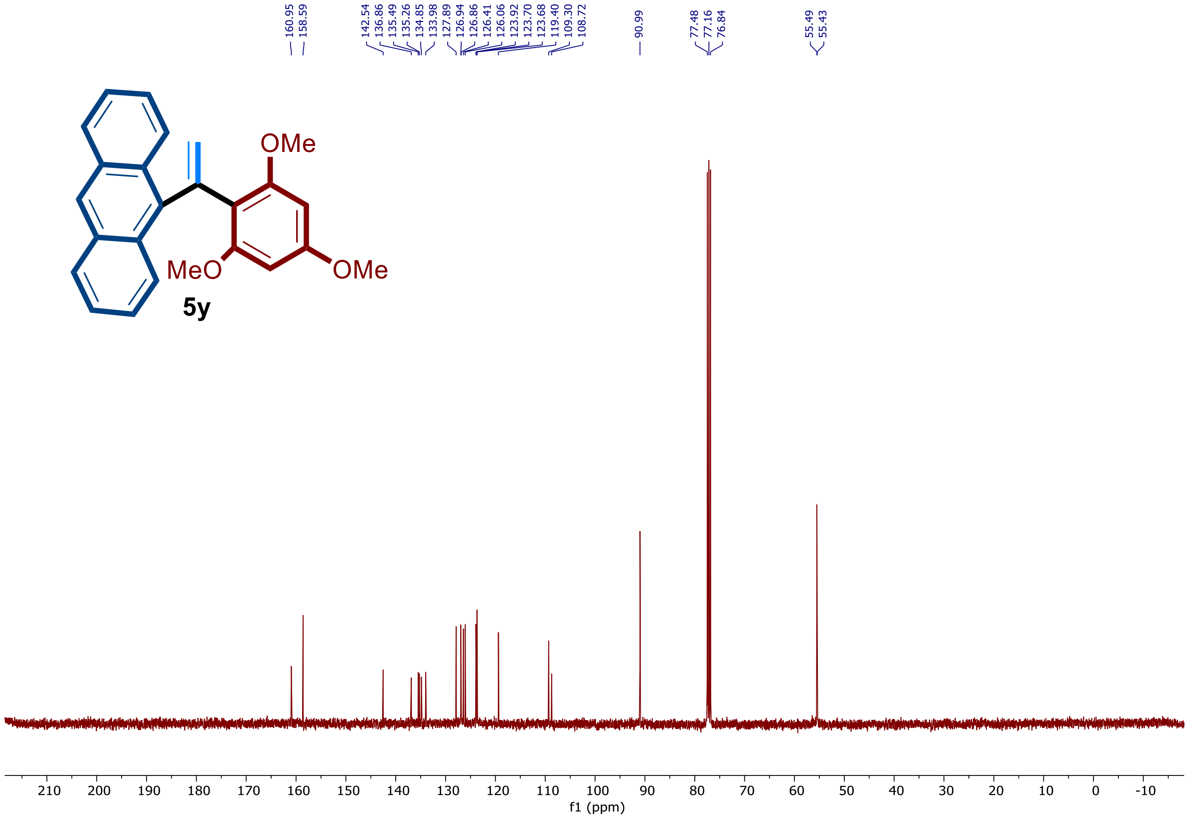

^{1}H NMR (400 MHz, $CDCl_3$) of compound (**5z**)

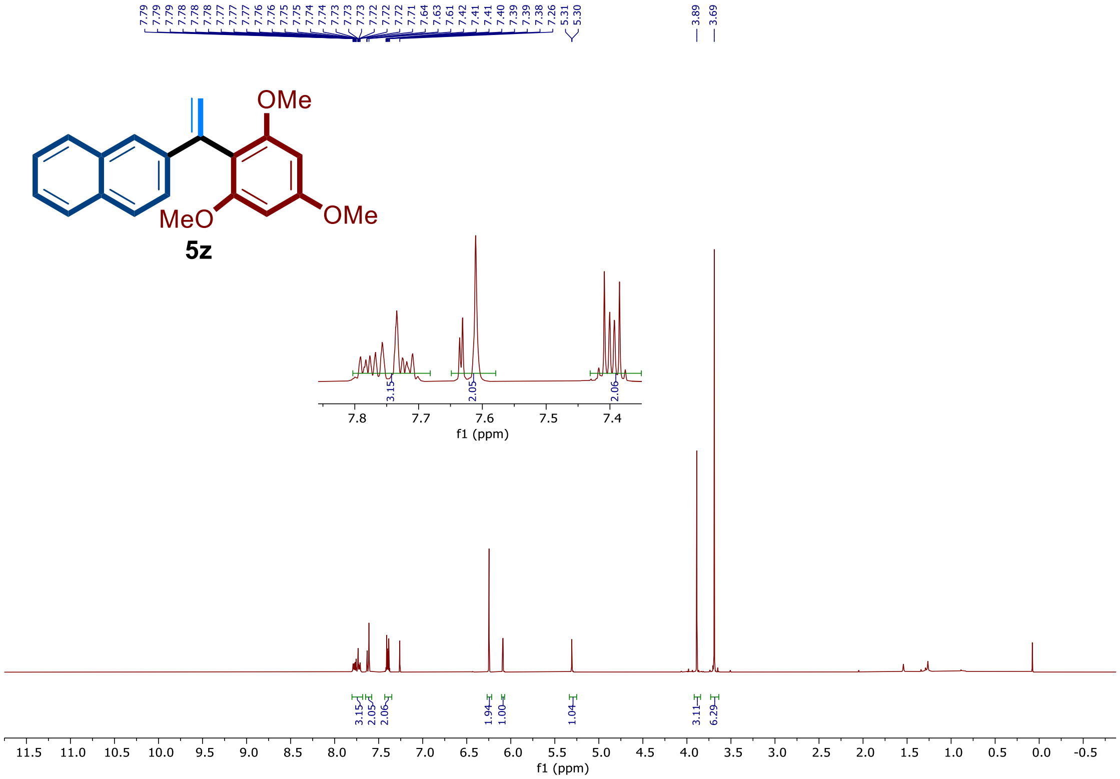

^{1}H NMR (400 MHz, $CDCl_3$) of compound (**5aa**)

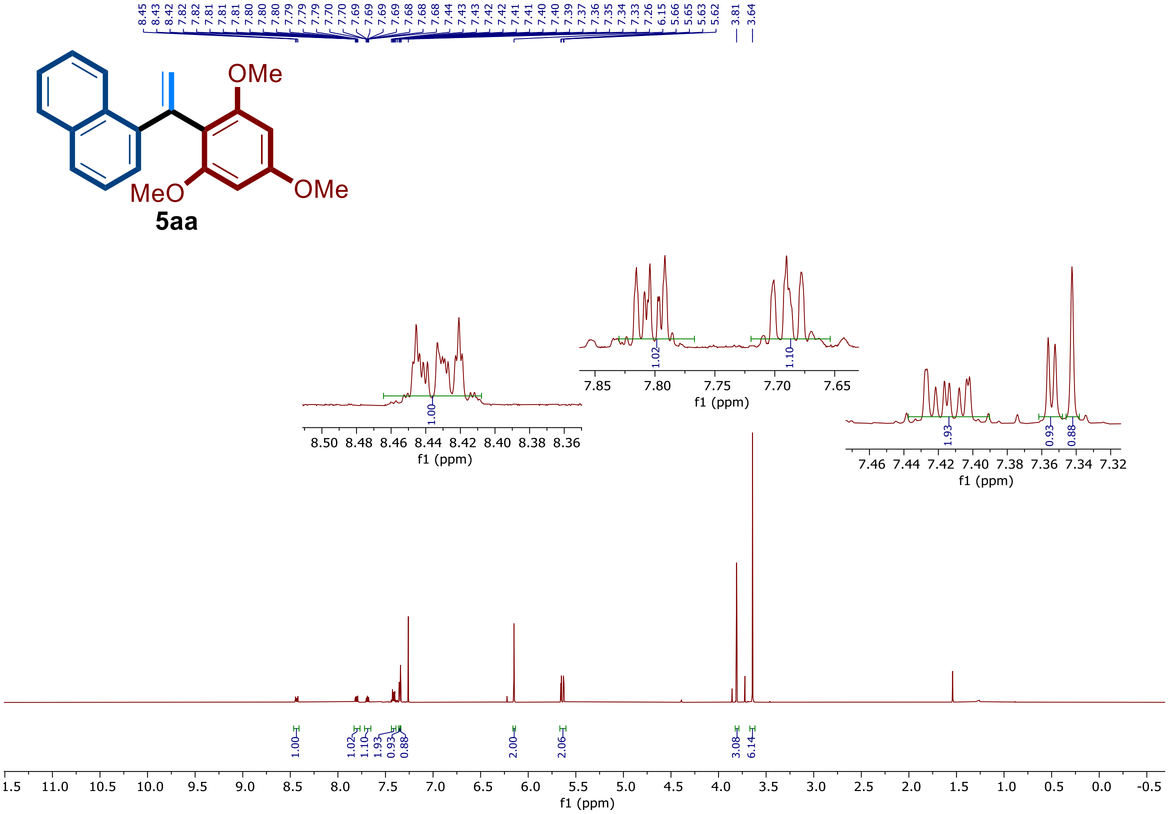

$^{13}C\{^1H\}$ NMR (101 MHz, $CDCl_3$) of compound (**5aa**)

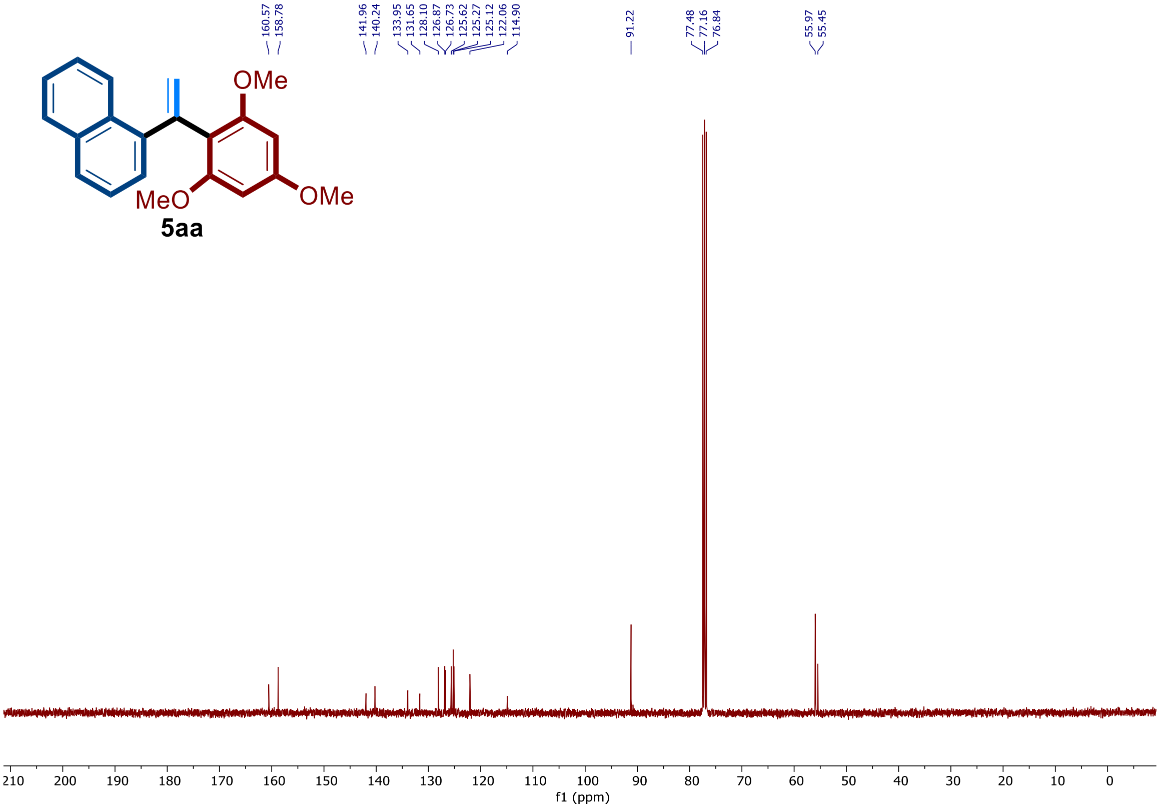

^1H NMR (400 MHz, $CDCl_3$) of compound (**5ab**)

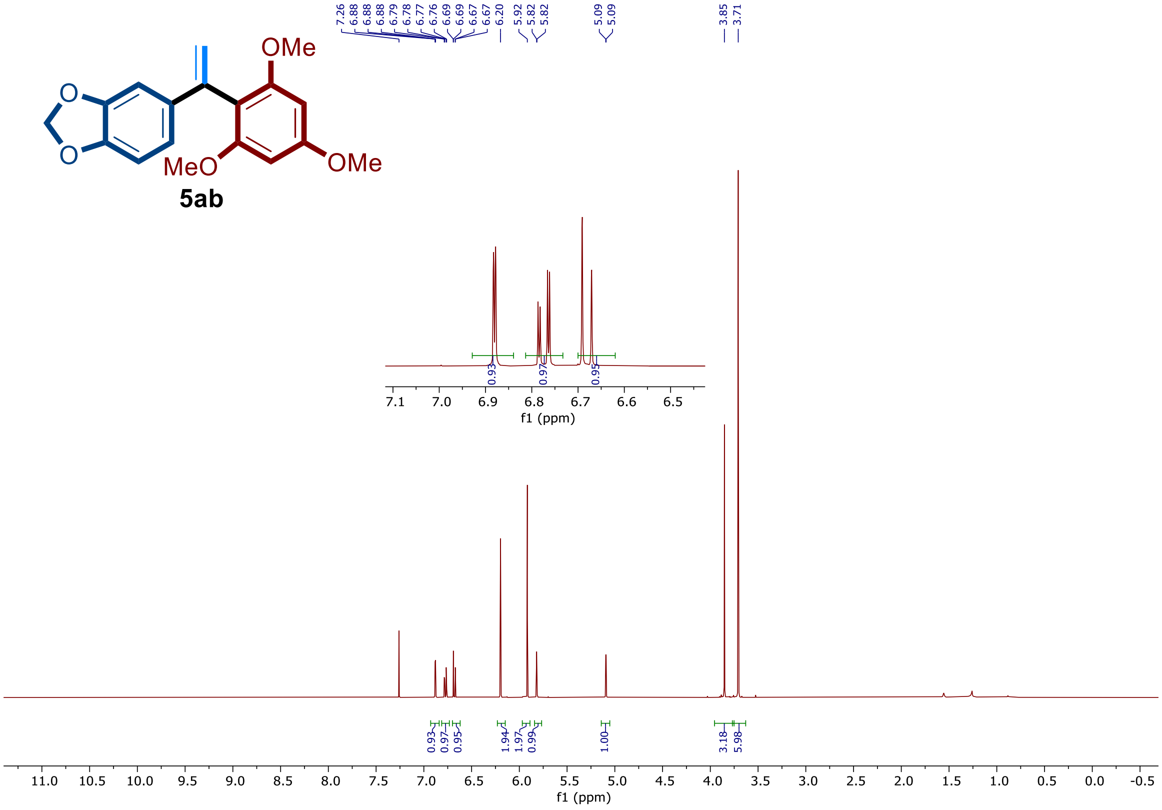

$^{13}C\{^{1}H\}$ NMR (101 MHz, $CDCl_3$) of compound (**5ab**)

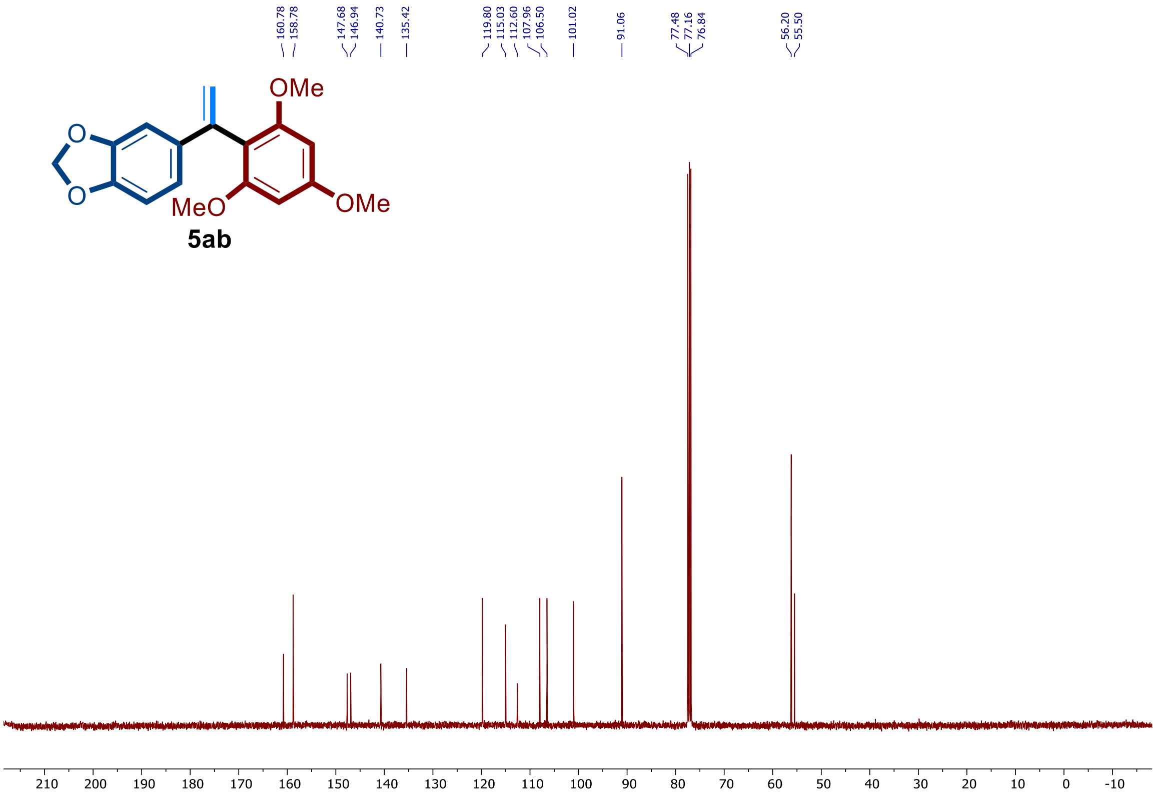

^{1}H NMR (400 MHz, $CDCl_3$) of compound (**5ac**)

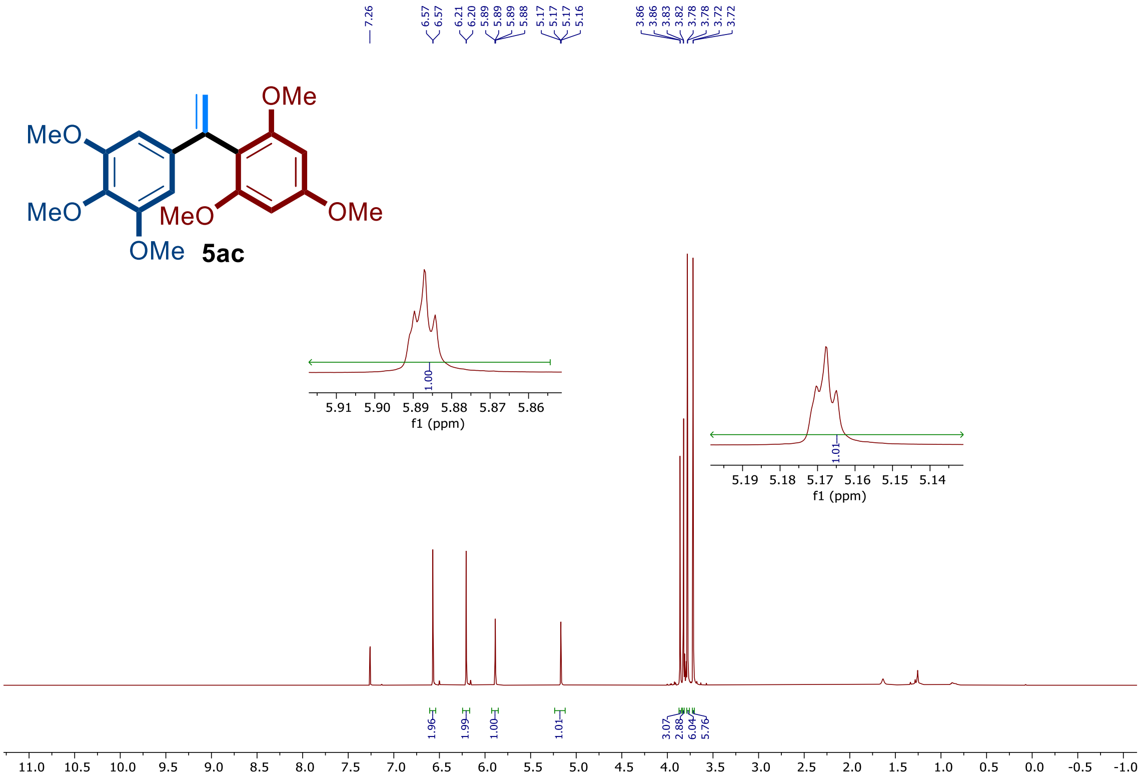

$^{13}C\{^1H\}$ NMR (101 MHz, $CDCl_3$) of compound (**5ac**)

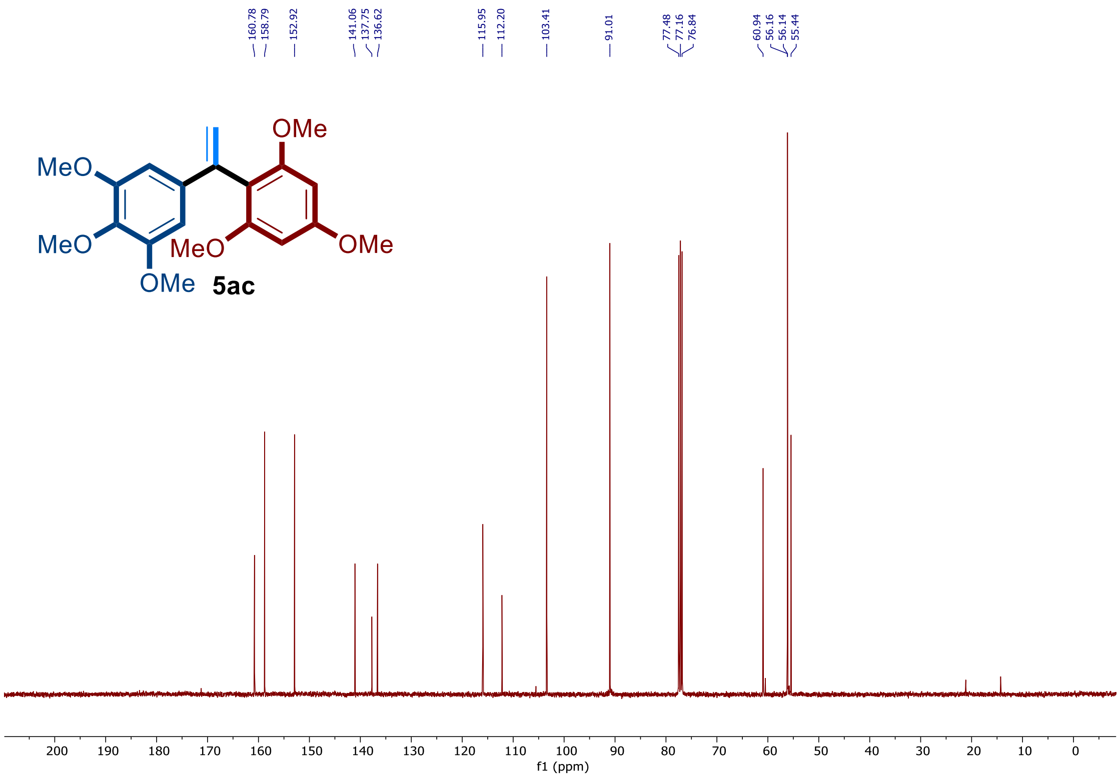

^1H NMR (400 MHz, $CDCl_3$) of compound (**5ad**)

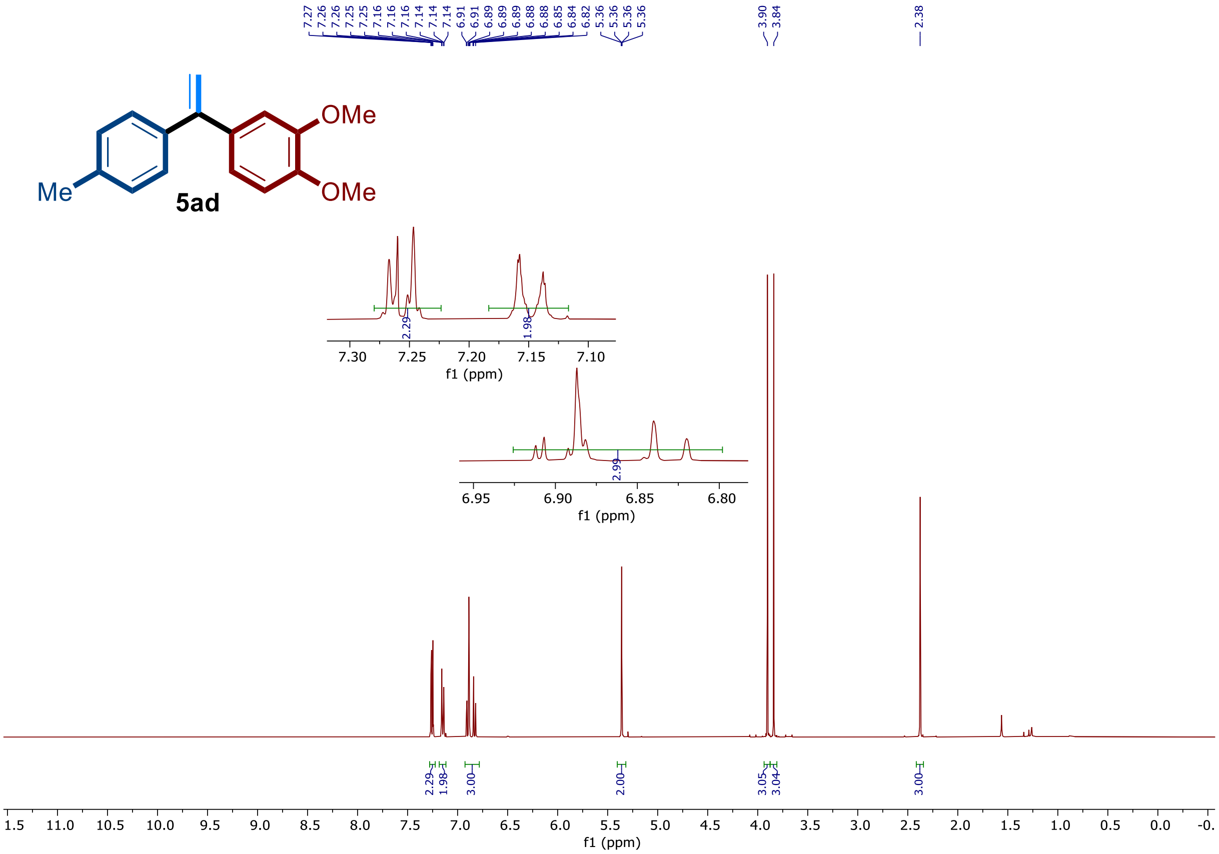

^{1}H NMR (400 MHz, $CDCl_3$) of compound (**5ae**)

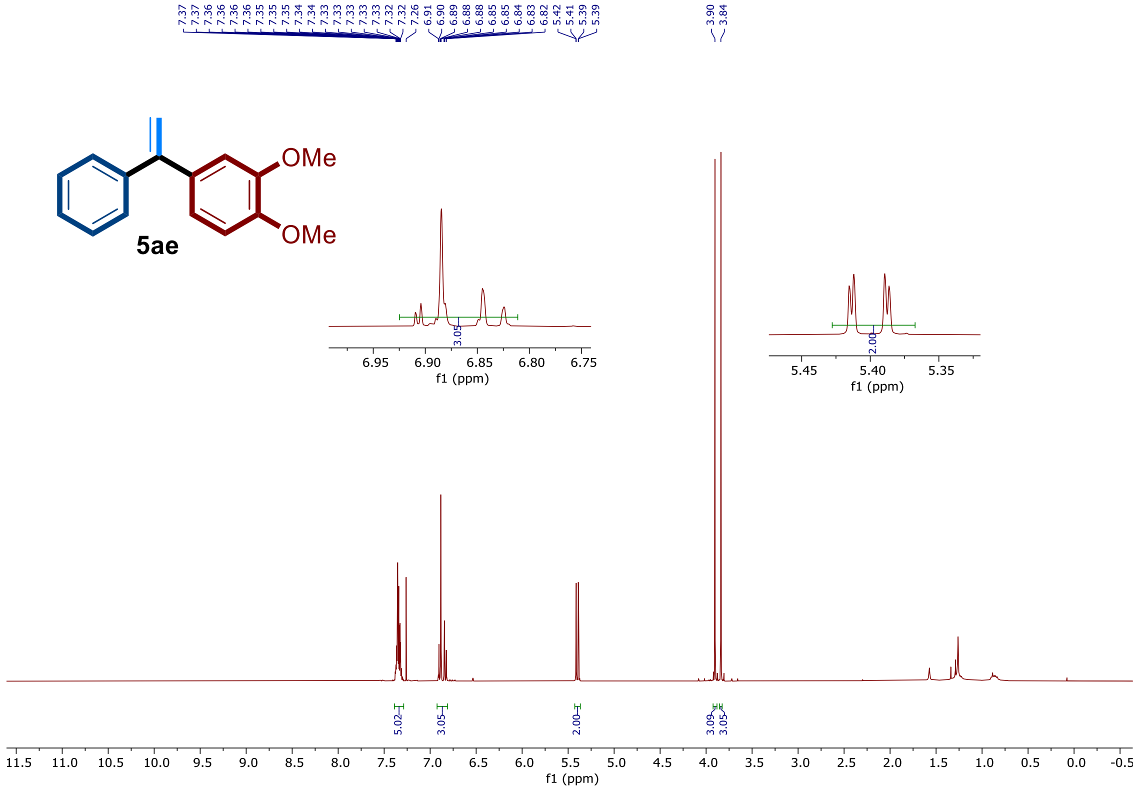

^{1}H NMR (400 MHz, $CDCl_3$) of compound (**5af**)

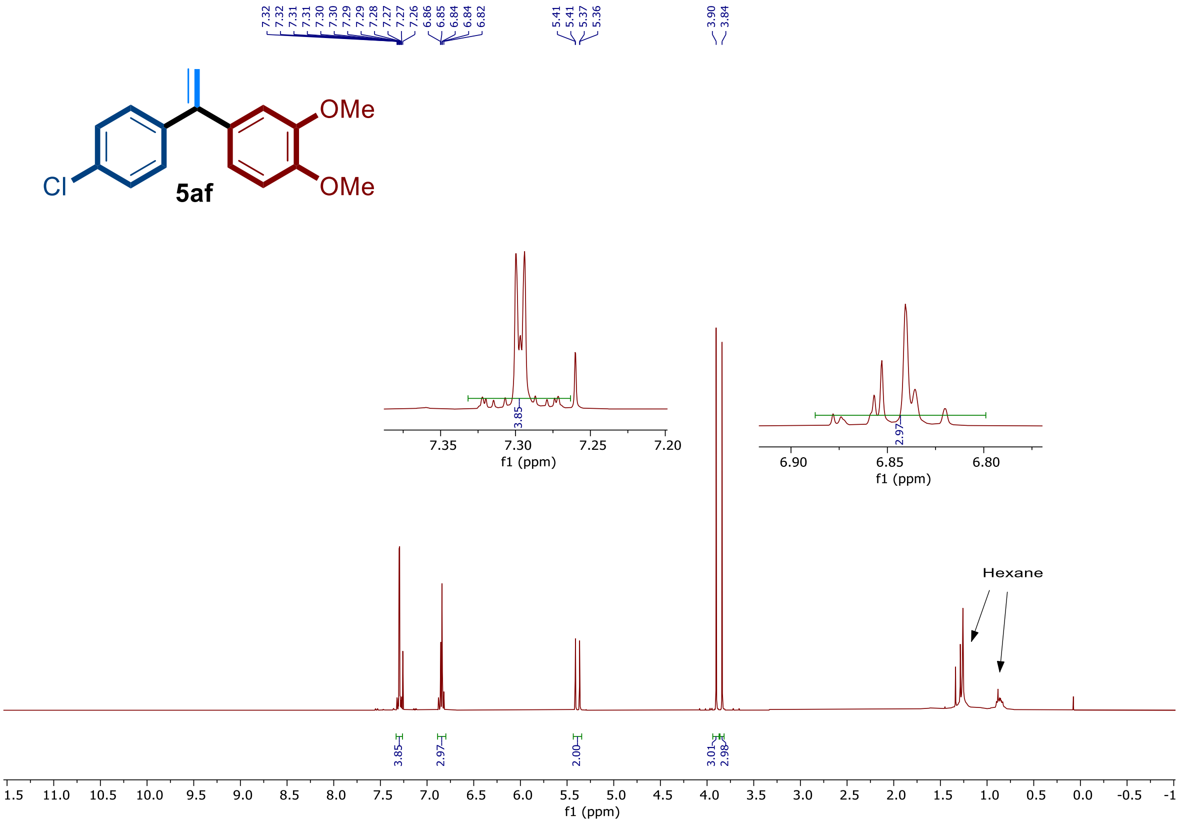

$^{13}C\{^{1}H\}$ NMR (101 MHz, $CDCl_3$) of compound (**5af**)

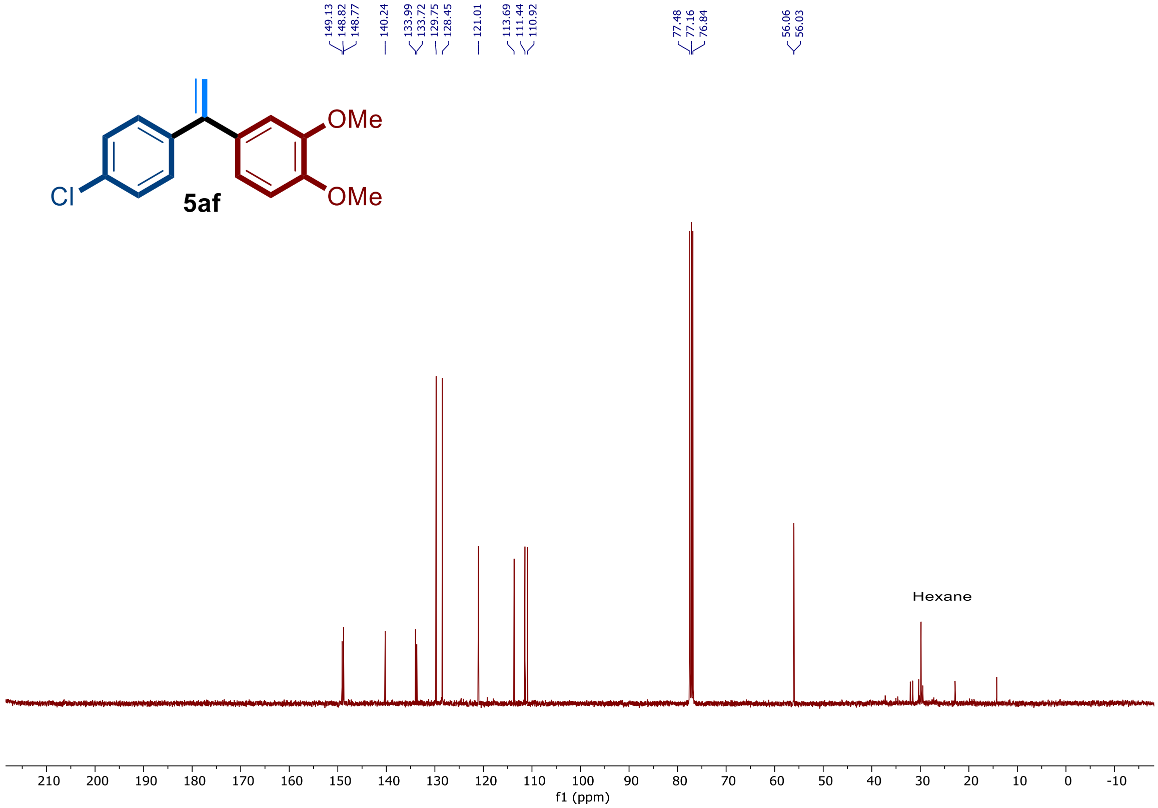

^{1}H NMR (400 MHz, $CDCl_3$) of compound (**5ag**)

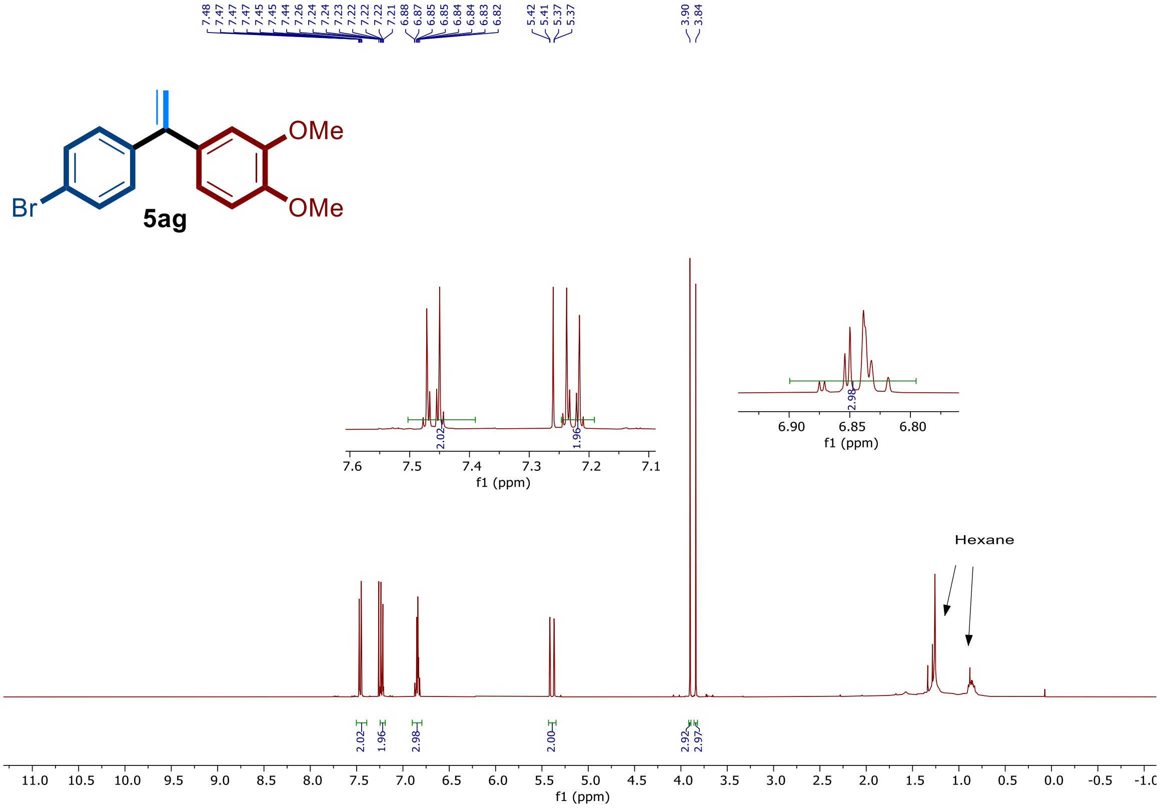

^{1}H NMR (400 MHz, $CDCl_3$) of compound (**5ah**)

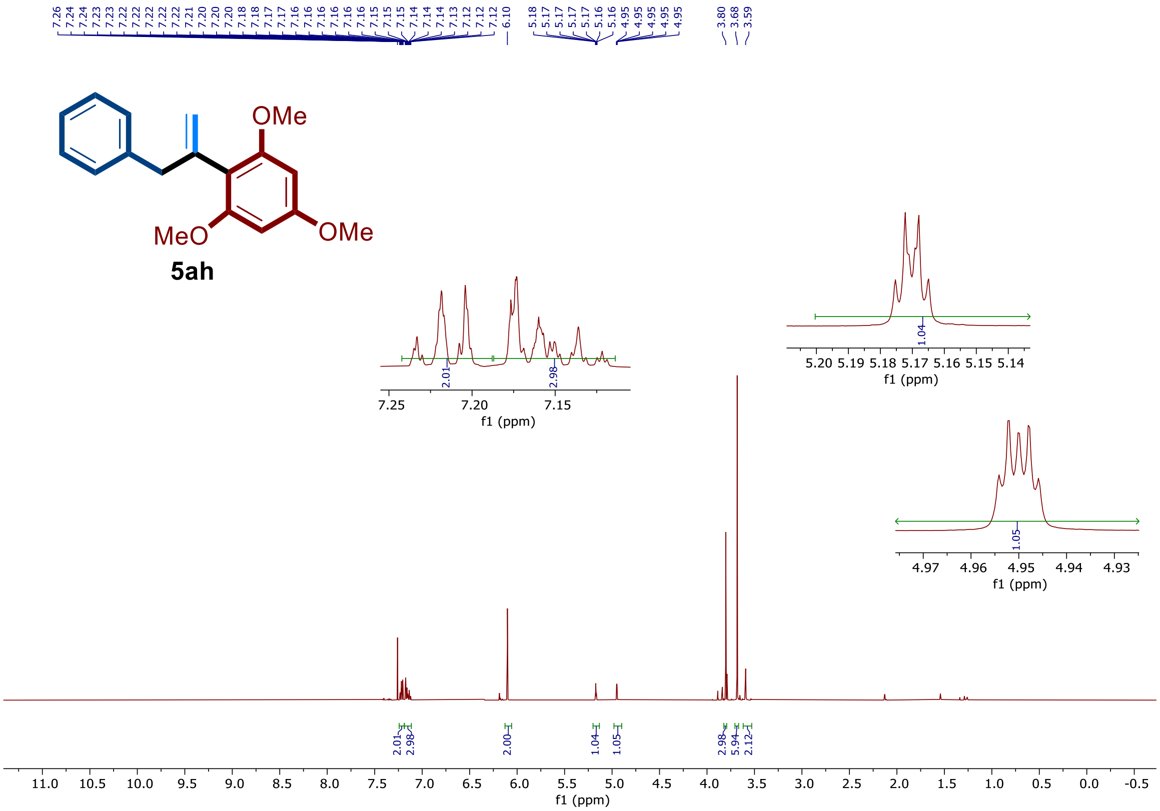

^{13}C{^{1}H} NMR (101 MHz, $CDCl_3$) of compound (**5ah**)

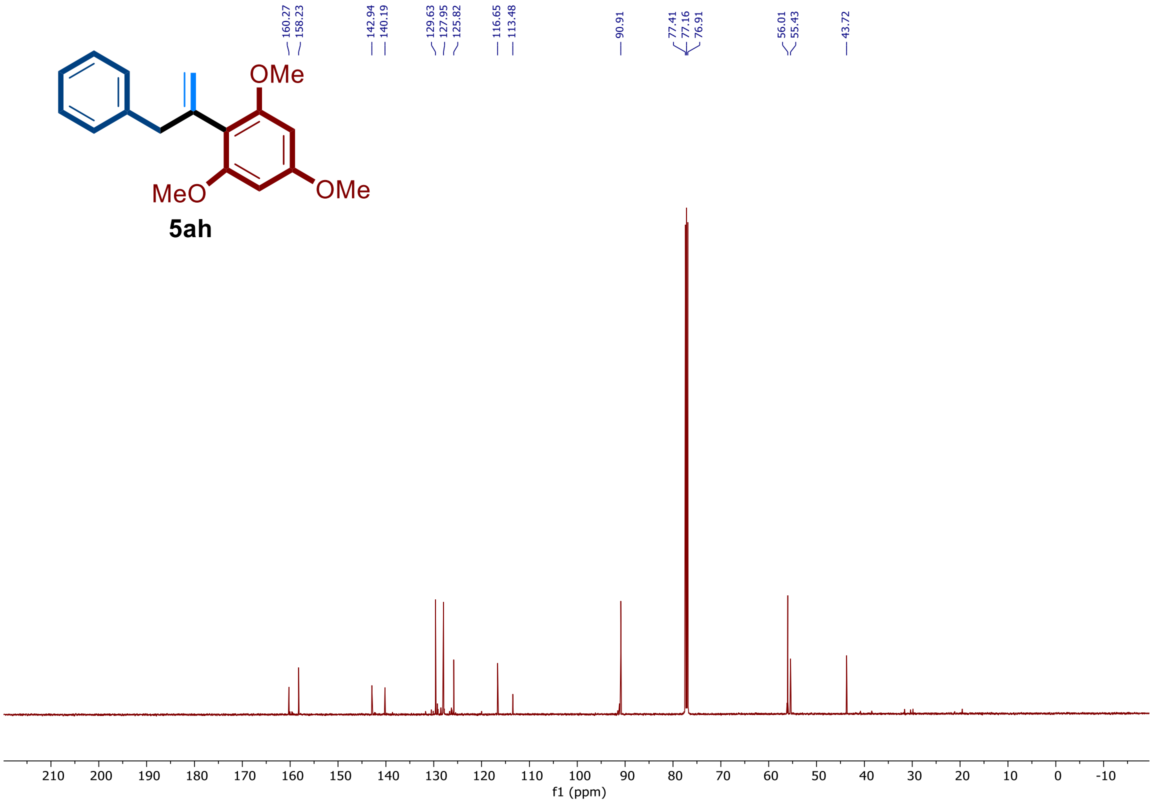

^{1}H NMR (400 MHz, $CDCl_3$) of compound (**5ai**)

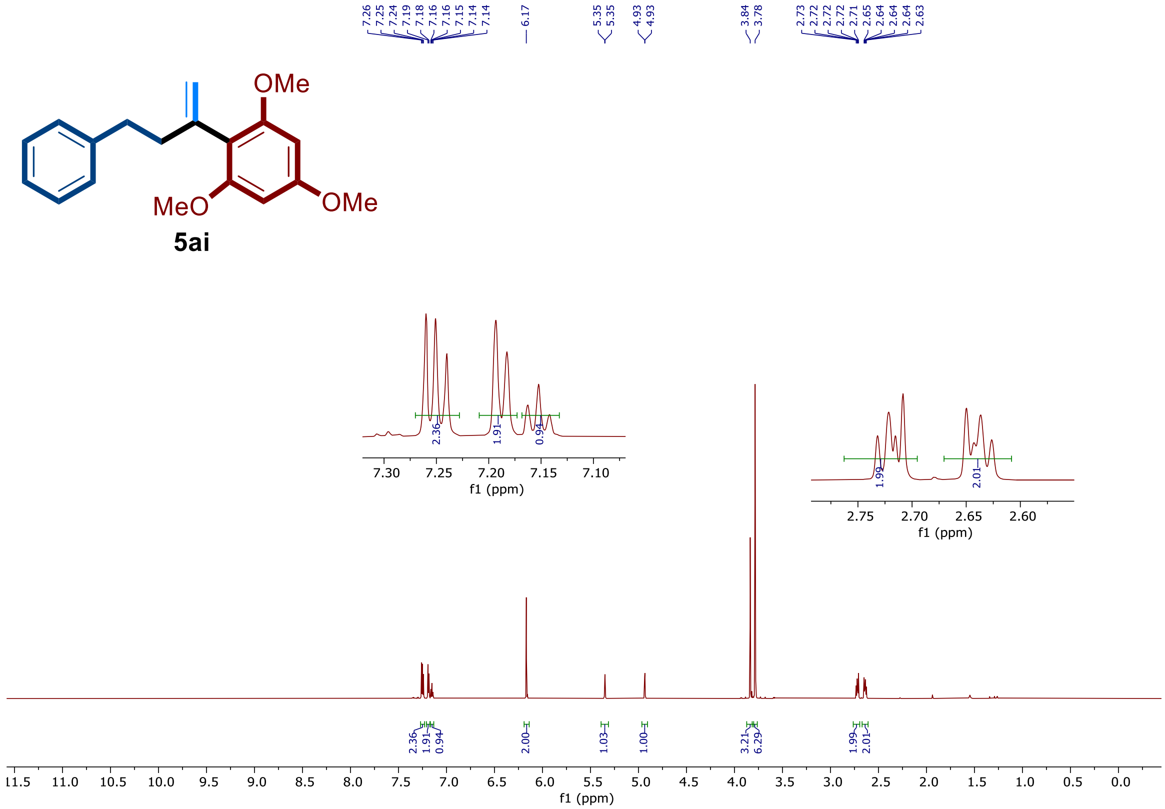

^{13}C{^{1}H} NMR (101 MHz, $CDCl_3$) of compound (**5ai**)

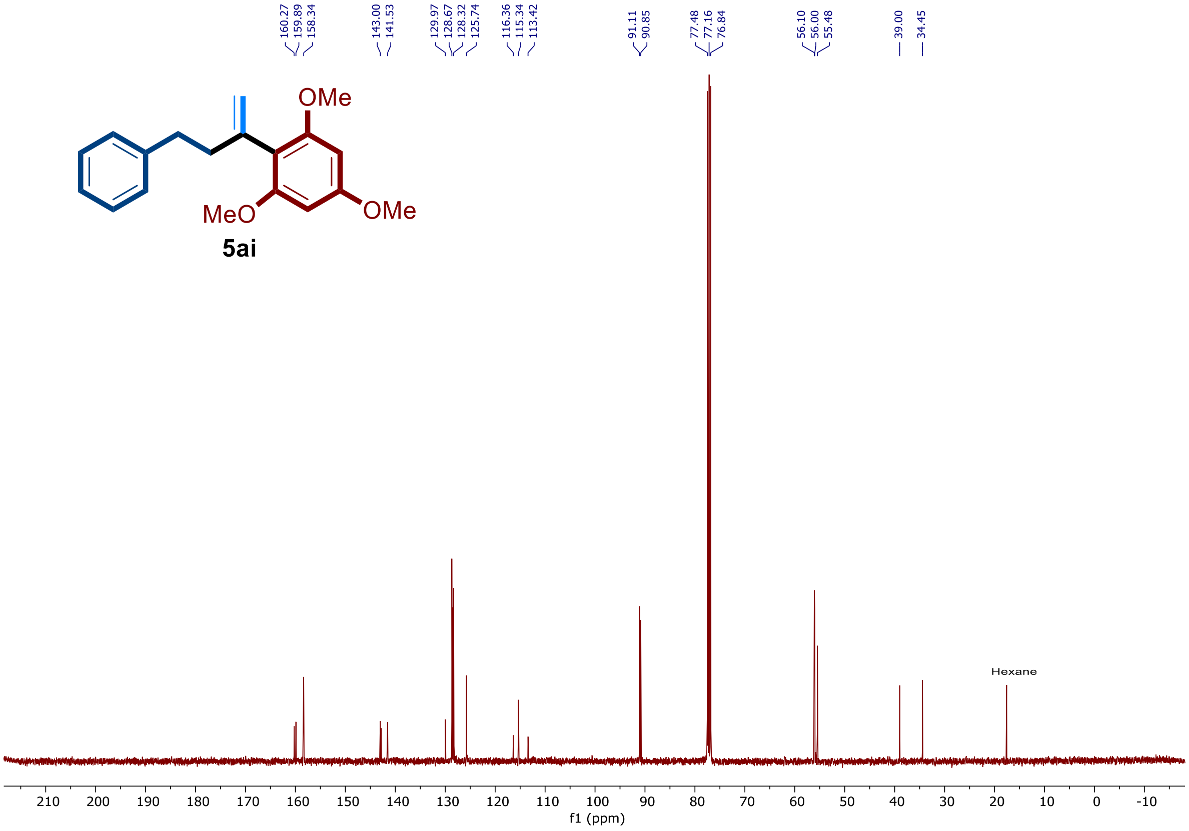

^{1}H NMR (400 MHz, $CDCl_3$) of compound (**5aj**)

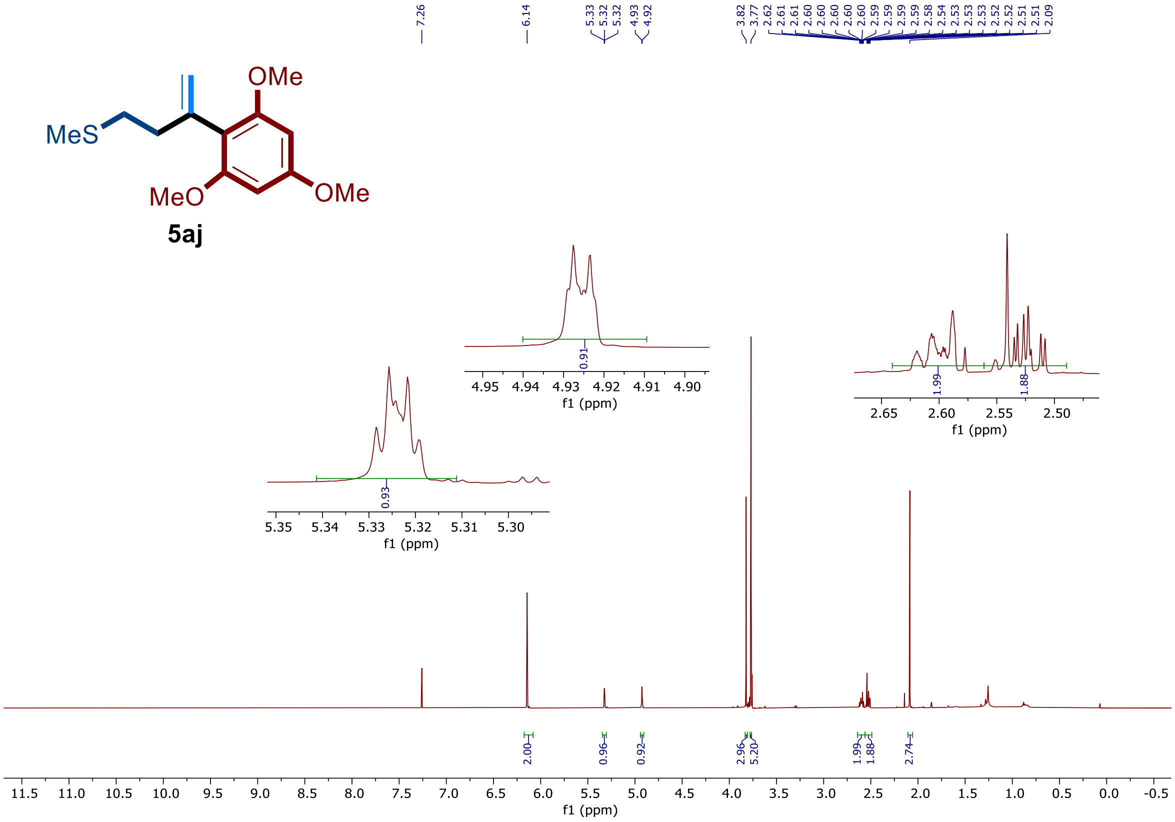

^{13}C{^{1}H} NMR (101 MHz, $CDCl_3$) of compound (**5aj**)

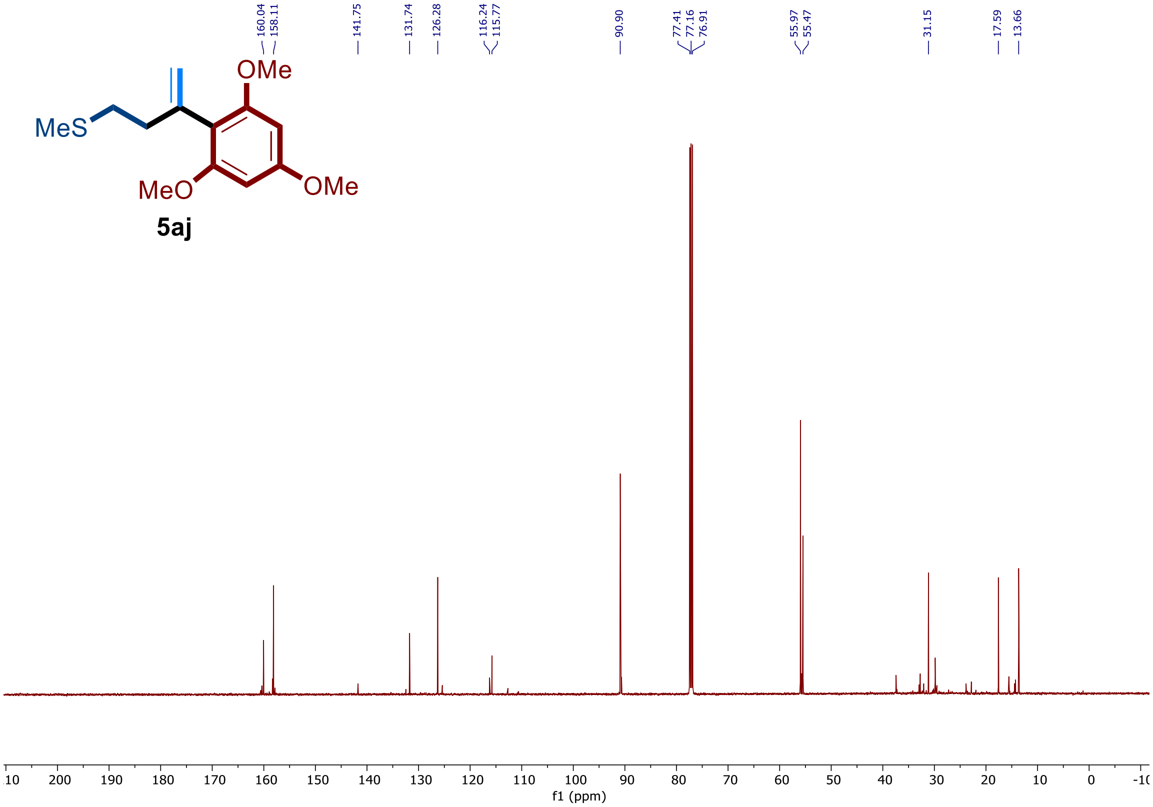

^{1}H NMR (400 MHz, $CDCl_3$) of compound (**5ak**)

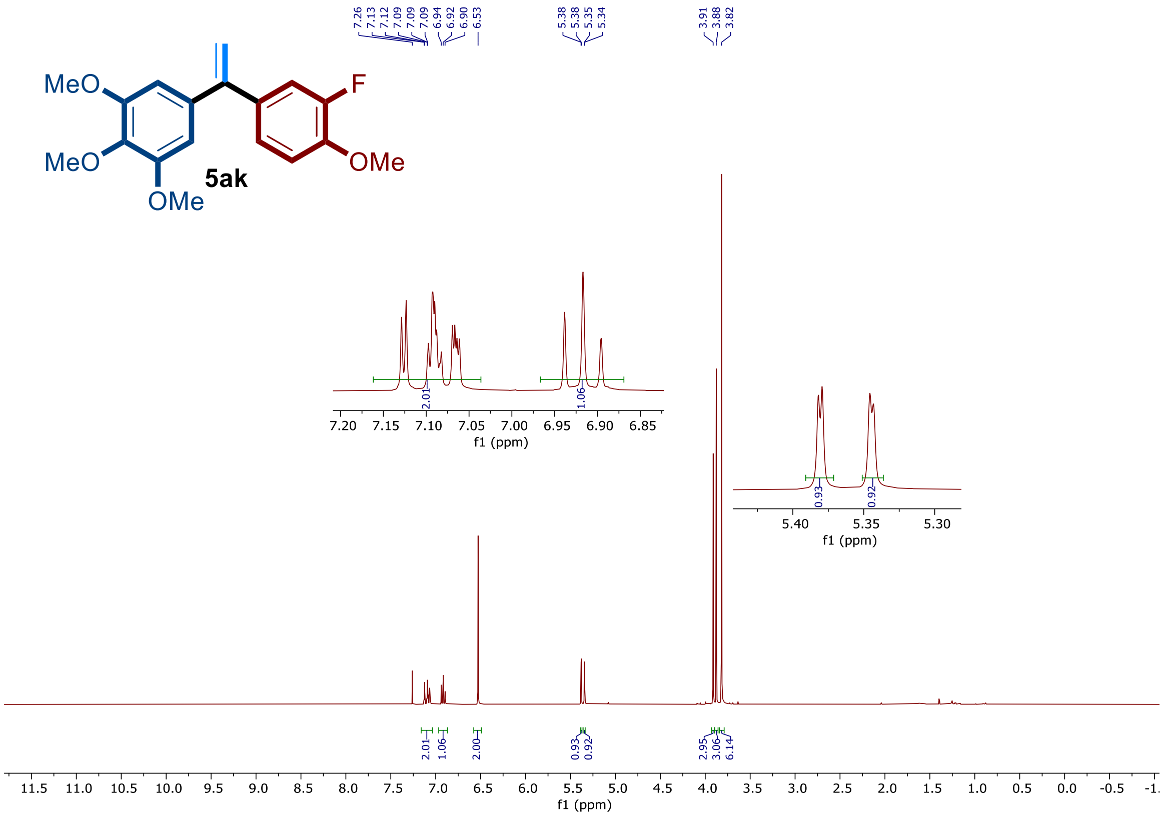

^{13}C{^{1}H} NMR (101 MHz, $CDCl_3$) of compound (**5ak**)

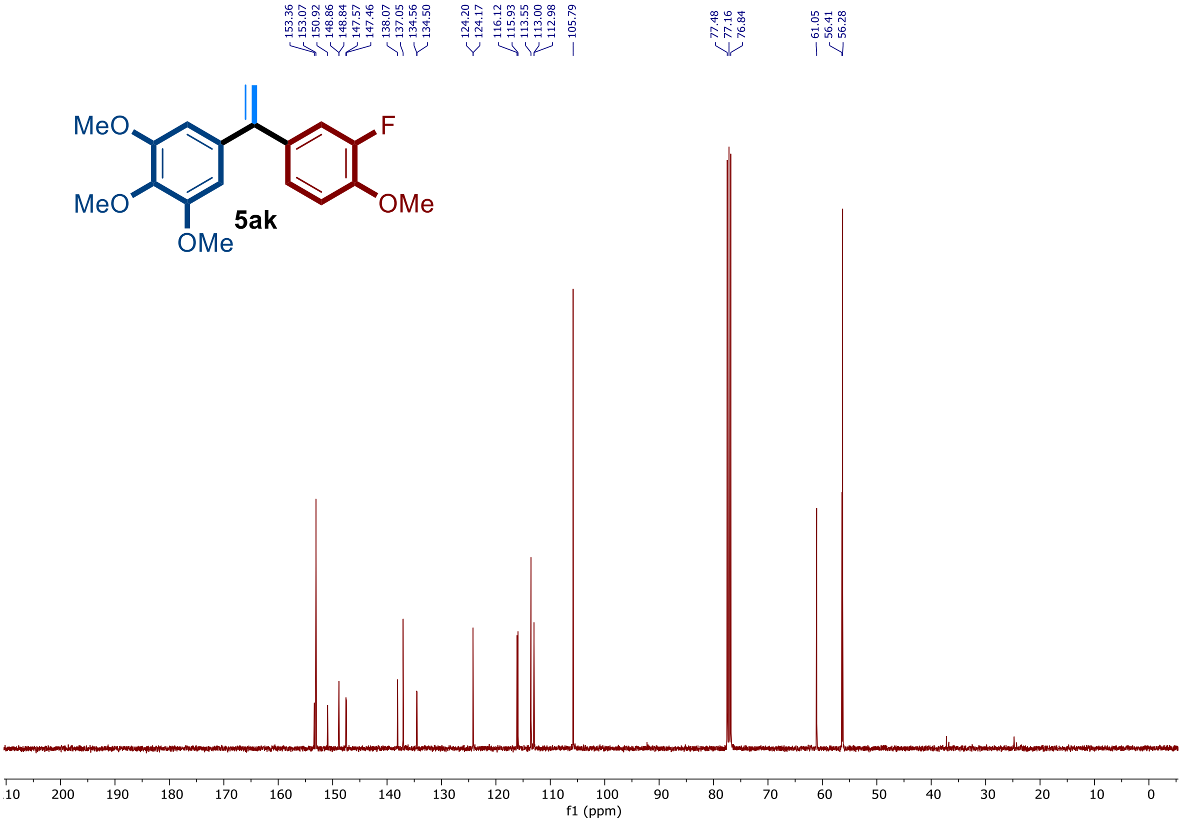

^{19}F NMR (376 MHz, CDCl$_3$) of compound (**5ak**)

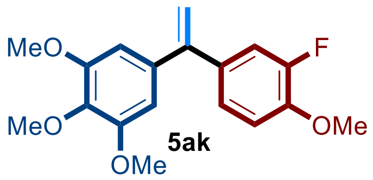

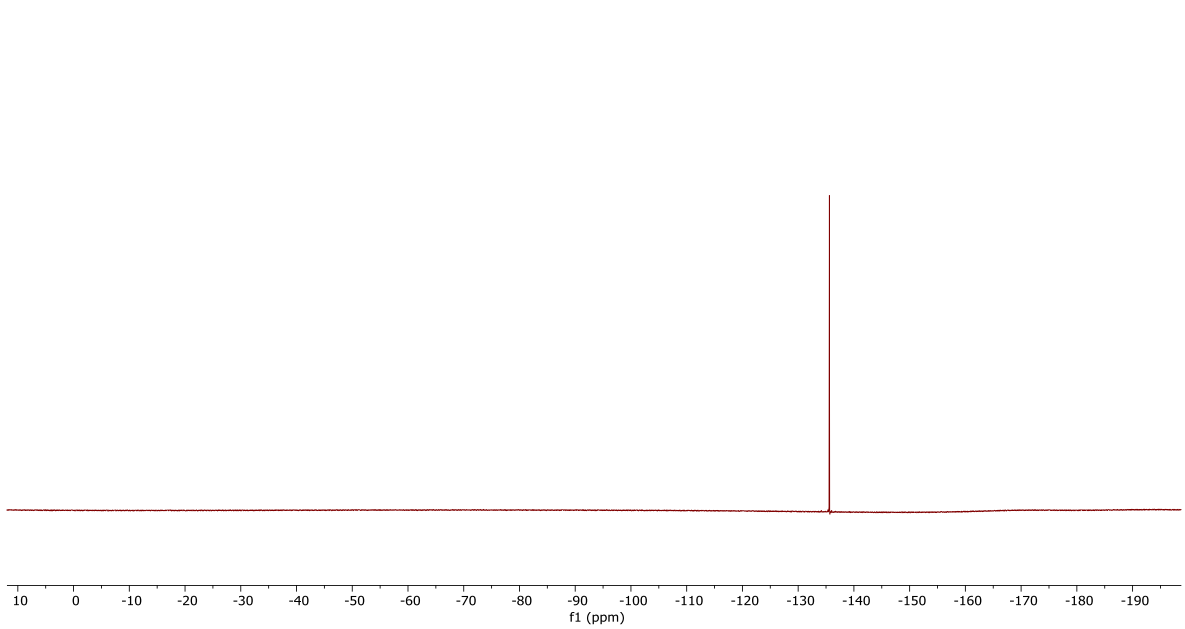

^{1}H NMR (400 MHz, CDCl$_3$) of compound (**6a**)

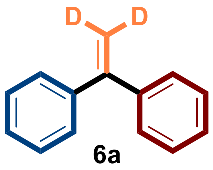

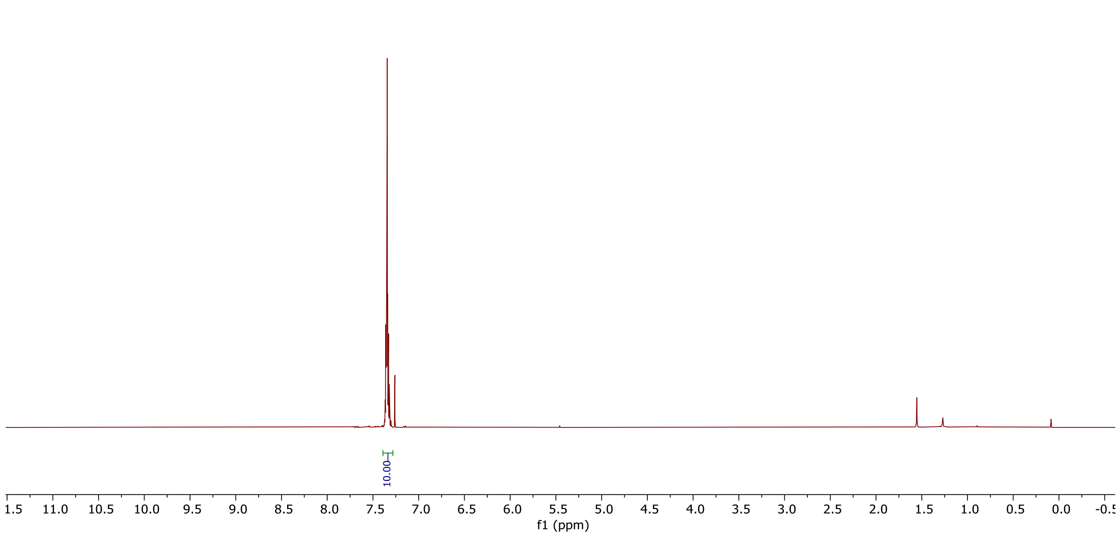

^{1}H NMR (400 MHz, $CDCl_3$) of compound (**6b**)

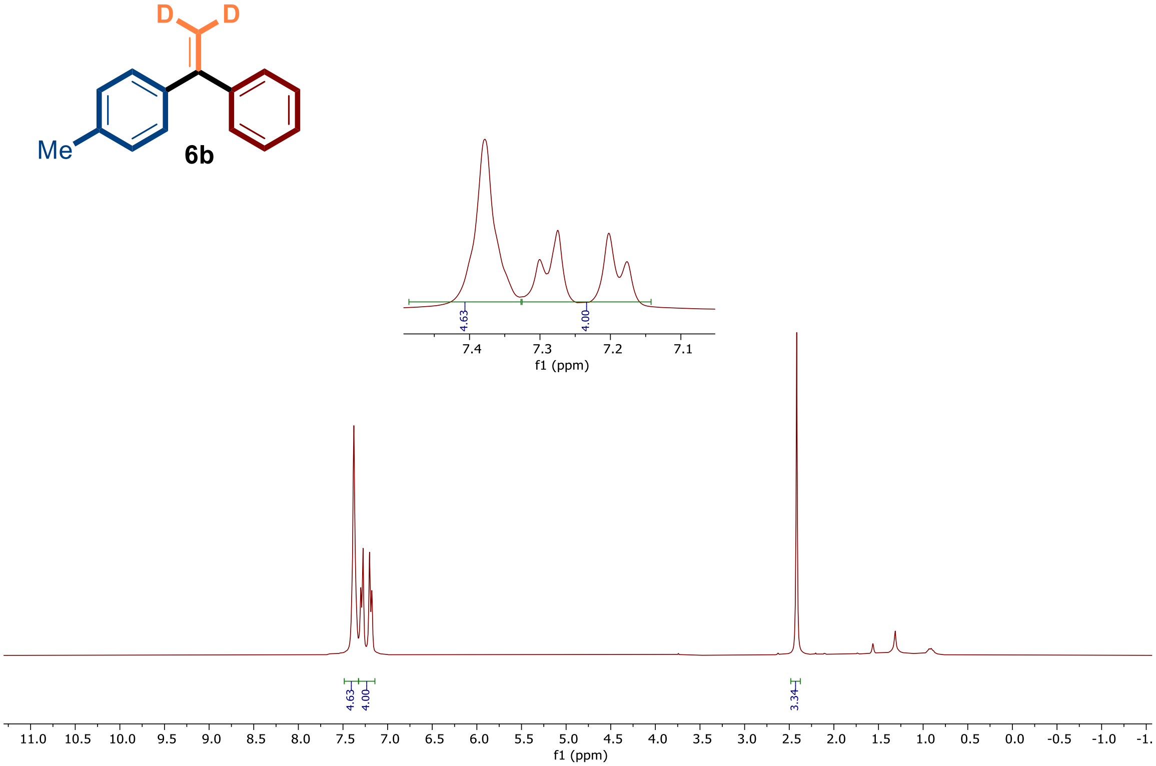

^{1}H NMR (400 MHz, $CDCl_3$) of compound (**6c**)

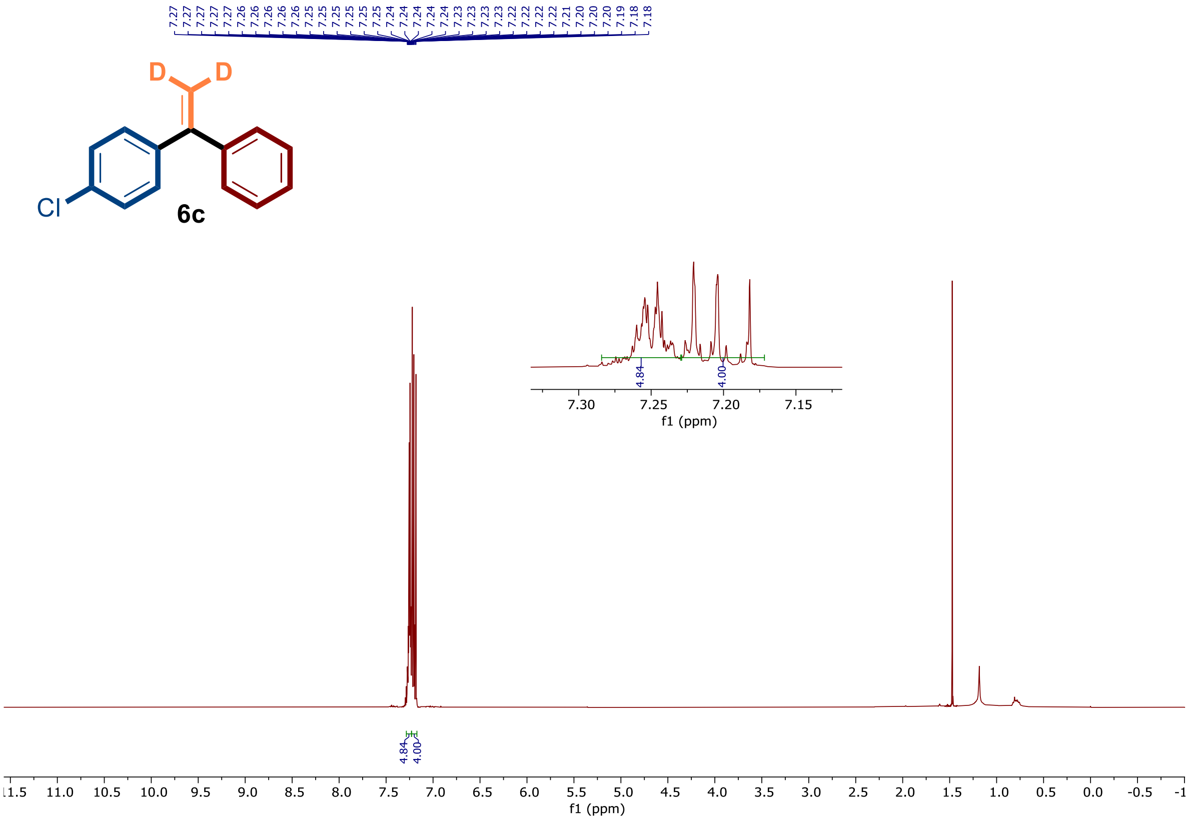

$^{13}C\{^{1}H\}$ NMR (101 MHz, $CDCl_3$) of compound (**6c**)

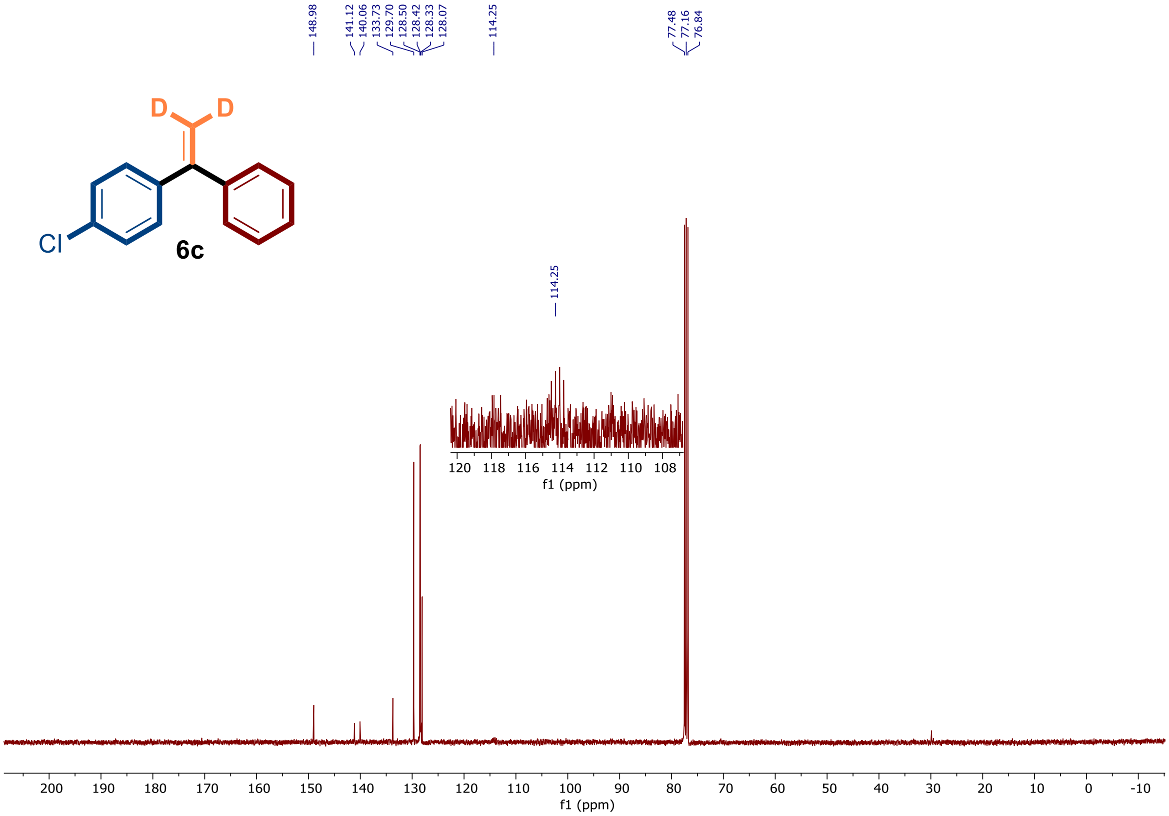

^{2}H NMR (77 MHz, $CDCl_3$) of compound (**6c**)

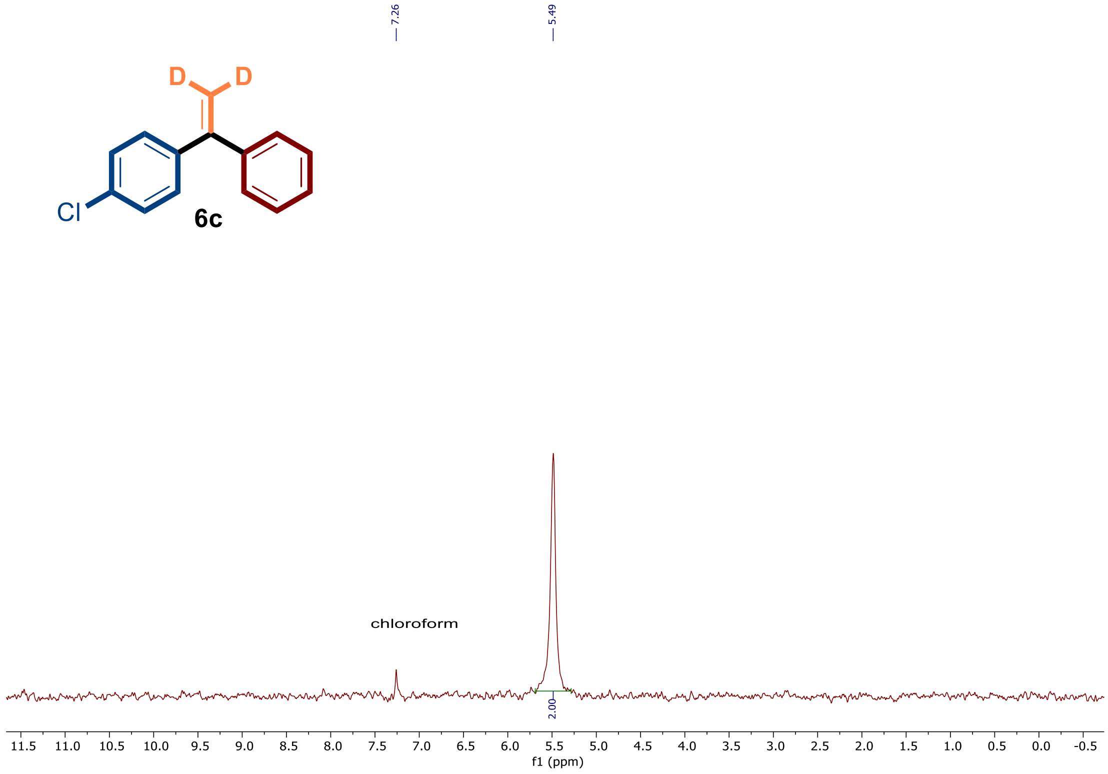

^{1}H NMR (400 MHz, CDCl$_3$) of compound (**6d**)

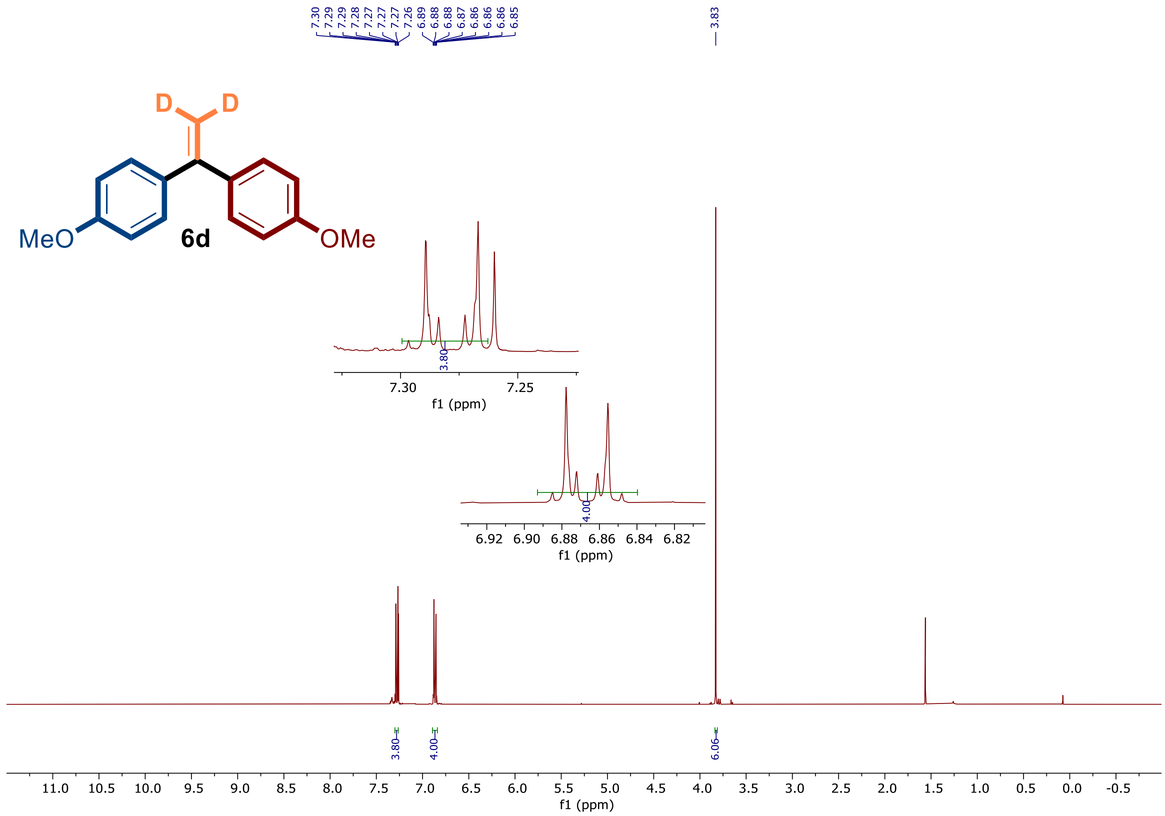

^{1}H NMR (400 MHz, CDCl$_3$) of compound (**6e**)

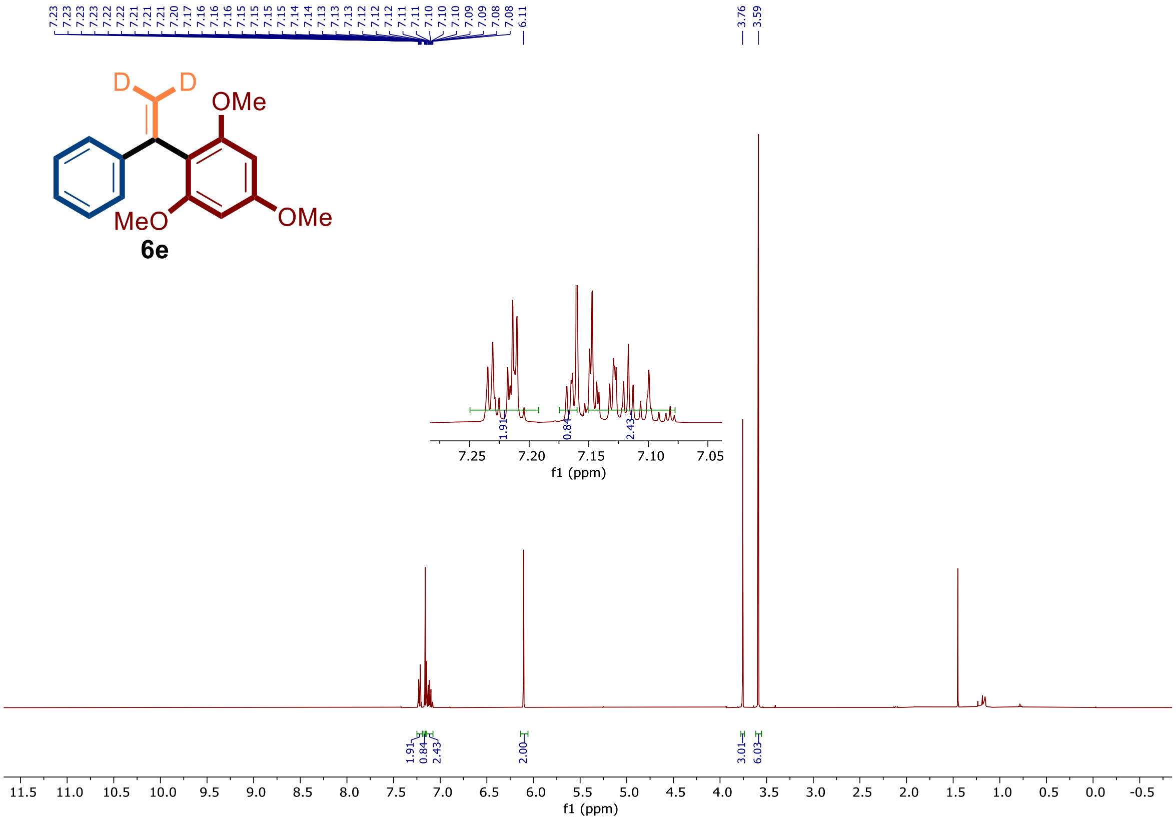

$^{13}C\{^1H\}$ NMR (101 MHz, $CDCl_3$) of compound (**6e**)

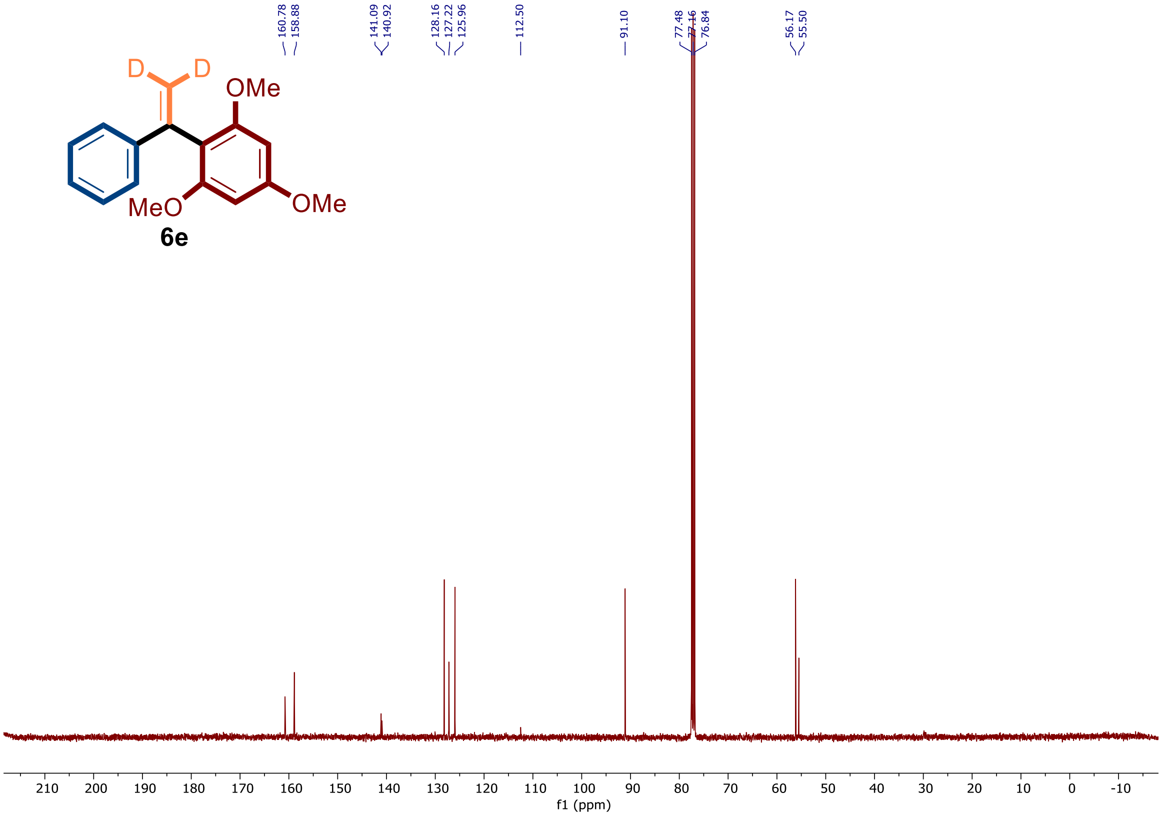

^2H NMR (77 MHz, $CDCl_3$) of compound (**6e**)

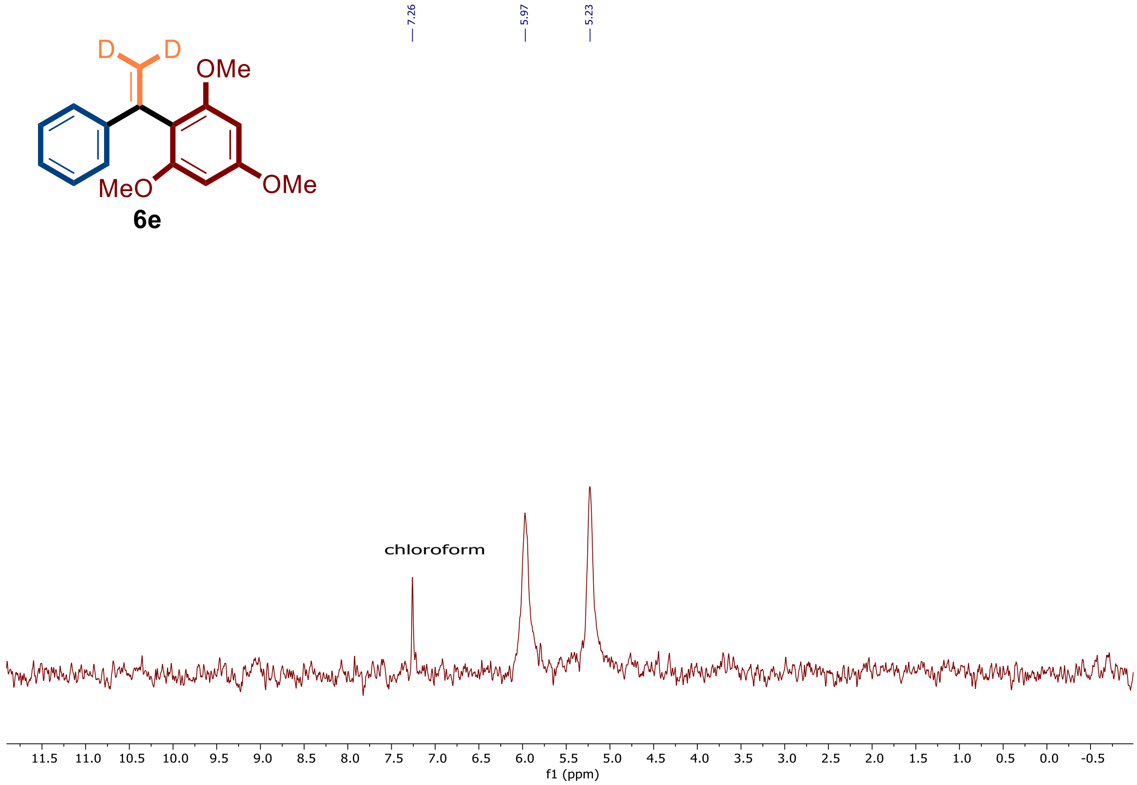

^{1}H NMR (400 MHz, $CDCl_3$) of compound (**6f**)

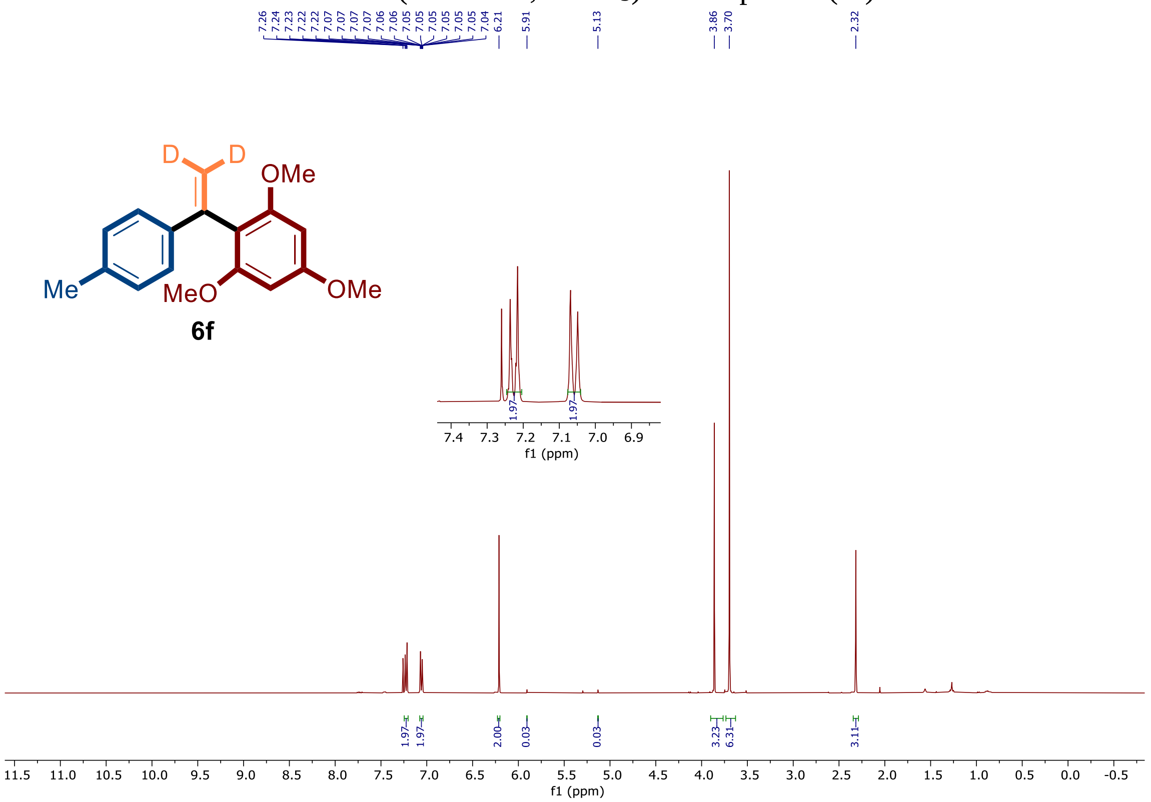

$^{13}C\{^{1}H\}$ NMR (101 MHz, $CDCl_3$) of compound (**6f**)

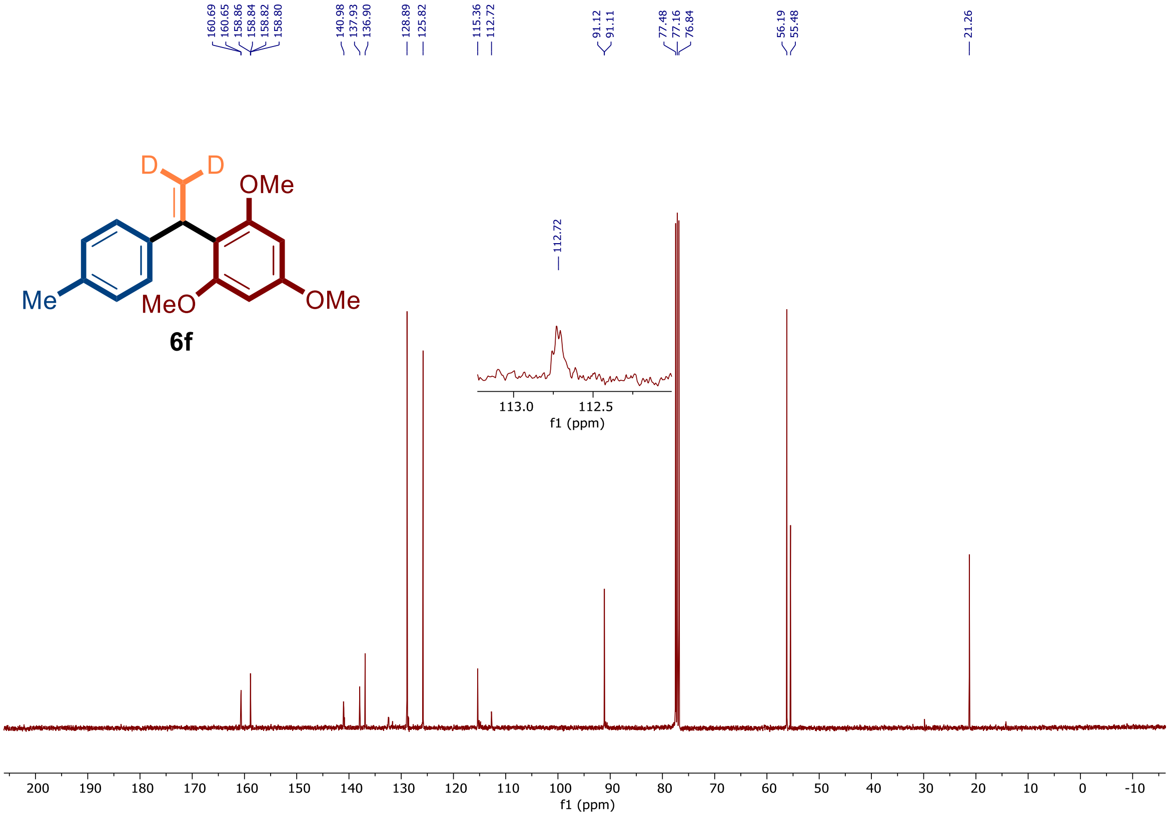

^{1}H NMR (400 MHz, $CDCl_3$) of compound (**6g**)

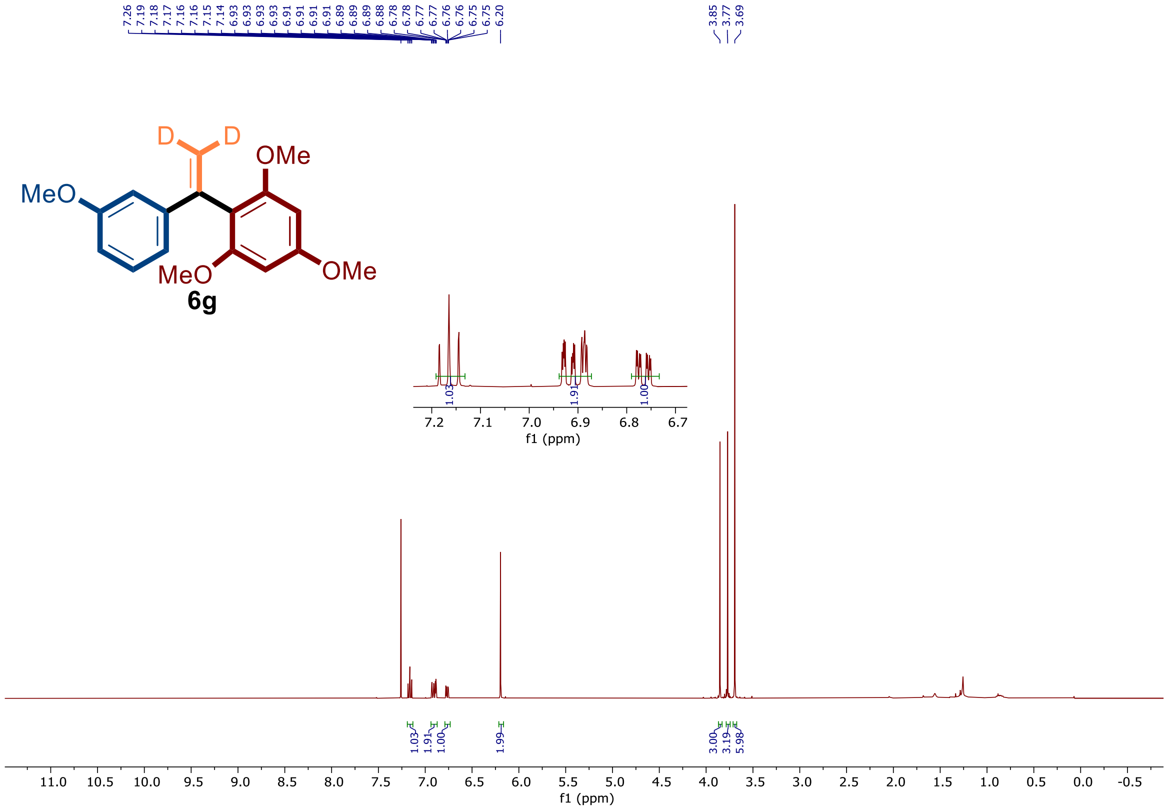

$^{13}C\{^{1}H\}$ NMR (101 MHz, $CDCl_3$) of compound (**6g**)

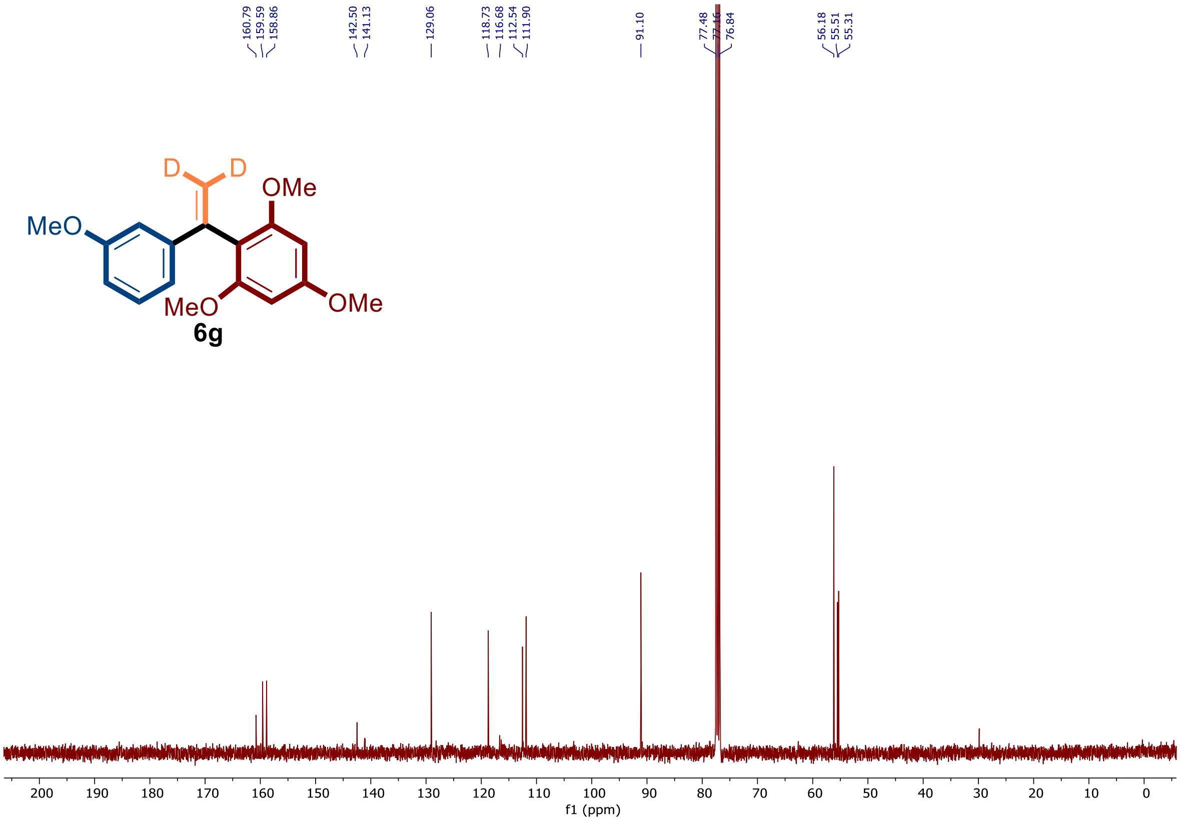

^{1}H NMR (400 MHz, $CDCl_3$) of compound (**6h**)

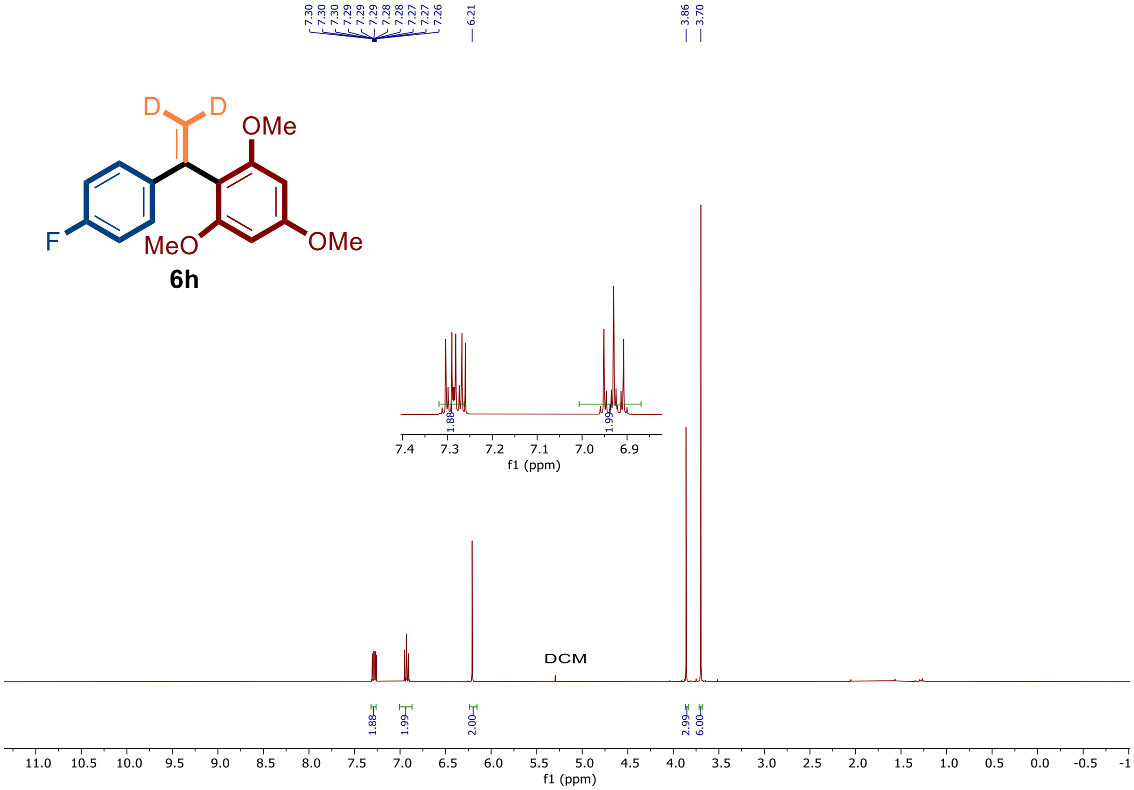

^{13}C{^{1}H} NMR (101 MHz, $CDCl_3$) of compound (**6h**)

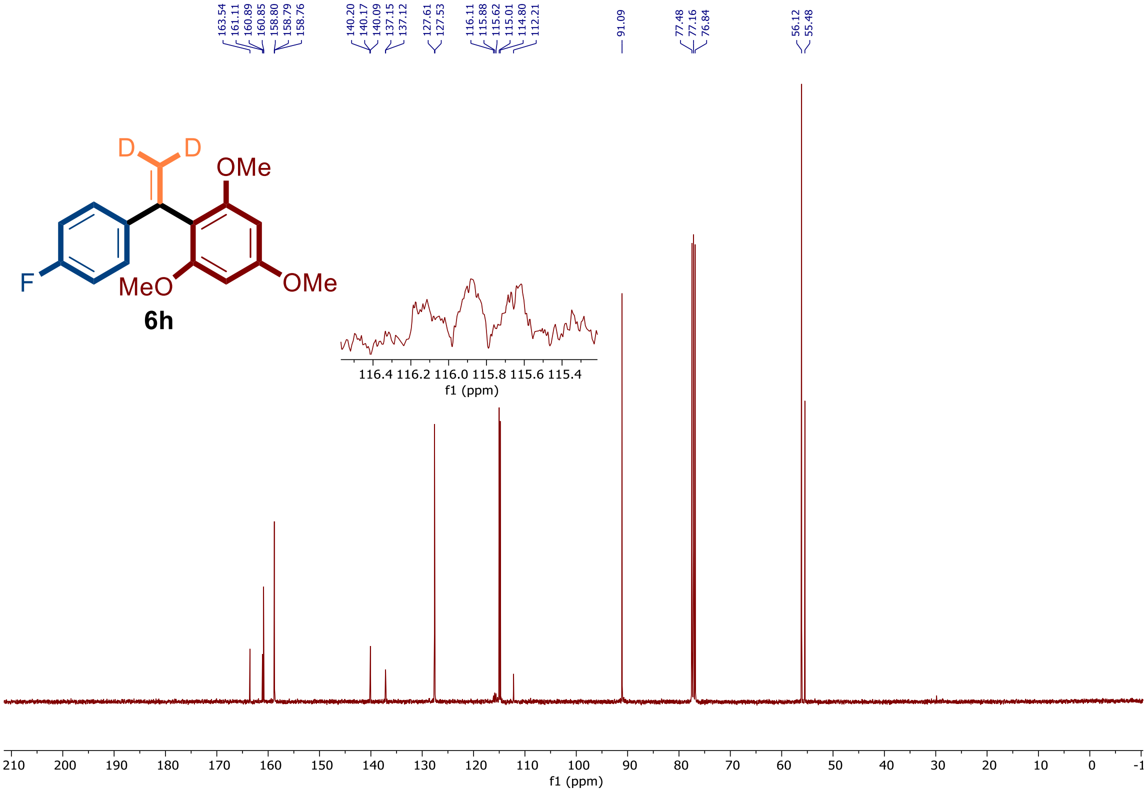

^{19}F NMR (376 MHz, CDCl$_3$) of compound (**6h**)

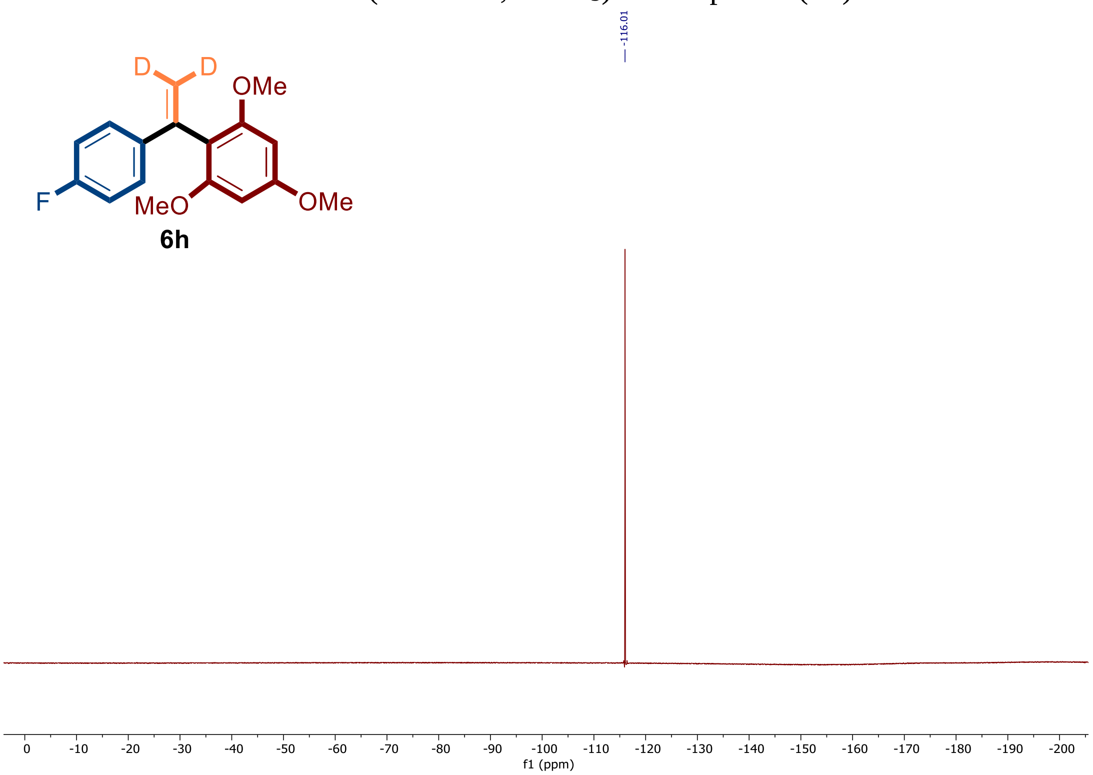

^{1}H NMR (400 MHz, CDCl$_3$) of compound (**6i**)

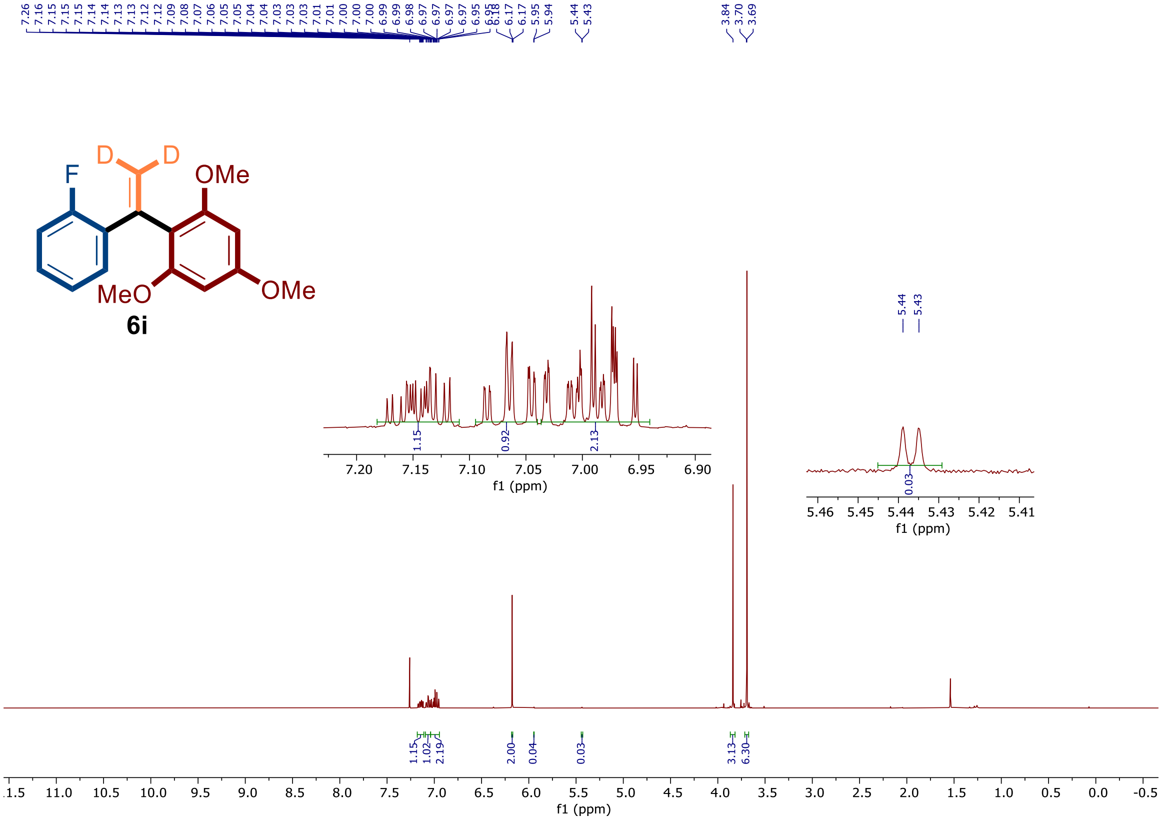

$^{13}C\{^{1}H\}$ NMR (101 MHz, $CDCl_3$) of compound (**6i**)

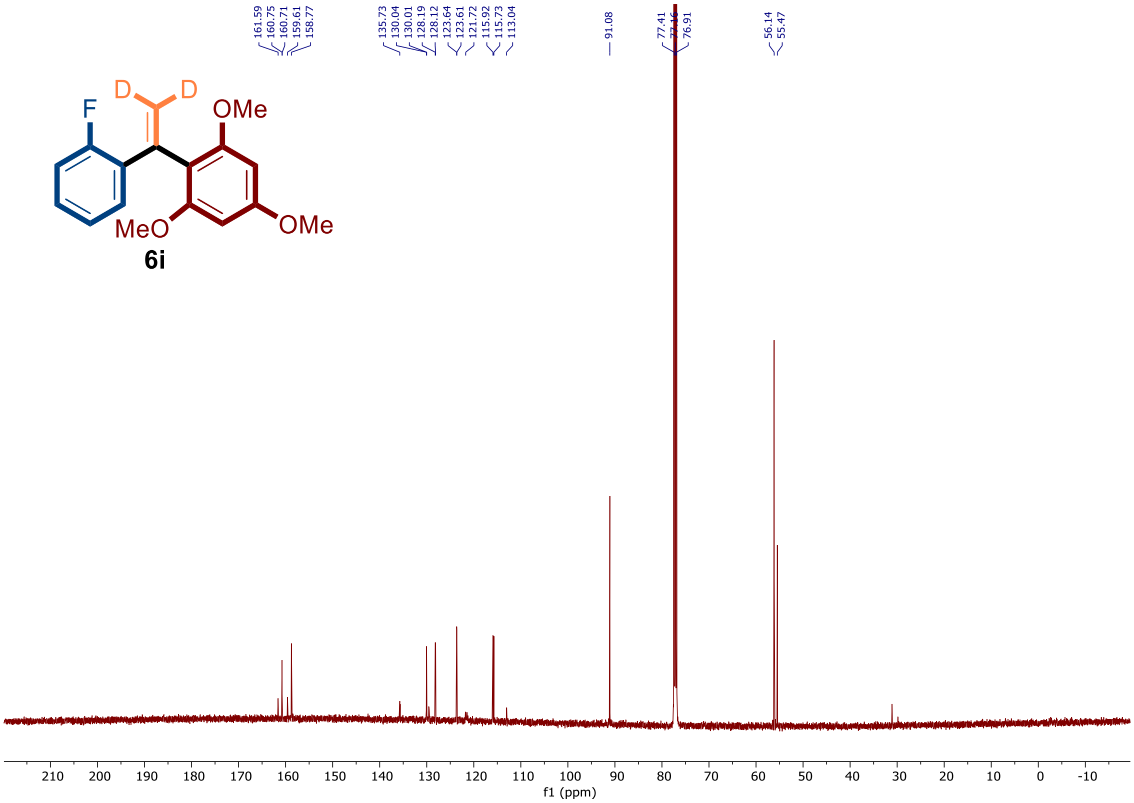

^{19}F NMR (376 MHz, $CDCl_3$) of compound (**6i**)

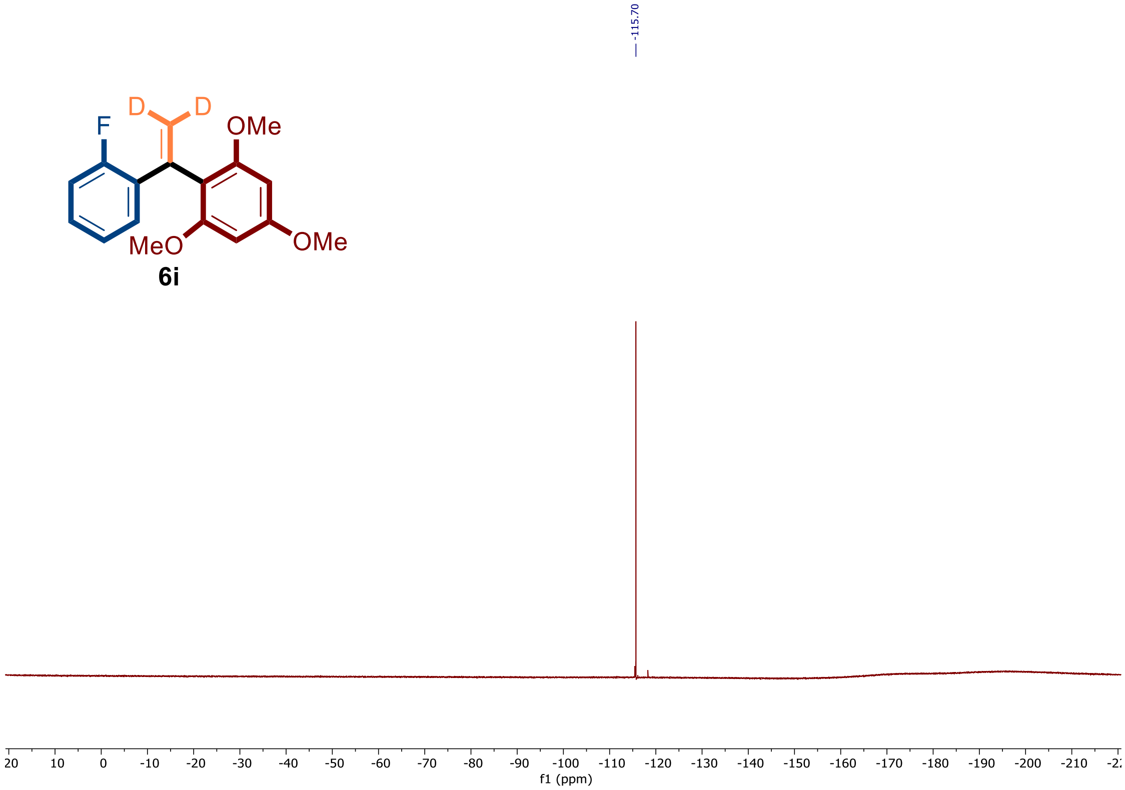

^{1}H NMR (400 MHz, $CDCl_3$) of compound (**6j**)

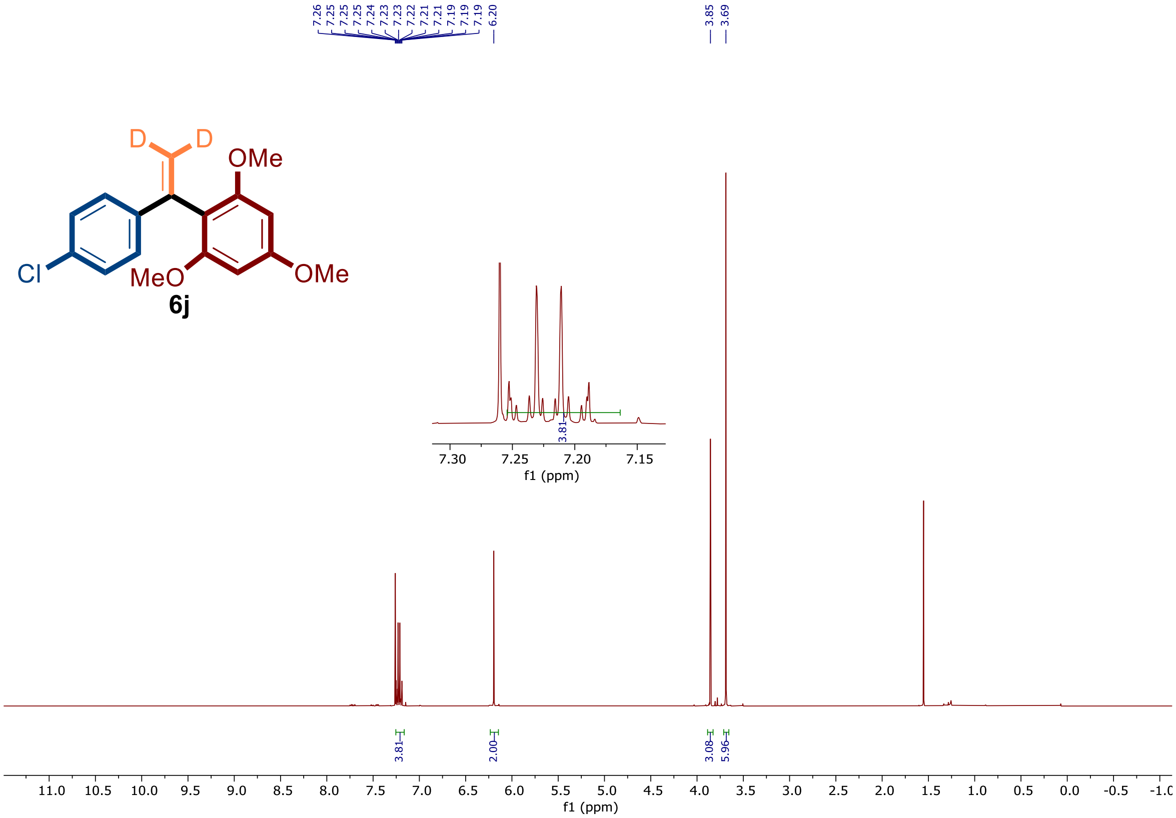

^{13}C{^{1}H} NMR (101 MHz, $CDCl_3$) of compound (**6j**)

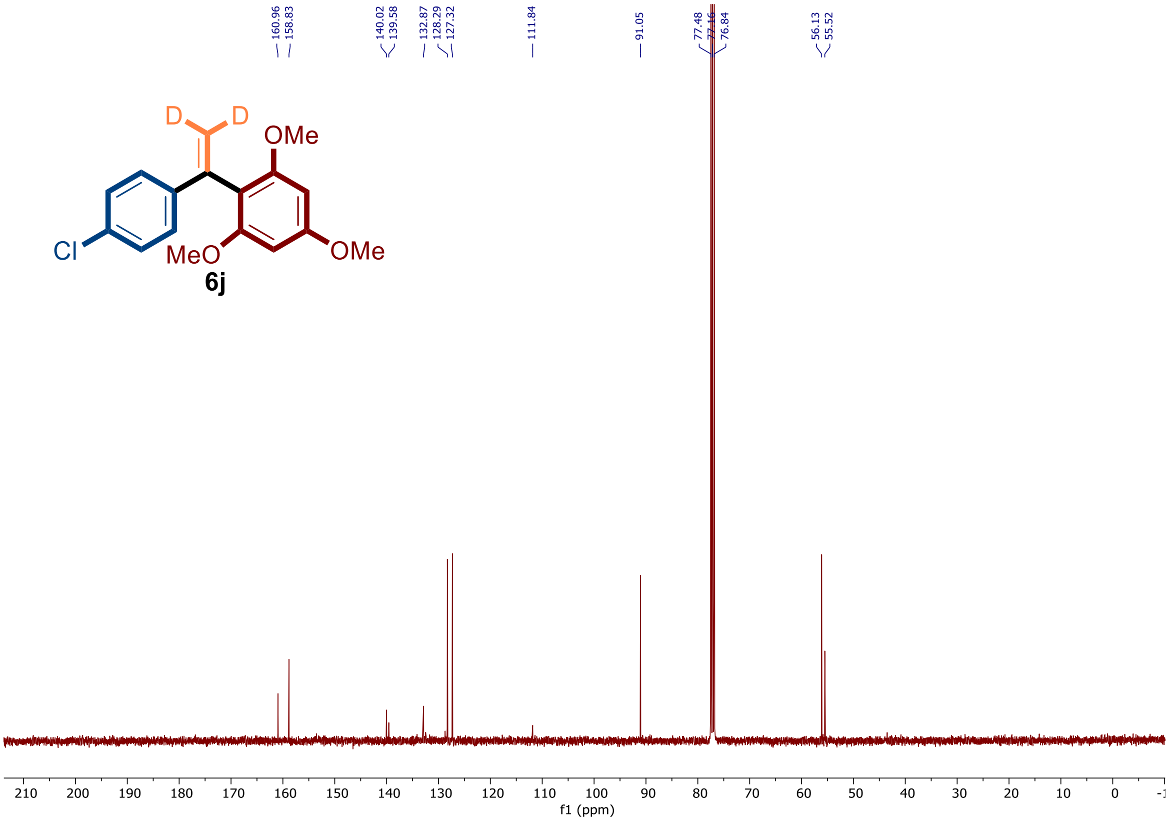

^{1}H NMR (400 MHz, $CDCl_3$) of compound (**6k**)

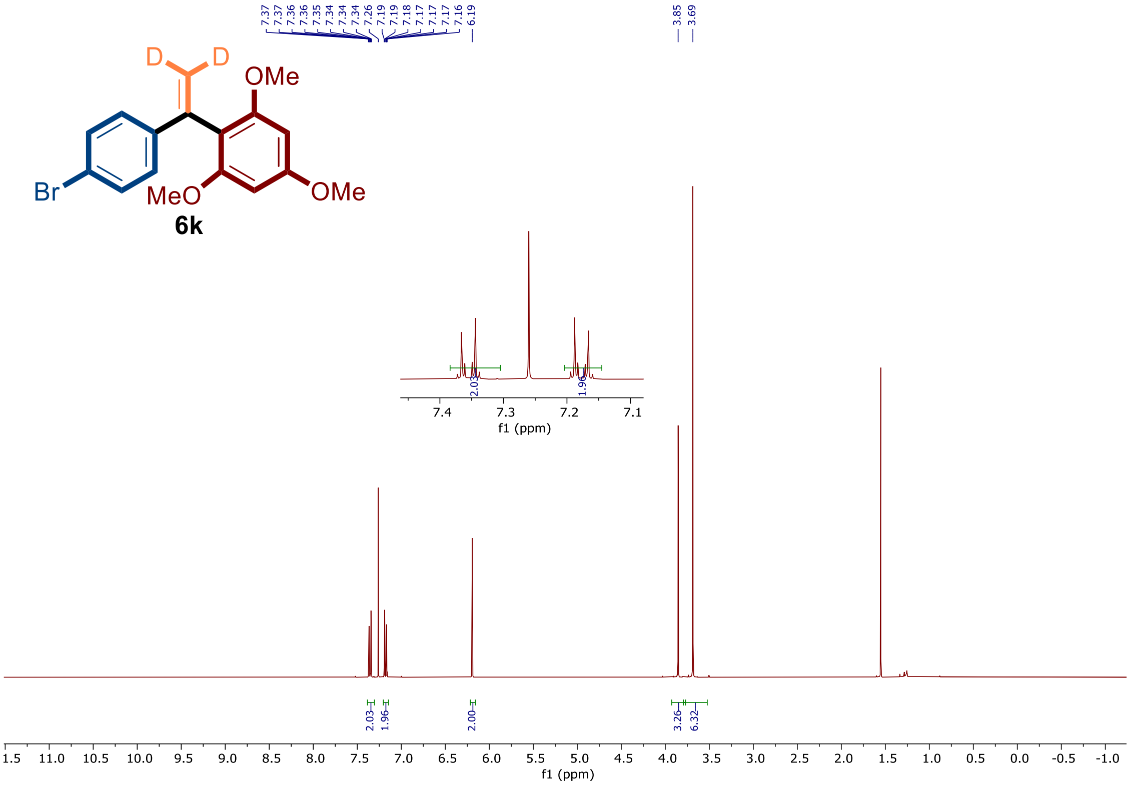

^{13}C NMR (101 MHz, $CDCl_3$) of compound (**6k**)

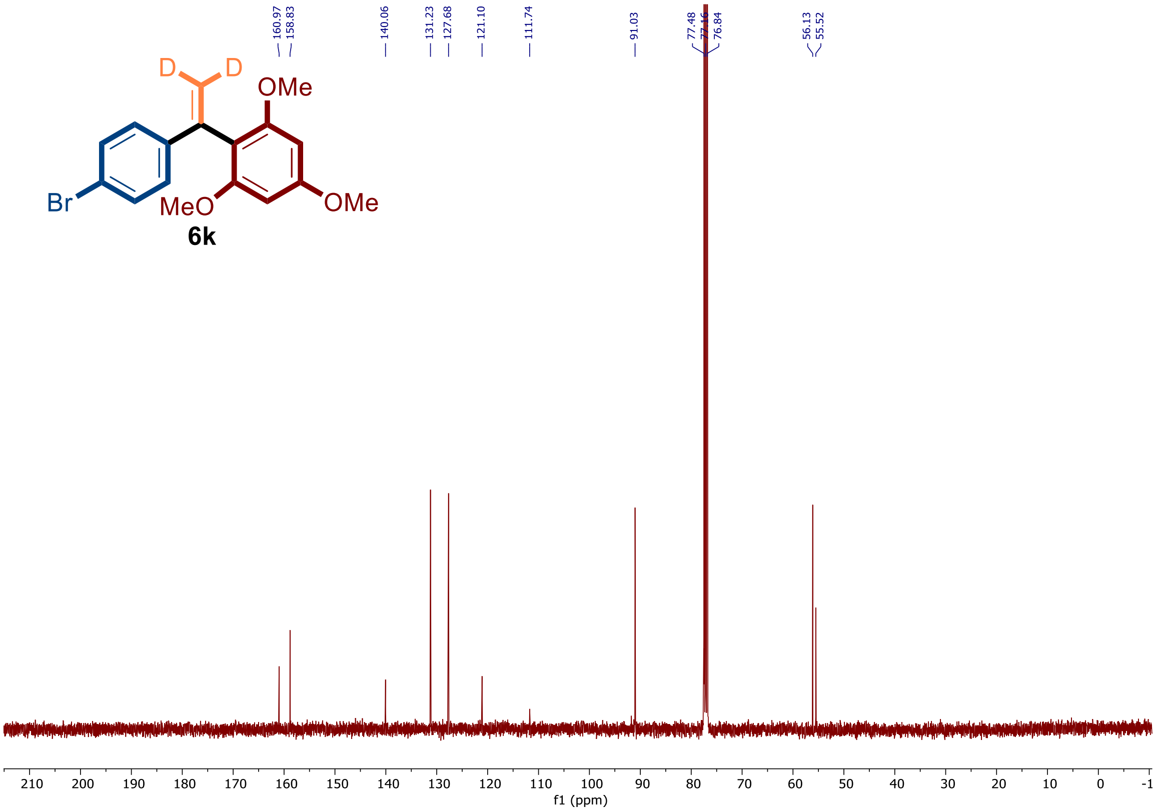

^{1}H NMR (400 MHz, $CDCl_3$) of compound (**6l**)

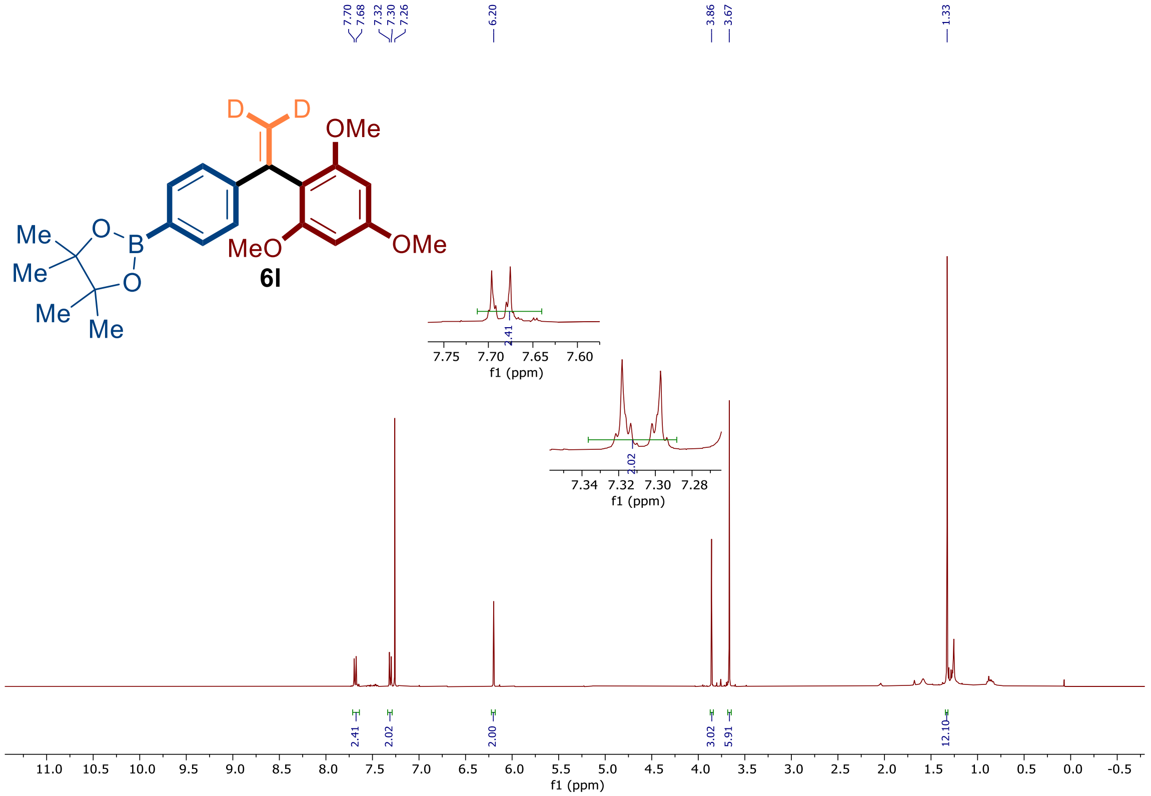

^{13}C{^{1}H} NMR (101 MHz, $CDCl_3$) of compound (**6l**)

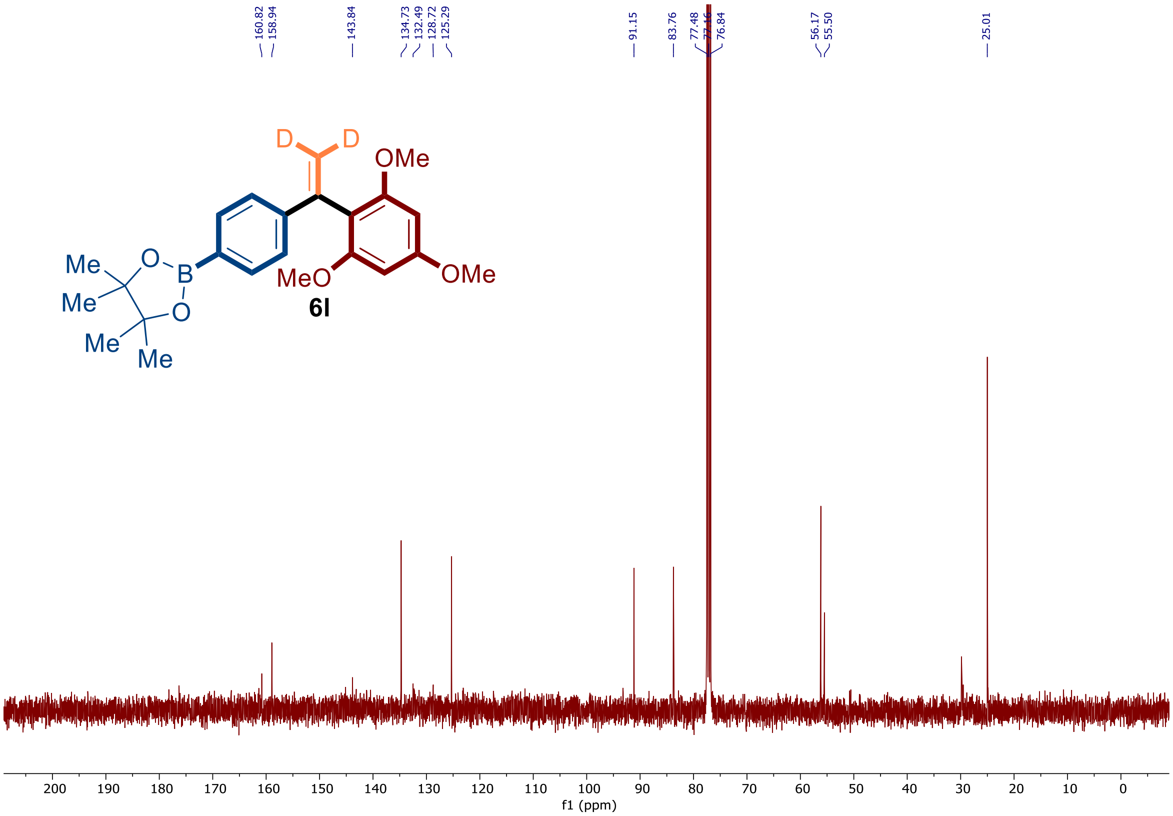

^{11}B NMR (128 MHz, $CDCl_3$) of compound (**6l**)

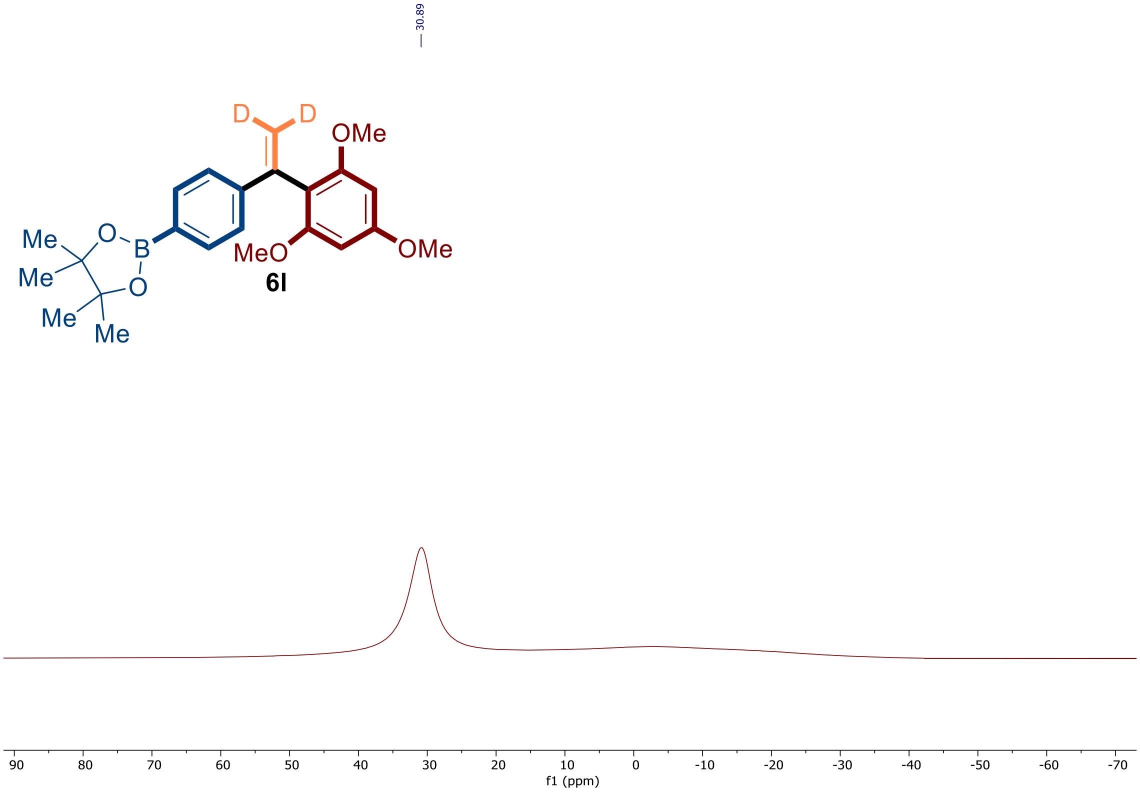

^{1}H NMR (400 MHz, $CDCl_3$) of compound (**6m**)

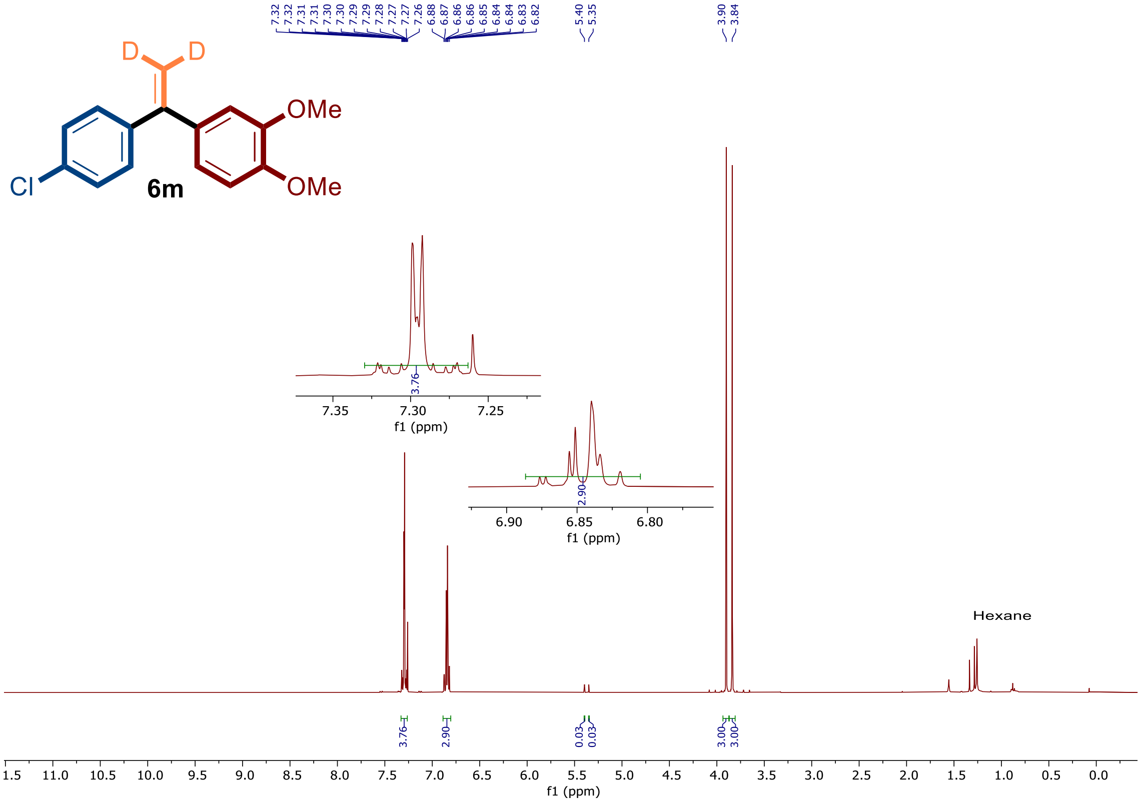

$^{13}C\{^1H\}$ NMR (101 MHz, $CDCl_3$) of compound (**6m**)

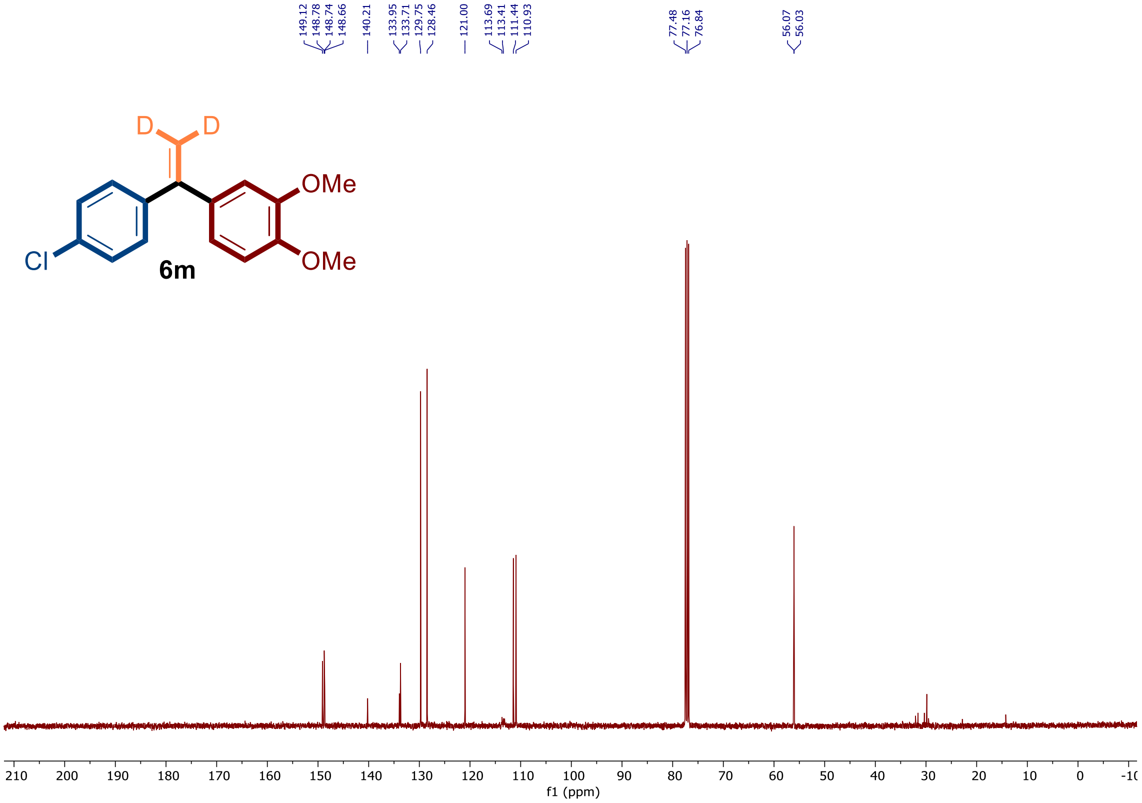

^1H NMR (400 MHz, $CDCl_3$) of compound (**6n**)

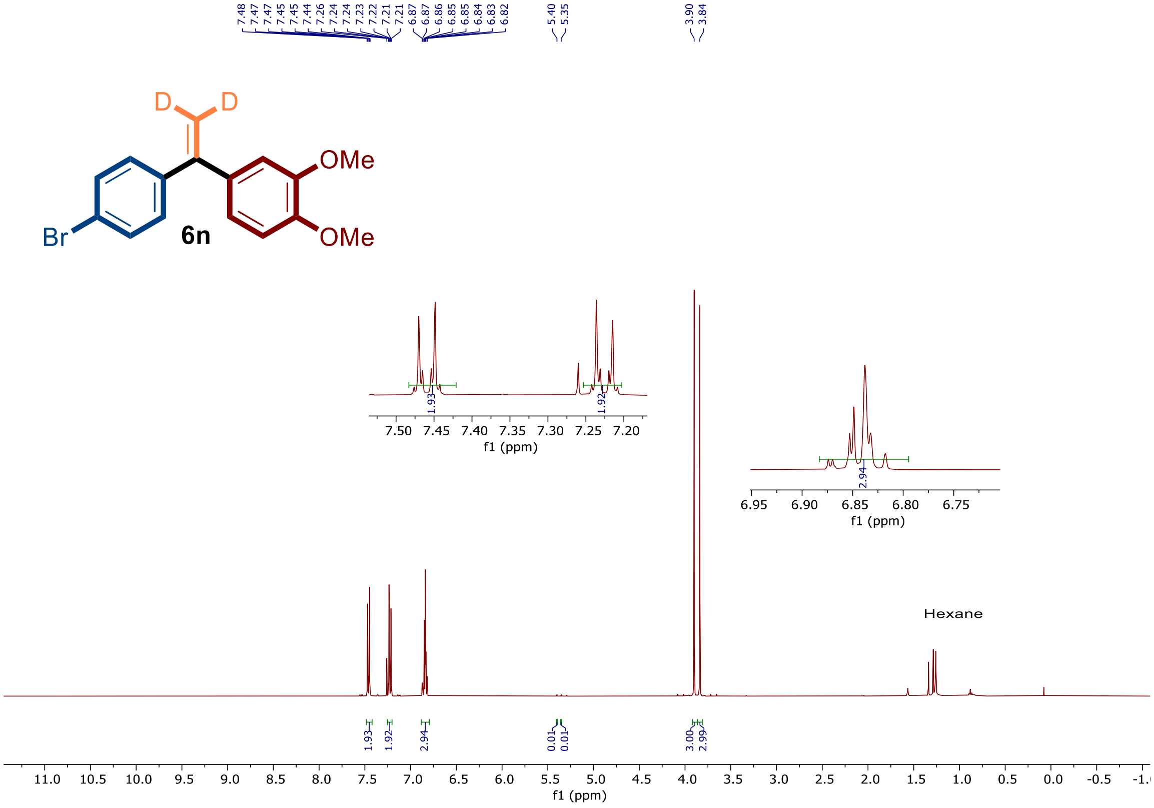

$^{13}C\{^{1}H\}$ NMR (101 MHz, $CDCl_3$) of compound (**6n**)

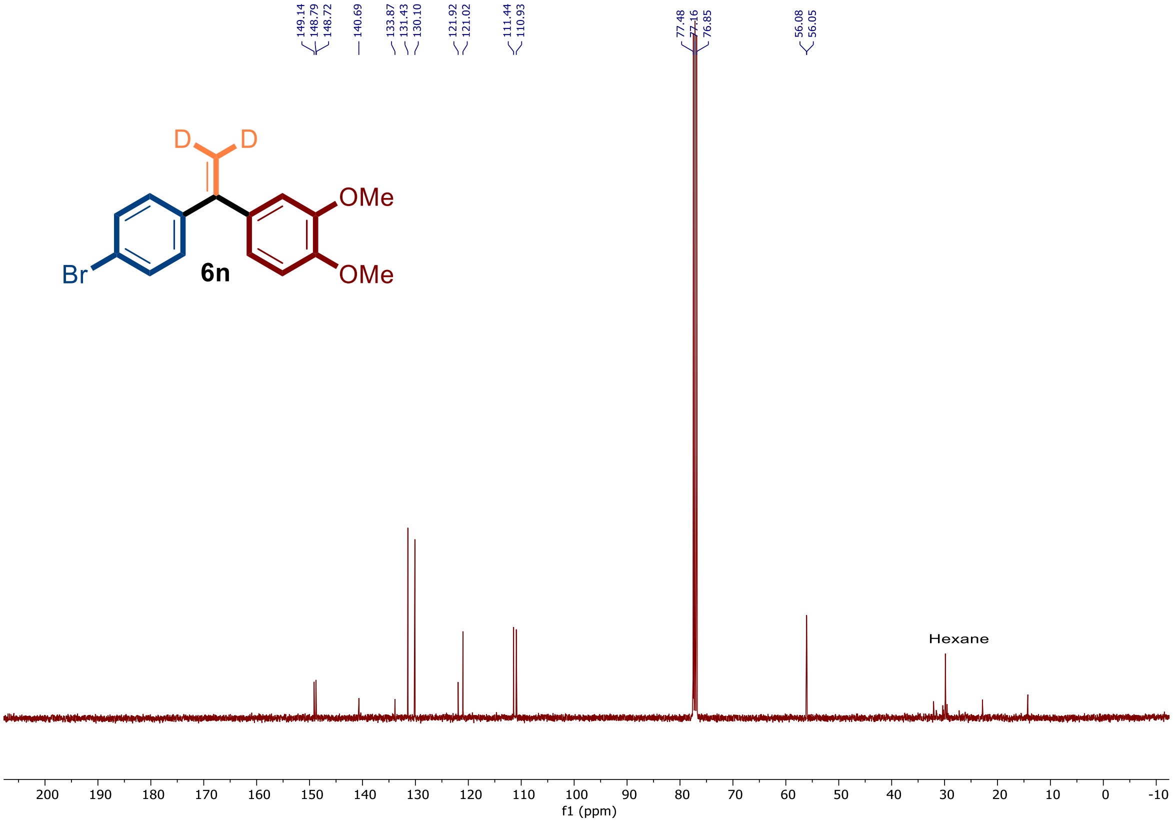

^{1}H NMR (400 MHz, $CDCl_3$) of compound (**6o**)

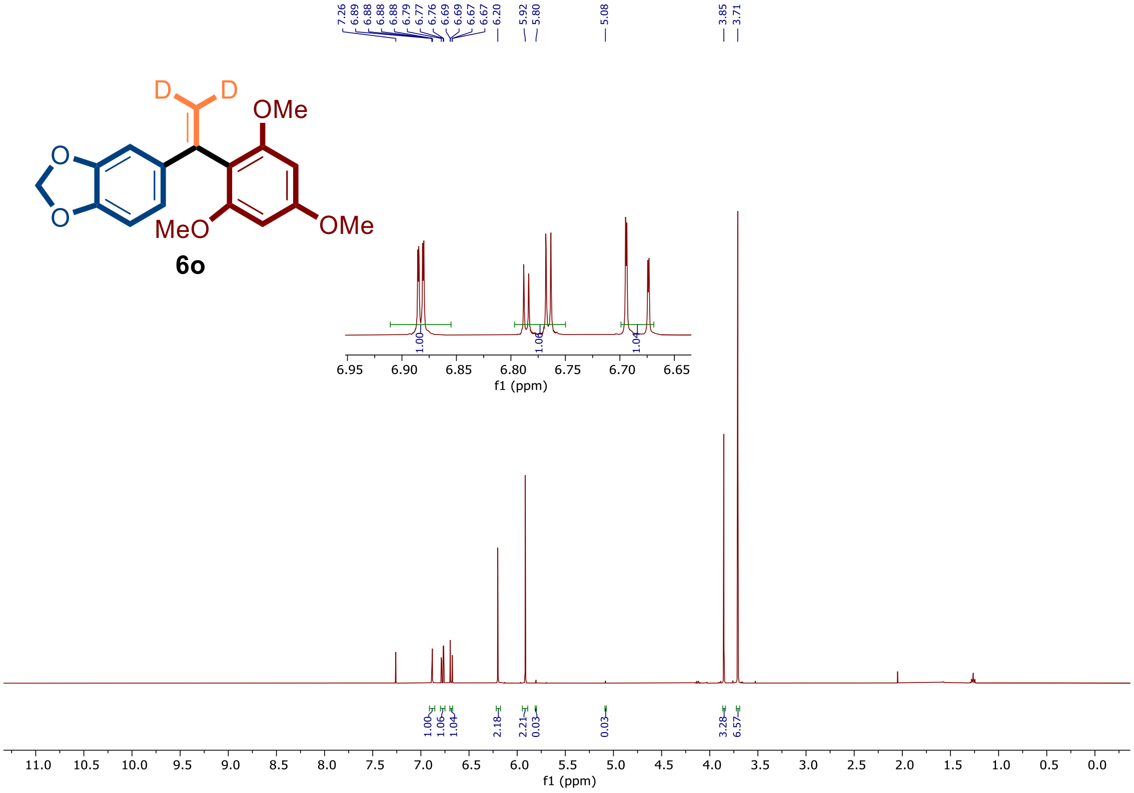

$^{13}C\{^{1}H\}$ NMR (101 MHz, $CDCl_3$) of compound (**6o**)

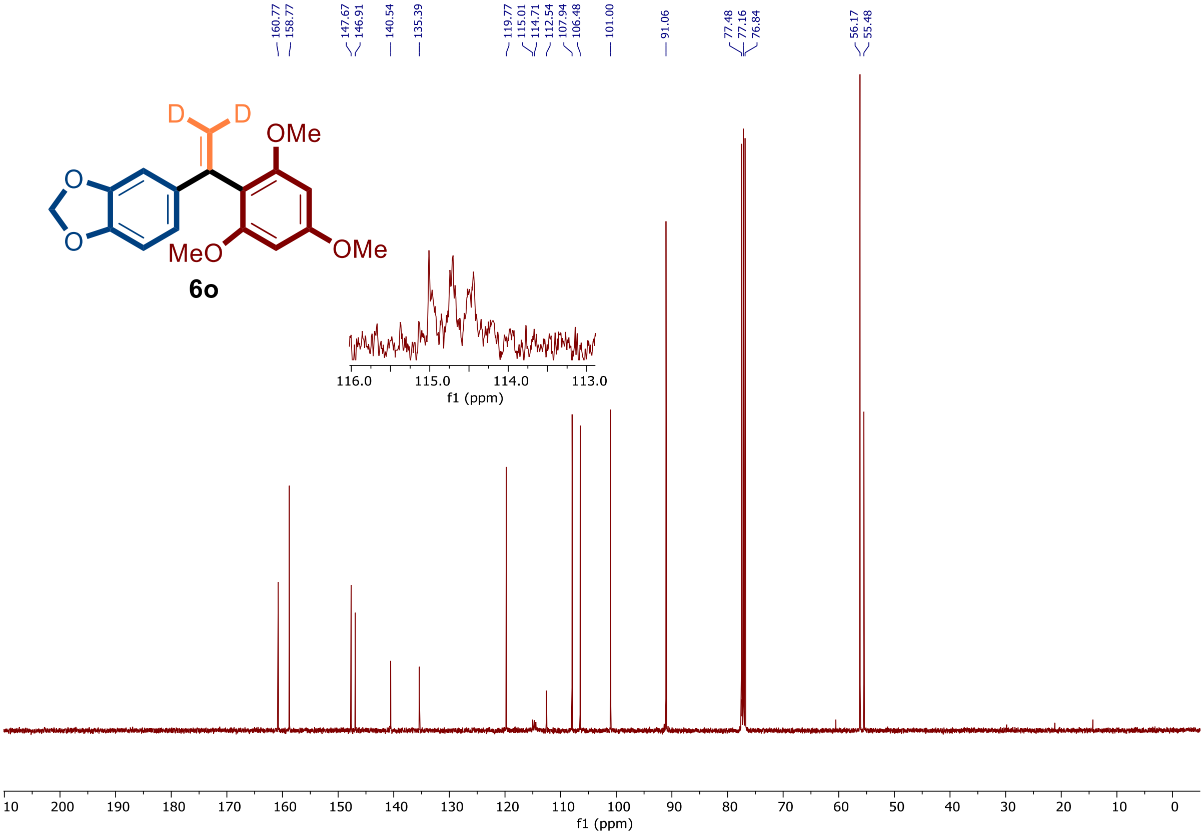

^{1}H NMR (400 MHz, $CDCl_3$) of compound (**6p**)

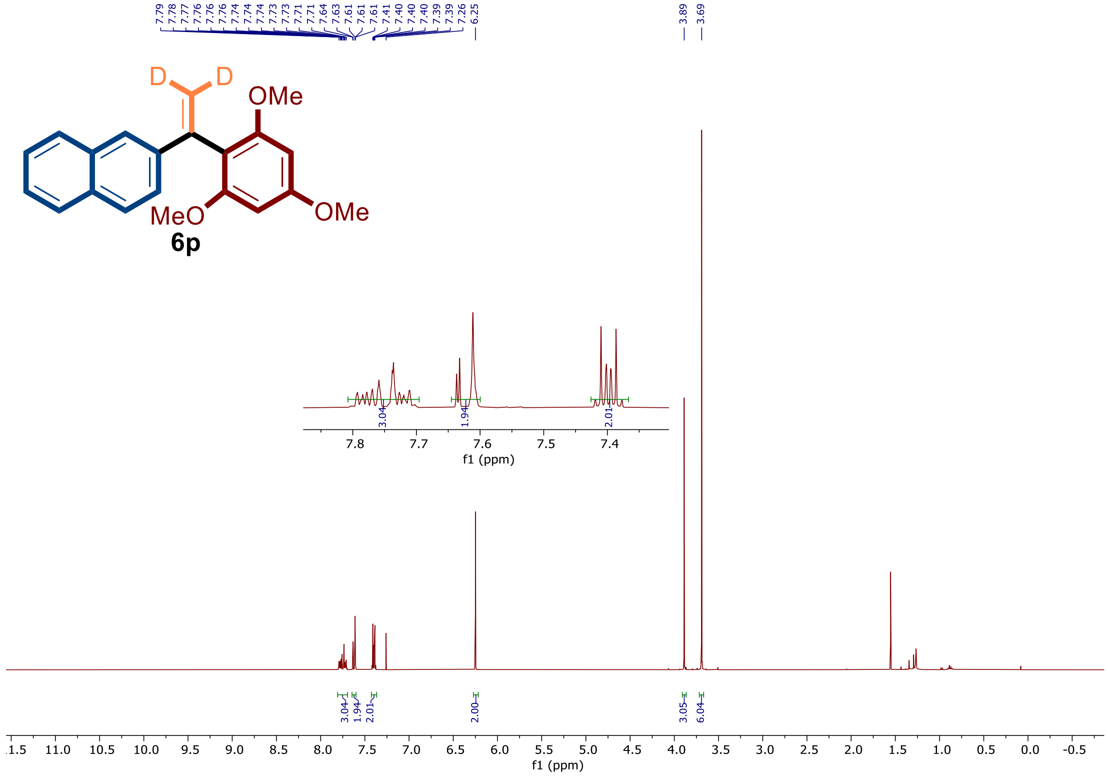

$^{13}C\{^1H\}$ NMR (101 MHz, $CDCl_3$) of compound (**6p**)

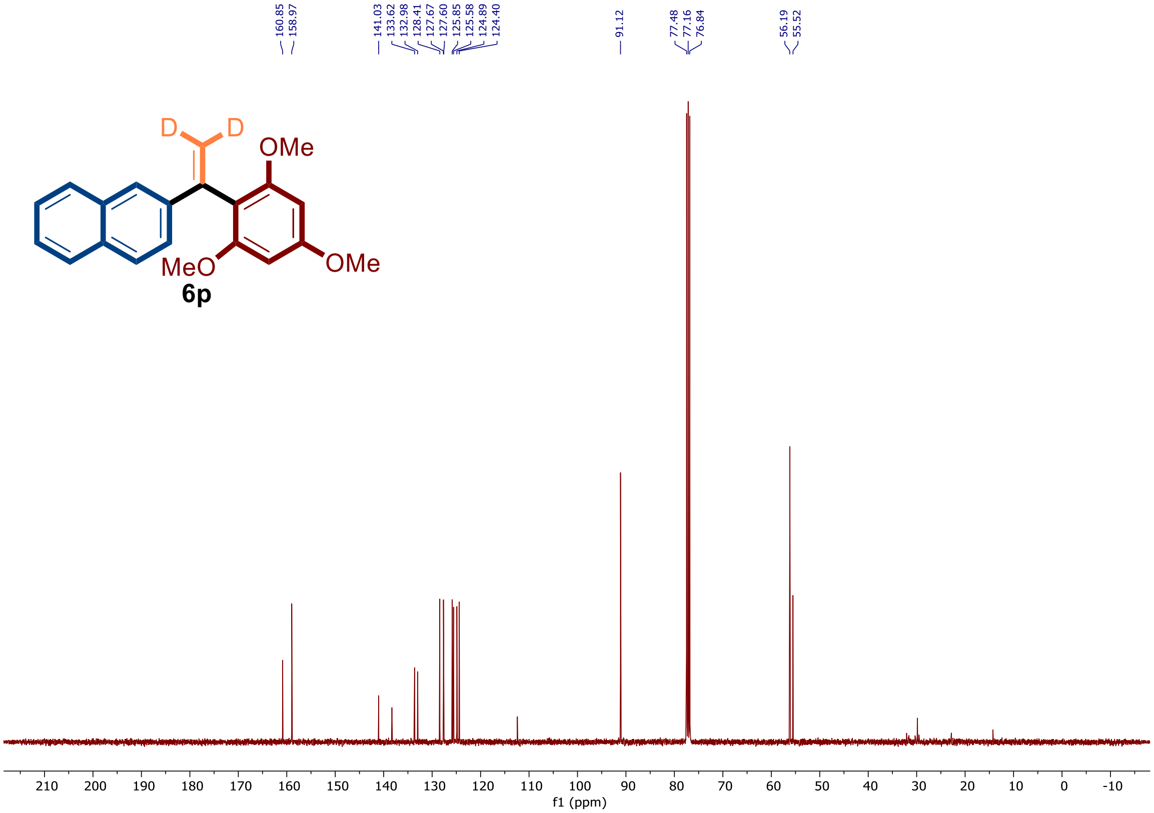

^1H NMR (400 MHz, $CDCl_3$) of compound (**6q**)

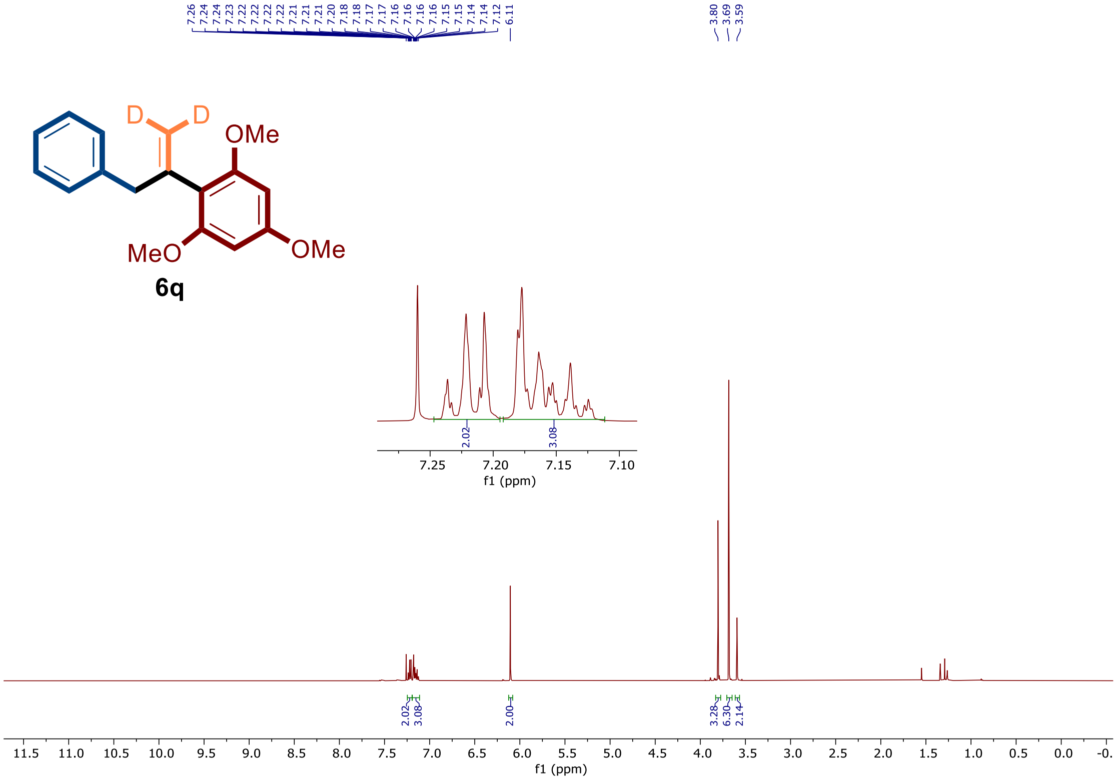

$^{13}C\{^{1}H\}$ NMR (101 MHz, $CDCl_3$) of compound (**6q**)

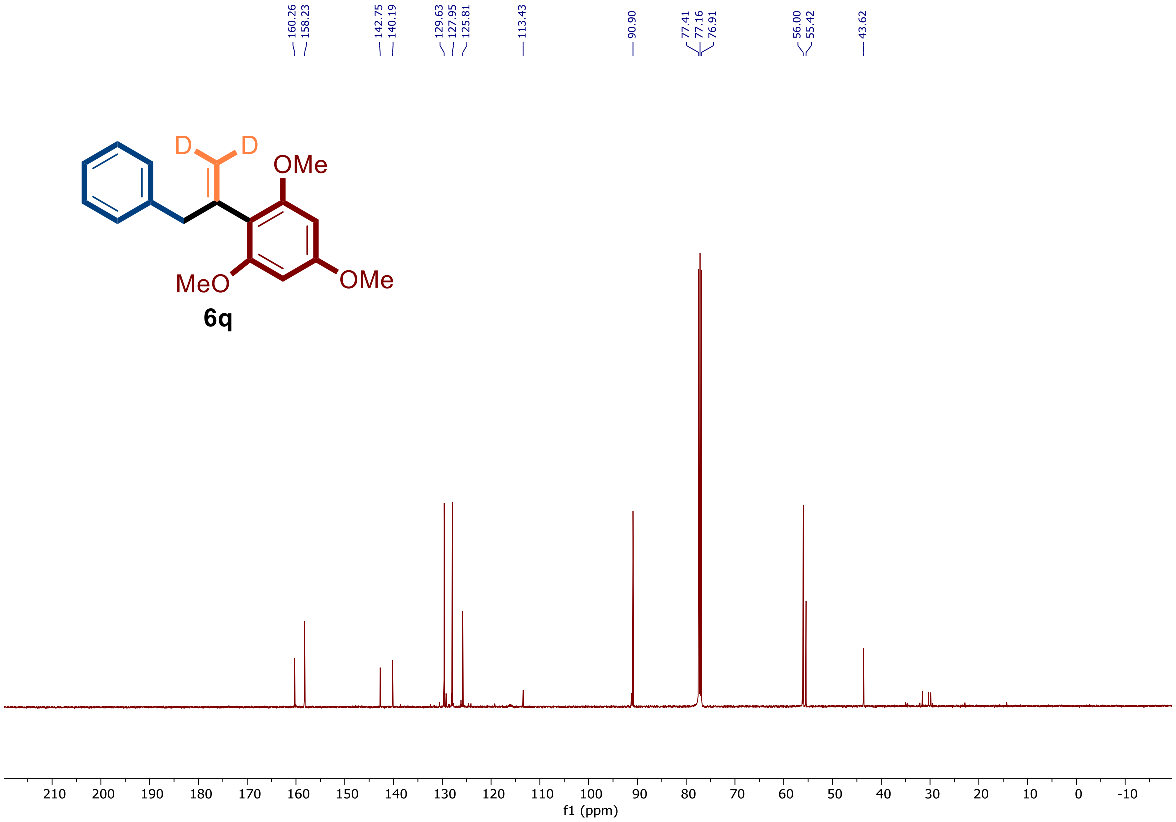

^{1}H NMR (400 MHz, $CDCl_3$) of compound (**6r**)

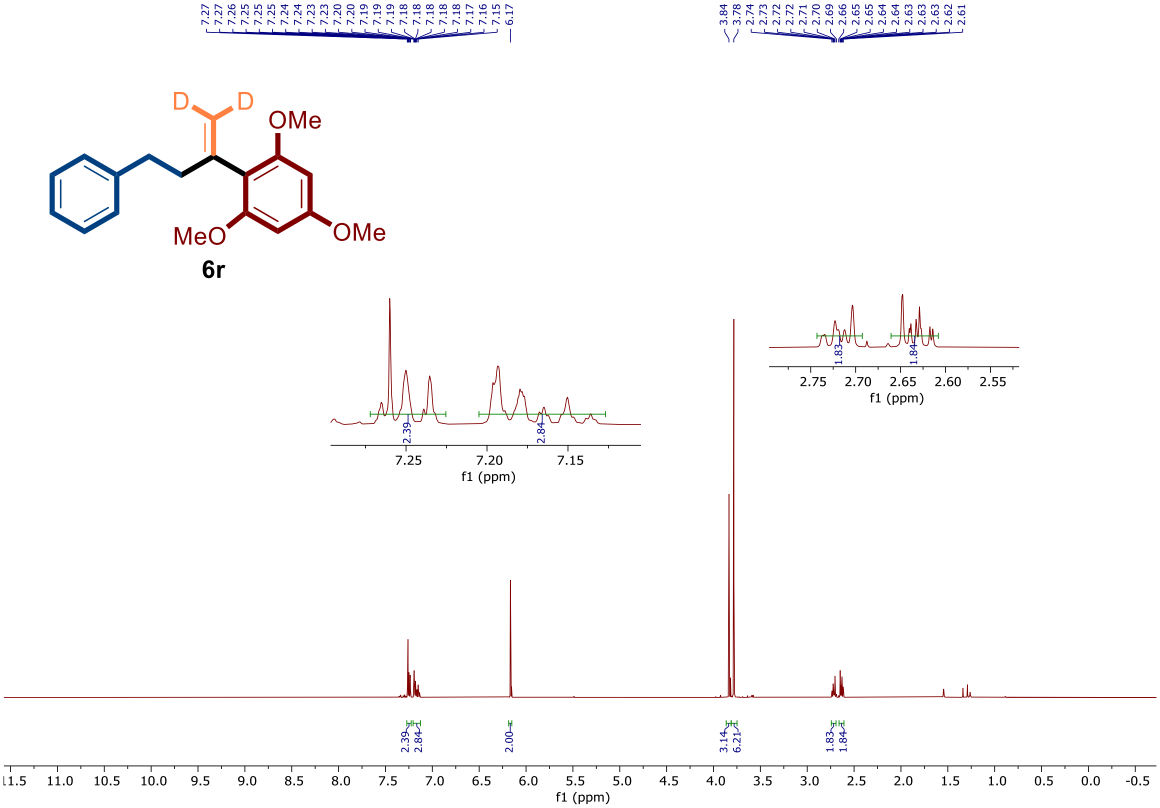

$^{13}C\{^{1}H\}$ NMR (101 MHz, $CDCl_3$) of compound (**6r**)

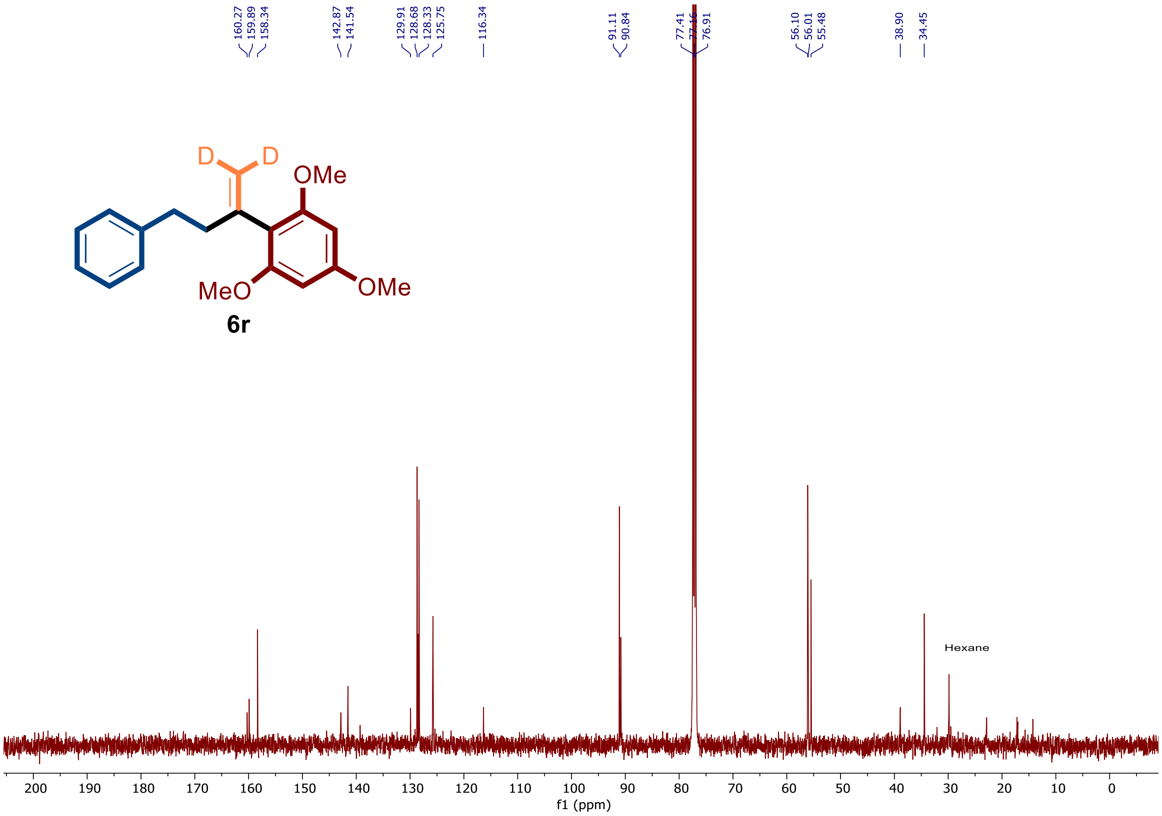

^{1}H NMR (400 MHz, $CDCl_3$) of compound (**6s**)

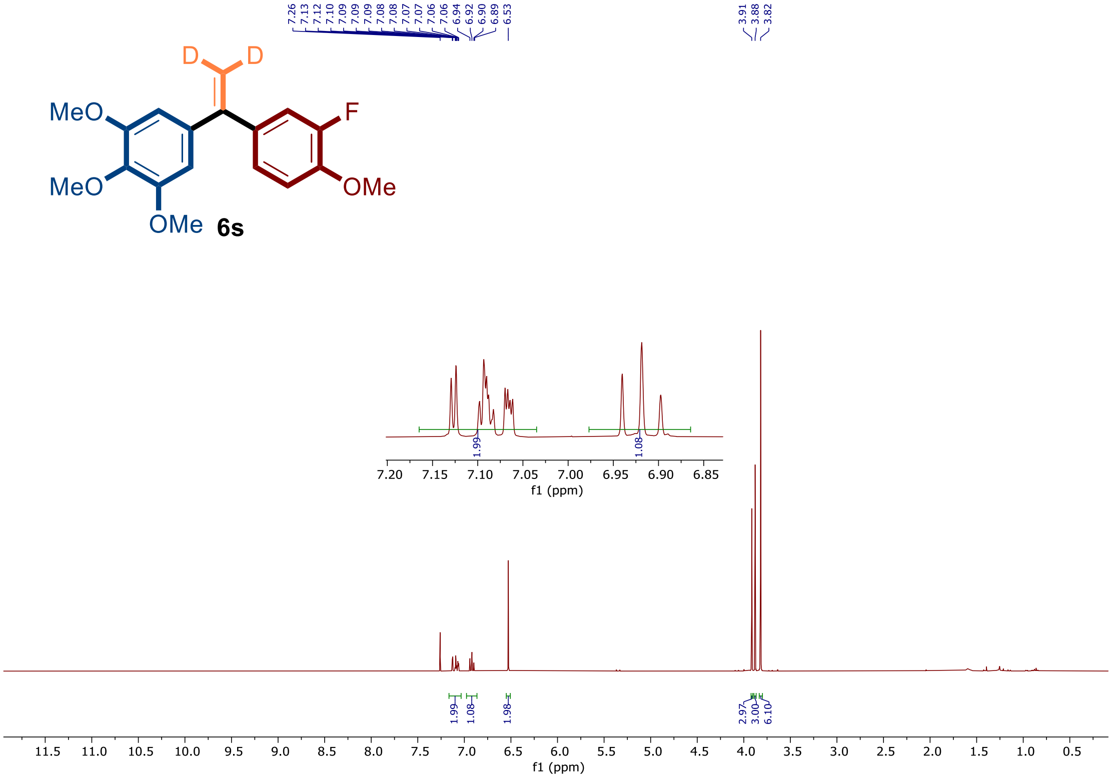

^{13}C{^{1}H} NMR (101 MHz, CDCl3) of compound (**6s**)

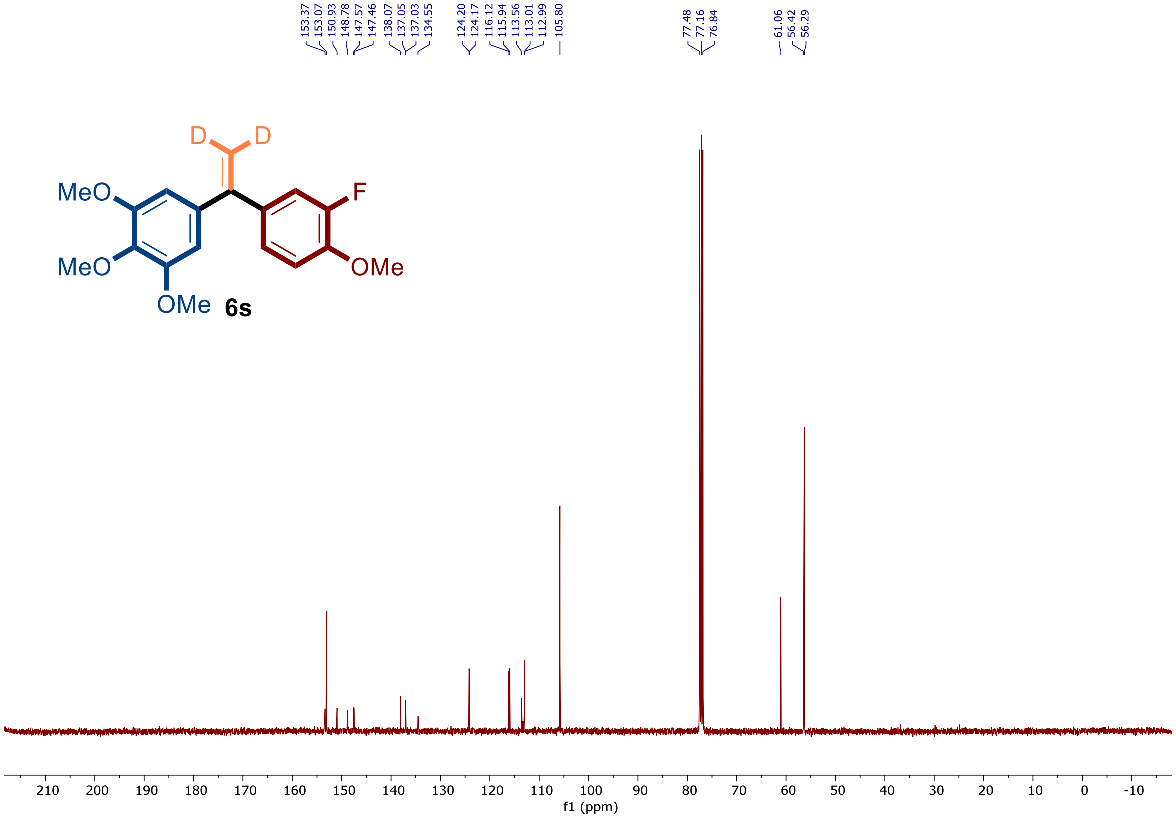

^{19}F NMR (376 MHz, CDCl3) of compound (**6s**)

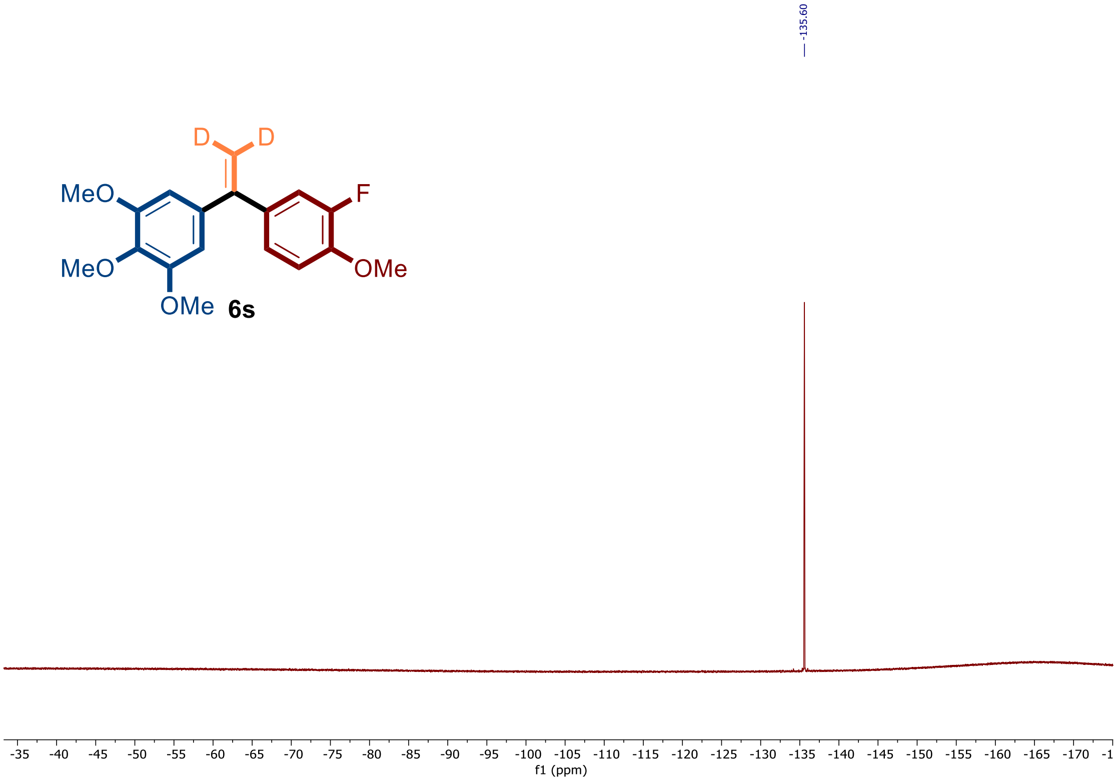

^{1}H NMR (400 MHz, $CDCl_3$) of compound (**2d**)

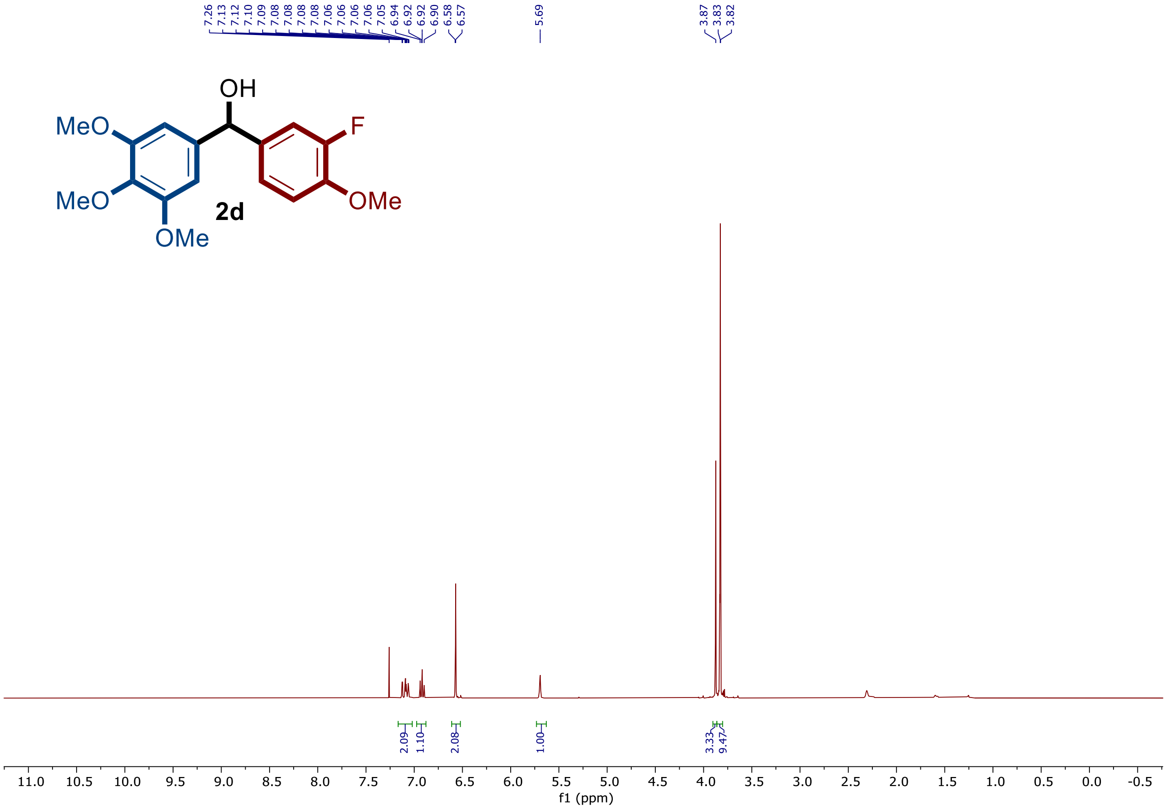

$^{13}C\{^{1}H\}$ NMR (101 MHz, $CDCl_3$) of compound (**2d**)

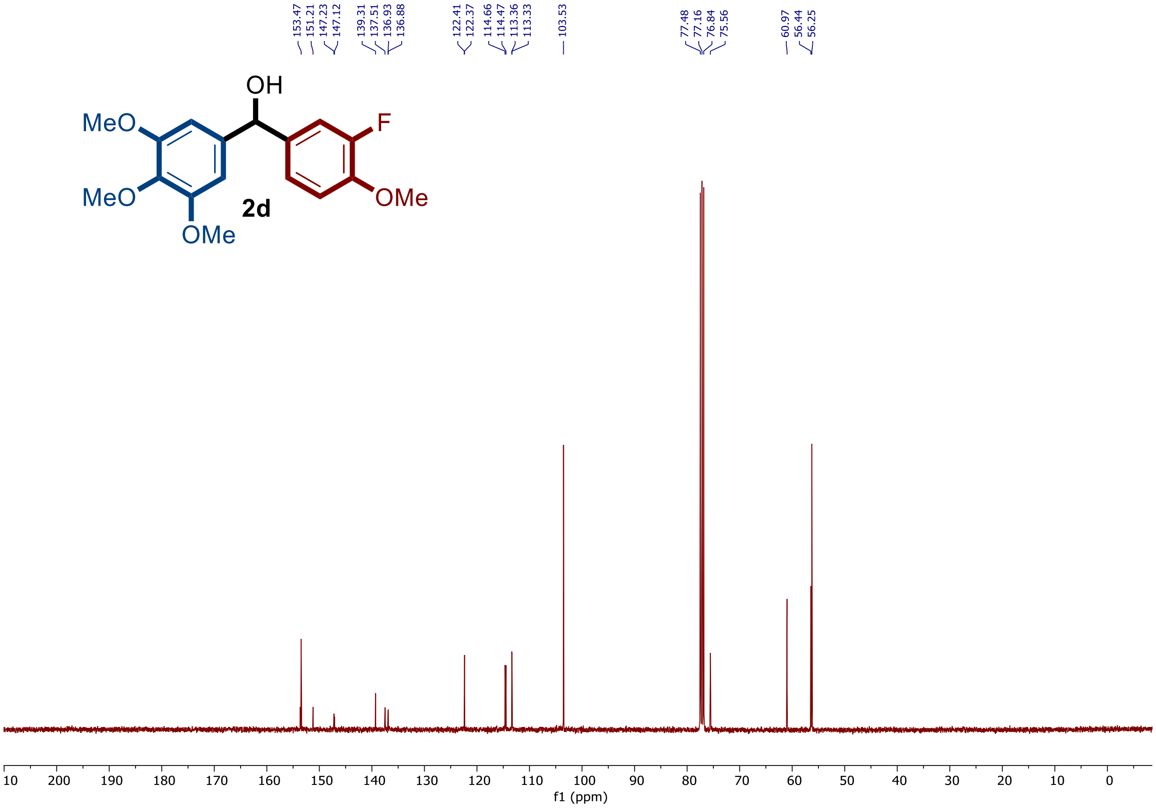

^{19}F NMR (376 MHz, $CDCl_3$) of compound (**2d**)

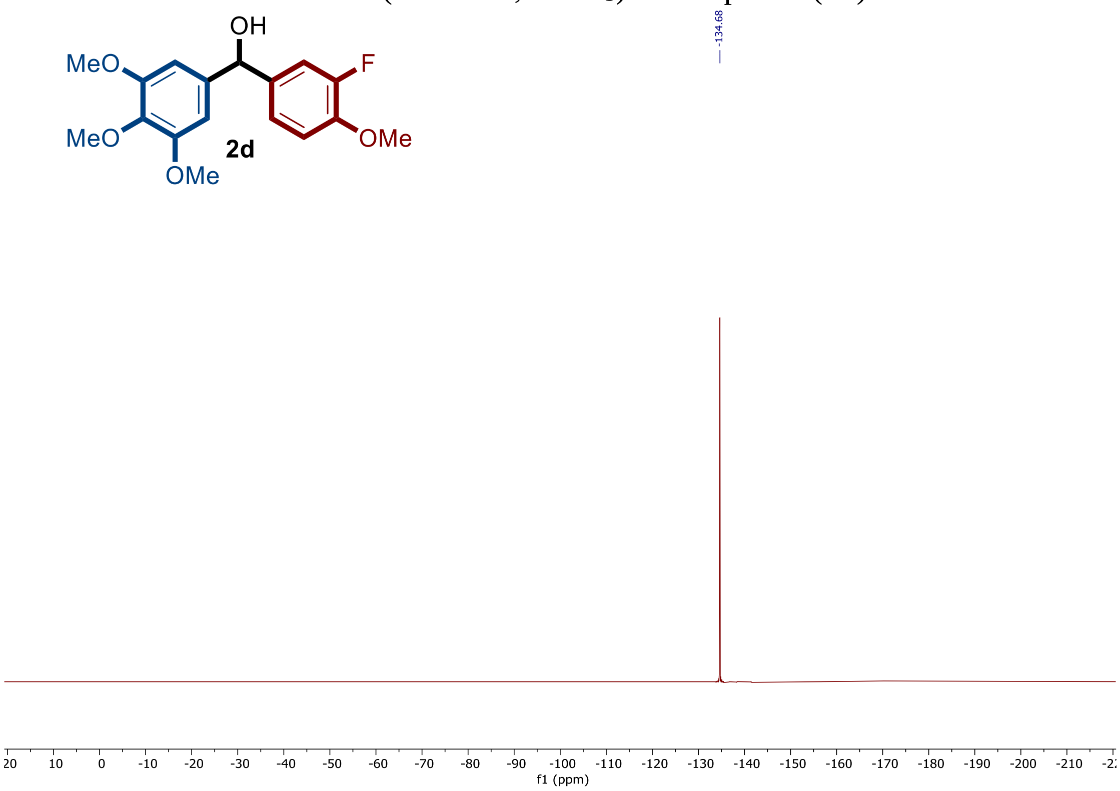

^{1}H NMR (400 MHz, $CDCl_3$) of compound (**11**)

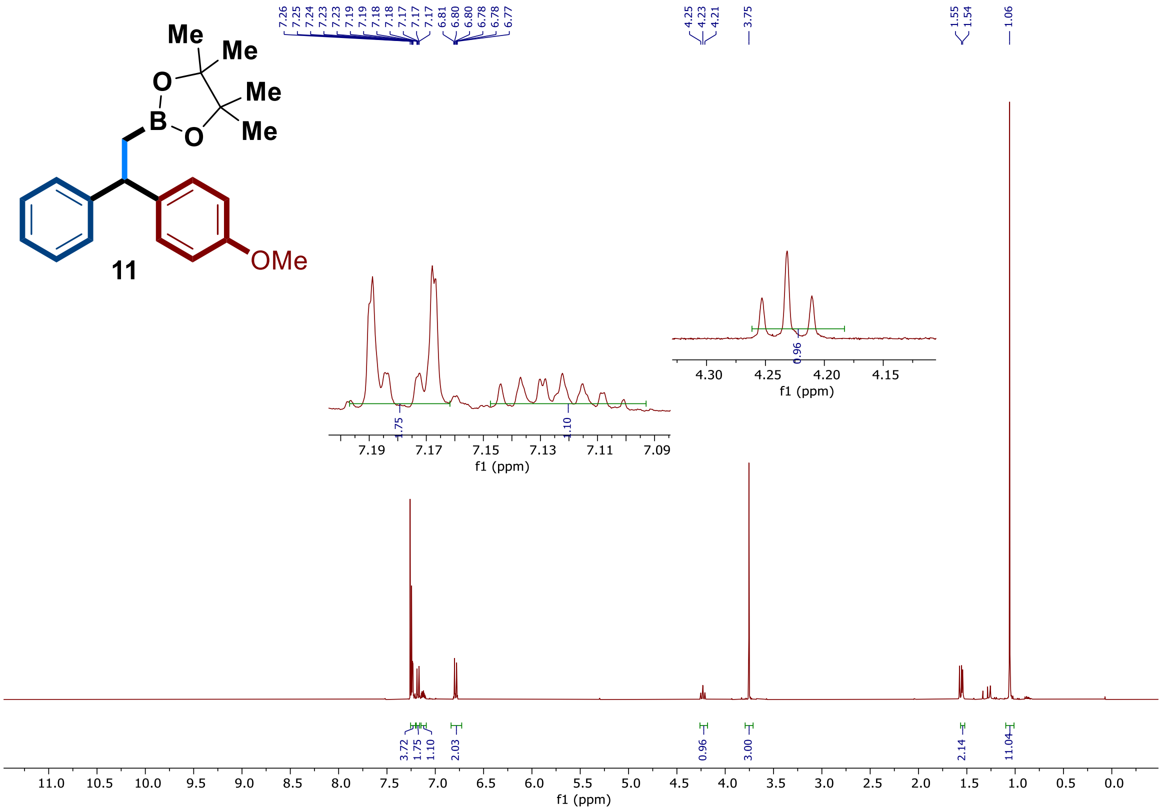

^{1}H NMR (400 MHz, $CDCl_3$) of compound (**12**)

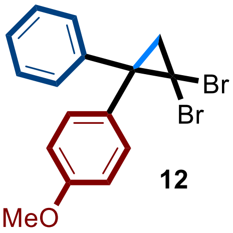

12

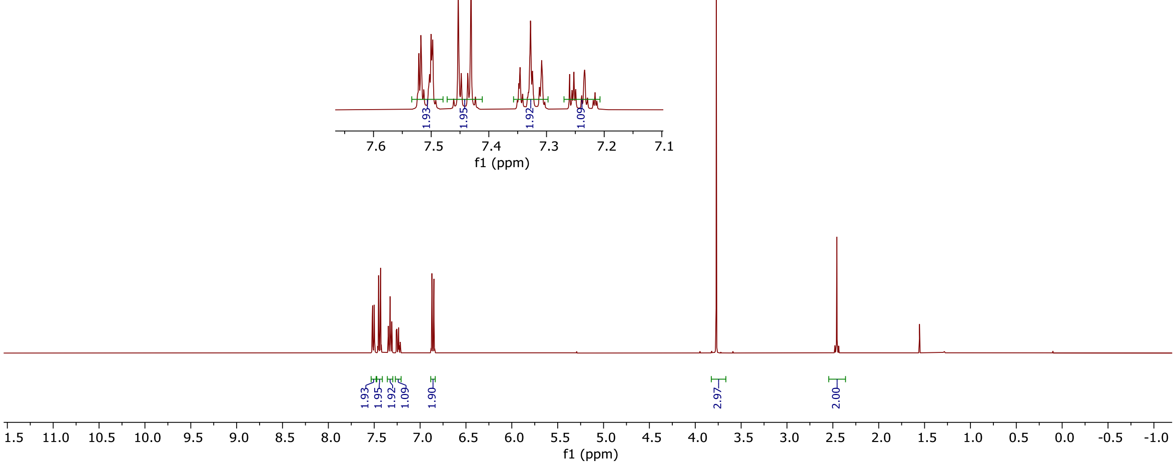

^{1}H NMR (400 MHz, $CDCl_3$) of compound (1**3**)

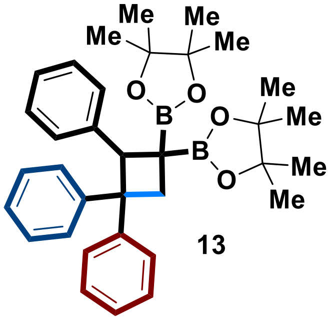

13

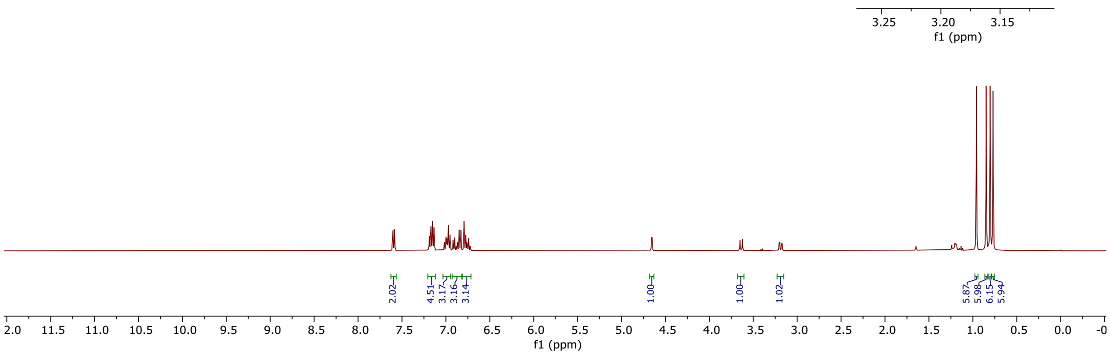

^{1}H NMR (400 MHz, $CDCl_3$) of compound (**14**)

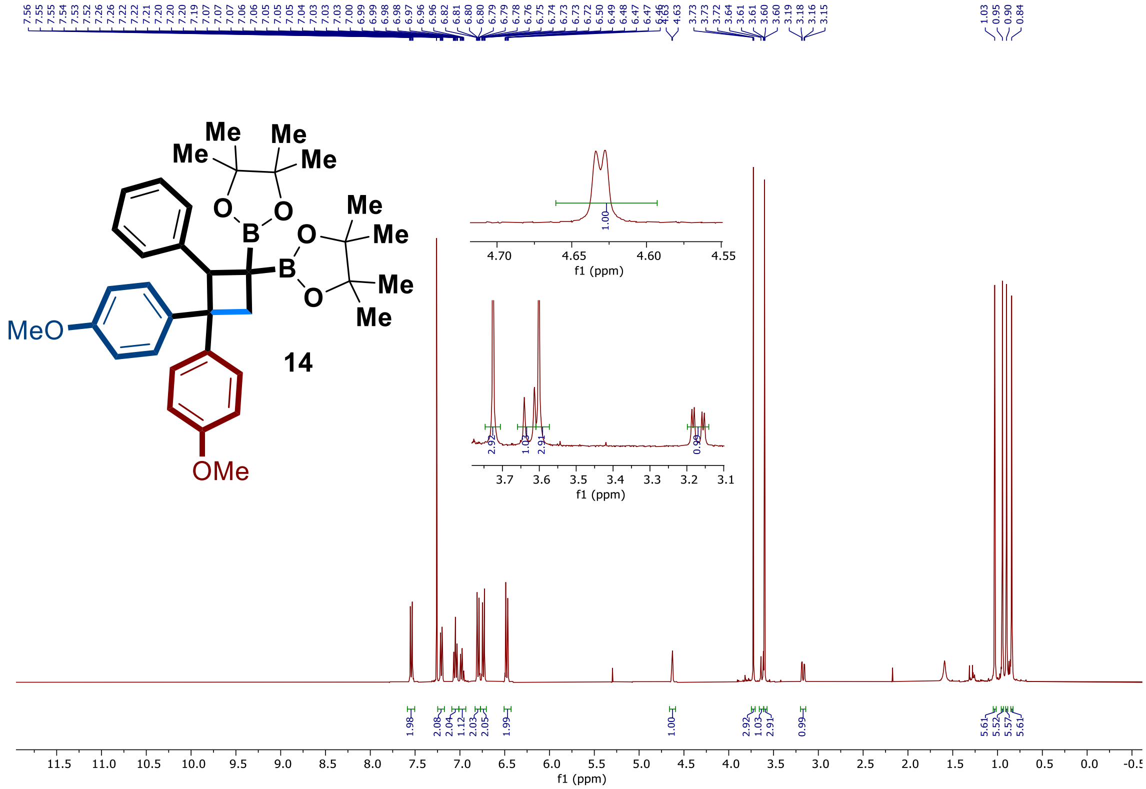

^{1}H NMR (400 MHz, $CDCl_3$) of compound (**15**)

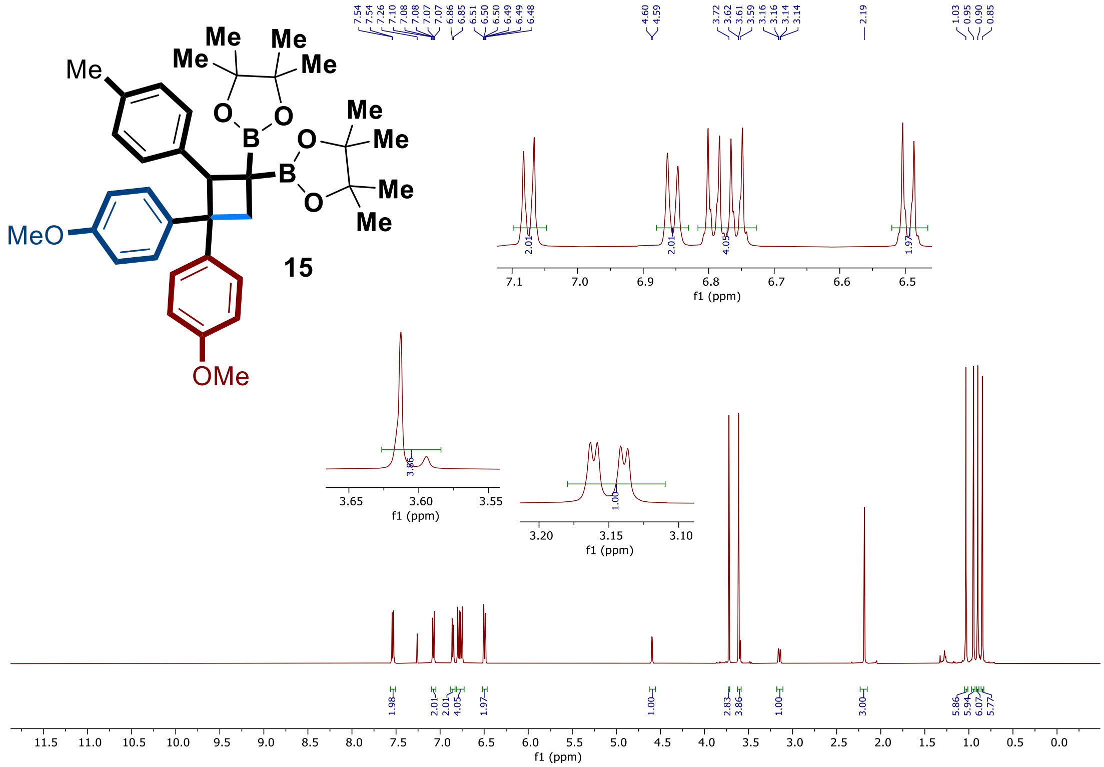

^{1}H NMR (400 MHz, $CDCl_3$) of compound (**16**)

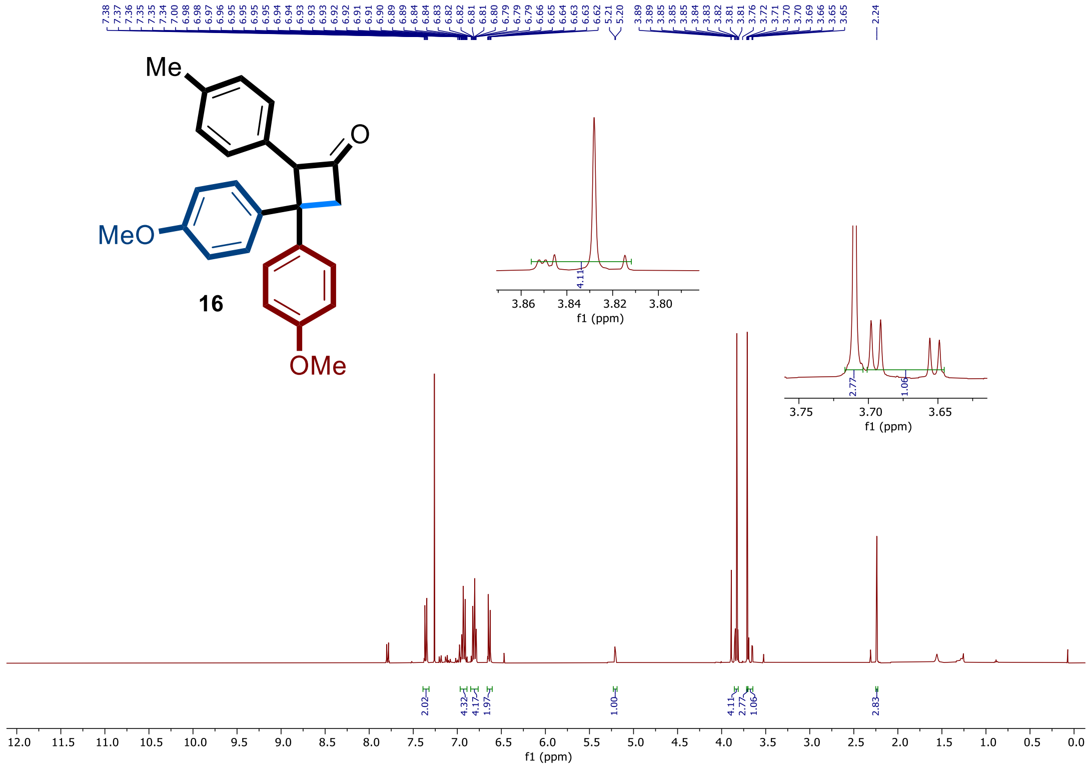

^{1}H NMR (400 MHz, $CDCl_3$) of compound (**17**)

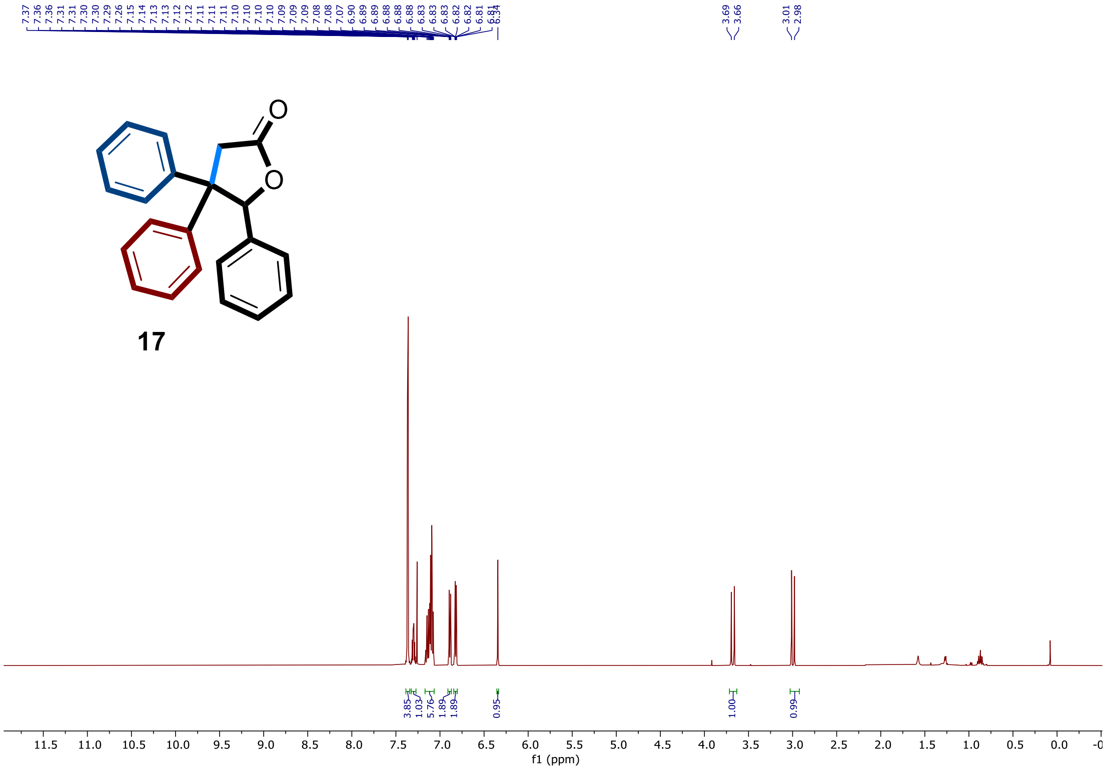

3. References:

(1) Babu, K. N.; Massarwe, F.; Shioukhi, I.; Masarwa, A. Sequential Selective C− H and C (Sp3)−+ P Bond Functionalizations: An Entry to Bioactive Arylated Scaffolds. *Angew. Chem. Int. Ed.* **2021**, *60* (50), 26199–26209.

(2) Gavit, V. R.; Hanania, N.; Eghbarieh, N.; Shioukhi, I.; Masarwa, A. Programmable Strategies for the Conversion of Aldehydes to Unsymmetrical (Deuterated) Diarylmethanes and Diarylketones. *Org. Lett.* **2025**, *27* (14), 3637–3642.

(3) Guo, M. M.; Qin, G. Q.; Jiang, X. Y.; Xu, H.; Ma, M.; Shen, Z. L.; Chu, X. Q. Aerobic Coupling of Organophosphonium Salts with Alkenes: Catalyst-Free C (Sp3)− C (Sp2) Bond Formation. *Adv. Synth. Catal.* **2023**, *365* (11), 1871–1876.

(4) Ji, Y.-F.; Zhang, C.; Hu, Y.-F.; Chen, D.-P.; Chu, X.-Q.; Ge, D. C-+ P to C–P (O) Bond Exchange of Organophosphonium Salts: Synthesis of 3-(Phosphoryl) Methylindoles under Additive-and Catalyst-Free Conditions. *J. Org. Chem.* **2025**, *90* (33), 11780–11790.

(5) Xu, L.; Li, B.-J.; Wu, Z.-H.; Lu, X.-Y.; Guan, B.-T.; Wang, B.-Q.; Zhao, K.-Q.; Shi, Z.-J. Nickel-Catalyzed Efficient and Practical Suzuki− Miyaura Coupling of Alkenyl and Aryl Carbamates with Aryl Boroxines. *Org. Lett.* **2010**, *12* (4), 884–887.

(6) Lamaa, D.; Messe, E.; Gandon, V.; Alami, M.; Hamze, A. Toward a Greener Barluenga–Valdés Cross-Coupling: Microwave-Promoted C–C Bond Formation with a Pd/Peg/H2o Recyclable Catalytic System. *Org. Lett.* **2019**, *21* (21), 8708–8712.

(7) Zou, Y.; Qin, L.; Ren, X.; Lu, Y.; Li, Y.; Zhou, J. Selective Arylation and Vinylation at the A Position of Vinylarenes. *Chem. Eur. J.* **2013**, *19* (10), 3504–3511.

(8) Zhao, X.; Jing, J.; Lu, K.; Zhang, Y.; Wang, J. Pd-Catalyzed Oxidative Cross-Coupling of N-Tosylhydrazones with Arylboronic Acids. *Chem. Commun.* **2010**, *46* (10), 1724–1726.

(9) Roche, M.; Salim, S. M.; Bignon, J.; Levaique, H.; Brion, J.-D.; Alami, M.; Hamze, A. Palladium-Catalyzed One-Pot Reaction of Hydrazones, Dihaloarenes, and Organoboron Reagents: Synthesis and Cytotoxic Activity of 1, 1-Diarylethylene Derivatives. *J. Org. Chem.* **2015**, *80* (13), 6715–6727.

(10) Blons, C.; Mallet-Ladeira, S.; Amgoune, A.; Bourissou, D. (P, C) Cyclometalated Gold (Iii) Complexes: Highly Active Catalysts for the Hydroarylation of Alkynes. *Angew. Chem. Int. Ed.* **2018**, *57* (36), 11732–11736.

(11) Roche, M.; Hamze, A.; Provot, O.; Brion, J.-D.; Alami, M. Synthesis of Ortho/Ortho′-Substituted 1, 1-Diarylethylenes through Cross-Coupling Reactions of Sterically Encumbered Hydrazones and Aryl Halides. *J. Org. Chem.* **2013**, *78* (2), 445–454.

(12) Bhilare, S. V.; Darvatkar, N. B.; Deorukhkar, A. R.; Raut, D. G.; Trivedi, G. K.; Salunkhe, M. M. Synthesis of 1, 1-Diaryl Ethylenes by Cu-Catalyzed Arene C–H Addition to Aryl Acetylenes. *Tetrahedron Letters* **2009**, *50* (8), 893–896.
(13) McLean, E. B.; Cutolo, F. M.; Cassidy, O. J.; Burns, D. J.; Lee, A.-L. Selectivity Control in Gold-Catalyzed Hydroarylation of Alkynes with Indoles: Application to Unsymmetrical Bis (Indolyl) Methanes. *Org. Lett.* **2020**, *22* (17), 6977–6981.
(14) Angeles-Dunham, V. V.; Nickerson, D. M.; Ray, D. M.; Mattson, A. E. Nitrimines as Reagents for Metal-Free Formal C (Sp2)–C (Sp2) Cross-Coupling Reactions. *Angew. Chem. Int. Ed.* **2014**, *53* (52), 14538–14541.
(15) Hayashi, R.; Shimizu, A.; Davies, J. A.; Ishizaki, Y.; Willis, C.; Yoshida, J. i. Metal-and Oxidant-Free Alkenyl C− H/Aromatic C− H Cross-Coupling Using Electrochemically Generated Iodosulfonium Ions. *Angew. Chem. Int. Ed.* **2018**, *57* (39), 12891–12895.
(16) Hamze, A.; Brion, J.-D.; Alami, M. Synthesis of 1, 1-Diarylethylenes Via Efficient Iron/Copper Co-Catalyzed Coupling of 1-Arylvinyl Halides with Grignard Reagents. *Org. Lett.* **2012**, *14* (11), 2782–2785.
(17) Jia, Z.; Luo, S. Visible Light Promoted Direct Deuteration of Alkenes Via Co (Iii)–H Mediated H/D Exchange. *CCS Chem.* **2023**, *5* (5), 1069–1076.
(18) Dong, Z.; Tong, R.-J.; Xu, L.; Xu, H.-J.; Xu, J. Palladium-Catalyzed Regioselective Decarboxylative Hydroarylation of Alkynyl Carboxylic Acids with Arylboronic Acids. *Org. Biomol. Chem.* **2023**, *21* (20), 4220–4224.
(19) Smith, K. B.; Logan, K. M.; You, W.; Brown, M. K. Alkene Carboboration Enabled by Synergistic Catalysis. *Chem. Eur. J.* **2014**, *20* (38), 12032–12036.
(20) Yamazaki, S.; Yamamoto, Y.; Fukushima, Y.; Takebayashi, M.; Ukai, T.; Mikata, Y. Lewis Acid Promoted Reactions of Ethenetricarboxylates with Allenes: Synthesis of Indenes and Γ-Lactones Via Conjugate Addition/Cyclization Reaction. *J. Org. Chem.* **2010**, *75* (15), 5216–5222.
(21) Hanania, N.; Eghbarieh, N.; Masarwa, A. Polyborylated Alkenes as Energy-Transfer Reactive Groups: Access to Multi-Borylated Cyclobutanes Combined with Hydrogen Atom Transfer Event. *Angew. Chem. Int. Ed.* **2024**, *63* (25), e202405898.
